# The Biicosahedral Complex Anions [M(B_11_H_11_)_2_]^3−^ (M = Cu, Ag, Au): Synthesis and Unexpected Low‐Temperature Phase Transition of [Ag(η^5^‐B_11_H_11_)_2_]^3−^ to [Ag(η^2^‐B_11_H_11_)_2_]^3−^

**DOI:** 10.1002/anie.202519283

**Published:** 2025-11-10

**Authors:** Eduard Bernhardt, Tanja Knuplez, Tobias Preitschopf, Andreas Drichel, Björn Beele, Alexey Maximenko, Maik Finze, Adam Slabon

**Affiliations:** ^1^ Chair of Inorganic Chemistry University of Wuppertal Gaußstr. 20 42119 Wuppertal Germany; ^2^ Institut für Anorganische Chemie Institut für nachhaltige Chemie & Katalyse mit Bor (ICB) Julius‐Maximilians‐Universität Würzburg Am Hubland 97074 Würzburg Germany; ^3^ National Synchrotron Radiation Centre SOLARIS Jagiellonian University Kraków, Małopolska Poland

**Keywords:** Boranes, Copper, Gold, Silver, Valence isomerization

## Abstract

We disclose the synthesis and properties of [M(B_11_H_11_)_2_]^3−^ (M = Cu, Ag, Au) with the first gold‐based anion [Au(B_11_H_11_)_2_]^3−^. The *nido*‐[B_11_H_11_]^4−^ ligand stabilizes complexes with copper, silver, and gold in the highest known oxidation state +V. The relative stability of [M^+V^(*nido*‐B_11_X_11_)_2_]^3−^, [M^+III^(*nido*‐B_11_X_11_)(*closo*‐B_11_X_11_)]^3−^, and [M^+I^(*closo*‐B_11_X_11_)_2_]^3−^ isomers (X = H, F) was calculated at the DFT level. Unlike for [Cu(B_11_H_11_)_2_]^3−^ and [Au(B_11_H_11_)_2_]^3−^, in case of [Ag(B_11_H_11_)_2_]^3−^, we discovered the equilibrium between the kinetically stable [Ag^+V^(*nido*‐B_11_H_11_)_2_]^3−^ (η^5^) and kinetically labile [Ag^+I^(*closo*‐B_11_H_11_)_2_]^3−^ (η^2^) by X‐ray diffraction. The isomerization of the Ag complex shows signs of Ag(V)–Ag(I) redox processes. When a crystal is cooled, a phase transition occurs at 110–130 K in which the coordination of the ligands on the silver changes from η^5^ to η^2^. The phase transition is reversible and upon heating to 170 K, the η^2^ to η^5^ coordination is transformed. The reaction of [M(B_11_H_11_)_2_]^3−^ with HF leads to the partial substitution of hydrogen atoms by fluorine atoms to form [M(B_11_H_11−x_F_x_)_2_]^3−^ (M = Cu, Au; x ≈ 4). We synthesized 18 compounds containing [M(B_11_H_11_)_2_]^3−^ (M = Cu, Ag, Au) and their derivatives, followed by structural determination (X‐ray crystallography). XANES data confirm the high oxidation state of Cu in K_3_[Cu(B_11_H_11_)_2_]·5H_2_O.

## Graphical abstract

The unusually high oxidation state +V of Cu, Ag, and Au is stabilized by [B_11_H_11_]^4–^‐based anions. For (*n*‐Bu_4_N)_3_[Ag(B_11_H_11_)_2_], the crystal structures were determined for both isomers with Ag^+I^ and Ag^+V^.

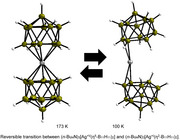

## Introduction

Boron clusters^[^ ^1^, ^2^, ^3^, ^4^, ^5^, ^6^ ^]^ are used in medicine, for example in BNCT^[^ ^7^, ^8^, ^9^ ^]^ or as antibiotics,^[^ ^10^ ^]^ membrane carriers,^[^ ^11^ ^]^ and pharmacophores.^[^ ^12^, ^13^ ^]^ They are also being discussed for other applications, such as selective extraction of ^137^Cs from radioactive waste, photoredox behavior, as weakly coordinating ions, in ionic liquids, or materials with cationic conductivity.^[^ ^8^, ^14^, ^15^, ^16^, ^17^, ^18^, ^19^, ^20^ ^]^ For example, the use of Li_2_[B_12_F_12_] in lithium batteries has certain advantages^[^ ^21^, ^22^, ^23^, ^24^, ^25^ ^]^ and a mixed electrolyte composed of Na_2_[B_10_H_10_]_0.5_[B_12_H_12_]_0.5_,^[^ ^26^, ^27^ ^]^ Na_5_(B_11_H_14_)(B_12_H_12_)_2_,^[^ ^18^ ^]^ or Li_2_(B_11_H_14_)(CB_11_H_12_)^[^ ^16^ ^]^ reveals promising properties for solid state sodium or lithium batteries.

The basic structures of boron clusters are the *closo*‐borates [B_n_H_n_]^2−^,^[^ ^28^, ^29^, ^30^, ^31^, ^32^, ^33^ ^]^ of which those with *n* = 6–12 have been synthesized.^[^ ^34^ ^]^ Of these, *closo*‐[B_12_H_12_]^2−^ is the most stable.^[^ ^35^ ^]^ Removal of a BH^2+^ unit from *closo*‐[B_n_H_n_]^2−^ leads to *nido*‐[B_n−1_H_n−1_]^4−^, which is also formed by the reduction of *closo*‐[B_n−1_H_n−1_]^2−^.^[^ ^11^ ^]^ Due to the relative instability of *closo*‐[B_11_H_11_]^2−^ compared to *closo*‐[B_12_H_12_]^2−^, *nido*‐[B_11_H_11_]^4−^ is relatively stable against oxidation compared to other *nido*‐[B_n_H_n_]^4−^ clusters (*n* = 5–10).^[^ ^36^, ^37^ ^]^ The *nido*‐[B_11_H_11_]^4−^ ion is an *η* ^5^ ligand with six valence electrons that can coordinate to transition metals and main group elements similar to the cyclopentadienyl anion C_5_H_5_^−^.^[^ ^38^ ^]^

**Scheme 1:**
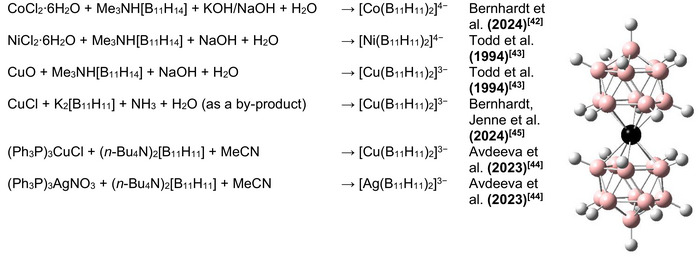
Summary of known syntheses of [M(B_11_H_11_)_2_]^n−^ (M = Co, Ni, Cu, Ag; *n* = 3, 4) that have been reported in the literature so far. Crystal structures: (*n*‐Bu_4_N)_3_[M(B_11_H_11_)_2_] (M = Cu^[^ ^43^ ^]^ and Ag^[^ ^44^ ^]^); M_4_[Co(B_11_H_11−x_(OH)_x_)_2_]·yH_2_O (M/x/y = K/1.81/2; Cs/0.89/4.56) and crystal structures of other derivatives containing the same anionic structure units.^[^ ^42^ ^]^

An overview of the chemistry of the [B_11_H_14_]^−^ and [B_11_H_11_]^2−^ ions was published in 2003.^[^ ^36^, ^37^, ^39^, ^40^, ^41^ ^]^ Many main group element compounds of the type [B_11_X_11_ML_x_]^n−^ are known that contain the [B_11_H_11_]^4−^ ligand (X = H, halogens and other ligands; L possibly additional ligands; *n* charge).^[^ ^42^ ^]^ On the other hand, only few complexes of the type [M(B_11_X_11_)_2_]^n−^ are known that contain either cobalt, nickel, copper, or silver as central metal atom (Scheme 1).^[^ ^42^, ^43^, ^44^ ^]^

For gold, neither complexes of the type [B_11_X_11_ML_x_]^n−^ nor complexes of the type [M(B_11_X_11_)_2_]^n−^ have been described. The [B_11_H_11_]^4−^ ligand is electron‐rich and stabilizes unusually high formal oxidation states for the transition metals (Co(+IV), Ni(+IV), Cu(+V), and Ag(+V)).^[^ ^42^, ^43^, ^44^ ^]^ Hirschfeld surface analyses and DFT calculations for [M(B_11_H_11_)_2_]^3−^ (M = Cu, Ag) were previously reported.^[^ ^44^, ^45^ ^]^ Such high oxidation states are only achievable for Ni(+IV) (K_2_[NiF_6_]),^[^ ^46^, ^47^, ^48^ ^]^ Cu(+IV) (Cs_2_[CuF_6_]),^[^ ^49^, ^50^, ^51^ ^]^ Ag(+V) (Cs_4_[GaF_6_][AgF_6_]),^[^ ^52^ ^]^ and Au(+V) (Cs[AuF_6_])^[^ ^48^, ^53^, ^54^, ^55^ ^]^ with the fluorido ligand. It should be noted that these high oxidation states are not typical for copper, silver, and gold. Compounds with these metals are usually found in the following oxidation states: Cu(+II), Ag(+I), and Au(+III).^[^ ^56^, ^57^ ^]^ In opposite to copper and gold, compounds with silver in the oxidation states +II and +III are very strong oxidizing agents.^[^ ^56^, ^57^ ^]^ In contrast to the corresponding compounds of Ni(+IV), Cu(+IV), and Ag(+V) with fluorido ligands, [Ni(B_11_H_11_)_2_]^4−^ and especially [Cu(B_11_H_11_)_2_]^3−^ are stable in aqueous solution at room temperature in air.^[^ ^43^, ^44^, ^58^ ^]^ Similar to how a strong [B_11_H_11_]^4−^ ligand stabilizes the +V oxidation state in copper, silver, and gold, a strong CF_3_^−^ ligand stabilizes the +III oxidation state in [M(CF_3_)_4_]^−^ (M = Cu, Ag, Au), which are also stable in water at room temperature.^[^ ^59^, ^60^ ^]^ Our theoretical calculations (*vide infra*) show that the anion [Au(B_11_H_11_)_2_]^3−^ should be significantly more stable than the corresponding copper and silver counterpart.

So far, no derivatives of [M(B_11_H_11_)_2_]^3−^ (M = Cu, Ag and Au) are known in which the hydrogen atoms are substituted by other atoms. Due to the different size of the alkali metal ions and [M(B_11_X_11_)_2_]^3−^ (M = Cu, Ag, and Au) and the weak ion–ion interactions, salts of the type A_3_[M(B_11_X_11_)_2_] (A = Li, Na, K, Rb, Cs) are of interest as potential ion conductors.^[^ ^8^, ^14^, ^15^, ^16^, ^17^, ^18^, ^19^, ^20^, ^26^, ^27^, ^61^ ^]^ In addition, [M(B_11_F_11_)_2_]^3−^ could be a weakly coordinating anion albeit its high negative charge.^[^ ^62^, ^63^, ^64^ ^]^

**Scheme 2:**
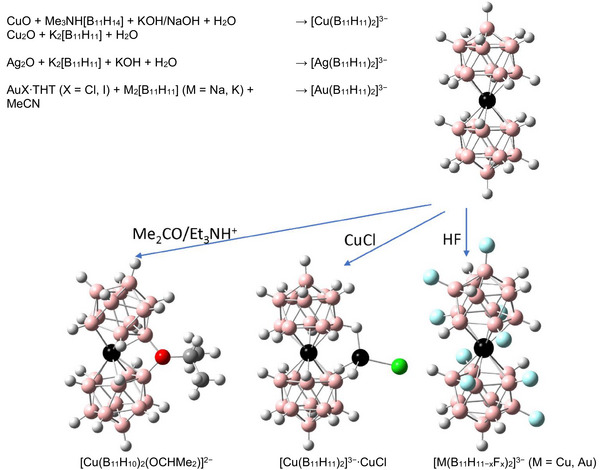
Summary of the syntheses demonstrated in this work (the calculated structures are shown.).

The aim of the present work was the synthesis of compounds exhibiting the complex anion [Au(B_11_H_11_)_2_]^3−^ and derivatives of [M(B_11_H_11_)_2_]^3−^ (M = Cu, Ag and Au). The syntheses conducted in the course of this project are summarized in Scheme 2. During our study, we discovered an unexpected low temperature phase transition for (*n*‐Bu_4_N)_3_[Ag(B_11_H_11_)_2_] that corresponds to an isomerization of the complex anion from [Ag(η^5^‐B_11_H_11_)_2_]^3−^ to [Ag(η^2^‐B_11_H_11_)_2_]^3−^ at lower temperature. To the best of our knowledge, this is the first example of such an isomerization for this class of compounds.^[^ ^65^ ^]^

## Results and Discussion

### Syntheses of the Anion [M(B_11_H_11_)_2_]^3−^ (M = Cu, Ag, Au)

The syntheses presented in this contribution are summarized in Scheme 2. The salts of [Cu(B_11_H_11_)_2_]^3−^ are formed by the reaction of [B_11_H_14_]^−^, or [B_11_H_13_]^2−^, with various copper reagents (Cu_2_O, CuCl, CuO, or CuCl_2_) in aqueous solutions in the presence of NaOH.^[^ ^43^ ^]^

$${\left[B_{11}H_{14}\right]}^{-}+{\text{OH}}^{-}\to {\left[B_{11}H_{13}\right]}^{2-}+H_{2}O$$

$$2{\left[B_{11}H_{13}\right]}^{2-}+\text{CuO}\to {\left[\text{Cu}{\left(B_{11}H_{11}\right)}_{2}\right]}^{3-}+3/2H_{2}+{\text{OH}}^{-}$$

$$2{\left[B_{11}H_{13}\right]}^{2-}+\text{CuCl}\to {\left[\text{Cu}{\left(B_{11}H_{11}\right)}_{2}\right]}^{3-}+2H_{2}+{\text{Cl}}^{-}$$

[Cu(B_11_H_11_)_2_]^3−^ forms as a side‐product during the copper‐catalyzed synthesis of [RC(O)CH_2_CB_11_H_11_]^−^.^[^ ^58^ ^]^ [Cu(B_11_H_11_)_2_]^3−^ is also formed from [B_11_H_11_]^2−^ and (Ph_3_P)_3_CuCl in acetonitrile.^[^ ^44^ ^]^

$$2{\left[B_{11}H_{11}\right]}^{2-}+{\left({\text{Ph}}_{3}P\right)}_{3}\text{CuCl}\to {\left[\text{Cu}{\left(B_{11}H_{11}\right)}_{2}\right]}^{3-}+\cdots$$

So far, only the tetrabutylammonium salt (*n*‐Bu_4_N)_3_[Cu(B_11_H_11_)_2_] was obtained by these methods.^[^ ^43^, ^44^ ^]^ We also observed that [Cu(B_11_H_11_)_2_]^3−^ is formed in the reactions of [B_11_H_14_]^−^, [B_11_H_13_]^2−^, or [B_11_H_11_]^2−^ with different copper compounds (Cu_2_O, CuCl, CuI, CuO, or CuCl_2_) both in acetonitrile (with Et_3_N as a base) and in aqueous solutions in the presence of NaOH, KOH, or NH_3_. Interestingly, CuCN does not react with [B_11_H_14_]^−^, [B_11_H_13_]^2−^, or [B_11_H_11_]^2−^ to form [Cu(B_11_H_11_)_2_]^3−^. During the synthesis of [Cu(B_11_H_11_)_2_]^3−^, it is advantageous to use salts of [B_11_H_14_]^−^, since these are more readily available. In this work, we were able to prepare water‐soluble sodium and potassium salts of [Cu(B_11_H_11_)_2_]^3−^. These salts are also soluble in acetonitrile, acetone, and other polar organic solvents. Using these salts as starting materials, salts with other cations (e.g., *n*‐Bu_4_N^+^, NH_4_^+^, Bpy_4_^3+^, [Co(NH_3_)_6_]^3+^, and others) can be readily prepared by metathesis.

The synthesis of (*n*‐Bu_4_N)_3_[Ag(B_11_H_11_)_2_] was achieved by the reaction of (*n*‐Bu_4_N)_2_[B_11_H_11_] with (Ph_3_P)_3_AgNO_3_ in acetonitrile.^[^ ^44^ ^]^

$${\left({\text{Ph}}_{3}P\right)}_{3}{\text{AgNO}}_{3}+2{\left[B_{11}H_{11}\right]}^{2-}\to {\left[\text{Ag}{\left(B_{11}H_{11}\right)}_{2}\right]}^{3-}+\cdots$$

We observed that the reaction of Ag_2_O with [B_11_H_14_]^−^ ([B_11_H_13_]^2−^) in aqueous NaOH or KOH solution resulted in the formation of [Ag(B_11_H_11_)_2_]^3−^, which was clearly evident from the orange color of the solution. [Ag(B_11_H_11_)_2_]^3−^ reacted rapidly with [B_11_H_13_]^2−^ to form elemental silver, and the orange color of the solution disappeared after a few minutes. The reaction of stoichiometric amounts of K_2_[B_11_H_11_] and Ag_2_O in aqueous KOH solution led to the quantitative formation of [Ag(B_11_H_11_)_2_]^3−^, which was isolated as K_3_[Ag(B_11_H_11_)_2_]·5H_2_O. K_3_[Ag(B_11_H_11_)_2_]·5H_2_O readily releases some of the water to form K_3_[Ag(B_11_H_11_)_2_]·2H_2_O. Upon addition of K_2_CO_3_, K_3_[Ag(B_11_H_11_)_2_]·2H_2_O crystallizes directly from the aqueous solution at room temperature. Compared to [Cu(B_11_H_11_)_2_]^3−^ and [Au(B_11_H_11_)_2_]^3−^, [Ag(B_11_H_11_)_2_]^3−^ is significantly less stable. Thus, [Ag(B_11_H_11_)_2_]^3−^ is rapidly decomposed not only by [B_11_H_13_]^2−^ (see above), but also by sulfide and iodide ions:

$$2{\left[\text{Ag}{\left(B_{11}H_{11}\right)}_{2}\right]}^{3-}+S^{2-}\to 4{\left[B_{11}H_{11}\right]}^{2-}+{\text{Ag}}_{2}S↓$$

Neither [Cu(B_11_H_11_)_2_]^3−^ nor [Au(B_11_H_11_)_2_]^3−^ react with [B_11_H_13_]^2−^, Cl^−^, Br^−^, I^−^, or S^2−^ at room temperature. In contrast to [Cu(B_11_H_11_)_2_]^3−^ and [Au(B_11_H_11_)_2_]^3−^, in the case of [Ag(B_11_H_11_)_2_]^3−^ the equilibrium between kinetically stable [Ag^+V^(*nido*‐B_11_H_11_)_2_]^3−^ and kinetically labile [Ag^+I^(*closo*‐B_11_H_11_)_2_]^3−^ (*vide infra*) is evident.

Although [Au(B_11_H_11_)_2_]^3−^ is the most stable complex in the series [M(B_11_H_11_)_2_]^3−^ (M = Cu, Ag, Au), its synthesis is complicated by the easy reducibility of gold in the starting compounds. Thus, the reactions of [AuCl_4_]^−^, AuI, and Au_2_O_3_ with [B_11_H_14_]^−^, [B_11_H_13_]^2−^, or [B_11_H_11_]^2−^ in aqueous solution lead to the quantitative formation of elemental gold. AuCN does not react with [B_11_H_14_]^−^, [B_11_H_13_]^2−^, or [B_11_H_11_]^2−^ in aqueous solution to form [Au(B_11_H_11_)_2_]^3−^. The reaction of AuX·THT (X = Cl, I) in acetonitrile solution with M_2_[B_11_H_11_] (M = Na, K) leads to the formation of [Au(B_11_H_11_)_2_]^3−^ in acceptable yields (40%–60%).

$$\text{AuX}\cdot \text{THT}+2{\left[B_{11}H_{11}\right]}^{2-}\to {\left[\text{Au}{\left(B_{11}H_{11}\right)}_{2}\right]}^{3-}+\text{THT}+X^{-}$$

However, elemental gold, also in colloidal form, is formed as a byproduct in 30%–60% yield. [Au(B_11_H_11_)_2_]^3−^ is very stable and does not react with S^2−^, Cl^−^, Br^−^, I^−^, or [B_11_H_13_]^2−^. Salts with other cations can be synthesized from the resulting sodium and potassium salts by metathesis reactions.

### UV–Vis‐Spectra of [M(B_11_H_11_)_2_]^3−^ (M = Cu, Ag, Au)

**Figure 1:**
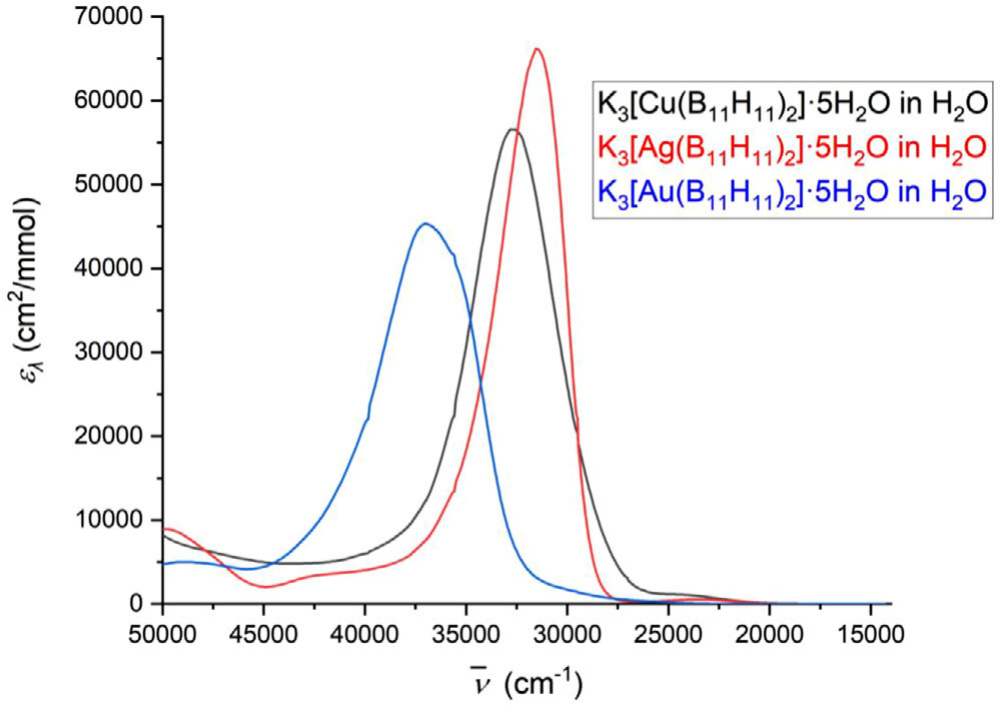
UV–vis spectra of K_3_[M(B_11_H_11_)_2_] (M = Cu, Ag, Au) in an acetonitrile solution.

While [Cu(B_11_H_11_)_2_]^3−^ and [Ag(B_11_H_11_)_2_]^3−^ are orange, [Au(B_11_H_11_)_2_]^3−^ is bright yellow. The difference is clearly visible in the UV–vis spectra (Figures 1, S16–S26, Table S4a). According to DFT calculations, the strong band at [Cu(B_11_H_11_)_2_]^3−^, [Ag(B_11_H_11_)_2_]^3−^, and [Au(B_11_H_11_)_2_]^3−^ and the relatively weak shoulder at [Cu(B_11_H_11_)_2_]^3−^ and [Au(B_11_H_11_)_2_]^3−^ and the weak band at [Ag(B_11_H_11_)_2_]^3−^ can be assigned to the transition of electrons from the B─M bond (*E*_1_ *_u_*) to the empty antibonding *d_xz_*, *d_yz_* orbitals (*E*_1_ *_g_*) (*E*_1_ *_u_* → *E*_1_ *_g_*: *A*_1_ *_u_* (weak), *E*_2_ *_u_* (weak) *A*_2_ *_u_* (strong)). The *E*_2u_ and *A*_1u_ transitions may gain their intensity from spin‐orbit coupling. In the case of [Cu(B_11_H_11_)_2_]^3−^, there is a transition (*E*_2_ *_u_*/*A*_2_ *_u_* → *E*_1_ *_g_*: *E*_1_ *_u_*) of relatively weak intensity, which also partially overlaps with *E*_2_ *_u_*/*A*_1_ *_u_*. This leads to *E*_2_ *_u_*/*A*_1_ *_u_* being observed only as a shoulder despite its relatively large distance from the strong band. The experimentally (Figures 1, S16–S26, Table S4a) and theoretically calculated (Figures S28–S42, Tables S6–S11) distance between *A*_2_ *_u_* and *E*_2_ *_u_*/*A*_1_ *_u_* decreases in the sequence [Cu(B_11_H_11_)_2_]^3−^ > [Ag(B_11_H_11_)_2_]^3−^ > [Au(B_11_H_11_)_2_]^3−^. Further discussion of the UV–vis spectra can be found in the Supporting Information.

### Crystal Structures with the Anion [M(B_11_H_11_)_2_]^3−^ (M = Cu, Ag, Au)

The previously published crystal structures of (*n*‐Bu_4_N)_3_[M(B_11_H_11_)_2_] (M = Cu,^[^ ^43^ ^]^ Ag^[^ ^44^ ^]^) are not of very good quality, which is due to the partially disordered *n*‐Bu_4_N^+^ cations. Furthermore, disordered solvent molecules are incorporated into the crystal structures. For this reason, we investigated the crystal structures with less problematic cations such as K^+^, Na^+^, Bpy_4_^3+^, [Co(NH_3_)_6_]^3+^, and Et_3_NH^+^. For salts containing cesium cations, the quality of the crystal structures is significantly poorer due to disorder. This is likely due to the relatively weak interaction between Cs^+^ and [M(B_11_H_11_)_2_]^3−^ and the much larger size of [M(B_11_H_11_)_2_]^3−^ compared to the Cs^+^ cations.

**Figure 2:**
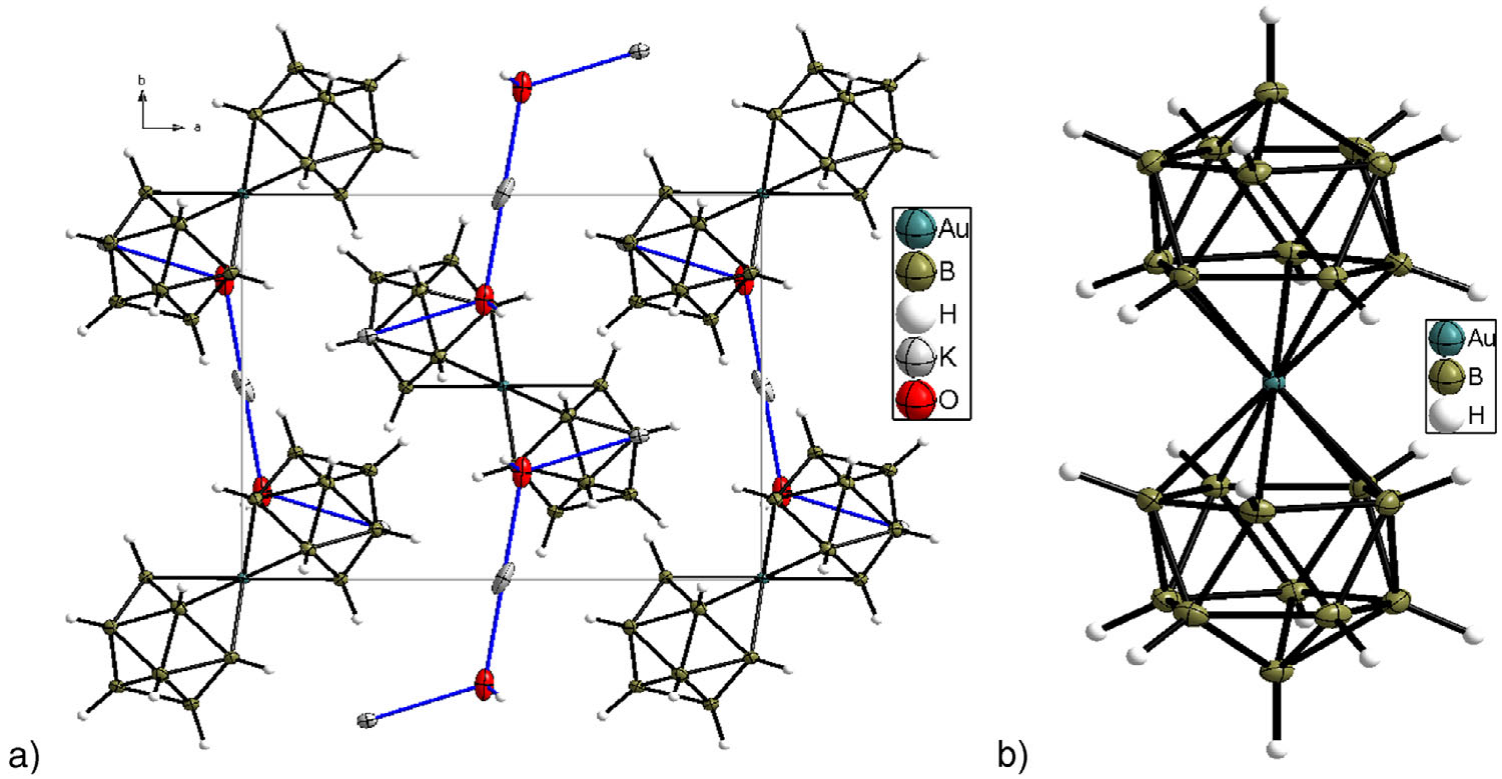
Structure of the cell a) and anions b) in K_3_[Au(B_11_H_11_)_2_]·2H_2_O at 100 K. Thermal ellipsoids are drawn at 50% probability.

We obtained the best crystal structures with the [M(B_11_H_11_)_2_]^3−^ anions for K_3_[M(B_11_H_11_)_2_]·2H_2_O (M = Cu, Ag, and Au) (Figures 2 and S2). In the case of M = Cu and Ag, K_3_[M(B_11_H_11_)_2_]·2H_2_O was crystallized from water at room temperature (22 °C) in the presence of K_2_CO_3_, as otherwise K_3_[M(B_11_H_11_)_2_]·5H_2_O (M = Cu and Ag) crystallized.

Regarding the compounds with multiply charged cations, single crystals of compounds with the cations Bpy_4_^3+^ and [Co(NH_3_)_6_]^3+^ were obtained. Bpy_4_[M(B_11_H_11_)_2_] (M = Cu, Au) is practically insoluble in water and most organic solvents. Suitable single crystals of Bpy_4_[M(B_11_H_11_)_2_] were obtained by slow evaporation of the solvent from the solution in dimethyl sulfoxide.

### XANES Data of K_3_[Cu(B_11_H_11_)_2_]·5H_2_O

**Figure 3:**
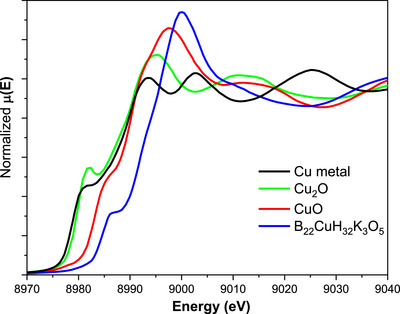
XANES spectra at the Cu K‐edge for the K_3_[Cu(B_11_H_11_)_2_]·5H_2_O, along with the corresponding reference samples.

The electronic structure and local geometry of K_3_[Cu(B_11_H_11_)_2_]·5H_2_O were investigated by analyzing the XANES region of the Cu K‐edge (Cu(1s)) X‐ray absorption spectra (Figure 3). The Cu K‐edge absorption edge for K_3_[Cu(B_11_H_11_)_2_]·5H_2_O, determined at the half‐height of the edge step, appears at 8991.1 eV more than 5 eV higher than for Cu_2_O (8984.6 eV) and CuO (8985.9 eV). Typically, the K‐edge position is sensitive to the oxidation state and local electron density at the copper site. A shift of more than 5 eV is unusual among copper compounds and is strong evidence of an exceptionally high oxidation state Cu environment in this boron‐rich cluster. These data are consistent with the X‐ray photoelectron spectrum data^[^ ^43^ ^]^ (Cu(2p_3/2_), SI). This observation is in agreement with the description of the coinage metal complexes [M(B_11_H_11_)_2_]^3−^ (M = Cu, Ag, Au) in the unusual formal oxidation state +V.

### NMR Data of [M(B_11_H_11_)_2_]^3−^ (M = Cu, Ag, Au)

**Figure 4:**
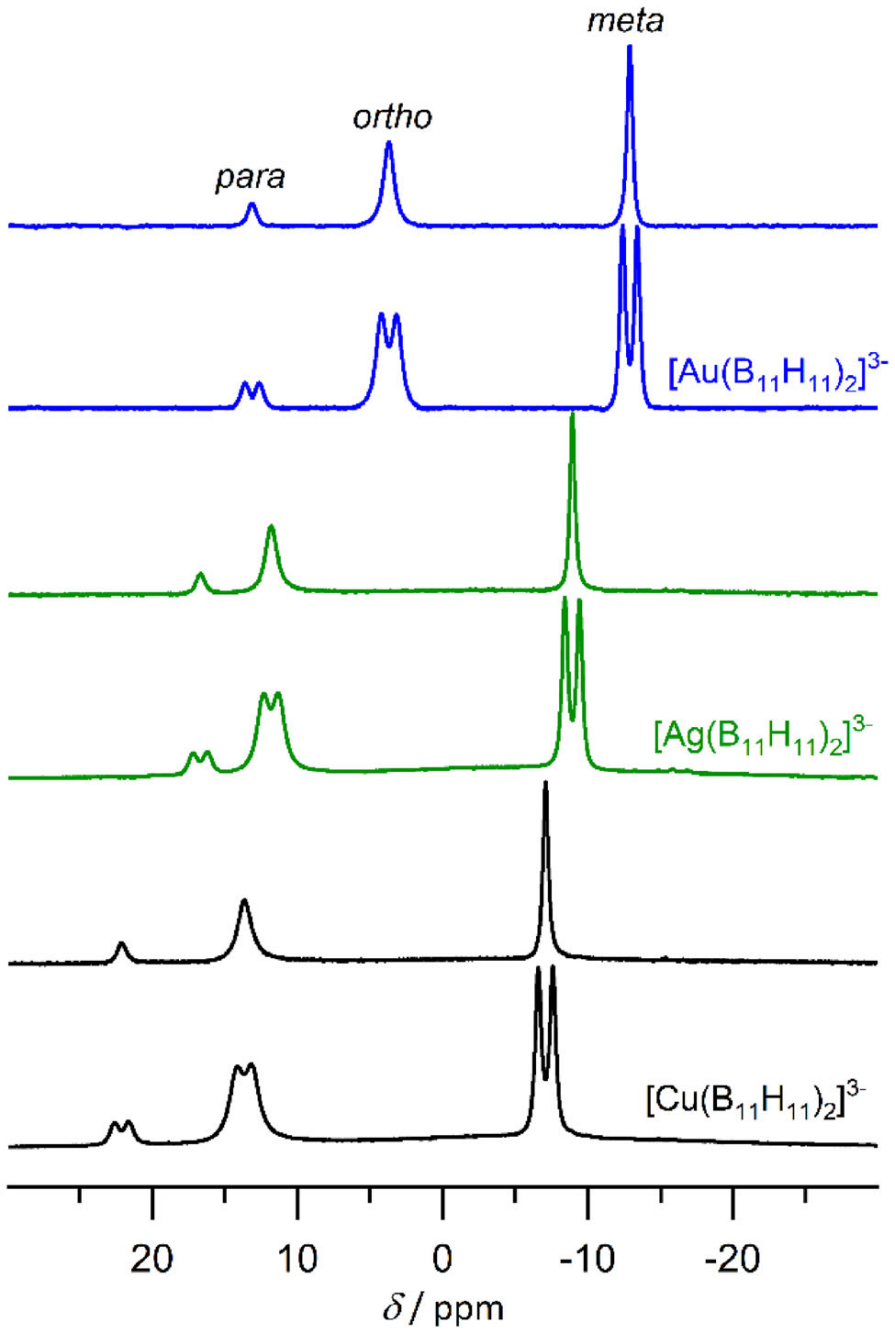
^11^B and ^11^B{^1^H} NMR spectra of [Cu(B_11_H_11_)_2_]^3−^ (black), [Ag(B_11_H_11_)_2_]^3−^ (green) and [Au(B_11_H_11_)_2_]^3−^ (blue) in CD_3_CN.

The ^11^B and ^11^B{^1^H} NMR spectra of [M(B_11_H_11_)_2_]^3−^ (M = Cu, Ag, Au) in CD_3_CN are shown in Figure 4. The NMR data of [M(B_11_H_11_)_2_]^3−^ (M = Cu, Ag, Au) are in Table S2. All further spectroscopic data (NMR and HRMS ESI) are provided in the Tables S2a–S4.

### Low‐Temperature Phase Transition of [Ag(η^5^‐B_11_H_11_)_2_]^3−^ to [Ag(η^2^‐B_11_H_11_)_2_]^3−^

**Figure 5:**
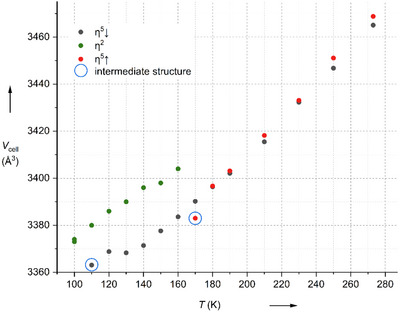
Change in cell volume of (*n*‐Bu_4_N)_3_[Ag(B_11_H_11_)_2_] with temperature.

**Figure 6:**
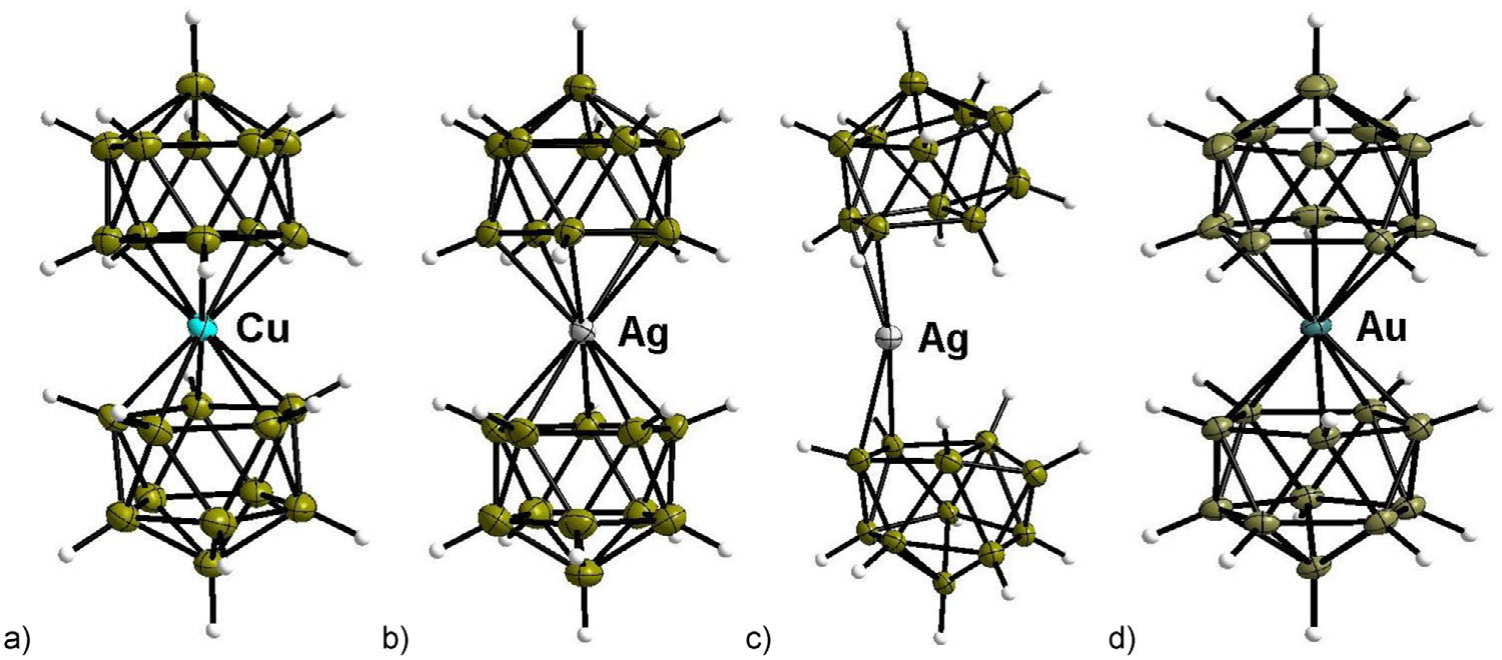
Crystal structures of anions: a) (*n*‐Bu_4_N)_3_[Cu(η^5^‐B_11_H_11_)_2_] (100 K), b) (*n*‐Bu_4_N)_3_[Ag(η^5^‐B_11_H_11_)_2_] (173 K), c) (*n*‐Bu_4_N)_3_[Ag(η^2^‐B_11_H_11_)_2_] (100 K,) and d) (*n*‐Bu_4_N)_3_[Au(η^5^‐B_11_H_11_)_2_] (100 K): Ellipsoids are drawn at 50% probability except for H atoms, which are depicted with arbitrary radii. The metal–boron bond lengths are in (*n*‐Bu_4_N)_3_[Cu(η^5^‐B_11_H_11_)_2_] 2.147(3)−2.166(3) Å (av. 2.157(7) Å), (*n*‐Bu_4_N)_3_[Ag(η^5^‐B_11_H_11_)_2_] 2.278(2)−2.291(2) Å (av. 2.283(5) Å), (*n*‐Bu_4_N)_3_[Ag(η^2^‐B_11_H_11_)_2_] B_7,8_: 2.343(3)−2.391(3), B_9,11_: 2.767(3)−2.954(4), B_10_: 3.769(3)−3.856(4) Å (av. 2.371(22)*4, 2.842(84)*4, 3.812(61)*2 Å) and (*n*‐Bu_4_N)_3_[Au(η^5^‐B_11_H_11_)_2_] 2.–57(3)‐2.276(3) Å (av. 2.266(6) Å).

**Figure 7:**
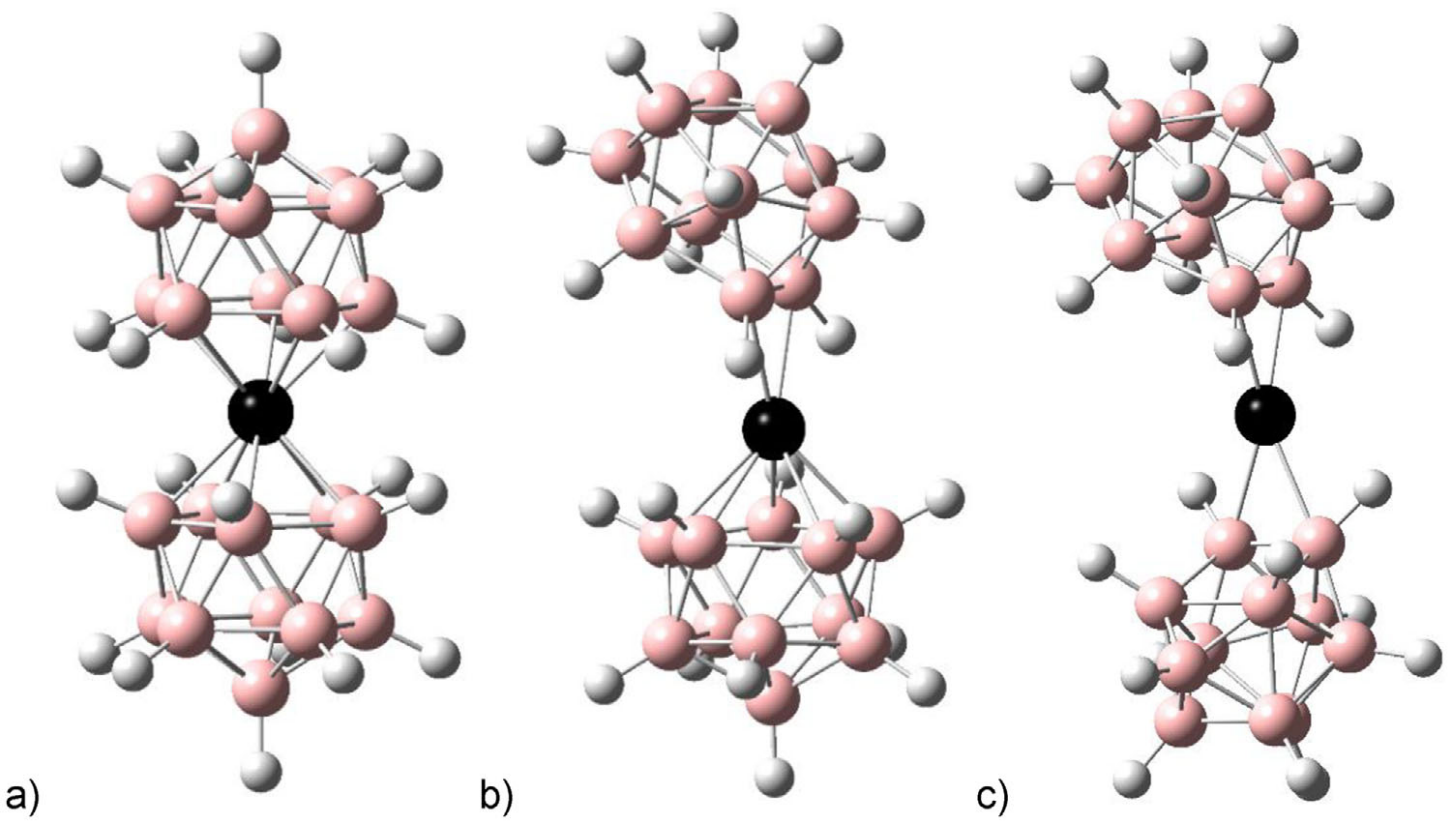
Structures of the isomers of [M(B_11_H_11_)_2_]^3−^ (M = Cu, Ag, Au): a) [M^+V^(*nido*‐B_11_H_11_)_2_]^3−^ (d^6^ complex), b) [M^+III^(*nido*‐B_11_H_11_)(*closo*‐B_11_H_11_)]^3−^ (d^8^ complex) and c) [M^+I^(*closo*‐B_11_H_11_)_2_]^3−^ (d^10^ complex).

The NMR data of (*n*‐Bu_4_N)_3_[Ag(B_11_H_11_)_2_] suggests an η^5^‐coordination mode of Ag^+V^ as it seems to be highly symmetrical, similar to the compounds containing copper and gold. After crystallizing (*n*‐Bu_4_N)_3_[Ag(B_11_H_11_)_2_] large, orange single crystals were studied with single crystal X‐ray diffraction at various temperatures. The phase transition was kinetically inhibited and started only with a delay. Once initiated, it occurred abruptly, often causing crystals to burst. Therefore, a suitable crystal was cooled gradually, and the crystal structure was determined at various temperatures (Figure 5). The temperature was lowered to 100 K and the unit cell slightly changed. The structure revealed a silver atom which was shifted toward one side of the five membered *ortho*‐rings of the boron clusters, so that the silver atom became η^2^‐coordinated. Besides the elongation of all distances (Figure 6), the structure also shows a significant distortion as well as tilting of the cluster structure similar to the calculated η^2^ isomer of [Ag^+I^(*closo*‐B_11_H_11_)_2_]^3−^ depicted in Figure 7c). After this, the single crystal was warmed stepwise and measured again at different temperatures. While at 160 K the structure remained unchanged with an η^2^‐coordination mode, it shifted back to the η^5^‐coordinated structure upon warming up to a higher temperature. While the majority of the cell volumes determined follow a clear trend, two of them were found to be outliners. Namely, the one determined at 110 K during cooling and the one at 170 K when warming up. Structure solution suggests an intermediate structure in which the silver atom reveals either an η^5^ or an η^2^ coordination for both ligands, respectively. Noteworthy, a mixed structure with one ligand being η^5^ and the second η^2^ coordinated to silver, i.e. [Ag^+III^(η^2^‐B_11_H_11_)(η^5^‐(B_11_H_11_)]^3−^, was not observed.

The geometry of the ligand significantly changes during isomerization (Figure 6). In [Ag^+V^(*nido*‐B_11_H_11_)_2_]^3−^ the arrangement of the boron atoms is similar to that found in crystal structures of the non‐coordinated but protonated *nido*‐cluster [B_11_H_14_]^–^.^[^ ^66^ ^]^ For example, all B–B distances of the B atoms coordinated to silver in the crystal structure of (*n*‐Bu_4_N)_3_[Ag^+V^(*nido*‐B_11_H_11_)_2_] are very similar (186.6(3)–184.0(3) pm) as expected for a regular pentagon, *i.e*. local *C*_5v_ symmetry of the *nido*‐cluster. In contrast, the boron clusters in [Ag^+I^(*closo*‐B_11_H_11_)_2_]^3−^ are not close to *C*_5v_ symmetry but their geometry resembles parallels to the geometry of the *closo‐*[B_11_H_11_]^2–^ dianion, which ideally is of *C*_2v_ symmetry.^[^ ^67^, ^68^ ^]^ Hence, these structural changes resemble the transition from a (*nido*‐B_11_H_11_)^4–^ to a (*closo*‐B_11_H_11_)^2–^ ligand, which is again in line with a change from an Ag(+V) to an Ag(+I) complex.

During the conversion from η^5^ to η^2^, the color of the crystals becomes significantly lighter (Figure S45). The frequency of the ν(BH) vibration also decreases slightly (Figure S46). These observations are consistent with theoretical calculations (Figures S32–S38, S47).

This conversion of the bis‐η^5^ to the bis‐η^2^ isomer at lower temperatures is unusual because the bis‐η^2^ isomer is slightly larger and has a higher entropy. The calculated entropy change Δ*S*_298_ = (Δ*H*_298_−Δ*G*_298_)/T at 298 K for η^2^ to η^5^ transition is −111.7 J·K^−1^·mol^−1^ (vacuum) or −80.5 J·K^−1^·mol^−1^ (SCRF(Solvent = Water)) for B3LYP/aug‐cc‐pvtz(‐pp). The main contribution to the entropy change comes from low‐frequency vibrations, which are strongly hindered in the solid. For this reason, it is possible that the entropy change Δ*S*_298_ in (*n*‐Bu_4_N)_3_[Ag(B_11_H_11_)_2_] is positive. This would mean that for the transition bis‐η^2^ to bis‐η^5^ in (*n*‐Bu_4_N)_3_[Ag(B_11_H_11_)_2_] Δ*H*_298_ and Δ*S*_298_ are positive.

In the cases of (*n*‐Bu_4_N)_3_[Cu(B_11_H_11_)_2_] and (*n*‐Bu_4_N)_3_[Au(B_11_H_11_)_2_], such a transformation was not observed. This correlates with theoretical calculations showing that η^5^ coordination in the condensed state is more stable than η^2^ coordination for [Cu(B_11_H_11_)_2_]^3−^ and [Au(B_11_H_11_)_2_]^3−^ (Table 1). In the case of [Ag(B_11_H_11_)_2_]^3−^, the relative stabilities of complexes with η^5^ and η^2^ coordination are similar. Since the energy differences between the rotamers are not large (Table S14 and Figure S48), the geometry of the η^2^ isomer of [Ag(B_11_H_11_)_2_]^3−^ is adapted to the crystal lattice of (*n*‐Bu_4_N)_3_[Ag(B_11_H_11_)_2_]. Thus, the value of the dihedral angle between the boron atoms coordinated to silver (139.0(2)°) is closer to that of *C*_2_ *_v_* rotamer (180°) than to that of the most stable rotamer C_2_ (80°).

**Table 1:** Relative energy (Δ*G*_298_, kJ mol^−1^) of the isomer with oxidation number + I (d^10^ complex) compared to the isomer with oxidation number +V (d^6^ complex) for [M(B_11_X_11_)_2_]^3−^ (M = Cu, Ag, Au; X = H, F).

**Functional**	**Basis set**	**Cu, H**	**Ag, H**	**Au, H**	**Cu, F**	**Ag, F**	**Au, F**
B3LYP	B, H, F: aug‐cc‐pvtz M: aug‐cc‐pvtz‐pp	−69.8	−106.5	76.0	78.5	11.3	129.4
B3LYP, SCRF (Solvent=Water)	B, H, F: aug‐cc‐pvtz M: aug‐cc‐pvtz‐pp	25.5	−9.6	146.6	130.3	64.2	(191)^a)^

^a)^ Optimization of [Au^+I^(*closo*‐B_11_H_11_)_2_]^3−^ in this case resulted in [Au^+V^(*nido*‐B_11_H_11_)_2_]^3−^. The given value is for the C_2h_ rotamer, which has an imagined frequency.

### DFT‐Calculations

**Figure 8:**
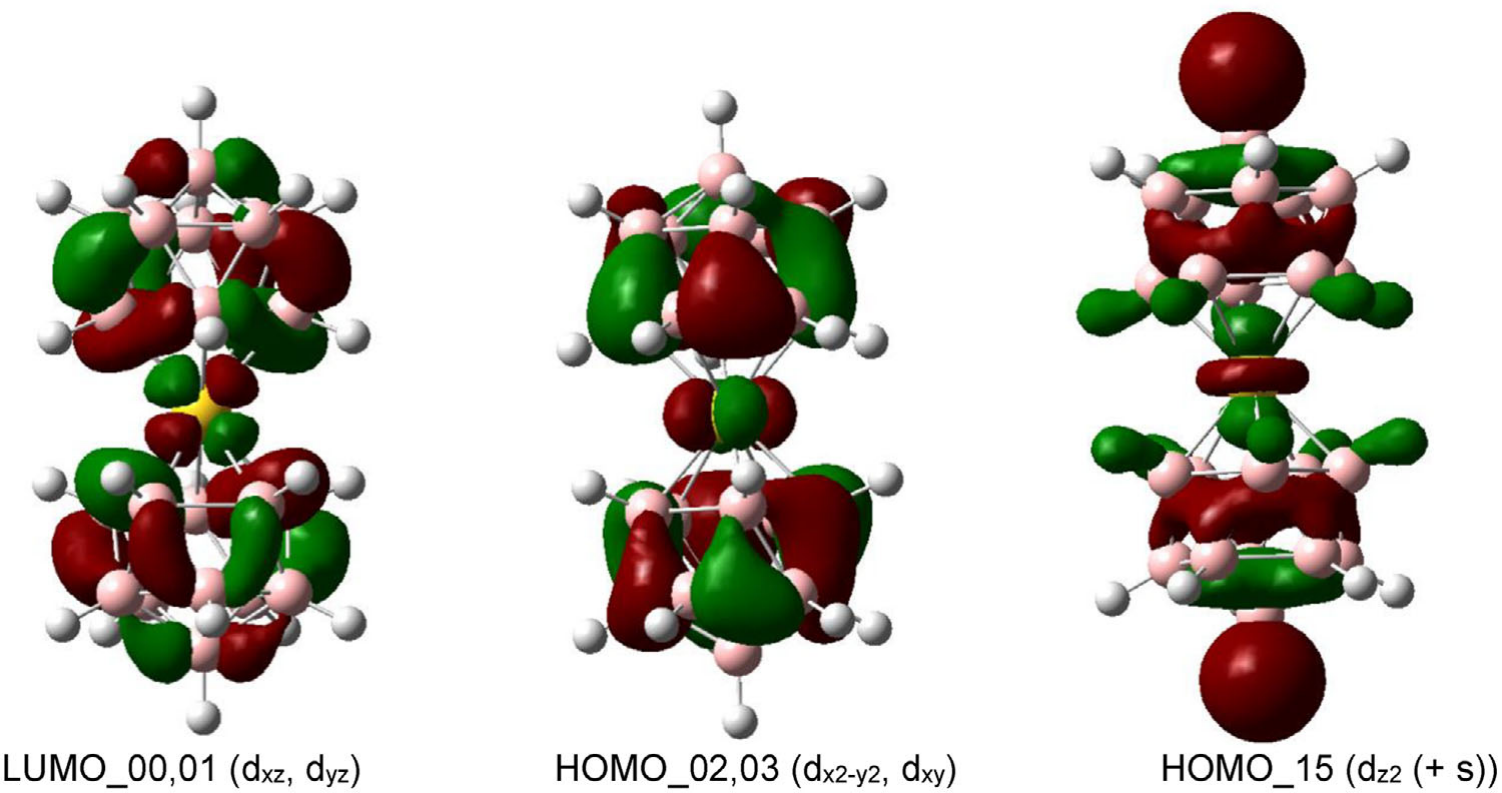
Selected MO for [Au^+V^(*nido*‐B_11_H_11_)_2_]^3−^ (SCRF(Solvent = Water) to visualize the chemical bonding situation, Functional: B3LYP, Basis set: B, H, F: 6‐311++g(d,p); Au: SDD Isovalue: 0.03). LUMO is the lowest unoccupied molecular orbital and HOMO is the highest occupied molecular orbital. The numbers show the order of the orbitals to the LUMO_00 and to the HOMO_00. The energy relative to HOMO_00 level is 4.28 eV for LUMO_00,01, −0.94 eV for HOMO_02,03, and −2.42 eV for HOMO_15.

The lower stability of [Ag(B_11_H_11_)_2_]^3−^ compared to [Cu(B_11_H_11_)_2_]^3−^ and [Au(B_11_H_11_)_2_]^3−^ is confirmed by DFT calculations. Since the d‐electrons of silver are energetically significantly lower than those of copper and gold, the relative stability of the isomer with the oxidation number +V (d^6^) to the isomer with the oxidation state +I (d^10^) for silver complex is lower compared to the corresponding copper and gold complexes (Tables 2 and S5). This becomes clear from the comparison of the relative energies of isomers with formal metal oxidation numbers +V ([M^+V^(*nido*‐B_11_H_11_)_2_]^3−^) and +I ([M^+I^(*closo*‐B_11_X_11_)_2_]^3−^) (Figure 7, Table 1). The two ligands in [M^+V^(*nido*‐B_11_H_11_)_2_]^3−^ can rotate against each other. The rotamers can have point groups *D*_5_, *D*_5_ *_d_*, and *D*_5_ *_h_*. The most stable rotamer has the point group *D*_5_ *_d_*. Selected molecular orbitals (MO) for [Au(B_11_H_11_)_2_]^3−^ are shown in Figure 8. The two ligands in [M^+I^(*closo*‐B_11_X_11_)_2_]^3−^ can also rotate against each other and the rotamers can adopt the point groups *C*_2_, *C*_2_ *_h_*, and *C*_2_ *_v_*. The most stable rotamer has the point group *C*_2_, but even the rotamers with the point group *C*_2_ *_h_* are only a few kJ mol^−1^ higher in energy. The rotamers of the [M^+III^(*nido*‐B_11_X_11_)(*closo*‐B_11_X_11_)]^3−^ isomer can adopt the point groups *C_s_* and *C*_1_. The geometry optimization of the [M^+III^(*nido*‐B_11_X_11_)(*closo*‐B_11_X_11_)]^3−^ isomers in point group *C*_1_ always resulted in either [M^+V^(*nido*‐B_11_H_11_)_2_]^3−^ or [M^+I^(*closo*‐B_11_X_11_)_2_]^3−^. The relative stability of isomers with oxidation numbers +V and +I correlates with the accessibility of the valence d‐electrons to the chemical bonds (Tables 2 and S5). The relative stabilities of [M^+V^(*nido*‐B_11_H_11_)_2_]^3−^ are calculated to be higher in solution (water) than in vacuum (Table 1).

**Table 2:** Orbital energies (eV) of the valence orbitals in Cu^+^, Ag^+^, and Au^+^ (B3LYP, Basis set: aug‐cc‐pvdz‐PP).

**Orbitals**	**Cu^+^, *n* = 4**	**Ag^+^, *n* = 5**	**Au^+^, *n* = 6**
Δ(*n*−1)d/ns	−4.547	−6.465	−4.420
(*n*−1)d	−15.636	−17.104	−16.672
ns	−11.089	−10.639	−12.252
np	−6.061	−5.919	−6.254

### Derivatization of [M(B_11_H_11_)_2_]^3−^ (M = Cu, Ag, Au)

Similar to the literature procedure,^[^ ^43^ ^]^ we used an excess of CuO in the synthesis of [Cu(B_11_H_11_)_2_]^3−^. Although salts with [Cu(B_11_H_11_)_2_]^3−^ are orange in color, the reaction mixtures are dark green and salts obtained directly from the reaction mixtures as precipitates are also green. Presumably, the green compounds are complexes of [Cu(B_11_H_11_)_2_]^3−^ with Cu(II). Attempts to obtain crystals suitable for X‐ray structure analysis failed so far. Furthermore, they slowly decompose to yield salts of [Cu(B_11_H_11−x_(OH)_x_)_2_]^3−^ among other unidentified products:

$$\begin{array}{cc} {\left[\text{Cu}{\left(B_{11}H_{11}\right)}_{2}\right]}^{3-}\cdot {\text{Cu}}^{2+} & \to {\left[\text{Cu}\left(B_{11}H_{11}\right)\left(B_{11}H_{10}\text{OH}\right)\right]}^{3-}\\ & \;+\text{Cu}↓+\cdots \end{array}$$

The green complexes react with sulfide ions to form [Cu(B_11_H_11_)_2_]^3−^ and CuS:

$${\left[\text{Cu}{\left(B_{11}H_{11}\right)}_{2}\right]}^{3-}\cdot {\text{Cu}}^{2+}+S^{2-}\to {\left[\text{Cu}{\left(B_{11}H_{11}\right)}_{2}\right]}^{3-}+\text{CuS}↓$$

**Figure 9:**
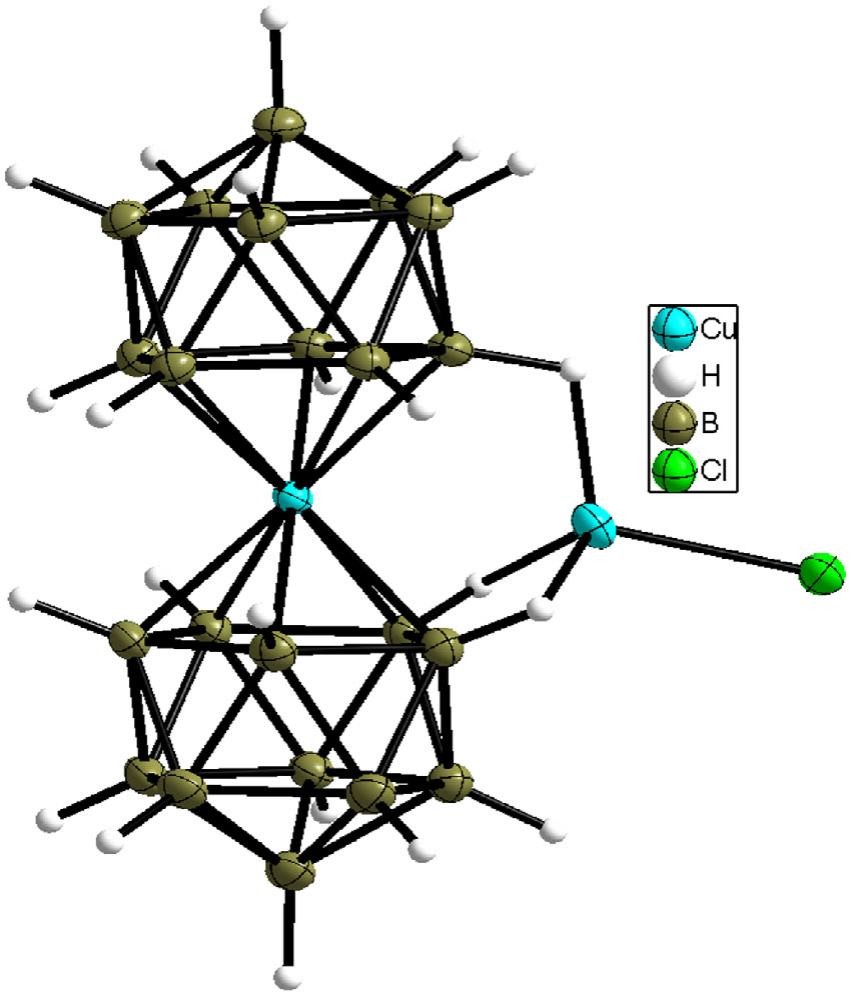
Structure of the anions in (Et_3_NH)_3_[Cu(B_11_H_11−x_ (OH)_x_)_2_]·0.95CuCl (*x* = 0.25) at 150 K. The OH groups and minor positions of copper atoms were not depicted. Thermal ellipsoids are drawn at 50% probability.

Orange (Et_3_NH)_3_[Cu(B_11_H_11_)_2_]·CuCl was isolated from these green compounds in the presence of Et_3_NH^+^ and Cl^−^. X‐ray crystallography confirms coordination of CuCl to [Cu(B_11_H_11_)_2_]^3−^ (Figures 9 and S4).

The hydrogen atoms in the *ortho* position to copper atoms in [Cu(B_11_H_11_)_2_]^3−^ carry a slightly larger negative charge and are therefore more reactive than the hydrogen atoms in the *meta* and *para* positions. This is because copper is less electronegative than boron.^[^ ^69^, ^70^ ^]^ Thus, the OH groups in [Cu(B_11_H_11−_ *_x_* (OH)*_x_*)_2_]^3−^ (*x* < 5) are almost always in the *ortho* position. However, the hydrogen atoms in *ortho* position in [Cu(B_11_H_11_)_2_]^3−^ are significantly less reactive than in [Co(B_11_H_11_)_2_]^4−[^ ^42^ ^]^ and are not hydroxylated at room temperature in neutral aqueous or acetonitrile solutions.

**Figure 10:**
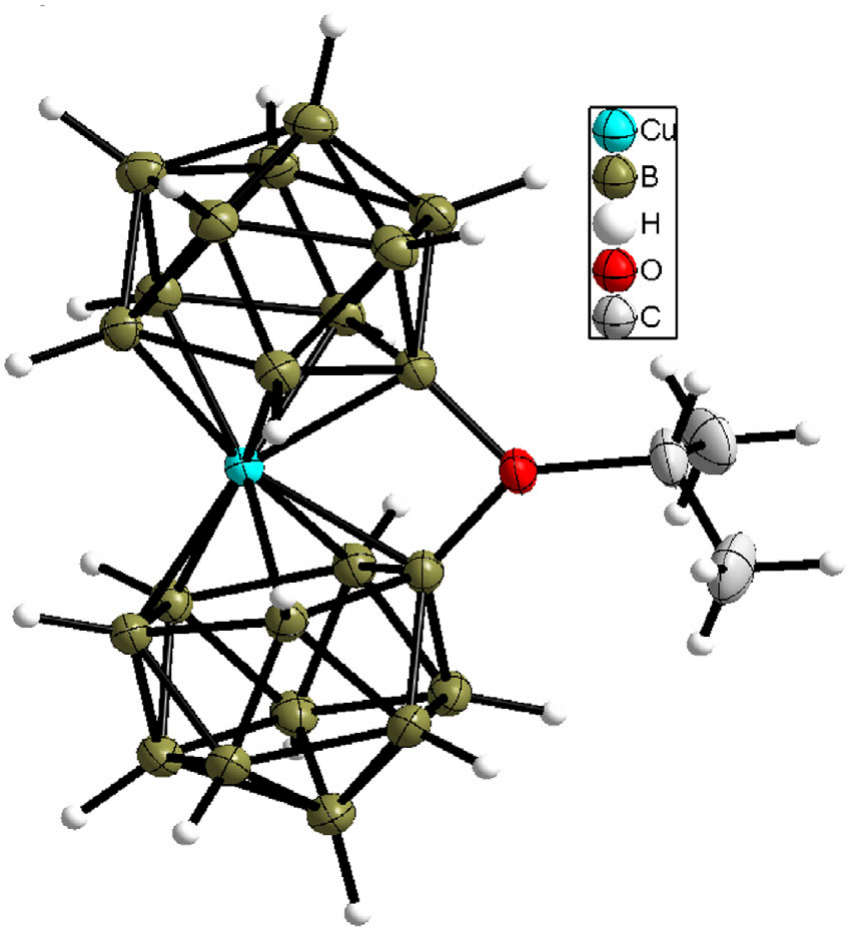
Structure of the anions in (Et_3_NH)_2_[Cu(B_11_H_10_)_2_(OCHMe_2_)]·H_2_O at 150 K. Thermal ellipsoids are drawn at 50% probability.

When [Cu(B_11_H_11_)_2_]^3−^ is heated to 50–60 °C in an acetone solution in the presence of Et_3_NH^+^, [Cu(B_11_H_10_)_2_(OCHMe_2_)]^2−^ is formed, which was isolated as (Et_3_NH)_2_[Cu(B_11_H_10_)_2_(OCHMe_2_)]·H_2_O (Figures 10 and S5).

Similar to [B_12_H_12_]^2−^,^[^ ^71^, ^72^, ^73^ ^]^ [Cu(B_11_H_11_)_2_]^3−^, and [Au(B_11_H_11_)_2_]^3−^ are only partially fluorinated by anhydrous HF at room temperature, and as in the case of [B_12_H_12_]^2−^, considerable amounts of insoluble polymeric boranes are formed.

The NMR (^11^B, ^1^H, ^19^F) spectra are not very informative in this case, as there are unresolved superimposed peaks from several complexes. Consequently, X‐ray crystallography is more conclusive. In the case of [Cu(B_11_H_11_)_2_]^3−^, after 3 days at room temperature, the average occupancy of fluorine atoms in *ortho* position is 0.77, and in *para* position 0.39 (Figure S1). In the case of [Au(B_11_H_11_)_2_]^3−^, after 1 day at room temperature and 2 days at 50–60 °C, the average occupancy degree of fluorine atoms in *ortho* position is 0.52, in *meta* position 0.26, and in *para* position 0.24 (Figure S3). The substitution pattern in the case of [Au(B_11_H_11_)_2_]^3−^ is slightly different from that in the case of [Cu(B_11_H_11_)_2_]^3−^ and is more similar to that in [B_12_H_8_F_4_]^2−^.^[^ ^71^, ^72^, ^73^ ^]^ From these data it can be concluded that the hydrogen atoms in [Au(B_11_H_11_)_2_]^3−^ are less hydridic and less reactive than in [Cu(B_11_H_11_)_2_]^3−^.

## Conclusion

During our study on the complex anions [M(B_11_H_11_)_2_]^3−^ (M = Cu, Ag, Au), we synthesized the gold representative [Au(B_11_H_11_)_2_]^3−^ and derivates of the copper complex, accompanied with the exploration of its chemical properties, e.g. leading to [Cu(B_11_H_10_)_2_(OCHMe_2_)]^2–^ as the first selectively functionalized cluster of this type of coinage metal complexes. The reaction of [M(B_11_H_11_)_2_]^3−^ with anhydrous HF led to the partial substitution of hydrogen by fluorine atoms to form [M(B_11_H_11−x_F_x_)_2_]^3−^ (M = Cu, Au; *x* ≈ 4).

Aside of the first synthesis of [Au(B_11_H_11_)_2_]^3−^, the key advancement of this work is the discovery that the anion [Ag(B_11_H_11_)_2_]^3−^ exists an equilibrium between the kinetically stable [Ag^+V^(nido‐B_11_H_11_)_2_]^3−^ (η^5^) and the kinetically labile [Ag^+I^(closo‐B_11_H_11_)_2_]^3−^ (η^2^) form by structural analysis using single‐crystal XRD. When a single crystal is gradually cooled to 100 K, a phase transition occurs at 110–130 K in which the coordination of the ligands on the silver changes from η^5^ to η^2^. To the best of our knowledge, this is the first observation of this type of low temperature isomerization for this class of compounds.

## Supporting Information

The authors have cited additional references within the Supporting Information.^[^ ^56^, ^57^, ^74^, ^75^, ^76^, ^77^, ^78^, ^79^, ^80^, ^81^, ^82^, ^83^, ^84^, ^85^, ^86^, ^87^, ^88^, ^89^, ^90^, ^91^, ^92^, ^93^, ^94^, ^95^, ^96^, ^97^, ^98^, ^99^, ^100^, ^101^, ^102^, ^103^, ^104^, ^105^, ^106^, ^107^, ^108^, ^109^, ^110^, ^111^, ^112^ ^]^ Synthesis of the following compounds is given in Supporting Information: Na_2_[B_11_H_11_]·3C_4_H_8_O_2_, BPy_4_I_3_, Na_3_[Cu(B_11_H_11_)_2_]·8H_2_O, (Et_3_NH)_2_[Cu(B_11_H_10_)_2_(OCHMe_2_)]·H_2_O, (Et_3_NH)_3_[Cu(B_11_H_11_)_2_]·CuCl, K_3_[Cu(B_11_H_11_)_2_]·5H_2_O, K_3_[Cu(B_11_H_11_)_2_], BPy_4_[Cu(B_11_H_11_)_2_], Cs_3_[Cu(B_11_H_11−x_F_x_)_2_], K_3_[Ag(B_11_H_11_)_2_]·5H_2_O, K_3_[Ag(B_11_H_11_)_2_], Na_3_[Au(B_11_H_11_)_2_]·8H_2_O, K_3_[Au(B_11_H_11_)_2_]·2H_2_O, K_3_[Au(B_11_H_11_)_2_], K_3_[Au(B_11_H_11−x_F_x_)_2_], (*n*‐Bu_4_N)_3_[M(B_11_H_11_)_2_] (M = Cu, Ag, Au). The crystal structure was determined for the following compounds (CCDC number in brackets): Na_3_[Cu(B_11_H_11_)_2_]·8H_2_O (2421085), (2449213), K_3_[Cu(B_11_H_11_)_2_]·5H_2_O (2421080), K_3_[Cu(B_11_H_11_)_2_]·2H_2_O (2421088), K_3_[Cu(B_11_H_11_)_2_]·3.5H_2_O·0.5KOH (2454502), (NH_4_)_3_[Cu(B_11_H_11_)_2_]·3NH_4_Cl (2421078), BPy_4_[Cu(B_11_H_11_)_2_] (2421077), BPy_4_[Cu(B_11_H_11_)_2_]·5Me_2_SO (2421089), (*n*‐Bu_4_N)_3_[Cu(η^5^‐B_11_H_11_)_2_] (2454491), Na_3_[Cu(B_11_H_11−x_(OH)_x_)_2_]·8H_2_O (x = 0.19, 2421086), K_3_[Cu(B_11_H_11−x_(OH)_x_)_2_]·5H_2_O (x = 0.14, 2421083), [Co(NH_3_)_6_]_3_[Cu(B_11_H_11−x_(OH)_x_)_2_]·2H_2_O (x = 0.15, 2421081), (Et_3_NH)_3_[Cu(B_11_H_11−x_ (OH)_x_)_2_]·0.88H_2_O (x = 0.11, 2421087), (Et_3_NH)_3_[Cu(B_11_H_11−x_(OH)_x_)_2_]·0.95CuCl (x = 0.25, 2421084), (Et_3_NH)_3_[Cu(B_11_H_10_)_2_ (OCHMe_2_)]·H_2_O (2421082), Cs_3_[Cu(B_11_H_11−x_F_x_)_2_] (x = 4.26, 2421079), K_3_[Ag(B_11_H_11_)_2_]·2H_2_O (2421091, 2454493, 2421090), K_3_[Ag(B_11_H_11_)_2_]·5H_2_O (2421092), (*n*‐Bu_4_N)_3_[Ag(η^2^‐B_11_H_11_)_2_] (2454494), (*n*‐Bu_4_N)_3_[Ag(η^5^‐B_11_H_11_)_2_] (2454490), (*n*‐Bu_4_N)_3_[Ag(η^5^‐B_11_H_11_)_2_]·0.5H_2_O·0.5Me_2_SO (2469093, 2469092), Na_3_[Au(B_11_H_11_)_2_]·8H_2_O (2421097, 2421093), K_3_[Au(B_11_H_11_)_2_]·2H_2_O (2421099, 2421096, 2421094), K_3_[Au(B_11_H_11_)_2_]·3CH_3_CN (2454492), BPy_4_[Au(B_11_H_11_)_2_] (2421098), (*n*‐Bu_4_N)_3_[Au(η^5^‐B_11_H_11_)_2_] (2455740), K_3_[Au(B_11_H_11−x_F_x_)_2_]·4H_2_O (x = 4.12, 2421095), Na_2_[B_11_H_11_]·5C_4_H_8_O_2_·2MeCN·2H_2_O (2421101), Na_2_[B_11_H_11_]·3C_4_H_8_O_2_ (2421100), K_2_[B_11_H_11_]·H_2_O (2421102), BPy_4_I_3_ (2432369), BPy_4_I_3_·H_2_O (2432365), BPy_4_I_3_·2H_2_O (2432363), BPy_4_(NO_3_)_3_ (2432366), BPy_4_(BF_4_)_3_ (2432364), BPy_4_(PF_6_)_3_ (2432368), BH_2_Py_2_I_3_ (2456440), *p*‐(C_5_H_5_N)C_5_H_4_NHI_2_·H_2_O (2432367). Crystallographic data for the structures in this paper (Table S1 and Figures S1–S5) have been deposited with the Cambridge Crystallographic Data Centre, CCDC, 12 Union Road, Cambridge CB21EZ, UK. Further data on NMR, HRMS, DSC, DTG, STA, Cyclic voltammetry, UV–vis, XANES, EXAFS, computational details (DFT) and Raman spectra and color of [*n‐*Bu_4_N]_3_[Ag(B_11_H_11_)_2_] at different temperatures are provided in Supporting Information. MO images and atomic coordinates of [M(B_11_H_11_)_2_]^3−^ (M = Cu, Ag, Au) are in Supporting Information in additional files.

## Conflict of Interests

The authors declare no conflict of interest.

## Supporting information



Supporting Information

Supporting Information

## Data Availability

The data that support the findings of this study are available in the Supporting Information of this article.

## References

[1] A. Stock , C. Massenez , Ber. Dtsch. Chem. Ges. 1912, 45, 3539–3568, 10.1002/cber.191204503113.[CrossRef]

[2] E. Wiberg , Ber. dtsch. Chem. Ges. A/B 1936, 69, 2816–2842, 10.1002/cber.19360691241.[CrossRef]

[3] A. Stock , Naturwissenschaften 1937, 25, 417–420, 10.1007/BF01492209.[CrossRef]

[4] H. I. Schlesinger , H. C. Brown , B. Abraham , A. C. Bond , N. Davidson , A. E. Finholt , J. R. Gilbreath , H. Hoekstra , L. Horvitz , E. K. Hyde , J. J. Katz , J. Knight , R. A. Lad , D. L. Mayfield , L. Rapp , D. M. Ritter , A. M. Schwartz , I. Sheft , L. D. Tuck , A. O. Walker , J. Am. Chem. Soc. 1953, 75, 186–190, 10.1021/ja01097a049.[CrossRef]

[5] H. I. Schlesinger , H. C. Brown , A. E. Finholt , J. Am. Chem. Soc. 1953, 75, 205–209, 10.1021/ja01097a054.[CrossRef]

[6] V. A. Brattsev , Pecherskij , Maksim , Viktorovich , E. L. Gurkova , Storozhenko Pavel Arkadevich, RU2344070C1, 2007.

[7] T. Luo , W. Huang , F. Chu , T. Zhu , B. Feng , S. Huang , J. Hou , L. Zhu , S. Zhu , W. Zeng , Mol. Pharmaceutics 2023, 20, 4942–4970, 10.1021/acs.molpharmaceut.3c00701.[CrossRef] 37728998

[8] J. Plesek , Chem. Rev. 1992, 92, 269–278, 10.1021/cr00010a005.[CrossRef]

[9] A. H. Soloway , W. Tjarks , B. A. Barnum , F.‐G. Rong , R. F. Barth , I. M. Codogni , J. G. Wilson , Chem. Rev. 1998, 98, 1515–1562, 10.1021/cr941195u.[CrossRef] 11848966

[10] K. Fink , M. Uchman , Coord. Chem. Rev. 2021, 431, 213684, 10.1016/j.ccr.2020.213684.[CrossRef]

[11] A. Barba‐Bon , G. Salluce , I. Lostalé‐Seijo , K. I. Assaf , A. Hennig , J. Montenegro , W. M. Nau , Nature 2022, 603, 637–642, 10.1038/s41586-022-04413-w.[CrossRef] 35322251 PMC8942850

[12] Y. Wang , A. M. Spokoyny , ACS Cent. Sci. 2022, 8, 309–311, 10.1021/acscentsci.2c00187.[CrossRef] 35350607 PMC8949626

[13] Y. Endo , T. Yoshimi , C. Miyaura , Pure Appl. Chem. 2003, 75, 1197–1205, 10.1351/pac200375091197.

[14] J. C. Axtell , L. M. A. Saleh , E. A. Qian , A. I. Wixtrom , A. M. Spokoyny , Inorg. Chem. 2018, 57, 2333–2350, 10.1021/acs.inorgchem.7b02912. 29465227 PMC5985200

[15] Z. Huang , S. Wang , R. D. Dewhurst , N. V. Ignat'ev , M. Finze , H. Braunschweig , Angew. Chem. Int. Ed. 2020, 59, 8800–8816, 10.1002/anie.201911108. PMC7317435 31625661

[16] S. Payandeh , D. Rentsch , Z. Łodziana , R. Asakura , L. Bigler , R. Černý , C. Battaglia , A. Remhof , Adv. Funct. Mater. 2021, 31, 2010046.

[17] E. Didelot , Z. Łodziana , F. Murgia , R. Černý , Crystals 2019, 9, 372.

[18] S. Payandeh , R. Asakura , P. Avramidou , D. Rentsch , Z. Łodziana , R. Černý , A. Remhof , C. Battaglia , Chem. Mater. 2020, 32, 1101–1110, 10.1021/acs.chemmater.9b03933.

[19] Y. Sadikin , E. Didelot , Z. Łodziana , R. Černý , Dalton Trans. 2018, 47, 5843–5849, 10.1039/C8DT00381E. 29648562

[20] E. Didelot , Y. Sadikin , Z. Łodziana , R. Černý , Solid State Sci. 2019, 90, 86–94, 10.1016/j.solidstatesciences.2019.02.005.

[21] C. W. Jack, Jr , I. S. Vladimirovich , P. G. Peter , U. Michael , EP1513215 A2, 2004.

[22] J. Arai , A. Matsuo , T. Fujisaki , K. Ozawa , J. Power Sources 2009, 193, 851–854, 10.1016/j.jpowsour.2009.04.001.

[23] Z. Chen , J. Liu , A. N. Jansen , G. GirishKumar , B. Casteel , K. Amine , Electrochem. Solid‐State Lett. 2010, 13, A39, 10.1149/1.3299251.

[24] Z. Chen , Y. Ren , A. N. Jansen , C.‐K. Lin , W. Weng , K. Amine , Nat. Commun. 2013, 4, 1513, 10.1038/ncomms2518. 23443541

[25] S. V. Ivanov , S. M. Miller , O. P. Anderson , K. A. Solntsev , S. H. Strauss , J. Am. Chem. Soc. 2003, 125, 4694–4695, 10.1021/ja0296374. 12696872

[26] L. Duchêne , R.‐S. Kühnel , D. Rentsch , A. Remhof , H. Hagemann , C. Battaglia , Chem. Commun. (Cambridge, U. K.) 2017, 53, 4195. 10.1039/c7cc00794a 28345102

[27] R. Asakura , L. Duchêne , S. Payandeh , D. Rentsch , H. Hagemann , C. Battaglia , A. Remhof , ACS Appl. Mater. Interfaces 2021, 13, 55319–55328, 10.1021/acsami.1c15246. 34757707

[28] H. C. Longuet‐Higgins , Q. Rev., Chem. Soc. 1957, 11, 121, 10.1039/qr9571100121.

[29] W. H. Eberhardt , B. Crawford , W. N. Lipscomb , J. Chem. Phys. 1954, 22, 989–1001, 10.1063/1.1740320.

[30] W. N. Lipscomb , Boron Hydrides, W. A. Benjamin Inc, New York‐Amsterdam 1963.

[31] W. N. Lipscomb , Science (New York, N.Y.) 1977, 196, 1047–1055, 10.1126/science.196.4294.1047. 17778522

[32] K. Wade , J. Chem. Soc. D 1971, 792–793. 10.1039/c29710000792.

[33] E. D. Jemmis , P. D. Pancharatna , Appl. Organomet. Chem. 2003, 17, 480–492, 10.1002/aoc.462.

[34] F. Schlüter , E. Bernhardt , Inorg. Chem. 2011, 50, 2580–2589, 10.1021/ic102434t. 21341737

[35] J. Bicerano , D. S. Marynick , W. N. Lipscomb , Inorg. Chem. 1978, 17, 3443–3453, 10.1021/ic50190a028.

[36] O. Volkov , P. Paetzold , J. Organomet. Chem. 2003, 680, 301–311, 10.1016/S0022-328X(03)00460-1.

[37] I. B. Sivaev , Russ. J. Inorg. Chem. 2019, 64, 955–976, 10.1134/S003602361908014X.

[38] E. W. Abel , I. R. Butler , in Organometallic Chemistry (Eds.: J. L. Wardell , C. E. Housecroft , K. C. Molloy , D. A. Armitage , J. A. Timney , G. Hogarth , M. J. Winter , M. J. Morris , D. G. Evans , I. R. Butler , Eds. et al.), The Royal Society of Chemistry, UK 1994, pp. 376–440, 10.1039/9781847554123.

[39] V. D. Aftandilian , H. C. Miller , G. W. Parshall , E. L. Muetterties , Inorg. Chem. 1962, 1, 734–737, 10.1021/ic50004a003.

[40] G. B. Dunks , K. P. Ordonez , Inorg. Chem. 1978, 17, 1514–1516, 10.1021/ic50184a025.

[41] G. B. Dunks , K. Barker , E. Hedaya , C. Hefner , K. Palmer‐Ordonez , P. Remec , Inorg. Chem. 1981, 20, 1692–1697, 10.1021/ic50220a015.

[42] E. Bernhardt , A. Drichel , M. Krnel , E. Svanidze , A. Slabon , Inorg. Chem. 2024, 63, 5414–5422, 10.1021/acs.inorgchem.3c04032. 38478580

[43] J. G. Kester , D. Keller , J. C. Huffman , M. A. Benefiel , W. E. Geiger , C. Atwood , A. R. Siedle , G. A. Korba , L. J. Todd , Inorg. Chem. 1994, 33, 5438–5442, 10.1021/ic00102a015.

[44] V. V. Avdeeva , A. S. Kubasov , A. V. Golubev , S. A. Anufriev , I. B. Sivaev , S. E. Nikiforova , L. V. Goeva , E. A. Malinina , N. T. Kuznetsov , Inorg. Chim. Acta 2023, 556, 121675, 10.1016/j.ica.2023.121675.

[45] D. Mingos , M. I. Forsyth , J. Organomet. Chem. 1978, 146, C37–C42, 10.1016/S0022-328X(00)88766-5.

[46] W. Klemm , E. Huss , Z. Anorg. Chem. 1949, 258, 221–226, 10.1002/zaac.19492580312.

[47] J. M. Whalen , G. M. Lucier , L. Chacón , N. Bartlett , J. Fluorine Chem. 1998, 88, 107–110, 10.1016/S0022-1139(98)00105-5.

[48] G. M. Lucier , C. Shen , S. H. Elder , N. Bartlett , Inorg. Chem. 1998, 37, 3829–3834, 10.1021/ic971603n. 11670486

[49] W. Harnischmacher , R. Hoppe , Angew. Chem. 1973, 85, 590.

[50] W. Harnischmacher , R. Hoppe , Angew. Chem. Int. Ed. Engl. 1973, 12, 582–583, 10.1002/anie.197305822.

[51] D. Kissel , R. Hoppe , Z. Anorg. Allg. Chem. 1988, 559, 40–48, 10.1002/zaac.19885590104.

[52] P. Sorbe , J. Fluorine Chem. 1978, 11, 243–250, 10.1016/S0022-1139(00)82444-6.

[53] K. Leary , N. Bartlett , J. Chem. Soc. Chem. Commun. 1972, 903–904, 10.1039/c39720000903.

[54] A. J. Edwards , W. E. Falconer , J. E. Griffiths , W. A. Sunder , M. J. Vasile , J. Chem. Soc. Dalton Trans. 1974, 1129–1133, 10.1039/dt9740001129.

[55] O. Graudejus , S. H. Elder , G. M. Lucier , C. Shen , N. Bartlett , Inorg. Chem. 1999, 38, 2503–2509, 10.1021/ic981397z. 11671173

[56] A. F. Holleman , E. Wiberg , Lehrbuch der anorganischen Chemie, De Gruyter, Berlin, Boston 1976, 10.1515/9783111509600.

[57] N. Wiberg , Inorganic Chemistry, De Gruyter Inc, Berlin/Boston 2008.

[58] V. C. Barra , E. Bernhardt , S. Fellinger , C. Jenne , S. S. Langenbach , Inorganics 2024, 12, 173, 10.3390/inorganics12060173.

[59] M. Baya , D. Joven‐Sancho , P. J. Alonso , J. Orduna , B. Menjón , Angew. Chem. Int. Ed. 2019, 58, 9954–9958, 10.1002/anie.201903496. 31095844

[60] M. Baya , D. Joven‐Sancho , P. J. Alonso , J. Orduna , B. Menjón , Angew. Chem. 2019, 131, 10059–10063, 10.1002/ange.201903496. 31095844

[61] J. Janek , W. G. Zeier , Nat. Energy 2023, 8, 230.

[62] M. R. Rosenthal , J. Chem. Educ. 1973, 50, 331, 10.1021/ed050p331.

[63] S. H. Strauss , Chem. Rev. 1993, 93, 927–942, 10.1021/cr00019a005.

[64] I. Krossing , I. Raabe , Angew. Chem. 2004, 116, 2116–2142, 10.1002/ange.200300620.

[65] R. N. Grimes , Carboranes, Academic Press an imprint of Elsevier, Amsterdam, Boston, Heidelberg 2016.

[66] O. Volkov , K. Radacki , R. L. Thomas , N. P. Rath , L. Barton , J. Organomet. Chem. 2005, 690, 2736–2744, 10.1016/j.jorganchem.2005.02.029.

[67] S. Z. Konieczka , F. Schlüter , C. Sindorf , C. Kerpen , E. Bernhardt , M. Finze , Chem.‐Eur. J. 2018, 24, 3528–3538, 10.1002/chem.201704860. 29251802

[68] O. Volkov , W. Dirk , U. Englert , P. Paetzold , Z. Anorg. Allg. Chem. 1999, 625, 1193–1200, 10.1002/(SICI)1521-3749(199907)625:7<1193::AID-ZAAC1193>3.0.CO;2-L.

[69] C. Tantardini , A. R. Oganov , Nat. Commun. 2021, 12, 2087, 10.1038/s41467-021-22429-0. 33828104 PMC8027013

[70] A. L. Allred , E. G. Rochow , J. Inorg. Nucl. Chem. 1958, 5, 264–268, 10.1016/0022-1902(58)80003-2.

[71] K. A. Solntsev , A. M. Mebel , N. A. Votinova , N. T. Kuznetsov , O. P. Charkin , Koord. Khim 1992, 18, 340.

[72] K. A. Solntsev , S. V. Ivanov , S. G. Sakharov , S. B. Katser , A. S. Chernyavskii , N. A. Votinova , E. A. Klyuchishche , N. T. Kuznetsov , Russ. J. Coord. Chem. 1997, 23, 369–376.

[73] K. A. Solntsev , S. V. Ivanov , S. G. Sakharov , S. B. Katser , A. S. Chernyavskii , N. A. Votinova , E. A. Klyuchishche , N. T. Kuznetsov , Koord. Khim. 1997, 23, 403–409.

[74] E. Bernhardt , H. Willner , DE102008004530A1, 2008.

[75] G. E. Ryschkewitsch , J. Am. Chem. Soc. 1967, 89, 3145–3148, 10.1021/ja00989a013.

[76] E. Bernhardt , C. Bach , B. Bley , R. Wartchow , U. Westphal , I. H. T. Sham , B. von Ahsen , C. Wang , H. Willner , R. C. Thompson , F. Aubke , Inorg. Chem. 2005, 44, 4189–4205, 10.1021/ic040115u. 15934748

[77] M. Finze , E. Bernhardt , H. Willner , C. W. Lehmann , F. Aubke , Inorg. Chem. 2005, 44, 4206–4214, 10.1021/ic0482483. 15934749

[78] W. C. Schumb , E. L. Gamble , M. D. Banus , J. Am. Chem. Soc. 1949, 71, 3225–3229, 10.1021/ja01177a084.

[79] H. C. Andersen , L. H. Belz , J. Am. Chem. Soc. 1953, 75, 4828, 10.1021/ja01115a501.

[80] R. D. Bohl , G. L. Galloway , J. Inorg. Nucl. Chem. 1971, 33, 885–887, 10.1016/0022-1902(71)80494-3.

[81] E. Haack , DE598879A.

[82] E. Haack , DE600499A.

[83] E. Koenigs , H. Greiner , Ber. dtsch. Chem. Ges. A/B 1931, 64, 1049–1056, 10.1002/cber.19310640518.

[84] E. Haack , DE613402A.

[85] *CrysAlisPro 1.171.42.88a*, Rigaku OD, 2023.

[86] G. M. Sheldrick , Acta Crystallogr. Sect. A:Found. Crystallogr. 2008, 64, 112–122, 10.1107/S0108767307043930. 18156677

[87] L. J. Farrugia , J. Appl. Crystallogr. 2012, 45, 849–854, 10.1107/S0021889812029111.

[88] G. M. Sheldrick , Acta Crystallogr. Sect. C:Struct. Chem. 2015, 71, 3–8, 10.1107/S2053229614024218. 25567568 PMC4294323

[89] G. M. Sheldrick , SHELXL‐2019/1, Bruker AXS Inc., Madison, Wisconsin, USA, Madison, Wisconsin, USA 2019.

[90] K. Brandenburg , *Diamond, v.3.2f*, Crystal Impact GbR, 2001.

[91] R. K. Harris , E. D. Becker , S. M. Cabral de Menezes , R. Goodfellow , P. Granger , Pure Appl. Chem. 2001, 73, 1795–1818, 10.1351/pac200173111795.

[92] G. R. Fulmer , A. J. M. Miller , N. H. Sherden , H. E. Gottlieb , A. Nudelman , B. M. Stoltz , J. E. Bercaw , K. I. Goldberg , Organometallics 2010, 29, 2176–2179, 10.1021/om100106e.

[93] E. A. Bernhardt , P. N. Komozin , Russ. J. Inorg. Chem. 1997, 42, 540–557.

[94] E. A. Bernhardt , P. N. Komozin , Zurn. Neorg. Khim 1997, 42, 614–631.

[95] W. Kohn , L. J. Sham , Phys. Rev. 1965, 140, A1133–A1138, 10.1103/PhysRev.140.A1133.

[96] A. D. Becke , Phys. Rev. A 1988, 38, 3098–3100, 10.1103/PhysRevA.38.3098. 9900728

[97] A. D. Becke , J. Chem. Phys. 1993, 98, 5648–5652, 10.1063/1.464913.

[98] C. Lee , W. Yang , R. G. Parr , Phys. Rev. B 1988, 37, 785–789, 10.1103/PhysRevB.37.785. 9944570

[99] R. Krishnan , J. S. Binkley , R. Seeger , J. A. Pople , J. Chem. Phys. 1980, 72, 650–654, 10.1063/1.438955.

[100] T. Clark , J. Chandrasekhar , G. W. Spitznagel , P. V. R. Schleyer , J. Comput. Chem. 1983, 4, 294.

[101] M. Dolg , U. Wedig , H. Stoll , H. Preuss , J. Chem. Phys. 1987, 86, 866–872, 10.1063/1.452288.

[102] D. Andrae , U. Huermann , M. Dolg , H. Stoll , H. Preu , Theoret. Chim. Acta 1990, 77, 123–141, 10.1007/BF01114537.

[103] P. Schwerdtfeger , M. Dolg , W. H. E. Schwarz , G. A. Bowmaker , P. D. W. Boyd , J. Chem. Phys. 1989, 91, 1762–1774, 10.1063/1.457082.

[104] T. H. Dunning , J. Chem. Phys. 1989, 90, 1007–1023, 10.1063/1.456153.

[105] R. A. Kendall , T. H. Dunning , R. J. Harrison , J. Chem. Phys. 1992, 96, 6796–6806, 10.1063/1.462569.

[106] K. A. Peterson , C. Puzzarini , Theoret. Chim. Acta 2005, 114, 283–296, 10.1007/s00214-005-0681-9.

[107] L. S. Kau , D. J. Spira‐Solomon , J. E. Penner‐Hahn , K. O. Hodgson , E. I. Solomon , J. Am. Chem. Soc. 1987, 109, 6433–6442, 10.1021/ja00255a032.

[108] A. A. Guda , S. A. Guda , A. Martini , A. N. Kravtsova , A. Algasov , A. Bugaev , S. P. Kubrin , L. V. Guda , P. Šot , J. A. van Bokhoven , C. Copéret , A. V. Soldatov , npj Comput. Mater. 2021, 7, 203.

[109] G. N. George , I. J. Pickering , X‐Ray Absorption Spectroscopy, De Gruyter, Berlin, Boston 2024, 10.1515/9783110570441.

[110] M. J. Frisch , G. W. Trucks , H. B. Schlegel , G. E. Scuseria , M. A. Robb , J. R. Cheeseman , G. Scalmani , V. Barone , G. A. Petersson , H. Nakatsuji , X. Li , M. Caricato , A. V. Marenich , J. Bloino , B. G. Janesko , R. Gomperts , B. Mennucci , H. P. Hratchian , J. V. Ortiz , A. F. Izmaylov , J. L. Sonnenberg , D. Williams‐Young , F. Ding , F. Lipparini , F. Egidi , J. Goings , B. Peng , A. Petrone , T. Henderson , D. Ranasinghe , et al., Gaussian 16, Revision A.03, Gaussian, Inc., Wallingford CT, 2016.

[111] M. J. Frisch , G. W. Trucks , H. B. Schlegel , G. E. Scuseria , M. A. Robb , J. R. Cheeseman , J. A. Montgomery, Jr. , T. Vreven , K. N. Kudin , J. C. Burant , J. M. Millam , S. S. Iyengar , J. Tomasi , V. Barone , B. Mennucci , M. Cossi , G. Scalmani , N. Rega , G. A. Petersson , H. Nakatsuji , M. Hada , M. Ehara , K. Toyota , R. Fukuda , J. Hasegawa , M. Ishida , T. Nakajima , Y. Honda , O. Kitao , H. Nakai, et al., Gaussian 03, Revision D.01, Gaussian, Inc., Wallingford CT, 2004.

[112] R. Dennington , T. Keith , J. Millam , GaussView Version 4.1.2, Semichem, Inc., Shawnee Mission, KS, 2007.

