# Supplementary material for: The Biicosahedral Complex Anions [M(B11H11)2]3− (M = Cu, Ag, Au): Synthesis and Unexpected Low‐Temperature Phase Transition of [Ag(η5‐B11H11)2]3− to [Ag(η2‐B11H11)2]3−
Source: Angew Chem Int Ed Engl. 2025 Nov 10;65(2):e19283. doi: 10.1002/anie.202519283 (PMC12790380; doi:10.1002/anie.202519283)
Supplement: Supplementary file 1 — Supporting Information [file ANIE-65-e19283-s002.pdf]

Supporting Information

The Biicosahedral Complex Anions $[M(B_{11}H_{11})_2]^{3-}$ (M = Cu, Ag, Au): Synthesis and Unexpected Low-Temperature Phase Transition of $[Ag(\eta^5\text{-}B_{11}H_{11})_2]^{3-}$ to $[Ag(\eta^2\text{-}B_{11}H_{11})_2]^{3-}$

Eduard Bernhardt*[a], Tanja Knuplez[b], Tobias Preitschopf[b], Andreas Drichel[a], Björn Beele[a], Alexey Maximenko[c], Maik Finze[b]*, Adam Slabon[a]*

[a] Chair of Inorganic Chemistry, University of Wuppertal, Gaußstr. 20, D-42119 Wuppertal, Germany
[b] Institut für Anorganische Chemie, Institut für nachhaltige Chemie & Katalyse mit Bor (ICB), Julius-Maximilians-Universität Würzburg, Am Hubland, 97074, Würzburg, Germany
[c] National Synchrotron Radiation Centre SOLARIS Jagiellonian University: Kraków, Małopolska, Poland

Number of Pages: 70
Number of Figures: 52

Table of contents

Tables / Figures	Page
**Synthetic procedures.**	S.3-S.10
**Table S1.** Summarized crystallographic data.	S.11-S.20
**Figure S1.** Structure of the cell and anions in $Cs_3[Cu(B_{11}H_{11-x}F_x)_2]$	S.21
**Figure S2.** Structure of the cell and anions in $K_3[Au(B_{11}H_{11})_2]\cdot 2H_2O$	S.22
**Figure S3.** Structure of the cell and anions in $K_3[Au(B_{11}H_{11-x}F_x)_2]\cdot 4H_2O$	S.22
**Figure S4.** Structure of the cell and anions in $(Et_3NH)_3[Cu(B_{11}H_{11-x}(OH)_x)_2]\cdot 0.95CuCl$	S.23
**Figure S5.** Structure of the cell and anions in $(Et_3NH)_2[Cu(B_{11}H_{10})_2(OCHMe_2)]\cdot H_2O$	S.23
NMR spectroscopy, **Tables S2a, S2, S3**	S.24-S.25
NMR spectroscopy of $[Cu(B_{11}H_{10})_2(OCHMe_2)]^{2-}$, **Figure S6, Table S4**	S.26-S.27
Atomic coordinates for $[Cu(B_{11}H_{10})_2(OCHMe_2)]^{2-}$	S.28-S.29
Some additional NMR and HRMS data.	S.30-S.31
DSC, DTG and STA. **Figures S7-S15**	S.32-S.37
Cyclic voltammetry	S.38
UV-Vis spectroscopy, **Tables S4a and S4b**	S.38-S39
**Figure S16a.** MO diagram of $[M(B_{11}H_{11})_2]^{3-}$ (M = Cu, Ag, Au).	S.39
**Figures S16-S26.** UV-Vis spectra of $K_3[M(B_{11}H_{11})_2]$ (M = Cu, Ag, Au)	S.40-S.45
**Figure S27.** UV-Vis spectra of $K_2[B_{11}H_{11}]\cdot H_2O$ in a water solution.	S.45
Computational details	S.46
**Table S5.** Orbital energies (eV) of the valence orbitals in Cu, Ag, and Au	S.46
**Table S5a.** Relative energy (ΔG_{298}, kJ/mol) of the isomer with oxidation number +I (d^{10} complex) compared to the isomer with oxidation number +V (d^6 complex) for $[M(B_{11}X_{11})_2]^{3-}$ (M = Cu, Ag, Au; X = H, F).	S.47
**Figures S28-S42.** Theoretical UV-Vis spectra of $K_3[M(B_{11}H_{11})_2]$ (M = Cu, Ag, Au)	S.48-S.55
**Figure S43.** Theoretical UV-Vis spectrum of *closo*-$[B_{12}H_{12}]^{2-}$	S.56
**Figure S44.** Theoretical UV-Vis spectrum of *closo*-$[B_{11}H_{11}]^{2-}$	S.56
**Tables S6-S11.** Calculated transitions in $K_3[M(B_{11}H_{11})_2]$ (M = Cu, Ag, Au)	S.57-S.59
**Table S12.** Orbital energies of the d-electrons in $[M(B_{11}H_{11})_2]^{3-}$ (M = Cu, Ag, Au).	S.59
**Table S13.** Some thermal ellipsoids in $K_3[M(B_{11}H_{11})_2]\cdot 2H_2O$ (M = Cu, Ag und Au) at 100 K.	S.60
**Figure S45.** Color of $[n\text{-}Bu_4N]_3[Ag(B_{11}H_{11})_2]$ at different temperatures	S.61
**Figure S46.** Raman spectra of $[n\text{-}Bu_4N]_3[Ag(B_{11}H_{11})_2]$ at different temperatures.	S.61
**Figure S47.** Calculated IR spectra of $[Ag(\eta^5\text{-}B_{11}H_{11})_2]^{3-}$ and $[Ag(\eta^2\text{-}B_{11}H_{11})_2]^{3-}$.	S.62
**Table S14.** Relative energy (ΔE, kJ/mol), dihedral angle τ (4-5-15-16, °) and average angle α_{av} (3-4-1 and 6-5-1, °) of the rotamers of the isomer with oxidation number +I (d^{10} complex) for $[M(B_{11}H_{11})_2]^{3-}$ (M = Cu, Ag, Au).	S.63
**Figure S48.** The rotamers of the isomer with oxidation number +I (d^{10} complex) for $[M(B_{11}H_{11})_2]^{3-}$ (M = Cu, Ag, Au).	S.63
X-ray absorption spectroscopy	S.64-68
XANES data of $K_3[Cu(B_{11}H_{11})_2]\cdot 5H_2O$	S.64-S.65
EXAFS data of $K_3[Cu(B_{11}H_{11})_2]\cdot 5H_2O$	S.66-S.68
References	S.69-S70

Synthetic procedures.

Synthesis of the following compounds is given below: $Na_2[B_{11}H_{11}]\cdot 3C_4H_8O_2$, BPy_4I_3, $Na_3[Cu(B_{11}H_{11})_2]\cdot 8H_2O$, $(Et_3NH)_2[Cu(B_{11}H_{10})_2(OCHMe_2)]\cdot H_2O$, $(Et_3NH)_3[Cu(B_{11}H_{11})_2]\cdot CuCl$, $K_3[Cu(B_{11}H_{11})_2]\cdot 5H_2O$, $K_3[Cu(B_{11}H_{11})_2]$, $BPy_4[Cu(B_{11}H_{11})_2]$, $Cs_3[Cu(B_{11}H_{11-x}F_x)_2]$, $K_3[Ag(B_{11}H_{11})_2]\cdot 5H_2O$, $K_3[Ag(B_{11}H_{11})_2]$, $Na_3[Au(B_{11}H_{11})_2]\cdot 8H_2O$, $K_3[Au(B_{11}H_{11})_2]\cdot 2H_2O$, $K_3[Au(B_{11}H_{11})_2]$, $K_3[Au(B_{11}H_{11-x}F_x)_2]$, $(n\text{-}Bu_4N)_3[M(B_{11}H_{11})_2]$ (M = Cu, Ag, Au).

Cu_2O (EGA-Chemie), CuO (Strem Chemicals, 99%), Ag_2O (Merck 99%), Au (Merck 99.99%; Degussa 99.99%), $Na[AuCl_4]\cdot 2H_2O$ (Merck 99.99%), I_2 (Acros Organics, 99%),, $Na[BH_4]$ (Fluka, 96%; Alfa Aesar, 97%), KOH (Fisher Chemicals, 86%), NaOH (Crüssing GhbH, 99%), K_2CO_3 (Merck KGaA, 99%), Cs_2CO_3 (Merck 99%), Na_2S (VWR International, 58–64%), PbO_2 (Riedel-de Haën, 99.5%), anhydrous HF (Solvay, AG Hannover, Germany), hydrochloric acid (HCl, Thermo Scientific, 37%), nitric acid (HNO_3, WVR Chemicals, 68%), tetrafluoroboric acid ($H[BF_4]$, Acros organics, 50%), hexafluorophosphoric acid ($H[PF_6]$, Aldrich, 60%), Me_3NHCl (Merck Schuchardt OHG, 98%), Et_3NHCl (Sigma Chemicals), n-Bu_4NBr (Fluka AG, 98%), n-Bu_4NHSO_4 (Alfa Aesar 97%), ascorbic acid (Alfa Aesar, 99%), cellulose for column chromatography (Carl Roth GmbH), tetrahydrothiophene (C_4H_8S, Sigma-Aldrich, 99%), acetone (Me_2CO, Fisher Chemicals, 99,8%), methanol (MeOH, Fisher Chemical, 99.8%), ethanol (EtOH, Th. Gegen GmbH & Co. KG, 99.9%), acetonitrile (MeCN, Fisher chemicals, 99.9%), dimethylformamide (DMF, Me_2NCHO, Riedel de Haën, 99.5%), diethyl ether (Et_2O, VWR International, 99.5%), dichloromethane (CH_2Cl_2, VWR International, 100%), dimethyl sulfoxide (Me_2SO, Carl Roth GmbH, 99,8%), 1,2-dimethoxyethane (DME, $C_2H_4(OMe)_2$, Fluka, 99.5%), 1,4-dioxane ($C_4H_8O_2$, Carl Roth GmbH, 99,5%) and pyridine (C_5H_5N, Fisher chemicals, 99.5%) were obtained from commercial sources. $Me_3NH[B_{11}H_{14}]$ was synthesized by slightly modified method[1] of Dunks *et al.*[2,3]. $K_2[B_{11}H_{11}]\cdot H_2O$ was synthesized by our reported method.[4] $BH_2Py_2I_3$ was synthesized according to the method described for BH_2Py_2I [5] by reacting a solution of $NaBH_4$ in pyridine with excess iodine.

For synthetic reactions in solutions in anhydrous HF, a reactor was used consisting of a 120-mL PFA bulb with an NS 29 socket standard taper (Bohlender, Lauda, Germany) in connection with a PFA NS 29 cone standard taper and a PFA needle valve (Bohlender, Lauda, Germany). The two parts were pressed together with a metal flange, and the reactor was leak-tight ($<10^{-5}$ mbar L s^{-1}) without use of grease.[6,7]

Caution: Na_2S, methanol, anhydrous HF and volatile boranes (for example B_2H_6 and $B_{10}H_{14}$) are toxic. Volatile boranes and organic solvents used herein are flammable. Anhydrous HF, acids, KOH, NaOH, K_2CO_3, Cs_2CO_3 are corrosive. For this reason, all syntheses described should be carried out in a fume hood.

Synthesis of $Na_2[B_{11}H_{11}]\cdot 3C_4H_8O_2$

The synthesis of $Na_2[B_{11}H_{11}]\cdot 3C_4H_8O_2$ was carried out similar to the synthesis of $K_2[B_{11}H_{11}]\cdot H_2O$.[4]

$$Me_3NH[B_{11}H_{14}] + 2\ NaOH + PbO_2 \rightarrow Na_2[B_{11}H_{11}] + Me_3N + PbO + 3\ H_2O$$

13.8 g (71.2 mmol) of $Me_3NH[B_{11}H_{14}]$ was reacted with a solution of 79.2 g (1.98 mol) of NaOH in 90 mL of water and 18.8 g (78.5 mmol) of PbO_2. The reaction mixture (black suspension) was cooled to room temperature with ice. Since $[PbB_{11}H_{11}]^{2-}$ (δ −5.3 (5B, o), −6.3 (1B, p), −11.5 (5B, m) ppm) was still present in the ^{11}B NMR spectrum after two days of reaction, the mixture was treated with

an additional 17.3 g (72.2 mmol) of PbO_2. After additional four days of reaction, only $[B_{11}H_{11}]^{2-}$ ($\delta(^{11}B)$ = −17.6 ppm) and small amounts of $[B(OH)_4]^-$ (approximately 4% of all boron atoms; $\delta(^{11}B)$ = 1.5 ppm) were detected in the ^{11}B NMR spectrum. The product was extracted in several portions with acetonitrile (12 x 40 mL). The combined acetonitrile solutions were mixed with 100 mL of dioxane and concentrated on a rotary evaporator at room temperature. The product was washed with approximately 100 mL of diethyl ether. The yield of $Na_2[B_{11}H_{11}]\cdot3C_4H_8O_2$ is 25.7 g (58.4 mmol, 82%).

Without the addition of dioxane, the product remained liquid after concentration on a rotary evaporator. Upon addition of dioxane to this liquid product, $Na_2[B_{11}H_{11}]\cdot5C_4H_8O_2\cdot2MeCN\cdot2H_2O$ immediately crystallized.

Synthesis of BPy_4I_3

BI_3 was prepared in situ from $Na[BH_4]$ and I_2.[8,9] BPy_4I_3 was prepared from BI_3 and pyridine.[10]

$3\ NaBH_4 + 8I_2 \rightarrow 3\ BI_3 + 3\ NaI + 4\ H_2 + 4\ HI$

$BI_3 + 4Py \rightarrow BPy_4I_3$

112 g (441 mmol) of I_2 were weighed and transferred to a 250 mL flask fitted with a reflux condenser. The reaction mixture was heated to approximately 120 °C under protective gas (iodine must melt). 4.0 g (106 mmol) of $Na[BH_4]$ were added to the reaction mixture in several portions. The reaction is very vigorous. After the reaction was complete (approx. 1-2 hours), the reaction mixture was treated with approximately 160 mL (2 mol) of pyridine and heated under reflux for one day. The reaction mixture was cooled to room temperature, filtered, and the crude product was washed with approximately 200 mL of acetone, approximately 200 mL of Et_2O, and air-dried. The crude product (46 g) is brown. It was dissolved in a mixture of 150 mL of water and 20 mL of 37% HCl, and the solution was filtered through Celite. The solution (215 g) was concentrated to approximately 80 g on a rotary evaporator (25 °C, 10 mbar), treated with 500 mL of acetone, and filtered. The product was washed with 300 mL of ethanol, 200 mL of acetone, and 100 mL of Et_2O. After air drying, 32 g of BPy_4I_3 were obtained (45 mmol, yield 42%).

A large excess of iodine should be avoided during the reaction, as pyridine reacts with iodine under these conditions to form $4\text{-}PyC_5H_4NHI_2$. The reaction of pyridine with I_2 is similar to the reactions of pyridine with Br_2,[11,12] $SOCl_2$[13] and Cl_2.[14] According to the reaction equation, approximately 75 g of iodine would also be sufficient. $4\text{-}PyC_5H_4NHI_2$ is not easily separated from BPy_4I_3 because their solubilities are similar. $4\text{-}PyC_5H_4NHI_2$ is more soluble in acetone and ethanol than BPy_4I_3. $4\text{-}PyC_5H_4NHI_2$ can be separated more easily with ethanol than with acetone. 2.1 g (5 mmol) of $4\text{-}PyC_5H_4NHI_2\cdot H_2O$ were separated, which was still contaminated with BPy_4I_3.

Reactions of BPy_4I_3 with HNO_3 (68%), $H[BF_4]$ (50%), and $H[PF_6]$ (60%) yielded small amounts of $BPy_4(NO_3)_3$, or $BPy_4[BF_4]_3$, or $BPy_4[PF_6]_3$, respectively, which were crystallized from water/HNO_3, water/$H[BF]_4$ or dimethyl sulfoxide, respectively by slow evaporation. BPy_4I_3 crystallizes from dimethyl sulfoxide solvate-free, from dimethyl sulfoxide/water as $BPy_4I_3\cdot H_2O$, and from HCl-acidified water as $BPy_4I_3\cdot2H_2O$.

Synthesis of $Na_3[Cu(B_{11}H_{11})_2]\cdot8H_2O$

$2Me_3NH[B_{11}H_{14}] + 1/2\ Cu_2O + 3NaOH + 9/2H_2O \rightarrow \quad Na_3[Cu(B_{11}H_{11})_2]\cdot8H_2O + 2Me_3N + 2\ H_2$

3.06 g (15.8 mmol) of $Me_3NH[B_{11}H_{14}]$ was reacted with a solution of 6.07 g (152 mmol) of NaOH in 7 mL of water and 6.97 g (48.7 mmol) of Cu_2O in a 100 mL round-bottom flask (with a plastic stopper and magnetic stir bar) at room temperature (22 °C) with vigorous stirring. A dark brown suspension formed and gas evolution occurred. The reaction mixture was diluted with 15 mL of water. To prevent oxidation of $[Cu(B_{11}H_{11})_2]^{3-}$ to $[Cu(B_{11}H_{11-x}(OH)_x)_2]^{3-}$, the reaction mixture was treated

with 0.12 g (0.7 mmol) of ascorbic acid. After two hours, no further gas evolution was observed and after additional two hours, the solution had turned deep green. The reaction mixture was stirred overnight. The mixture was treated with a solution of 4.37 g of $Na_2S{\cdot}xH_2O$ (60% Na_2S, 34 mmol) in 12 mL of water while cooling with water. Since the solution was still deep green, the reaction mixture was treated with another 15.47 g of $Na_2S{\cdot}xH_2O$ (60% Na_2S, 198 mmol) and 22 mL of water until the solution turned orange. The product was extracted with acetone. The acetone phase (approx. 150 g) was separated, filtered and concentrated. The crude product (approx. 3 g) was washed with diethyl ether, dissolved in acetone, and filtered off (44 g of solution). The solution was concentrated. The product was dissolved in water and filtered off (38 g solution), and slowly concentrated at room temperature. The $Na_3[Cu(B_{11}H_{11})_2]{\cdot}8H_2O$ that crystallized from the solution was separated from the mother liquor, washed with diethyl ether, and air-dried. Yield: 1.75 g (3.3 mmol, 41% of theory).

Further product can be isolated from the mother liquor. If KOH is used instead of NaOH, $K_3[Cu(B_{11}H_{11})_2]{\cdot}5H_2O$ can be synthesized analogously. It should be noted, however, that the solubility of $K_3[Cu(B_{11}H_{11})_2]{\cdot}5H_2O$ in water and acetone is lower than that of $Na_3[Cu(B_{11}H_{11})_2]{\cdot}8H_2O$. Acetonitrile could also be used instead of acetone. The NMR (^1H, ^{11}B) data are in Table S2. The DSC/DTG data are on Figure S2.

Synthesis of $(Et_3NH)_2[Cu(B_{11}H_{10})_2(OCHMe_2)]{\cdot}H_2O$

$2Me_3NH[B_{11}H_{14}] + CuO + 3NaOH + 4H_2O \rightarrow Na_3[Cu(B_{11}H_{11})_2]{\cdot}8H_2O + 2Me_3N + 3/2H_2$

$Na_3[Cu(B_{11}H_{11})_2]{\cdot}8H_2O + 3\ Et_3NHCl + Me_2CO \rightarrow (Et_3NH)_2[Cu(B_{11}H_{10})_2(OCHMe_2)]{\cdot}H_2O + H_2 + 3\ NaCl + Et_3N + 7\ H_2O$

3.02 g (15.6 mmol) of $Me_3NH[B_{11}H_{14}]$ was reacted with NaOH (6.02 g (151 mmol) in 12 mL water) and 3.94 g (44.0 mmol) of CuO at room temperature. A dark-gray suspension was formed, which was diluted with approximately 1 mL of water. Gas evolution was clearly observed for approximately 3 hours. After a reaction time of approximately 5 hours, the dark-brown reaction mixture was treated with $Na_2S{\cdot}xH_2O$ (60% Na_2S, 5.800 g (45 mmol) in 16 mL water). The product was extracted with acetone until the organic phase was only slightly colored. The acetone solution (yellow-brown, approximately 500-800 mL) was filtered through Celite and treated with Et_3NHCl (3.86 g (28 mmol) in 7 mL water). The precipitated NaCl was filtered off, and the solution was concentrated at room temperature. The product was dissolved in 800 mL of acetone, filtered off, and concentrated at 50-60 °C. The solution is yellow-orange. Initially, some Et_3NHCl crystallized. The product was dissolved in CH_2Cl_2 and filtered off (55.5 g of solution). The product was washed with a little water. Sparingly water-soluble $(Et_3NH)_2[Cu(B_{11}H_{10})_2(OCHMe_2)]{\cdot}H_2O$ (0.25 g, 0.41 mmol, 5% of theory) was obtained. The NMR (^1H, ^{11}B) data are in the Supporting Information (Tab. S4, Fig. S1). The DSC/DTG data are on Figure S5. Reaction of the aqueous phase with NaOH additionally afforded 0.8 g (1.5 mmol, 20% of theory) of $Na_3[Cu(B_{11}H_{11-x}(OH)_x)_2]{\cdot}8H_2O$ (x = 0.18).

Synthesis of $(Et_3NH)_3[Cu(B_{11}H_{11})_2]{\cdot}CuCl$

$2Me_3NH[B_{11}H_{14}] + CuO + 3NaOH + 4H_2O \rightarrow Na_3[Cu(B_{11}H_{11})_2]{\cdot}8H_2O + 2\ Me_3N + 3/2\ H_2$

$Na_3[Cu(B_{11}H_{11})_2]{\cdot}8H_2O + 3Et_3NHCl + CuCl \rightarrow (Et_3NH)_3[Cu(B_{11}H_{11})_2]{\cdot}CuCl + 3NaCl + 8H_2O$

2,84 g (14.7 mmol) of $Me_3NH[B_{11}H_{14}]$ was reacted with 6,15 g (154 mmol) of NaOH in 24 mL of water and 4,11 g (45.9 mmol) of CuO in a 100 mL beaker at room temperature. A dark gray suspension was formed, which was diluted with approximately 5 mL of water. After a reaction time of approximately 10 hours, the reaction mixture was extracted with acetone. The dark green acetone solution (approx. 180 g) was filtered off and concentrated. The crude product (1.8 g) was redissolved in acetone (dark green solution, 85 g), filtered off and concentrated. The dark green product (1,45 g) was

dissolved in 10 mL of water and reacted with 2,46 g (17.9 mmol) of Et_3NHCl in 11 g of water. An orange solution with a black precipitate formed. The mixture was treated with 88 g of acetone, which turned into a deep green solution. The solution (139 g) was filtered and concentrated. A crude black product was obtained that was dissolved in water and filtered. The aqueous solution was yellow-orange (453 g). Upon slow concentration of the aqueous solution, 1.2 g (1.6 mmol, 22% yield) of $(Et_3NH)_3[Cu(B_{11}H_{11})_2]\cdot CuCl$ crystallized. The mother liquor was treated with excess NaOH, concentrated, and extracted with acetone. 0.5 g (0.9 mmol, 12% yield) of $Na_3[Cu(B_{11}H_{11})_2]\cdot 8H_2O$ was obtained from the acetone solution. The NMR (^1H, ^{11}B) data are in Table S2.

Synthesis of $K_3[Cu(B_{11}H_{11})_2]\cdot 5H_2O$

$2Me_3NH[B_{11}H_{14}] + CuO + 3NaOH + 4H_2O \rightarrow Na_3[Cu(B_{11}H_{11})_2]\cdot 8H_2O + 2Me_3N + 3/2H_2$

$Na_3[Cu(B_{11}H_{11})_2]\cdot 8H_2O + 3Et_3NHCl \rightarrow (Et_3NH)_3[Cu(B_{11}H_{11})_2] + 3NaCl + 8H_2O$

$(Et_3NH)_3[Cu(B_{11}H_{11})_2] + 3KOH + 2H_2O \rightarrow K_3[Cu(B_{11}H_{11})_2]\cdot 5H_2O + 3Et_3N$

1.04 g (5.38 mmol) of $Me_3NH[B_{11}H_{14}]$ was reacted with 4.2 g (105 mmol) of NaOH in 40 mL of water and 2.5 g (31.4 mmol) of CuO in a 100 mL beaker at room temperature. A dark gray suspension was formed. After a reaction time of approximately 10 hours, the reaction mixture was extracted with acetone. The dark green acetone solution was filtered off and concentrated. The product was again dissolved in acetone (dark green solution), filtered off and concentrated. The dark green product was dissolved in 10 mL of water and reacted with 3.3 g (24.0 mmol) of Et_3NHCl in 10 mL of water. An orange solution with a black precipitate was formed. The solution was filtered off and treated with excess KOH, concentrated, and extracted with acetonitrile. From the acetonitrile solution, 0.858 g (1.6 mmol, 59% yield) of $K_3[Cu(B_{11}H_{11})_2]\cdot 5H_2O$ was obtained, which was contaminated with $K_3[Cu(B_{11}H_{11})(B_{11}H_{10}(OH))]\cdot 5H_2O$. The salt was dissolved in a mixture of 80 mL MeCN and 40 mL (500 mmol) pyridine, cooled with ice, and treated with 3 mL (30 mmol) $POCl_3$. After approximately 15 hours, the solution was concentrated, treated with excess KOH, and extracted with acetonitrile. After concentration, 0.7 g (1.3 mmol, 49% yield) of $K_3[Cu(B_{11}H_{11})_2]\cdot 5H_2O$ was obtained without contamination of $K_3[Cu(B_{11}H_{11})(B_{11}H_{10}(OH))]\cdot 5H_2O$, since $K_5[Cu(B_{11}H_{11})(B_{11}H_{10}(OPO_3))]$ is not extracted with acetonitrile. The NMR (^1H, ^{11}B) data are in Table S2. The DSC/DTG data are on Figure S2.

Small amounts of $[Co(NH_3)_6][Cu(B_{11}H_{11})_2]\cdot 2H_2O$ (50 mg, 0.1 mmol) and $Cs_3[Cu(B_{11}H_{11})_2]\cdot H_2O$ (300 mg, 0.4 mmol) can be obtained from $K_3[Cu(B_{11}H_{11})_2]\cdot 5H_2O$ by reaction with $[Co(NH_3)_6]Cl_3$ or Cs_2CO_3.

Synthesis of $K_3[Cu(B_{11}H_{11})_2]$

$K_2[B_{11}H_{11}]$ (1.00 g, 4.80 mmol) was dissolved in water (10 mL) and Cu_2O (189 mg, 1.32 mmol) was added. The suspension was stirred at room temperature for 25 hours. Afterwards it was filtered, washed with water and the orange solution was evaporated to dryness. The orange residue was washed with cold ethanol (10 mL) and dried in vacuo.
Yield: 740 mg (70 %) of a yellow solid. The NMR (^1H, ^{11}B) and HRMS data are in the Supporting Information. The DSC/STA data are on Figure S6.

Synthesis of $BPy_4[Cu(B_{11}H_{11})_2]$

$Na_3[Cu(B_{11}H_{11})_2]\cdot 8H_2O + BPy_4I_3 \rightarrow BPy_4[Cu(B_{11}H_{11})_2] + 3NaI + 8H_2O$

0.146 g (0.27 mmol) of $Na_3[Cu(B_{11}H_{11})_2]\cdot 8H_2O$ was dissolved in water and treated with an aqueous solution of 0.245 g (0.35 mmol) of BPy_4I_3 (yellow). A yellow precipitate immediately formed. It was washed with water, acetone, and diethyl ether. Yield: 0.15 g (0.23 mmol, 85% yield).

$BPy_4[Cu(B_{11}H_{11})_2]$ is practically insoluble in water, acetone (< 25 mg in 100 g), acetonitrile (< 25 mg in 100 g), ethanol, methanol (< 10 mg in 100 g), and dimethylformamide (< 30 mg in 100 g). $BPy_4[Cu(B_{11}H_{11})_2]$ is soluble in dimethyl sulfoxide (> 1.5 g in 100 g).

When acetone diffuses into a solution of $BPy_4[Cu(B_{11}H_{11})_2]$ dimethyl sulfoxide at +5 °C, $BPy_4[Cu(B_{11}H_{11})_2]\cdot5Me_2SO$ crystallizes. The crystals of $BPy_4[Cu(B_{11}H_{11})_2]\cdot5Me_2SO$ are unstable at room temperature and decompose to result in $BPy_4[Cu(B_{11}H_{11})_2]$. Upon slow evaporation of dimethyl sulfoxide from a solution of $BPy_4[Cu(B_{11}H_{11})_2]$ or upon diffusion of acetone into a solution of $BPy_4[Cu(B_{11}H_{11})_2]$ in dimethyl sulfoxide at room temperature (22 °C), $BPy_4[Cu(B_{11}H_{11})_2]$ crystallizes. The DSC/DTG data are on Figure S4.

Synthesis of $Cs_3[Cu(B_{11}H_{11-x}F_x)_2]$

$Cs_3[Cu(B_{11}H_{11})_2] + 2xHF \rightarrow Cs_3[Cu(B_{11}H_{11-x}F_x)_2] + 2xH_2$

Approximately 100 mg (0.14 mmol) of $Cs_3[Cu(B_{11}H_{11})_2]\cdot H_2O$ was dried under vacuum in a 100 mL Kel-F flask. Approximately 20 mL of anhydrous HF was condensed onto the resulting $Cs_3[Cu(B_{11}H_{11})_2]$. After three days at room temperature, all volatile components were removed under vacuum. The product was dissolved in water (approximately 100 mL), neutralized with Cs_2CO_3, filtered, and concentrated. For purification, the product was extracted with acetonitrile, and the solution was filtered and concentrated. After crystallization from water, 95 mg (0.11 mmol, 82% yield) of $Cs_3[Cu(B_{11}H_{11-x}F_x)_2]$ (x is approximately 4) were obtained.

Synthesis of $K_3[Ag(B_{11}H_{11})_2]\cdot5H_2O$

$2K_2[B_{11}H_{11}]\cdot H_2O + 1/2Ag_2O + 11/2H_2O \rightarrow K_3[Ag(B_{11}H_{11})_2]\cdot5H_2O + 3KOH$

2.13 g (9.42 mmol) of $K_2[B_{11}H_{11}]\cdot H_2O$ were dissolved in 11.5 g of water and reacted with 0.54 g (2.34 mmol) of Ag_2O. The solution immediately turned orange. Since the solution was warm, it was cooled with ice. After one hour, the reaction mixture was treated with 2.4 g (17 mmol) of K_2CO_3 followed by the addition of additional 3.6 g (26 mmol) of K_2CO_3. The reaction mixture was extracted three times with 60 mL of acetonitrile each time. The combined acetonitrile solution was filtered off and concentrated. The product was washed three times with 60 mL of diethyl ether each time and air-dried. Yield: 2.66 g (4.62 mmol, 99% yield). An orange, microcrystalline solid was obtained. The NMR (^1H, ^{11}B) data are in Table S2. The DSC/DTG data are on Figure S2.

In a similar reaction, 0.256 g (0.44 mmol, 47%) of $K_3[Ag(B_{11}H_{11})_2]\cdot5H_2O$ was obtained from 420 mg (1.86 mmol) of $K_2[B_{11}H_{11}]\cdot H_2O$ and 108 mg (0.47 mmol) of Ag_2O.

$BPy_4[Ag(B_{11}H_{11})_2]$ was obtained by the reaction of $K_3[Ag(B_{11}H_{11})_2]\cdot5H_2O$ with $BPy_4(NO_3)_3$ in aqueous solution similar to the syntheses of $BPy_4[M(B_{11}H_{11})_2]$ (M = Cu, Au). The DSC/DTG data are on Figure S4.

Synthesis of $K_3[Ag(B_{11}H_{11})_2]$

$K_2[B_{11}H_{11}]$ (1.00 g, 4.80 mmol) was dissolved in water (12 mL) and Ag_2O (286 mg, 1.23 mmol) was added. The suspension was stirred at room temperature for 7 days. Afterwards it was filtered, and the orange solution was evaporated to dryness. The orange brown residue was dissolved in ethyl acetate (20 mL) and filtered. The bright orange solution was evaporated to dryness and dried in vacuo.
Yield: 844 mg (72%). The NMR (^1H, ^{11}B) and HRMS data are in the Supporting Information. The DSC/STA data are on Figure S7.

Synthesis of $Na_3[Au(B_{11}H_{11})_2]\cdot8H_2O$

$Au + 1/2I_2 + C_4H_8S \rightarrow C_4H_8SAuI$

$C_4H_8SAuI + 2Na_2[B_{11}H_{11}]\cdot3C_4H_8O_2 \rightarrow Na_3[Au(B_{11}H_{11})_2] + 6C_4H_8O_2 + C_4H_8S + NaI$

0.82 g (4.18 mmol) of Au was reacted with 10 mL of THT (C_4H_8S) and 0.55 g of I_2 and heated to 60 °C until the gold dissolved. The reaction mixture was treated at room temperature with $Na_2[B_{11}H_{11}]\cdot3C_4H_8O_2$ in 20 mL of acetonitrile. A dark green suspension formed. After a reaction time of half an hour, all volatile components were removed under vacuum. The product was dissolved in 40 mL of acetonitrile, filtered off, and concentrated under vacuum. The residue that was insoluble in acetonitrile was largely elemental gold. The product from the acetonitrile solution was washed with a 1:1 mixture of dimethoxyethane (approx. 150 mL) and diethyl ether (approx. 150 mL). The residue insoluble in DME/Et_2O was dissolved in approximately 50 mL of acetonitrile, the acetonitrile solution was filtered off, and the mixture was concentrated. The yield of the product obtained from the acetonitrile solution ($Na_3[Au(B_{11}H_{11})_2]\cdot8H_2O$) was 1.5 g (2.46 mmol, 59% yield). The NMR (^1H, ^{11}B) data are in Table S2.

Synthesis of $K_3[Au(B_{11}H_{11})_2]\cdot2H_2O$ from $Na_3[Au(B_{11}H_{11})_2]\cdot8H_2O$

$Na_3[Au(B_{11}H_{11})_2]\cdot8H_2O + 3/2K_2CO_3 \rightarrow K_3[Au(B_{11}H_{11})_2]\cdot2H_2O + 3/2Na_2CO_3 + 5H_2O$

1.5 g (2.46 mmol) of $Na_3[Au(B_{11}H_{11})_2]\cdot8H_2O$ was dissolved in approximately 20 mL of water and reacted with 3.13 g (23 mmol) of K_2CO_3 in approximately 10 mL of water, and the solution was filtered off. The aqueous solution was concentrated, and the product was extracted with approximately 60–70 mL of acetonitrile. The acetonitrile solution was filtered off, concentrated, and washed with approximately 75 mL of DME. The yield of $K_3[Au(B_{11}H_{11})_2]\cdot2H_2O$ was 1.35 g (2.21 mmol, 53% yield). Since the product was still brown, it was dissolved in approximately 5 mL of water and separated from the impurities by chromatography (cellulose, 10 cm, Ø 2.2 cm). The pink-colored impurities (approximately 20 mg were obtained) migrate significantly more slowly on the column. Presumably, the pink-colored impurities are colloidal gold. The yellow solution of the product (approximately 20 g) was concentrated. The product was dissolved in approximately 5 mL of water, reacted with approximately 2 g (15 mmol) of K_2CO_3, and extracted with approximately 50 mL of acetonitrile. After concentration of the acetonitrile solution, 1.2 g (1.97 mmol, 47% yield based on Au) of $K_3[Au(B_{11}H_{11})_2]\cdot2H_2O$ (yellow) was obtained. The NMR (^1H, ^{11}B) data are in Table S2 and Figure 2. The DSC/DTG data are on Figure S2.

Synthesis of $K_3[Au(B_{11}H_{11})_2]\cdot2H_2O$

$NaAuCl_4\cdot2H_2O + EtOH + C_4H_8S \rightarrow C_4H_8SAuCl$

$C_4H_8SAuCl + 2K_2[B_{11}H_{11}]\cdot H_2O \rightarrow K_3[Au(B_{11}H_{11})_2]\cdot2H_2O + KCl + C_4H_8S$

$K_3[Au(B_{11}H_{11})_2]\cdot2H_2O + BPy_4I_3 \rightarrow BPy_4[Au(B_{11}H_{11})_2] + 3KI + H_2O$

0.86 g (2.16 mmol) of $Na[AuCl_4]\cdot2H_2O$ was dissolved in aqua regia ($HCl+HNO_3$) and the solution was concentrated to 5 mL. The solution was diluted with ca. 5 mL of water and treated with 20 mL EtOH and 0.75 mL THT (C_4H_8S, 8.5 mmol). An orange precipitate of C_4H_8SAuCl formed, immediately. C_4H_8SAuCl was separated, dissolved in approximately 20 mL MeCN, and treated with 0.59 g (2.59 mmol) of $K_2[B_{11}H_{11}]\cdot H_2O$. A black suspension formed immediately. After a reaction time of half an hour, all volatile components were removed under vacuum. The product was dissolved in 40 mL acetonitrile, filtered off, and concentrated under vacuum. The residue insoluble in acetonitrile contained, among other substances, elemental gold. The product obtained from the acetonitrile solution was washed with approximately 20 mL of CH_2Cl_2 and dissolved in approximately 15 mL of acetonitrile. The acetonitrile solution was concentrated, and the product was dissolved in 5 mL water. The aqueous solution was filtered and concentrated. The resulting $K_3[Au(B_{11}H_{11})_2]\cdot2H_2O$ (0.32 g, 0.52 mmol, 40%

yield) was brown and it was purified by chromatography. The NMR (^{1}H, ^{11}B) data are in Table S2 and Figure 2. The DSC/DTG data are on Figure S2.

0.32 g (0.61 mmol) of $K_3[Au(B_{11}H_{11})_2]\cdot 2H_2O$ was dissolved in approximately 40 g of water (brown solution) and reacted with 0.51 g (0.71 mmol) of BPy_4I_3 in 17 g of water. A precipitate formed immediately, which was filtered off and washed with 100 mL of water and 300 mL of acetone. The product was washed with diethyl ether and air-dried. The yield of $BPy_4[Au(B_{11}H_{11})_2]$ was 0.26 g (0.33 mmol, 25% yield based on $Na[AuCl_4]\cdot 2H_2O$). The DSC/DTG data are on Figure S4.

Synthesis of $K_3[Au(B_{11}H_{11})_2]$

$K_2[B_{11}H_{11}]$ (14.5 mg, 69.6 µmol) and THTAuCl (11.7 mg, 34.8 µmol) were dissolved in CD_3CN (0.5 mL). After two days at room temperature the product was crystallized by diffusion of Et_2O into the black CD_3CN solution. The crystals were separated, dried, dissolved in CH_3CN and crystalized once more by diffusion of Et_2O into the acetonitrile solution. The supernatant solution was separated, and the colorless crystals were dried in vacuo. The NMR (^{1}H, ^{11}B) data are in the Supporting Information. The DSC/STA data are on Figure S8.

Synthesis of $K_3[Au(B_{11}H_{11-x}F_x)_2]$

$K_3[Au(B_{11}H_{11})_2] + 2xHF \rightarrow K_3[Au(B_{11}H_{11-x}F_x)_2] + 2xH_2$

Ca. 215 mg (0.35 mmol) of $K_3[Au(B_{11}H_{11})_2]\cdot 2H_2O$ was dried under vacuum overnight at 60 °C in a 100 mL Kel-F flask. Approximately 10 mL of anhydrous HF was condensed onto the resulting $K_3[Au(B_{11}H_{11})_2]$. After one day at room temperature and two days at 50-60 °C, all volatile components were removed under vacuum. The product was washed with diethyl ether, dissolved in water (ca. 5 mL), neutralized with K_2CO_3, and extracted with acetonitrile (approximately 50 mL). The acetonitrile solution was filtered off and concentrated. After crystallization from acetonitrile, 208 mg (0.26 mmol, 74 % yield) of $K_3[Au(B_{11}H_{11-x}F_x)_2]\cdot 4H_2O$ (x is ca. 4) were obtained.

Synthesis of $(n\text{-}Bu_4N)_3[M(B_{11}H_{11})_2]$ (M = Cu, Ag, Au)

$(n\text{-}Bu_4N)_3[M(B_{11}H_{11})_2]$ are formed quantitatively in the reaction of aqueous solution of $K_3[M(B_{11}H_{11})_2]\cdot xH_2O$ with aqueous solution of $n\text{-}Bu_4NBr$ (M = Cu, Au) or $n\text{-}Bu_4NHSO_4$ neutralized with NaOH (M = Ag). The NMR (^{1}H, ^{11}B) data are in Table S2. The DSC/DTG data are on Figure S3.

We were also able to obtain good single crystals of $(Bu_4N)_3[M(B_{11}H_{11})_2]\cdot 0.5Me_2SO\cdot 0.5H_2O$. For technical reasons, we have not yet been able to measure good data sets. For measurements at 200 K is the space group Pbcn and Z = 8. Lattice parameter are: a = 44.5335(14), b = 13.6354(5), c = 23.4304(12) Å, V = 14227.7(10) Å^3 for M = Cu; a = 44.5102(6), b = 13.7434(2), c = 23.2781(3) Å, V = 14239.7(4) Å^3 for M = Ag; a = 44.6054(9), b = 13.7146(3), c = 23.4221(4) Å, V = 14328.4(5) Å^3 for M = Au.

Synthesis of $[n\text{-}Bu_4N]_3[Cu(B_{11}H_{11})_2]$

$K_2[B_{11}H_{11}]$ (1.00 g, 4.80 mmol) was dissolved in water (10 mL), Cu_2O (206 mg, 1.44 mmol) was added, and the mixture was stirred at room temperature for 5 days. The orange aqueous solution was filtered and $[Bu_4N]Br$ (2.56g, 7.94 mmol) dissolved in water was added to the filtrate. The orange precipitate was filtered, washed with water and dried in vacuo.

Yield: 1.90 g (75 %) of a yellow solid. The NMR (^{1}H, ^{11}B, ^{13}C) and HRMS data are in the Supporting Information. The DSC/STA data are on Figure S9.

Synthesis of $[n\text{-}Bu_4N]_3[Ag(B_{11}H_{11})_2]$

$K_2[B_{11}H_{11}]$ (202 mg, 0.97 mmol) was dissolved in water (5 mL) and Ag_2O (56 mg, 0.24 mmol) was added. The mixture was stirred at room temperature for 18 hours The aqueous solution was filtered and $[Bu_4N]Br$ (236 mg, 0.72 mmol), dissolved in water was added. The precipitate was filtered, washed with water and dried in vacuo.

Yield: 249 mg (95%). The NMR (^1H, ^{11}B, ^{13}C) and HRMS data are in the Supporting Information. The DSC/STA data are on Figure S10.

Crystallography

Single crystals suitable for an X-ray diffraction study (Tab. S1) were attached to a goniometer head. The data collection was performed using an Oxford Diffraction Gemini E Ultra diffractometer with a 2K × 2K EOS CCD camera, a four-circle goniometer with κ geometry, a sealed-tube Mo radiation source, and an Oxford Instruments Cryojet cooling unit. Some structures were measured with Rigaku XtaLAB mini II ($Na_3[Cu(B_{11}H_{11})_2]\cdot 8H_2O$, 200 K; $(n\text{-}Bu_4N)_3[M(\eta^5\text{-}B_{11}H_{11})_2]\cdot 0.5H_2O\cdot 0.5Me_2SO$, 200 K, M = Cu, Ag, Au). Processing of the raw data, scaling of diffraction data, and the application of an empirical absorption correction were performed with the CrysAlisPro program.[15] The structures were solved by direct methods and refined against F^2.[16–19] The graphics were prepared with the program Diamond.[20] In the case of $K_3[Cu(B_{11}H_{11})_2]\cdot 3.5H_2O\cdot 0.5KOH$ single crystals suitable for X-ray diffraction studies were grown by slow evaporation of an H_2O solution. $[n\text{-}Bu_4N]_3[Cu(B_{11}H_{11})_2]$ and $[n\text{-}Bu_4N]_3[Ag(B_{11}H_{11})_2]$ were crystallized by slow evaporation of a MeCN solution as well as by diffusion of Et_2O into a solution of $(n\text{-}Bu_4N)_3[Cu(B_{11}H_{11})_2]$. Both methods resulted in orange, large crystals with the same properties. $K_3[Au(B_{11}H_{11})_2]\cdot 3(CH_3CN)$ was crystallized by slow diffusion of Et_2O into a MeCN solution of the respective cluster. Crystal data of $K_3[Cu(B_{11}H_{11})_2]\cdot 3.5H_2O\cdot 0.5KOH$, $K_3[Ag(B_{11}H_{11})_2]\cdot 2H_2O$, $(n\text{-}Bu_4N)_3[Au(B_{11}H_{11})_2]$ and $[n\text{-}Bu_4N]_3[Ag(\eta^2\text{-}B_{11}H_{11})_2]$ were collected on on a *XtaLAB* Synergy, Dualflex diffractometer with a hybrid pixel array detector, using Mo*Kα* radiation (micro-focus sealed X-ray tube λ_{Mo} = 0.71073 Å). The data of $[n\text{-}Bu_4N]_3[Ag(\eta^5\text{-}B_{11}H_{11})_2]$ was collected on the same machine but using $Cu_{K\alpha}$ radiation (rotating-anode X-ray tube, λ_{Cu} = 1.54184 Å) instead.

Crystal data of $[n\text{-}Bu_4N]_3[Cu(B_{11}H_{11})_2]$ and $K_3[Au(B_{11}H_{11})_2]\cdot 3(CH_3CN)$ were collected on a *XtaLAB* Synergy-R diffractometer with a hybrid pixel array detector, using $Cu_{K\alpha}$ radiation (rotating-anode X-ray tube, λ_{Cu} = 1.54184 Å). Measurements of $[n\text{-}Bu_4N]_3[Ag(B_{11}H_{11})_2]$ showed different structures depending on the temperature. To study the reversibility of the interconversion of the η^2- and η^5-coordination mode of Ag a single crystal was picked and mounted onto the goniometer head of a *XtaLAB* Synergy, Dualflex diffractometer equipped with a hybrid pixel array detector at room temperature. The crystal was cooled with a rate of 180 K/h and data was collected at 273, 250, 230, 210, 190, 180, 170, 160, 150, 140, 130, 120, 110, and 100 K. The temperature was held at 100 K for 60 minutes and after collecting data at this temperature once more, the crystal was warmed with a rate of 180 K/h. Data was collected at 110, 120, 130, 140, 150, 160, 170, 180, 190, 210, 230, 250, and 273 K. During cooling, η^5 coordination was found at temperatures of 120 K or higher, whereas at lower temperatures η^2 coordination was observed. During warming, the η^2 coordination remained until a temperature of 180 K. Starting at temperatures of 180 K η^5 coordination was observed. The structures of 110 K when cooling down and 170 K when warming up represent intermediate structures.

Crystallographic data for the structures in this paper (Tab. S1, Fig. S1-S5) have been deposited with the Cambridge Crystallographic Data Centre, CCDC, 12 Union Road, Cambridge CB21EZ, UK.

We obtained the best crystal structures with the $[M(B_{11}H_{11})_2]^{3-}$ anions for $K_3[M(B_{11}H_{11})_2]\cdot 2H_2O$ (M = Cu, Ag, and Au) (Fig. 2, S2 in the Supporting Information). In the case of M = Cu and Ag, $K_3[M(B_{11}H_{11})_2]\cdot 2H_2O$ was crystallized from water at room temperature (22 °C) in the presence of K_2CO_3,

as otherwise $K_3[M(B_{11}H_{11})_2]\cdot5H_2O$ (M = Cu and Ag) crystallized. In the crystal structure of $K_3[Cu(B_{11}H_{11})_2]\cdot2H_2O$, the vibrational ellipsoids of the potassium atoms and oxygen atoms are significantly larger than in $K_3[M(B_{11}H_{11})_2]\cdot5H_2O$ (M = Ag and Au). For this purpose, the positions of K1 (K1-K3 0.306 Å) and K2 (K2-K2 0.613 Å) were split (Tab. S13). From concentrated K_2CO_3 solutions, $K_3[Cu(B_{11}H_{11})_2]\cdot H_2O$ crystallized ($P4_122$, Z = 4, a = b = 8.2250(7) Å, c = 31.476(7) Å, at 100 K and a = b = 8.1914(10) Å, c = 31.47(2) Å, at 295 K). Measured data sets of $K_3[Cu(B_{11}H_{11})_2]\cdot H_2O$ could not be properly integrated due to very broad reflection profiles (e1 = 2.3 °, e2 = 1.5 °, e3 = 4.1 °). This resulted in reflections (h, k, l) strongly overlapping with (h, k, l±1). The crystals of $K_3[M(B_{11}H_{11})_2]\cdot5H_2O$ (M = Cu and Ag) tend to form twins, making it difficult to find non-twinned crystals. The crystal structures of $K_3[M(B_{11}H_{11})_2]\cdot5H_2O$ (M = Cu and Ag) are similar to those of $K_3[Cu(B_{11}H_{11})_2]\cdot2H_2O$.

In contrast to $A_3[M(B_{11}H_{11})_2]$ (M = Cu, Ag, Au; A = Na, K, Cs), $(NH_4)_3[Cu(B_{11}H_{11})_2]\cdot3NH_4Cl$ crystallized from aqueous solutions without water molecules in the crystal structure. We were able to obtain crystals of $(Et_3NH)_3[Cu(B_{11}H_{11-x}(OH)_x)_2]\cdot yH_2O$ (x = 0.11, y = 0.88) suitable for X-ray crystallography. However, these crystals were twinned. Some of the Et_3NH^+ cations are disordered.

Of the compounds with multiply charged cations, single crystals of compounds with the cations BPy_4^{3+} and $[Co(NH_3)_6]^{3+}$ were obtained. $BPy_4[M(B_{11}H_{11})_2]$ (M = Cu, Au) is practically insoluble in water and most organic solvents. Suitable single crystals of $BPy_4[M(B_{11}H_{11})_2]$ were obtained by slow evaporation of the solvent from the solution in dimethyl sulfoxide. These crystal structures exhibit no disorder. Crystals of $BPy_4[Cu(B_{11}H_{11})_2]\cdot5Me_2SO$ were obtained by slowly diffusing acetone into the solution of $BPy_4[Cu(B_{11}H_{11})_2]$ in dimethyl sulfoxide at 4 °C. These decompose rapidly at room temperature to form $BPy_4[Cu(B_{11}H_{11})_2]$ powder. Crystals of $[Co(NH_3)_6]_3[Cu(B_{11}H_{11-x}(OH)_x)_2]\cdot2H_2O$ (x = 0.15) can be obtained from aqueous solutions. In addition to the disorder caused by OH groups in the anion, the hydrogen atoms in the cation are also disordered.

The NMR data of $(n\text{-}Bu_4N)_3[Ag(B_{11}H_{11})_2]$ suggests an η^5-coordination mode of Ag^{+V} as it seems to be highly symmetrical, similar to the compounds containing copper and gold. After crystallizing $(n\text{-}Bu_4N)_3[Ag(B_{11}H_{11})_2]$ large, orange single crystals were studied with single crystal X-ray diffraction at various temperatures. Rapid cooling caused the crystals to fragment. The phase transition was kinetically inhibited and started only with a delay. Once initiated, it occured abruptly, often causing crystals to burst. Therefore, a suitable crystal was cooled gradually, and the crystal structure was determined at various temperatures (Fig. 5). The temperature was lowered to 100 K and the unit cell slightly changed. The structure revealed a silver atom which was shifted towards one side of the five membered *ortho*-rings of the boron clusters, so that the silver atom became η^2-coordinated. Besides the elongation of all distances (Figure 6), the structure also shows a significant distortion as well as tilting of the cluster structure similar to the calculated η^2 isomer of $[Ag^{+I}(closo\text{-}B_{11}H_{11})_2]^{3-}$ depicted in Figure 7 c). The measurement was repeated after one hour at 100 K with the same result. After this, the single crystal was warmed stepwise and measured again at different temperatures. While at 160 K the structure remained unchanged with an η^2-coordination mode, it shifted back to the η^5-coordinated structure upon warming up to a higher temperature. The cell volume of the η^5-structure was always smaller than the volume of the η^2 structure at the same temperature. The cell volumes decreased upon cooling of the crystal and, in the case of the η^5-structure the volume reached a minimum volume of 3368 $Å^3$. While the majority of the cell volumes determined follow a clear trend, two of them were found to be outliners. Namely, the one determined at 110 K during cooling and the one at 170 K when warming up. While the cell lengths and angles are very similar to those of the η^5 structure, the volume of both structures is surprisingly small. A stable refinement of the structures was not possible, but structure solution suggests an intermediate structure in which the silver atom reveals either an η^5 or an η^2 coordination for both ligands, respectively. Noteworthy, a mixed structure with one ligand being η^5 and the second η^2 coordinated to silver, i.e. $[Ag^{+III}(\eta^2\text{-}B_{11}H_{11})(\eta^5\text{-}(B_{11}H_{11})]^{3-}$, was not observed. During the conversion from η^5 to η^2, the color of the crystals becomes significantly lighter (Fig. S45). The frequency of the ν(BH) vibration also decreases slightly (Fig. S46). These observations are consistent with theoretical calculations (Fig. S32-S38, S47).

This conversion of the bis-η^5 to the bis-η^2 isomer at lower temperatures is unusual because the bis-η^2 isomer is slightly larger and has a higher entropy. The calculated entropy change $\Delta S_{298} = (\Delta H_{298} - \Delta G_{298})/T$ at 298 K for η^2 to η^5 transition is -111.7 $J \cdot K^{-1} \cdot mol^{-1}$ (vacuum) or -80.5 $J \cdot K^{-1} \cdot mol^{-1}$ (SCRF(Solvent=Water)) for B3LYP/aug-cc-pvtz(-pp). The main contribution to the entropy change comes from low-frequency vibrations, which are strongly hindered in the solid. For this reason, it is possible that the entropy change ΔS_{298} in $(n\text{-}Bu_4N)_3[Ag(B_{11}H_{11})_2]$ is positive. This would mean that for the transition bis-η^2 to bis-η^5 in $(n\text{-}Bu_4N)_3[Ag(B_{11}H_{11})_2]$ ΔH_{298} and ΔS_{298} are positive. In the cases of $(n\text{-}Bu_4N)_3[Cu(B_{11}H_{11})_2]$ and $(n\text{-}Bu_4N)_3[Au(B_{11}H_{11})_2]$, such a transformation was not observed. This correlates with theoretical calculations showing that η^5 coordination in the condensed state is more stable than η^2 coordination for $[Cu(B_{11}H_{11})_2]^{3-}$ and $[Au(B_{11}H_{11})_2]^{3-}$ (Tab. 1). In the case of $[Ag(B_{11}H_{11})_2]^{3-}$, the relative stabilities of complexes with η^5 and η^2 coordination are similar. Since the energy differences between the rotamers are not large (Tab. S14, Fig. S48), the geometry of the η^2 isomer of $[Ag(B_{11}H_{11})_2]^{3-}$ is adapted to the crystal lattice of $(n\text{-}Bu_4N)_3[Ag(B_{11}H_{11})_2]$. Thus, the value of the dihedral angle between the boron atoms coordinated to silver (139.0(2) °) is closer to that of C_{2v} rotamer (180 °) than to that of the most stable rotamer C_2 (80 °).

Table S1. Summarized crystallographic data which were determined within the scope of this work.

Chemical formula	CCDC number	*T* (K)	Space group	R_1 [a)]
$Na_3[Cu(B_{11}H_{11})_2]\cdot8H_2O$	2421085	150	*I*2/*m* (12)	0.0250; 0.0321
$Na_3[Cu(B_{11}H_{11})_2]\cdot8H_2O$	2449213	200	*I*2/*m* (12)	0.0379; 0.0439
$K_3[Cu(B_{11}H_{11})_2]\cdot5H_2O$	2421080	100	*P*−1 (2)	0.0319; 0.0399
$K_3[Cu(B_{11}H_{11})_2]\cdot2H_2O$	2421088	100	*Pnnm* (58)	0.0278; 0.0333
$K_3[Cu(B_{11}H_{11})_2]\cdot3.5H_2O\cdot0.5KOH$	2454502	100	*P*2$_1$/*c* (14)	0.0346; 0.0498
$(NH_4)_3[Cu(B_{11}H_{11})_2]\cdot3NH_4Cl$	2421078	150	*I*2/*m* (12)	0.0214; 0.0219
$BPy_4[Cu(B_{11}H_{11})_2]$	2421077	150	*C*2/*c* (15)	0.0408; 0.0636
$BPy_4[Cu(B_{11}H_{11})_2]\cdot5Me_2SO$	2421089	150	*P*2$_1$/*m* (11)	0.0565; 0.0969
(*n*-Bu$_4$N)$_3$[Cu(η^5-B$_{11}$H$_{11}$)$_2$]	2454491	100	*P*−1 (2)	0.0572; 0.0729
$Na_3[Cu(B_{11}H_{11-x}(OH)_x)_2]\cdot8H_2O$; x = 0.19	2421086	150	*I*2/*m* (12)	0.0188; 0.0192
$K_3[Cu(B_{11}H_{11-x}(OH)_x)_2]\cdot5H_2O$; x = 0.14	2421083	150	*P*2$_1$/*c* (14)	0.0237; 0.0323
$[Co(NH_3)_6]_3[Cu(B_{11}H_{11-x}(OH)_x)_2]\cdot2H_2O$; x = 0.15	2421081	150	*P*−1 (2)	0.0269; 0.0381
$(Et_3NH)_3[Cu(B_{11}H_{11-x}(OH)_x)_2]\cdot0.88H_2O$; x = 0.11	2421087	150	*P*−1 (2)	0.0480; 0.0698
$(Et_3NH)_3[Cu(B_{11}H_{11-x}(OH)_x)_2]\cdot0.95CuCl$; x = 0.25	2421084	150	*P*−1 (2)	0.0319; 0.0474
$(Et_3NH)_3[Cu(B_{11}H_{10})_2(OCHMe_2)]\cdot H_2O$	2421082	150	*P*2$_1$/*c* (14)	0.0346; 0.0443
$Cs_3[Cu(B_{11}H_{11-x}F_x)_2]$; x = 4.26	2421079	150	*Cmca* (64)	0.0257; 0.0376
$K_3[Ag(B_{11}H_{11})_2]\cdot2H_2O$	2421091	100	*Pnnm* (58)	0.0139; 0.0179
$K_3[Ag(B_{11}H_{11})_2]\cdot2H_2O$	2454493	100	*Pnnm* (58)	0.0193; 0.0220
$K_3[Ag(B_{11}H_{11})_2]\cdot2H_2O$	2421090	150	*Pnnm* (58)	0.0145; 0.0180
$K_3[Ag(B_{11}H_{11})_2]\cdot5H_2O$	2421092	100	*P*−1 (2)	0.0291; 0.0372
(*n*-Bu$_4$N)$_3$[Ag(η^2-B$_{11}$H$_{11}$)$_2$]	2454494	100	*P*−1 (2)	0.0600; 0.0934
(*n*-Bu$_4$N)$_3$[Ag(η^5-B$_{11}$H$_{11}$)$_2$]	2454490	173	*P*−1 (2)	0.0342; 0.0356
(*n*-Bu$_4$N)$_3$[Ag(η^5-B$_{11}$H$_{11}$)$_2$]·0.5H$_2$O·0.5Me$_2$SO	2469093	100	*P*2$_1$/*c* (14)	0.1170; 0.1628
(*n*-Bu$_4$N)$_3$[Ag(η^5-B$_{11}$H$_{11}$)$_2$]·0.5H$_2$O·0.5Me$_2$SO	2469092	200	*Pbcn* (60)	0.0786; 0.1017
$Na_3[Au(B_{11}H_{11})_2]\cdot8H_2O$	2421097	150	*I*2/*m* (12)	0.0282; 0.0379
$Na_3[Au(B_{11}H_{11})_2]\cdot8H_2O$	2421093	295	*I*2/*m* (12)	0.0221; 0.0271
$K_3[Au(B_{11}H_{11})_2]\cdot2H_2O$	2421099	100	*Pnnm* (58)	0.0128; 0.0172
$K_3[Au(B_{11}H_{11})_2]\cdot2H_2O$	2421096	150	*Pnnm* (58)	0.0136; 0.0195
$K_3[Au(B_{11}H_{11})_2]\cdot2H_2O$	2421094	200	*Pnnm* (58)	0.0131; 0.0205
$K_3[Au(B_{11}H_{11})_2]\cdot3CH_3CN$	2454492	100	*P*−1 (2)	0.0169; 0.0179
$BPy_4[Au(B_{11}H_{11})_2]$	2421098	150	*C*2/*c* (15)	0.0192; 0.0366
(*n*-Bu$_4$N)$_3$[Au(η^5-B$_{11}$H$_{11}$)$_2$]	2455740	100	*P*−1 (2)	0.0302; 0.0364
$K_3[Au(B_{11}H_{11-x}F_x)_2]\cdot4H_2O$; x = 4.12	2421095	150	*P*2$_1$/*n* (14)	0.0298; 0.0489
$Na_2[B_{11}H_{11}]\cdot5C_4H_8O_2\cdot2MeCN\cdot2H_2O$	2421101	150	*Pbcn* (60)	0.0593; 0.0921
$Na_2[B_{11}H_{11}]\cdot3C_4H_8O_2$	2421100	150	*P*−1 (2)	0.0505; 0.0933
$K_2[B_{11}H_{11}]\cdot H_2O$	2421102	150	*P*−1 (2)	0.0335; 0.0439
BPy_4I_3	2432369	150	*Pccn* (56)	0.0221; 0.0268
$BPy_4I_3\cdot H_2O$	2432365	150	*P*2$_1$/*c* (14)	0.0698; 0.0926
$BPy_4I_3\cdot2H_2O$	2432363	150	*Pbcn* (60)	0.1152; 0.2176
$BPy_4(NO_3)_3$	2432366	150	*P*2$_1$2$_1$2$_1$ (19)	0.0511; 0.0985
$BPy_4(BF_4)_3$	2432364	150	*P*2$_1$2$_1$2$_1$ (19)	0.0529; 0.0756
$BPy_4(PF_6)_3$	2432368	150	*P*2$_1$/*c* (14)	0.0420; 0.0539
$BH_2Py_2I_3$	2456440	150	*P*2/*n* (13)	0.0249; 0.0331
p-(C$_5$H$_5$N)C$_5$H$_4$NHI$_2$·H$_2$O	2432367	150	*P*2$_1$/*c* (14)	0.0282; 0.0383

a) First value is for reflections with $F^2>2\sigma(F^2)$, second value is for all reflections.

Table S1. Summarized crystallographic data (Continuation).

Compound	$Na_3[Cu(B_{11}H_{11})_2]$ ·$8H_2O$	$Na_3[Cu(B_{11}H_{11})_2]$ ·$8H_2O$	$K_3[Cu(B_{11}H_{11})_2]$ ·$5H_2O$	$K_3[Cu(B_{11}H_{11})_2]$ ·$2H_2O$
Measurement	exp_4190	exp_310	exp_4238	exp_4239
Radiation	Mo*Kα*, λ = 0.71073 Å			
Formula	$B_{22}CuH_{38}Na_3O_8$	$B_{22}CuH_{38}Na_3O_8$	$B_{22}CuH_{32}K_3O_5$	$B_{22}CuH_{26}K_3O_2$
Formula weight	536.63	536.63	530.91	476.87
Crystal system	monoclinic	monoclinic	triclinic	orthorhombic
Space group (Nr.)	*I*2/*m* (12)	*I*2/*m* (12)	*P*−1 (2)	*Pnnm* (58)
Color of crystal	orange	orange	orange	orange
a, Å	15.8892(2)	7.8759(6)	7.69546(16)	11.83564(13)
b, Å	9.03333(12)	9.0841(8)	13.0178(2)	9.90672(12)
c, Å	19.0546(3)	18.3502(14)	13.2166(3)	8.86166(11)
α, °	90	90	111.0906(18)	90
β, °	111.7158(18)	90.497(7)	95.3589(17)	90
γ, °	90	90	101.4672(16)	90
V, Å^3	2540.86(7)	1312.82(18)	1190.80(4)	1039.05(2)
Z	4	2	2	2
T, K	150	200	100	100
Unique reflections all / $F^2>2\sigma(F^2)$ / $d > d_{full}$	6969 / 6143 / 6622	2505 / 2306 / 1783	12216 / 10492 / 11593	2858 / 2541 / 2739
d_{full}, Å	0.595	0.744	0.599	0.593
Completeness for $d > d_{full}$	0.9989	1.0000	0.9997	1.0000
R_1 ($F^2>2\sigma(F^2)$)	0.0250	0.0379	0.0319	0.0278
R_1 (all)	0.0321	0.0439	0.0399	0.0333
GOF, ref	1.059	1.115	1.036	1.074
CCDC Number	2421085	2449213	2421080	2421088

Table S1. Summarized crystallographic data (Continuation).

Compound	$K_3[Cu(B_{11}H_{11})_2]$ ·$3.5H_2O$·0.5KOH	$(NH_4)_3[Cu(B_{11}H_{11})_2]$ ·$3NH_4Cl$	$BPy_4[Cu(B_{11}H_{11})_2]$	$BPy_4[Cu(B_{11}H_{11})_2]$ ·$5Me_2SO$
Measurement	TaKn170_a	exp_3823	exp_4507	exp_4505, twin
Radiation	Mo*Kα*, λ = 0.71073 Å			
Formula	$B_{22}CuH_{29.56}K_{3.56}O_4$	$B_{22}H_{46}Cl_3CuN_6$	$C_{20}H_{42}B_{23}CuN_4$	$C_{30}H_{72}B_{23}CuN_4O_5S_5$
Formula weight	534.17	538.14	650.74	1041.38
Crystal system	monoclinic	monoclinic	monoclinic	monoclinic
Space group (Nr.)	*P*2$_1$/*c* (14)	*I*2/*m* (12)	*C*2/*c* (15)	*P*2$_1$/*m* (11)
Color of crystal	yellow	orange	orange	orange
a, Å	7.6541(2)	12.85258(13)	18.3496(6)	14.1352(11)
b, Å	21.6140(5)	9.86884(10)	9.7605(2)	20.1554(13)
c, Å	14.8744(4)	10.61763(9)	19.7997(6)	19.6932(14)
α, °	90	90	90	90
β, °	104.455(2)	93.2985(8)	105.707(3)	107.396(8)
γ, °	90	90	90	90
V, Å^3	2382.86(11)	1344.51(2)	3413.72(17)	5354.0(7)
Z	4	2	4	4
T, K	100	150	150	150
Unique reflections all / $F^2>2\sigma(F^2)$ / $d > d_{full}$	6575 / 5001 / 4890	5687 / 5578 / 5664	3989 / 3066 / 3508	41923 (13689) / 25478 / (11298)
d_{full}, Å	0.799	0.506	0.798	0.799
Completeness for $d > d_{full}$	1.000	0.9993	0.9991	0.9990
R_1 ($F^2>2\sigma(F^2)$)	0.0346	0.0214	0.0408	0.0565
R_1 (all)	0.0498	0.0219	0.0636	0.0969
GOF, ref	1.073	1.083	1.029	0.919
CCDC Number	2454502	2421078	2421077	2421089

Table S1. Summarized crystallographic data (Continuation).

Compound	$(n\text{-}Bu_4N)_3$ $[Cu(B_{11}H_{11})_2]$	$Na_3[Cu(B_{11}H_{11-x}(OH)_x)_2]$ $\cdot 8H_2O$; x = 0.19	$K_3[Cu(B_{11}H_{11-x}(OH)_x)_2]$ $\cdot 5H_2O$; x = 0.14	$[Co(NH_3)_6]$ $[Cu(B_{11}H_{11-x}(OH)_x)_2]$ $\cdot 2H_2O$ x = 0.15
Measurement	TaKn199	exp_4180	exp_4580	exp_4568
Radiation	Cu*Kα*, λ = 1.54184 Å	Mo*Kα*, λ = 0.71073 Å		
Formula	$C_{48}H_{130}B_{22}CuN_3$	$B_{22}CuH_{38}Na_3O_{8.37}$	$B_{22}CuH_{32}K_3O_{5.27}$	$B_{22}CoCuH_{44}N_6O_{2.29}$
Formula weight	1050.90	542.55	535.19	525.18
Crystal system	triclinic	monoclinic	monoclinic	triclinic
Space group (Nr.)	*P*−1 (2)	*I*2/*m* (12)	*P*2₁/*c* (14)	*P*−1 (2)
Color of crystal	light orange	orange	orange	orange
a, Å	11.55610(10)	8.10250(10)	7.69249(9)	7.7130(4)
b, Å	13.0194(2)	9.01082(11)	21.6185(2)	9.4178(6)
c, Å	23.0248(3)	17.2487(2)	14.93201(19)	10.1186(6)
α, °	101.8560(10)	90	90	116.935(6)
β, °	99.5770(10)	95.4604(11)	104.6622(12)	111.545(5)
γ, °	91.4610(10)	90	90	90.344(5)
V, Å^3	3336.61(7)	1253.62(3)	2402.32(5)	596.18(7)
Z	2	2	4	1
T, K	100	150	150	150
Unique reflections all / $F^2>2\sigma(F^2)$ / $d > d_{full}$	12749 / 10321 / 12641	3403 / 3356 / 3239	8413 / 7012 / 7474	4928 / 4007 / 4531
d_{full}, Å	0.815	0.597	0.695	0.651
Completeness for $d > d_{full}$	0.981	1.0000	0.9995	1.0000
R_1 ($F^2>2\sigma(F^2)$)	0.0572	0.0188	0.0237	0.0269
R_1 (all)	0.0729	0.0192	0.0323	0.0381
GOF, ref	1.021	1.057	1.027	1.046
CCDC Number	2454491	2421086	2421083	2421081

Table S1. Summarized crystallographic data (Continuation).

Compound	$(Et_3NH)_3$ $[Cu(B_{11}H_{11-x}(OH)_x)_2]$ $\cdot 0.88H_2O$; x = 0.11	$(Et_3NH)_3$ $[Cu(B_{11}H_{11-x}(OH)_x)_2]$ $\cdot 0.95CuCl$; x = 0.25	$(Et_3NH)_2$ $[Cu(B_{11}H_{10})_2(OCHMe_2)]$ $\cdot H_2O$	$Cs_3[Cu(B_{11}H_{11-x}F_x)_2]$ x = 4.26
Measurement	exp_4181, twin	exp_4173	exp_4184	exp_4571 [b)]
Radiation	Mo*Kα*, λ = 0.71073 Å			
Formula	$C_{18}H_{71.75}B_{22}CuN_3O_{1.10}$	$C_{18}H_{70}B_{22}Cl_{0.95}Cu_{1.95}N_3O_{0.50}$	$C_{15}H_{61}B_{22}CuN_2O_2$	$B_{22}Cs_3CuF_{8.51}H_{13.49}$
Formula weight	649.49	732.17	603.01	875.29
Crystal system	triclinic	triclinic	monoclinic	orthorhombic
Space group (Nr.)	*P*−1 (2)	*P*−1 (2)	$P2_1/c$ (14)	*Cmca* (64)
Color of crystal	orange	orange	orange	orange
a, Å	9.0270(7)	11.2710(2)	11.4016(2)	10.80533(19)
b, Å	10.2353(8)	12.2575(3)	15.3185(2)	16.2184(3)
c, Å	11.6171(8)	14.5636(4)	20.6301(4)	14.0972(4)
α, °	64.703(7)	85.568(2)	90	90
β, °	83.961(6)	80.830(2)	104.1014(17)	90
γ, °	89.201(6)	81.468(2)	90	90
V, Å^3	964.46(14)	1961.38(9)	3494.58(10)	2470.46(9)
Z	1	2	4	4
T, K	150	150	150	150
Unique reflections all / $F^2>2\sigma(F^2)$ / $d > d_{full}$	21177 (5755) / 15709 / (4851)	9686 / 7792 / 8128	12384 / 10551 / 11198	2318 / 1847 / 2180
d_{full}, Å	0.746	0.796	0.689	0.677
Completeness for $d > d_{full}$	0.9992	0.9993	0.9994	1.0000
R_1 ($F^2>2\sigma(F^2)$)	0.0480	0.0319	0.0346	0.0257
R_1 (all)	0.0698	0.0474	0.0443	0.0376
GOF, ref	0.948	1.027	1.021	1.129
CCDC Number	2421087	2421084	2421082	2421079

Table S1. Summarized crystallographic data (Continuation).

Compound	$K_3[Ag(B_{11}H_{11})_2]\cdot 2H_2O$	$K_3[Ag(B_{11}H_{11})_2]\cdot 2H_2O$	$K_3[Ag(B_{11}H_{11})_2]\cdot 2H_2O$	$K_3[Ag(B_{11}H_{11})_2]\cdot 5H_2O$
Measurement	exp_4237	TaKn239_b	exp_3852	exp_4243, twin
Radiation	Mo*Kα*, λ = 0.71073 Å			
Formula	$AgB_{22}H_{26}K_3O_2$	$AgB_{22}H_{26}K_3O_2$	$AgB_{22}H_{26}K_3O_2$	$AgB_{22}H_{32}K_3O_5$
Formula weight	521.20	521.20	521.20	575.24
Crystal system	orthorhombic	orthorhombic	orthorhombic	triclinic
Space group (Nr.)	*Pnnm* (58)	*Pnnm* (58)	*Pnnm* (58)	*P*−1 (2)
Color of crystal	orange	orange	orange	orange
a, Å	12.03397(11)	12.0340(2)	12.04197(18)	7.71101(19)
b, Å	9.89649(9)	9.90130(10)	9.89648(14)	13.2516(3)
c, Å	8.90077(8)	8.91460(10)	8.92265(13)	13.4332(4)
α, °	90	90	90	112.368(2)
β, °	90	90	90	94.781(2)
γ, °	90	90	90	102.042(2)
V, Å^3	1060.029(17)	1062.19(2)	1063.34(3)	1221.17(5)
Z	2	2	2	2
T, K	100	100	150	100
Unique reflections all / $F^2>2\sigma(F^2)$ / $d > d_{full}$	2917 / 2609 / 2803	1718 / 1578 / 1170	2920 / 2623 / 2810	44912 (12378) / 37992 / (11850)
d_{full}, Å	0.593	0.799	0.593	0.600
Completeness for $d > d_{full}$	1.0000	1.000	0.9989	0.9988
R_1 ($F^2>2\sigma(F^2)$)	0.0139	0.0193	0.0145	0.0291
R_1 (all)	0.0179	0.0220	0.0180	0.0372
GOF, ref	1.047	1.063	1.089	0.939
CCDC Number	2421091	2454493	2421090	2421092

Table S1. Summarized crystallographic data (Continuation).

Compound	(*n*-Bu$_4$N)$_3$ [Ag(η^2-$B_{11}H_{11}$)$_2$]	(*n*-Bu$_4$N)$_3$ [Ag(η^5-$B_{11}H_{11}$)$_2$]	(*n*-Bu$_4$N)$_3$ [Ag(η^5-$B_{11}H_{11}$)$_2$] $\cdot 0.5H_2O\cdot 0.5Me_2SO$	(*n*-Bu$_4$N)$_3$ [Ag(η^5-$B_{11}H_{11}$)$_2$] $\cdot 0.5H_2O\cdot 0.5Me_2SO$
Measurement	TaKn173_a	TaKn241_4_173K_a	exp_4595, twin	exp_280
Radiation	Mo*Kα*, λ = 0.71073 Å	Cu*Kα*, λ = 1.54184 Å	Mo*Kα*, λ = 0.71073 Å	
Formula	$C_{48}H_{130}AgB_{22}N_3$	$C_{48}H_{130}AgB_{22}N_3$	$C_{49}H_{134}AgB_{22}N_3OS_{0.5}$	$C_{49}H_{134}AgB_{22}N_3OS_{0.5}$
Formula weight	1095.23	1095.23	1143.30	1143.79
Crystal system	triclinic	triclinic	monoclinic	orthorhombic
Space group (Nr.)	*P*−1 (2)	*P*−1 (2)	*P*2$_1$/*c* (14)	*Pbcn* (60)
Color of crystal	orange	orange	orange	orange
a, Å	11.0102(3)	11.4445(1)	23.0563(6)	44.5102(6)
b, Å	13.2574(3)	12.8636(1)	44.0609(14)	13.7434(2)
c, Å	24.1778(5)	23.9836(2)	13.6457(3)	23.2781(3)
α, °	76.801(2)	103.804(1)	90	90
β, °	80.194(2)	96.252(1)	90.765(2)	90
γ, °	82.275(2)	94.630(1)	90	90
V, Å^3	3368.90(14)	3387.55(5)	13861.1(7)	14239.7(4)
Z	2	2	8	8
T, K	100	173	100	200
Unique reflections all / $F^2>2\sigma(F^2)$ / $d > d_{full}$	17523 / 12665 / 13798	13884 / 13198 / 13627	276414 (36622) / 170726 / (28432)	23699 / 18056 / 21737
d_{full}, Å	0.799	0.800	0.799	0.700
Completeness for $d > d_{full}$	0.999	0.983	0.9998	1.0000
R_1 ($F^2>2\sigma(F^2)$)	0.0600	0.0342	0.1170	0.0786
R_1 (all)	0.0934	0.0356	0.1628	0.1017
GOF, ref	1.024	1.094	2.588	1.217
CCDC Number	2454494	2454490	2469093	2469092

Table S1. Summarized crystallographic data (Continuation).

Compound	$Na_3[Au(B_{11}H_{11})_2]$ $\cdot 8H_2O$	$Na_3[Au(B_{11}H_{11})_2]$ $\cdot 8H_2O$	$K_3[Au(B_{11}H_{11})_2]$ $\cdot 2H_2O$	$K_3[Au(B_{11}H_{11})_2]$ $\cdot 2H_2O$
Measurement	exp_4434	exp_4351	exp_4367	exp_4366
Radiation	Mo*Kα*, λ = 0.71073 Å			
Formula	$AuB_{22}H_{38}Na_3O_8$	$AuB_{22}H_{38}Na_3O_8$	$AuB_{22}H_{26}K_3O_2$	$AuB_{22}H_{26}K_3O_2$
Formula weight	670.06	670.06	610.29	610.29
Crystal system	monoclinic	monoclinic	orthorhombic	orthorhombic
Space group (Nr.)	*I*2/*m* (12)	*I*2/*m* (12)	*Pnnm* (58)	*Pnnm* (58)
Color of crystal	yellow	yellow	yellow	yellow
a, Å	15.9758(3)	16.0958(4)	12.00315(12)	12.01281(19)
b, Å	9.05385(15)	9.1128(2)	9.89879(10)	9.90092(16)
c, Å	19.2531(5)	19.4654(6)	8.91459(9)	8.94017(15)
α, °	90	90	90	90
β, °	110.507(3)	110.563(3)	90	90
γ, °	90	90	90	90
V, Å^3	2608.34(10)	2673.24(13)	1059.203(18)	1063.32(3)
Z	4	4	2	2
T, K	150	295	100	150
Unique reflections all / $F^2>2\sigma(F^2)$ / $d > d_{full}$	4825 / 4209 / 4216	3548 / 3204 / 2915	2914 / 2508 / 2759	2925 / 2415 / 2768
d_{full}, Å	0.700	0.799	0.595	0.595
Completeness for $d > d_{full}$	0.9993	0.9993	0.9996	0.9996
R_1 ($F^2>2\sigma(F^2)$)	0.0282	0.0221	0.0128	0.0136
R_1 (all)	0.0379	0.0271	0.0172	0.0195
GOF, ref	1.031	1.076	1.083	1.041
CCDC Number	2421097	2421093	2421099	2421096

Table S1. Summarized crystallographic data (Continuation).

Compound	$K_3[Au(B_{11}H_{11})_2]$ $\cdot 2H_2O$	$K_3[Au(B_{11}H_{11})_2]$ $\cdot 3CH_3CN$	$BPy_4[Au(B_{11}H_{11})_2]$	$(n\text{-}Bu_4N)_3$ $[Au(\eta^5\text{-}B_{11}H_{11})_2]$
Measurement	exp_4368	TaKn215_4_a	exp_4553	TaKn244_3_a
Radiation	Mo*Kα*, λ = 0.71073 Å	Cu*Kα*, λ = 1.54184 Å	Mo*Kα*, λ = 0.71073 Å	
Formula	$AuB_{22}H_{26}K_3O_2$	$C_6H_{31}AuB_{22}K_3N_3$	$C_{20}H_{42}AuB_{23}N_4$	$C_{48}H_{130}AuB_{22}N_3$
Formula weight	610.29	697.42	784.17	1184.33
Crystal system	orthorhombic	triclinic	monoclinic	triclinic
Space group (Nr.)	*Pnnm* (58)	*P*−1 (2)	*C*2/*c* (15)	*P*−1 (2)
Color of crystal	yellow	colourless	yellow	colourless
a, Å	12.07008(13)	9.9279(2)	18.4363(3)	11.4487(1)
b, Å	9.93456(11)	10.3095(2)	9.76945(12)	12.7763(1)
c, Å	8.99829(10)	15.3186(2)	20.0741(3)	23.8347(2)
α, °	90	89.8630(10)	90	103.489(1)
β, °	90	73.6530(10)	106.6044(15)	96.512(1)
γ, °	90	67.750(2)	90	94.527(1)
V, Å^3	1078.99(2)	1382.86(5)	3464.82(8)	3348.21(5)
Z	2	2	4	2
T, K	200	100	150	100
Unique reflections all / $F^2>2\sigma(F^2)$ / $d > d_{full}$	2935 / 2324 / 2816	5484 / 5185 /5299	4570 / 3309 / 3942	16354 / 14663 / 13729
d_{full}, Å	0.595	0.815	0.772	0.799
Completeness for $d > d_{full}$	0.9989	0.989	0.9997	1.000
R_1 ($F^2>2\sigma(F^2)$)	0.0131	0.0169	0.0192	0.0302
R_1 (all)	0.0205	0.0179	0.0366	0.0364
GOF, ref	1.044	1.057	1.084	1.063
CCDC Number	2421094	2454492	2421098	2455740

Table S1. Summarized crystallographic data (Continuation).

Compound	$K_3[Au(B_{11}H_{11-x}F_x)_2]\cdot 4H_2O$; x = 4.12	$Na_2[B_{11}H_{11}]\cdot 5C_4H_8O_2\cdot 2MeCN\cdot 2H_2O$	$Na_2[B_{11}H_{11}]\cdot 3C_4H_8O_2$	$K_2[B_{11}H_{11}]\cdot H_2O$
Measurement	exp_4464 [c)]	exp_4340	exp_4349	exp_4577
Radiation	Mo*Kα*, *λ* = 0.71073 Å			
Formula	$AuB_{22}F_{8.24}H_{21.76}K_3O_4$	$C_{24}H_{61}B_{11}N_2Na_2O_{12}$	$C_{12}H_{35}B_{11}Na_2O_6$	$B_{11}H_{13}K_2O$
Formula weight	794.58	734.63	440.29	226.21
Crystal system	monoclinic	orthorhombic	triclinic	triclinic
Space group (Nr.)	$P2_1/n$ (14)	*Pbcn* (60)	*P*−1 (2)	*P*−1 (2)
Color of crystal	yellow	colorless	colorless	colorless
a, Å	10.7540(2)	18.2155(9)	7.3504(7)	11.7898(4)
b, Å	10.81807(15)	14.6541(7)	9.0848(9)	13.7279(4)
c, Å	11.25601(18)	14.9237(5)	17.6859(17)	14.2989(4)
α, °	90	90	98.420(8)	107.302(3)
β, °	104.8554(18)	90	93.534(8)	90.538(3)
γ, °	90	90	98.850(8)	90.524(3)
V, Å^3	1265.73(4)	3983.6(3)	1150.1(2)	2209.29(12)
Z	2	4	2	8
T, K	150	150	150	150
Unique reflections all / $F^2>2\sigma(F^2)$ / $d > d_{full}$	3777 / 2798 / 3140	4815 / 3276 / 4088	5283 / 3527 / 4717	14991 / 12624 / 13480
d_{full}, Å	0.750	0.799	0.799	0.700
Completeness for $d > d_{full}$	1.0000	0.9978	0.9989	0.9992
R_1 ($F^2>2\sigma(F^2)$)	0.0298	0.0593	0.0505	0.0335
R_1 (all)	0.0489	0.0921	0.0933	0.0439
GOF, ref	1.126	1.042	1.002	1.063
CCDC Number	2421095	2421101	2421100	2421102

Table S1. Summarized crystallographic data (Continuation).

Compound	BPy_4I_3	$BPy_4I_3\cdot H_2O$	$BPy_4I_3\cdot 2H_2O$	$BPy_4(NO_3)_3$
Measurement	exp_4548	exp_4554, twin	exp_4504	exp_4509
Radiation	Mo*Kα*, *λ* = 0.71073 Å			
Formula	$C_{20}H_{20}BI_3N_4$	$C_{20}H_{22}BI_3N_4O$	$C_{20}H_{24}BI_3N_4O_2$	$C_{20}H_{20}BN_7O_9$
Formula weight	707.91	725.92	743.94	513.24
Crystal system	orthorhombic	monoclinic	orthorhombic	orthorhombic
Space group (Nr.)	*Pccn* (56)	$P2_1/c$ (14)	*Pbcn* (60)	$P2_12_12_1$ (19)
Color of crystal	yellow	yellow	yellow	colorless
a, Å	9.27921(11)	8.0643(5)	17.155(2)	8.1727(5)
b, Å	17.2069(2)	17.3017(11)	18.497(5)	16.3335(15)
c, Å	15.3974(2)	17.5113(19)	7.8516(12)	16.7403(10)
α, °	90	90	90	90
β, °	90	94.045(6)	90	90
γ, °	90	90	90	90
V, Å^3	2458.46(6)	2437.2(3)	2491.5(8)	2234.6(3)
Z	4	4	4	4
T, K	150	150	150	150
Unique reflections all / $F^2>2\sigma(F^2)$ / $d > d_{full}$	5286 / 4724 / 4858	11800 (5718) / 8802 / (4985)	3082 / 1316 / 2558	5263 / 3454 / 4576
d_{full}, Å	0.642	0.799	0.799	0.799
Completeness for $d > d_{full}$	1.0000	0.9990	0.9996	0.9996
R_1 ($F^2>2\sigma(F^2)$)	0.0221	0.0698	0.1152	0.0511
R_1 (all)	0.0268	0.0926	0.2176	0.0529
GOF, ref	1.102	1.041	1.516	0.970
CCDC Number	2432369	2432365	2432363	2432366

Table S1. Summarized crystallographic data (Continuation).

Compound	$BPy_4(BF_4)_3$	$BPy_4(PF_6)_3$	$BH_2Py_2I_3$	*p*-$(C_5H_5N)C_5H_4NHI_2\cdot H_2O$
Measurement	exp_4523	exp_4543	exp_4503	exp_4494
Radiation	Mo*Kα*, λ = 0.71073 Å			
Formula	$C_{20}H_{20}B_4F_{12}N_4$	$C_{20}H_{20}BF_{18}N_4P_3$	$C_{10}H_{12}BI_3N_2$	$C_{10}H_{12}I_2N_2O$
Formula weight	587.64	762.12	551.73	430.02
Crystal system	orthorhombic	monoclinic	monoclinic	monoclinic
Space group (Nr.)	$P2_12_12_1$ (19)	$P2_1/c$ (14)	$P2/n$ (13)	$P2_1/c$ (14)
Color of crystal	colorless	colorless	brown	yellow
a, Å	8.0678(2)	17.2961(4)	10.3719(2)	13.6117(6)
b, Å	17.0787(5)	9.37708(16)	4.45412(10)	7.3213(2)
c, Å	17.1593(6)	17.5709(3)	16.6727(4)	13.7900(5)
α, °	90	90	90	90
β, °	90	107.928(2)	95.771(2)	98.516(4)
γ, °	90	90	90	90
V, Å^3	2364.35(13)	2711.40(9)	766.34(3)	1359.10(9)
Z	4	4	2	4
T, K	150	150	150	150
Unique reflections all / $F^2>2\sigma(F^2)$ / $d > d_{full}$	5465 / 4220 / 4842	7121 / 5898 / 5940	2702 / 2326 / 2406	3166 / 2672 / 2833
d_{full}, Å	0.799	0.781	0.695	0.795
Completeness for $d > d_{full}$	0.9992	0.9997	1.0000	0.9989
R_1 ($F^2>2\sigma(F^2)$)	0.0529	0.0420	0.0249	0.0282
R_1 (all)	0.0756	0.0539	0.0331	0.0383
GOF, ref	1.037	1.020	1.076	1.081
CCDC Number	2432364	2432368	2456440	2432367

[b)] Occupation factors for fluorine atoms in $Cs_3[Cu(B_{11}H_{11-x}F_x)_2]$, x = 4.26; exp_4571 (CCDC 2421079):
F1 0.717(13)·c1; F2 0.820(10)·c1; F3 0.662(9)·c1; F7 0.465(11)·c1; F21 1.0·c2; F22 1.0·c2; F23 0.74(3)·c2; F27 0.74(3)·c2
Main component: c1 = 0,778(3) minor component: c2 = 0,222(3).

[c)] Occupation factors for fluorine atoms in $K_3[Au(B_{11}H_{11-x}F_x)_2]\cdot 4H_2O$, x = 4.12; exp_4464 (CCDC 2421095): F1 0.777(9); F2 0.438(9); F3 0.188(10); F4 0.951(8); F5 0.223(8); F6 0.531(10); F8 0.775(8); F11 0.236(9)

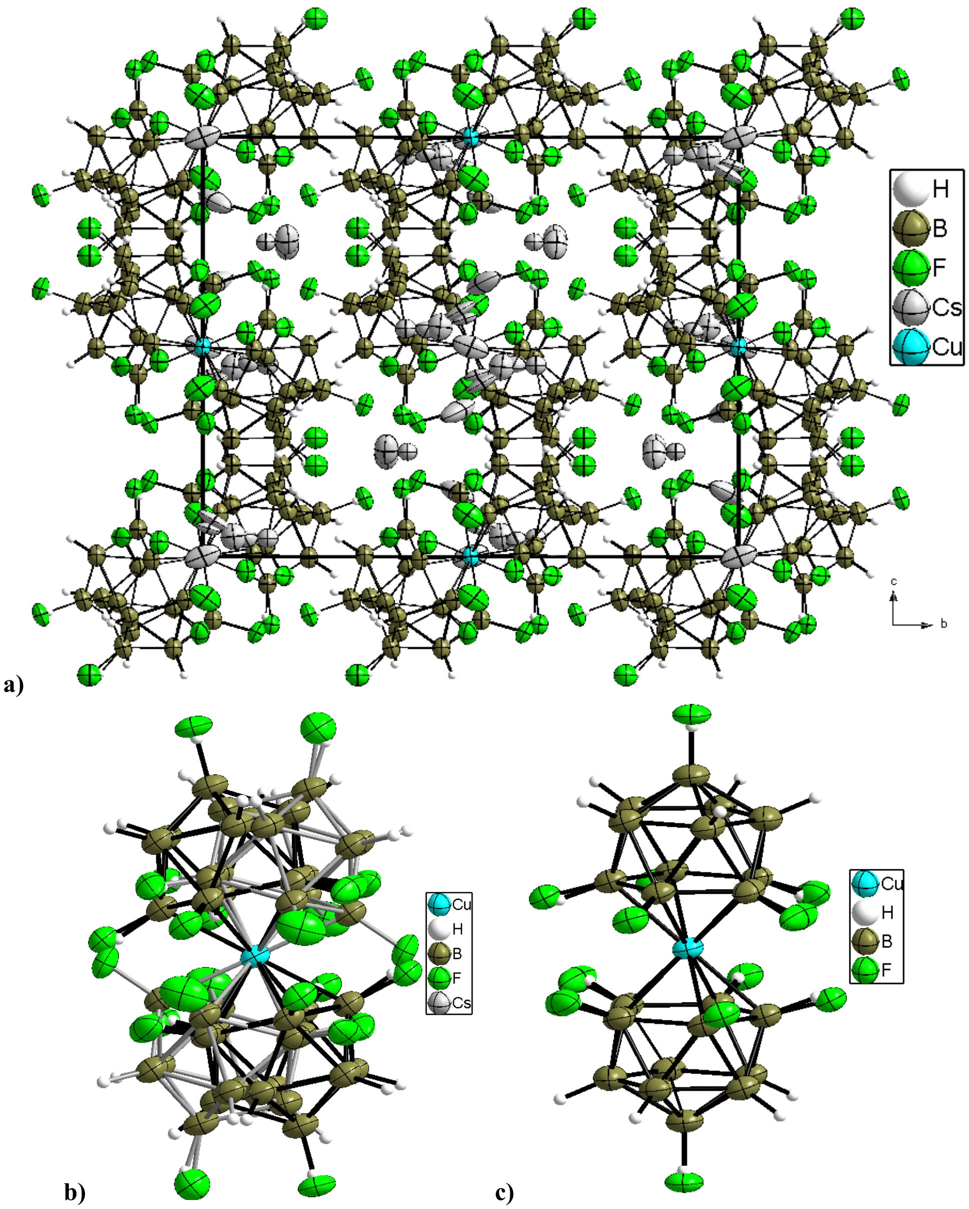

Figure S1. Structure of the cell (a), anions (b) and the main component of the anions (c) in $Cs_3[Cu(B_{11}H_{11-x}F_x)_2]$ (x = 4.26) at 150 K. Thermal ellipsoids are drawn at 50% probability. Occupation factors for fluorine atoms: F1 0.717(13)·c1; F2 0.820(10)·c1; F3 0.662(9)·c1; F7 0.465(11)·c1; F21 1.0·c2; F22 1.0·c2; F23 0.74(3)·c2; F27 0.74(3)·c2;
Main component: c1 = 0,778(3) minor component: c2 = 0,222(3).

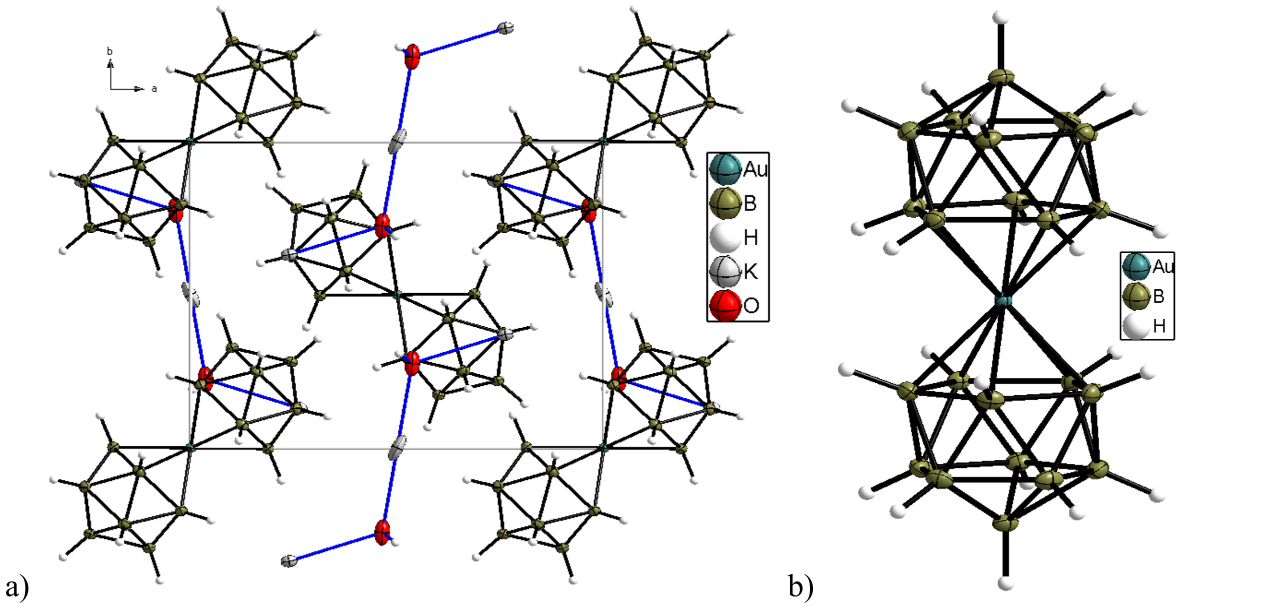

Figure S2. Structure of the cell (a) and anions (b) in $K_3[Au(B_{11}H_{11})_2]\cdot 2H_2O$ at 100 K. Thermal ellipsoids are drawn at 50% probability.

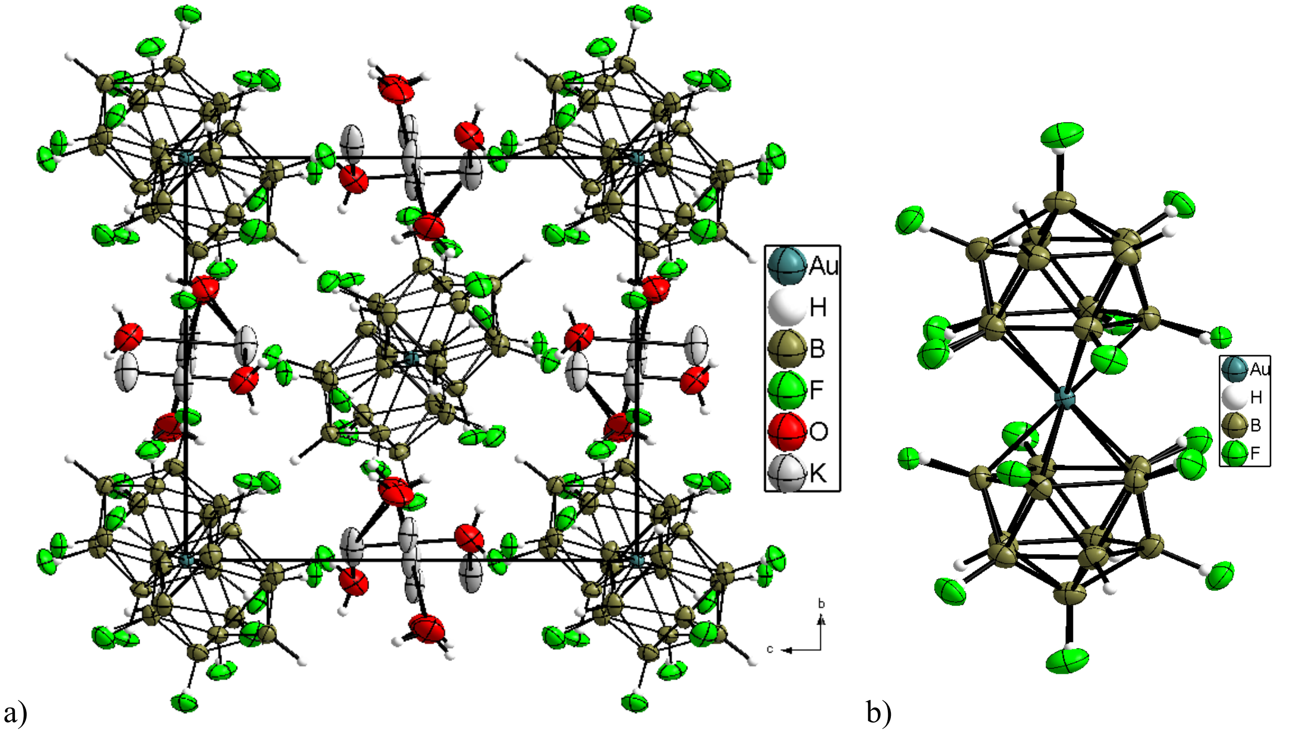

Figure S3. Structure of the cell (a) and anions (b) in $K_3[Au(B_{11}H_{11-x}F_x)_2]\cdot 4H_2O$ (x = 4.12) at 150 K. Thermal ellipsoids are drawn at 50% probability. Occupation factors for fluorine atoms: F1 0.777(9); F2 0.438(9); F3 0.188(10); F4 0.951(8); F5 0.223(8); F6 0.531(10); F8 0.775(8); F11 0.236(9)

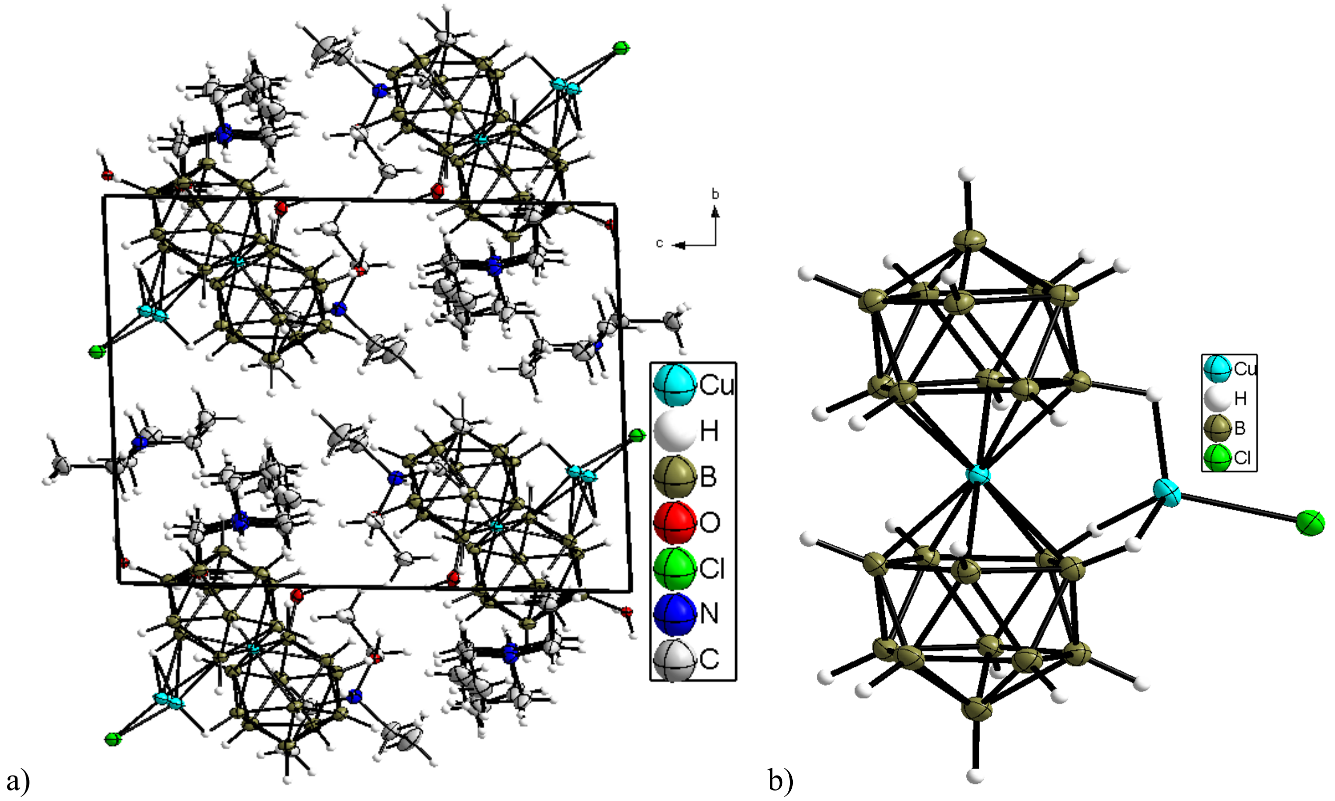

Figure S4. Structure of the cell (a) and anions (b) in $(Et_3NH)_3[Cu(B_{11}H_{11-x}(OH)_x)_2]\cdot 0.95CuCl$ (x = 0.25) at 150 K. The OH groups (sof = 0.296(5), 0.122(5), 0.046(3) and 0.031(3)) and minor positions of copper atoms (sof = 0.0185(16)) were not depicted in (b). Thermal ellipsoids are drawn at 50% probability.

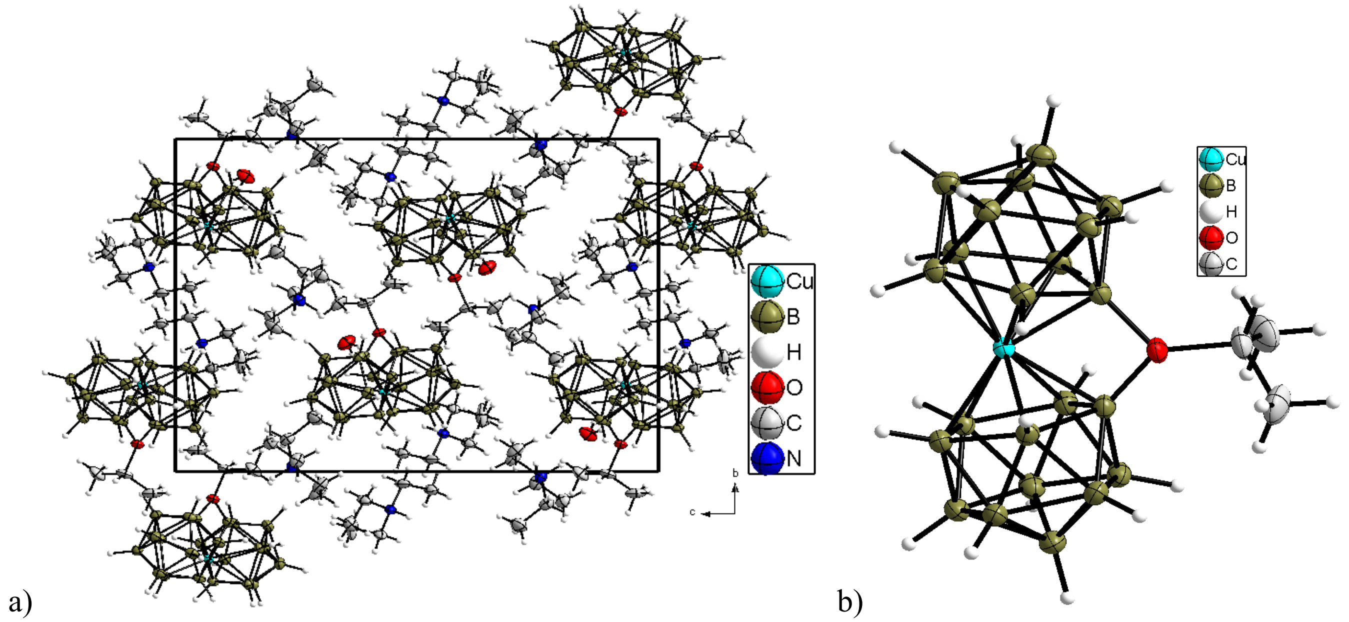

Figure S5. Structure of the cell (a) anions (b) in $(Et_3NH)_2[Cu(B_{11}H_{10})_2(OCHMe_2)]\cdot H_2O$ at 150 K. Thermal ellipsoids are drawn at 50% probability.

NMR spectroscopy

Nuclear magnetic resonance measurements were performed on either a BRUKER Avance 400 spectrometer with following probe heads: 5 mm broadband BBFO probe with automatic frequency tuning, and 5 mm broadband inverse probe or on a BRUKER Avance III 600 spectrometer with the probe heads 5 mm broadband inverse with automatic frequency tuning. Acetonitrile-d_3 and water-d_2 (Deutero) were used as solvents.

The ^{11}B and $^{11}B\{^1H\}$ NMR spectra of $[M(B_{11}H_{11})_2]^{3-}$ (M = Cu, Ag, Au) in CD_3CN are shown in Figure 4. The NMR data of $[M(B_{11}H_{11})_2]^{3-}$ (M = Cu, Ag, Au) are in Table S2. All further spectroscopic data (NMR and HRMS ESI) are provided in Tab. S2a-S4.

Table S2a. Resonance frequencies and standards of measured nuclei.

**Nuclei**	**Resonance frequencies [MHz]**		**Standard**
	BRUKER Avance 400	BRUKER Avance III 600	
^1H	400.13	600.27	$(CH_3)_4Si$
^{11}B	128.38	192.59	$BF_3 \cdot OEt_2$
^{13}C	100.63	150.95	$(CH_3)_4Si$
^{14}N	28.91	43.37	CH_3NO_2
^{15}N	40.55	60.84	CH_3NO_2

Table S2. NMR Data for $[M(B_{11}H_{11})_2]^{3-}$ in CD_3CN

Assignment	$\delta^{11}B$, ppm			$\delta^{1}H$, ppm			$^1J(^1H,^{11}B)$, Hz			$\sigma(^{11}B)$, Hz[a]		
	Cu	Ag	Au	Cu	Ag	Au	Cu	Ag	Au	Cu	Ag	Au
Solvent	CD_3CN											
para	22.8	17.2	13.0	3.71	3.76	4.71	126	131	125	92	100	100
ortho	13.7	11.9	3.7	2.83	2.84	2.28	137	133	135	153	120	130
meta	−6.9	−8.7	−13.0	1.72	1.81	1.95	130	130	128	58	50	50
Solvent	D_2O											
para	23.3	17.0	13.5	4.03	3.60	4.93	114	140	127	148	110	100
ortho	13.9	11.4	4.2	3.12	2.62	2.41	148	150	128	171	190	130
meta	−7.0	−9.3	−13.0	2.02	1.62	2.13	133	129	131	63	70	50

[a] half width

Table S3. NMR-Data for BPy_4I_3, *p*-$(C_5H_5N)C_5H_4NHI$ and $BH_2Py_2^+$.

	BPy_4I_3, D_2O (HCl, CD_3CN)	*p*-$(C_5H_5N)C_5H_4NHI$,	$BH_2Py_2I_3$, CD_3CN
δ(^{11}B), ppm	7.5		2.1
$^1J(^{11}B, ^1H)$, Hz			115(t, BH)
δ(^{1}H), ppm	8.34(m), 8.81(o, br), 8.95(p)	8.46(m2), 8.70(m1), 8.99(p2), 9.34(o1), 9.39(o2)	3.83(BH), 7.91(m), 8.39(p), 8.78(o)
$^3J(^1H, ^1H)$, Hz	7.0(t, m), 6(t, o), 7.8 (t, p)	7.0(d, m2), 7.8(d, m2), 7.2(d, m1), 7,9(t, p2), 7.1(d, o1), 6.9(d, o2)	112(q, BH), 7.2(t, m), 7.8(t, p), 5.9(d, o)
$^4J(^1H, ^1H)$, Hz		1.3(t, p2), 1.3(d, o2)	1.5(t, p)
$^1J(^1H, ^{13}C)$, Hz			173.2(d, m), 170.0(d, p), 187.0(d, o)
δ(^{13}C), ppm	131.0(br, o), 147.9(m), 151.5(p)		128.8(m), 145.0(p), 148,5(br, o)
$^1J(^{13}C, ^1H)$, Hz	178(d, o), 190(d, m), 174(d, o)		172.0(d, m), 169.9(d, p), 187.5(d, o)
$^{2,3}J(^{13}C, ^1H)$, Hz	6.3(q, m), 6.2(t, p)		6.0(t, m), 6.3(t, p), 6(q, o)
δ(^{14}N), ppm	-178		-151
δ(^{15}N), ppm	-179		

m - meta, o - ortho, p - para, d - duplet, t - triplet, q - quartet, br - broad,
m1 - meta, o1 - ortho for -C_5H_4NH, m2 - meta, o2 - ortho, p2 - para for C_5H_5N in *p*-$(C_5H_5N)C_5H_4NHI$.

NMR-Data for $(Et_3NH)_2[Cu(B_{11}H_{10})_2(OCHMe_2)]$

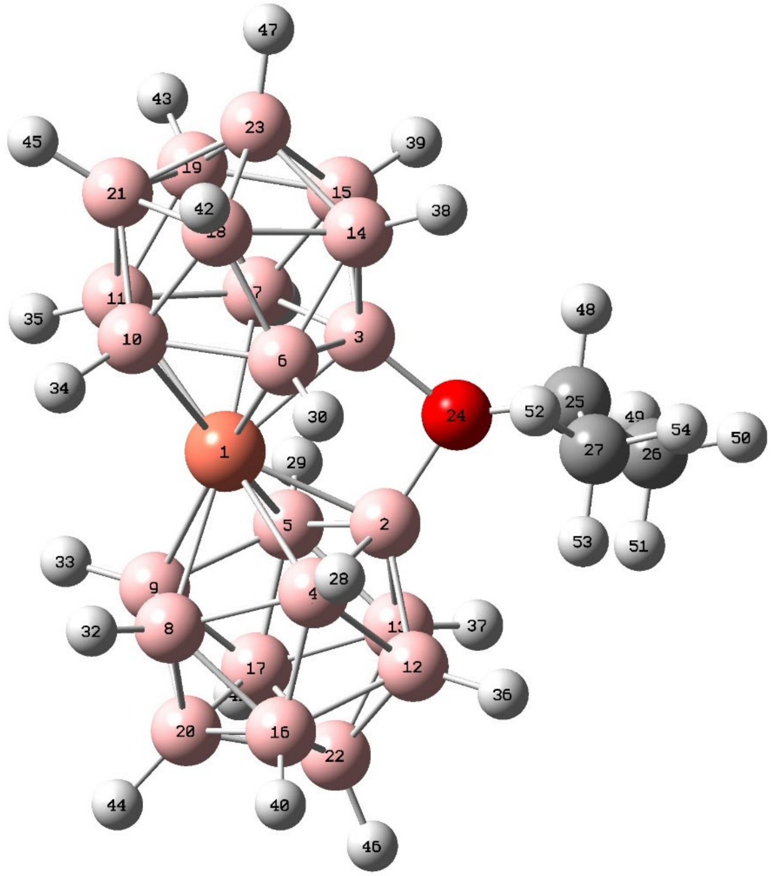

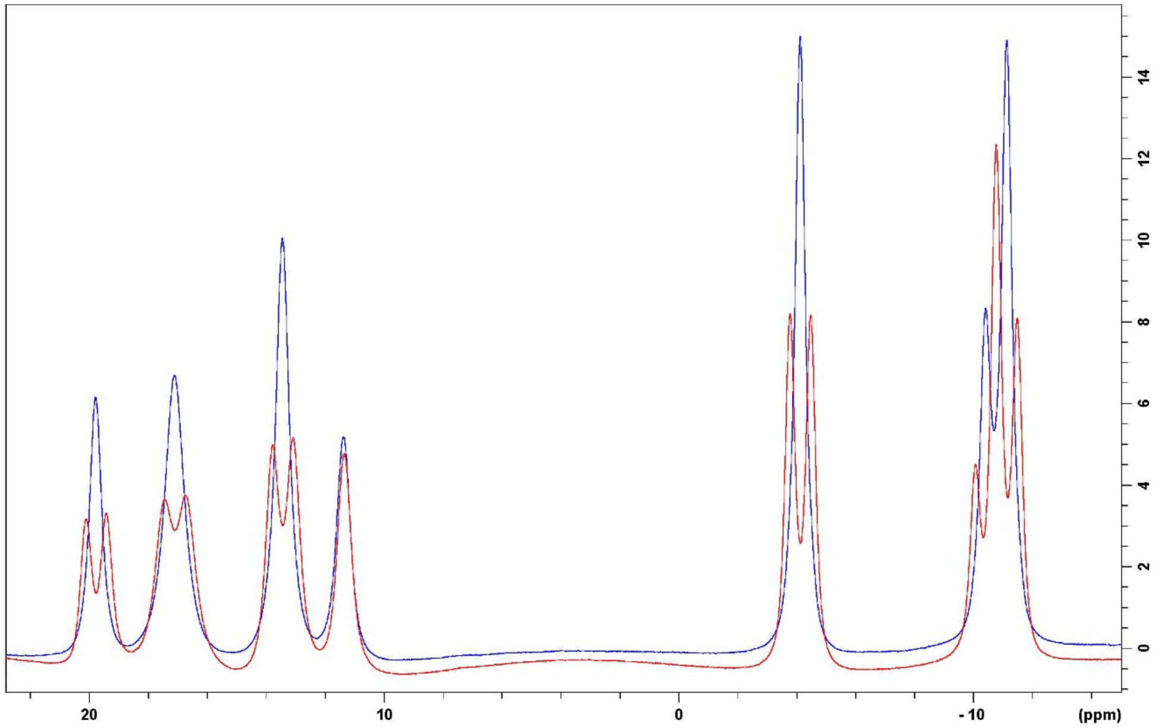

Figure S6. ^{11}B (red) and $^{11}B\{^1H\}$ (blue) spectra of $[Cu(B_{11}H_{10})_2(OCHMe_2)]^{2-}$ in Acetone-d^6

NMR-Data for $(Et_3NH)_2[Cu(B_{11}H_{10})_2(OCHMe_2)]$

Table S4. NMR-Data for $(Et_3NH)_2[Cu(B_{11}H_{10})_2(OCHMe_2)]$ in Acetone-d^6

Nr1	Nr2	Atom		σ_1, ppm	σ_2, ppm	δ_1, ppm	δ_2, ppm	δ_{av}, ppm	δ_{exp}, ppm	J_{exp}, Hz
1		Cu		1157.3996						
24		O		266.5010						
22	23	B	p	81.4091	81.3760	21.54	21.57	21.56	19.78	132, d
4	6	B	o	80.2642	80.6891	22.69	22.26	22.05	17.11	141, d
5	7	B	o	81.3727	81.2955	21.58	21.65			
8	10	B	o	85.3052	84.8112	17.64	18.14	17.40	13.44	136, d
9	11	B	o	86.2718	85.8093	16.68	17.14			
2	3	B	o	89.7249	89.8145	13.23	13.14	13.19	11.57	
16	18	B	m	106.4929	106.6782	-3.54	-3.73	-3.64	-4.13	135, d
17	19	B	m	106.4585	106.7422	-3.51	-3.79			
20	21	B	m	112.3722	112.0621	-9.42	-9.11	-9.27	-10.42	135, d
12	14	B	m	113.8443	114.7615	-10.89	-11.81	-11.14	-11.13	138, d
13	15	B	m	113.2026	114.5547	-10.25	-11.60			
28	30	H	o	27.1930	27.1777	4.76	4.77	4.68	4.41	(q)
29	31	H	o	27.3412	27.3581	4.61	4.59			
48		H	CH	27.5858		4.36		4.36	4.25	6.40, qi
46	47	H	p	28.3093	28.3360	3.64	3.61	3.63	3.64	(q)
32	34	H	o	28.7420	28.7015	3.21	3.25	3.18	2.93	(q)
33	35	H	o	28.8363	28.7925	3.11	3.16			
40	42	H	m	29.5329	29.5621	2.42	2.39	2.39	2.25	(q)
41	43	H	m	29.5670	29.5844	2.38	2.37			
36	38	H	m	29.7521	29.8582	2.20	2.09	2.10	2.01	(q)
37	39	H	m	29.8600	29.9232	2.09	2.03			
44	45	H	m	30.0393	30.0024	1.91	1.95	1.93	1.74	(q)
49	52	H	Me	30.5703	30.1841	1.38	1.77			
50	53	H	Me	30.9539	30.2365	1.00	1.71	1.40	1.45	6.40, d
51	54	H	Me	30.2660	31.0793	1.68	0.87			
25		C	CH	89.1236		95.47		95.47	86.71	151, d; 4.4 qi
26	27	C	Me	161.7922	163.1644	22.80	21.43	22.11	21.12	127, q; 4.0, d

GIAO//B3LYP/6-311++g(d,p), SCRF(Solvent=Acetone), referenziert auf $BF_3 \cdot OEt_2$ ($\delta(^{11}B) = 0$ ppm) $\delta(^{11}B) = 102.95 - \sigma(^{11}B)$ und Me_4Si ($\delta(^{13}C) = 0$ ppm, $\delta(^{1}H) = 0$ ppm) $\delta(^{13}C) = 184.59 - \sigma(^{13}C)$, $\delta(^{1}H) = 31.95 - \sigma(^{1}H)$; d dublett, q quartett, qi quintett. The assignment of terminal hydrogen atoms to the corresponding boron atoms was determined by ^{1}H-^{11}B COSY.

Atomic coordinates for $[Cu(B_{11}H_{10})_2(OCHMe_2)]^{2-}$

B3LYP/6-311++g(d,p), SCRF(Solvent=Acetone)

Cu	-0.1314633729	-0.9987056029	0.062354046
B	1.172949206	0.642308788	-0.1636247925
B	-1.0985703448	0.837729658	-0.1947169446
B	1.3522973245	-0.215761455	1.4899052678
B	1.3489811474	-0.6252786972	-1.5262086437
B	-1.4765706903	0.0481293433	1.4586523866
B	-1.4848235736	-0.369784473	-1.5628308773
B	1.5307547199	-1.9990130162	1.1091101883
B	1.5277053144	-2.2431638624	-0.6871941528
B	-1.9822577235	-1.6638956253	1.0734580798
B	-1.9852269196	-1.9126754739	-0.7257696463
B	2.6919199263	0.6523765325	0.7334255022
B	2.6916428113	0.4076575932	-1.0396450407
B	-2.6029317304	1.1543676001	0.6695381424
B	-2.6105299628	0.9070277837	-1.1046540815
B	2.9301307482	-0.96761743	1.4980058244
B	2.9273001582	-1.3565982557	-1.340750389
B	-3.169593325	-0.3837653301	1.428974038
B	-3.1779812727	-0.7791609225	-1.4110361579
B	3.0586470403	-2.1772438569	0.2168327235
B	-3.4949899768	-1.5519426283	0.1447149429
B	3.7791698178	-0.5528888674	-0.0068398964
B	-3.8869339964	0.1809990597	-0.0959734992
O	0.1158057892	1.7184900931	-0.3622605277
C	0.1485895322	3.1932878895	-0.136846327
C	1.1883854384	3.7997006778	-1.0603938938
C	0.3554997347	3.512402134	1.3336510357
H	0.9508288573	0.2137860305	2.5088688454
H	0.9398015225	-0.4775488917	-2.619325671
H	-1.0229680684	0.4017780248	2.4850321762
H	-1.0320541445	-0.3065650828	-2.6469481013
H	1.1634210769	-2.8314123538	1.8784255788
H	1.1571808082	-3.2506929391	-1.2045737972
H	-1.7979380667	-2.551867621	1.8466204794
H	-1.8027517125	-2.9774372565	-1.2287346467
H	3.1018683725	1.6534328277	1.2306668943
H	3.0901023087	1.2401406266	-1.7915190759
H	-2.8131157966	2.2231226679	1.1525574872
H	-2.8165357073	1.806253545	-1.8597298118
H	3.4983396905	-1.0652441437	2.5424921348
H	3.4904535802	-1.7318840181	-2.3234318449
H	-3.7711439728	-0.3669235164	2.4591651917
H	-3.782502206	-1.0453272714	-2.4045511025
H	3.7340436492	-3.1519604115	0.3503991856
H	-4.3454371944	-2.3807144902	0.2629114319
H	4.9583001808	-0.3753104529	-0.0305375091
H	-5.009433622	0.5805979933	-0.1470011332

H -0.8517599092 3.4951804332 -0.4500991031
H 0.9987498993 3.52372491 -2.0991060398
H 1.1376689373 4.8884261914 -0.9782547978
H 2.1965319344 3.483288242 -0.7877571628
H -0.3995921569 3.0259317424 1.9517904207
H 1.3461863569 3.2034383561 1.6701733722
H 0.2660235629 4.5935072023 1.4714402916

E(RB3LYP) = -2393.83086074
Zero-point correction= 0.387693 (Hartree/Particle)
Thermal correction to Energy= 0.410788
Thermal correction to Enthalpy= 0.411732
Thermal correction to Gibbs Free Energy= 0.341598
Sum of electronic and zero-point Energies= -2393.443167
Sum of electronic and thermal Energies= -2393.420072
Sum of electronic and thermal Enthalpies= -2393.419128
Sum of electronic and thermal Free Energies= -2393.489263

	E (Thermal)	CV	S
	KCal/Mol	Cal/Mol-Kelvin	Cal/Mol-Kelvin
Total	257.774	112.306	147.611

The following NMR and HRMS data were measured on other devices:
^{1}H, ^{13}C and ^{11}B NMR spectra were recorded at 25 °C in D_2O or CD_3CN on a *Bruker* Avance NEO 400 NMR spectrometer. The NMR signals were referenced against TMS (^{1}H and ^{13}C) and $BF_3{\cdot}OEt_2$ (^{11}B) with Ξ[^{13}C] = 25.1504 MHz and Ξ[^{11}B] = 32.0897 MHz.[21] ^{1}H and ^{13}C chemical shifts were calibrated against the residual solvent signal (CHD_2CN: *d*(^{1}H) = 1.94 ppm, HDO: *d*(^{1}H) = 4.79 ppm, CD_3CN *d*(^{13}C) = 1.32 and 118.26 ppm).[22]
HRMS ESI spectra were recorded using an Exactive Plus mass spectrometer with Orbitrap (*Thermo Scientific*) equipped with an ESI source (3.5 kV spray voltage). (HRMS = high resolution mass spectrometry, ESI = electrospray ionization, ASAP = atmospheric pressure solids analysis probe, APCI = atmospheric pressure chemical ionization).

$K_3[Cu(B_{11}H_{11})_2]$

^{1}H NMR (400.3 MHz, CD_3CN): δ = 4.30−1.07 (m, 22H $(B_{11}H_{11})_2$) ppm.
^{1}H{^{11}B} NMR (400.3 MHz, CD_3CN): δ = 3.70 (s, 2H, B_1−H), 2.83 (s, 10H, $B_{2–6}$−H), 1.71 (s, 10H, $B_{7–11}$−H) ppm.
^{11}B NMR (128.4 MHz, CD_3CN): δ = 22.7 (d, 2B, B_1−H, ^{1}J(^{11}B, ^{1}H) = 126.9 Hz), 13.7 (d, 10B, $B_{2–6}$−H, ^{1}J(^{11}B, ^{1}H) = 122.3 Hz), −6.9 (d, 10B, $B_{7–11}$−H, ^{1}J(^{11}B, ^{1}H) = 129.3 Hz) ppm.
^{11}B{^{1}H} NMR (128.4 MHz, CD_3CN): *d* = 22.7 (s, 2B, B_1−H), 13.7 (s, 10B, $B_{2–6}$−H), −6.9 (s, 10B, $B_{7–11}$−H) ppm.
HRMS *m/z* (isotopic abundance >10) calculated for $K[Cu(B_{11}H_{11})_2]^{2-}$: 181.1429 (100.), 180.6447 (89), 181.6411 (85), 180.1465 (63), 182.1392 (51), 181.6438 (46), 182.1420 (45), 182.6402 (38), 179.6483 (36), 181.1456 (28), 183.1383 (27), 182.6374 (20), 179.1501 (17), 180.6474 (16).
found: 181.1421 (100), 180.6439 (88), 181.6403 (88), 180.1457 (78), 182.1411 (56), 181. 6429 (47), 182.6393 (46), 179.6475 (41), 181.1447 (40), 183.1375 (26), 182.6366 (22), 179.1493 (13).

$K_3[Ag(B_{11}H_{11})_2]$

^{1}H NMR (400.3 MHz, D_2O): δ = 4.67−1.33 (m, 22H B_1−H) ppm.
^{1}H{^{11}B} NMR (400.3 MHz, D_2O): δ = 4.01 (s, 2H, B_1−H), 3.05 (s, 10H, $B_{2–6}$−H), 2.04 (s, 10H, $B_{7–11}$−H) ppm.
^{11}B NMR (128.4 MHz, D_2O): δ = 17.1 (d, 2B, B_1−H, ^{1}J(^{11}B, ^{1}H) = 123.9 Hz), 11.7 (d, 10B, $B_{2–6}$−H, ^{1}J(^{11}B, ^{1}H) = 124.1 Hz), −9.1 (d, 10B, $B_{7–11}$−H, ^{1}J(^{11}B, ^{1}H) = 130.4 Hz) ppm.
^{11}B{^{1}H} NMR (128.4 MHz, D_2O): δ = 17.1 (s, 2B, B_1−H), 11.7 (s, 10B, $B_{2–6}$−H), −9.1 (s, 10B, $B_{7–11}$−H,) ppm.
HRMS *m/z* (isotopic abundance >10) calculated for $K[Ag(B_{11}H_{11})_2]^{2-}$: 203.1306 (100), 204.1305 (93), 202.6324 (89), 203.6288 (85), 203.6323 (83), 204.6286 (79), 202.1343 (63), 203.1341 (59), 204.1270 (51), 205.1268 (48), 201.6361 (36), 202.6359 (33), 204.6252 (20), 205.6250 (18), 201.1379 (17), 202.1377 (16).
found: 204.1298 (100), 203.1300 (99), 202.6318 (91), 203.6316 (86), 203.6282 (81), 202.1336 (78), 204.6280 (74), 203.1334 (69), 204.1264 (62), 205.1262 (52), 201.6355 (40), 202.6352 (35), 205.6244 (21), 202.1371 (18), 206.2231 (14), 201.1371 (12), 204.2198 (11).

$K_3[Au(B_{11}H_{11})_2]$

^{1}H NMR (400.3 MHz, CD_3CN): δ = 5.32−4.06 (m, 2H, B_1−H), 3.00−1.20 (m, 20H, $B_{2–11}$−H) ppm.
^{1}H{^{11}B} NMR (400.3 MHz, CD_3CN): δ = 4.72 (s, 2H, B_1−H), 2.27 (s, 10H, $B_{2–6}$−H), 1.96 (s, 10H, $B_{7–11}$−H) ppm.
^{11}B NMR (128.4 MHz, CD_3CN): δ = 13.2 (d, 2B, B_1−H, ^{1}J(^{11}B, ^{1}H) = 127.4 Hz), 3.8 (d, 10B, $B_{2–6}$−H, ^{1}J(^{11}B, ^{1}H) = 128.0 Hz), −12.9 (d, 10B, $B_{7–11}$−H, ^{1}J(^{11}B, ^{1}H) = 124.8 Hz) ppm.
^{11}B{^{1}H} NMR (128.4 MHz, CD_3CN): δ = 13.2 (s, 2B, B_1−H), 3.8 (s, 10B, $B_{2–6}$−H), −12.9 (s, 10B, $B_{7–11}$−H) ppm.
HRMS *m/z* (isotopic abundance >10): 248.1614 (100), 247.6632 (89), 248.6595 (84), 247.1650 (63), 249.1577 (51), 246.6668 (36), 249.6559 (20), 246.1686 (17).
found: 248.1612 (100), 248.6630 (88), 248.6593 (82), 247.1649 (64), 247.1574 (49), 246.6667 (35), 249.6555 (17), 246.1685 (15).

$[n\text{-}Bu_4N]_3[Cu(B_{11}H_{11})_2]$

^{1}H NMR (400.3 MHz, CD_3CN): δ = 4.33−1.07 (m, 22H $(B_{11}H_{11})_2$), 3.10 (m, 8H, $NC\mathbf{H_2}CH_2CH_2CH_3$), 1.61 (m, 8H, $NCH_2C\mathbf{H_2}CH_2CH_3$), 1.36 (m, 8H, $NCH_2CH_2C\mathbf{H_2}CH_3$), 0.97 (t, 12H, $NCH_2CH_2CH_2C\mathbf{H_3}$, $^3J(^1H, ^1H)$ = 7.4 Hz) ppm.

^{1}H{^{11}B} NMR (400.3 MHz, CD_3CN): δ = 3.66 (s, 2H, B_1−H), 3.10 (m, 8H, $NC\mathbf{H_2}CH_2CH_2CH_3$), 2.85 (s, 10H, B_{7-11}−H), 1.68 (s, 10H, B_{7-11}−H), 1.61 (m, 8H, $NCH_2C\mathbf{H_2}CH_2CH_3$), 1.36 (m, 8H, $NCH_2CH_2C\mathbf{H_2}CH_3$), 0.97 (t, 12H, $NCH_2CH_2CH_2C\mathbf{H_3}$, $^3J(^1H, ^1H)$ = 7.4 Hz) ppm.

^{11}B NMR (128.4 MHz, CD_3CN): δ = 22.0 (d, 2B, B_1−H, $^1J(^{11}B, ^1H)$ = 126.7 Hz), 13.6 (d, 10B, B_{2-6}−H, $^1J(^{11}B, ^1H)$ = 122.3 Hz), −7.1 (d, 10B, B_{7-11}−H, $^1J(^{11}B, ^1H)$ = 128.8 Hz) ppm.

^{11}B{^{1}H} NMR (128.4 MHz, CD_3CN): δ = 22.0 (s, 2B, B_1−H), 13.6 (s, 10B, B_{2-6}−H), −7.1 (s, 10B, B_{7-11}−H) ppm.

^{13}C NMR (100.7 MHz, CD_3CN): δ = 59.4 (t, 1C, $N\mathbf{C}H_2CH_2CH_2CH_3$, $^1J(^{13}C, ^1H)$ = 143.0 Hz), 24.4 (t, 1C, $NCH_2CH_2\mathbf{C}H_2CH_3$, $^1J(^{13}C, ^1H)$ = 127.4 Hz), 20.3 (t, 1C, $NCH_2\mathbf{C}H_2CH_2CH_3$, $^1J(^{13}C, ^1H)$ = 125.8 Hz), 13.8 (qtt, 1C, $NCH_2CH_2CH_2\mathbf{C}H_3$, $^1J(^{13}C, ^1H)$ = 125.3 Hz, $^2J(^{13}C, ^1H)$ = 7.8 Hz, $^3J(^{13}C, ^1H)$ = 3.9 Hz) ppm.

^{13}C{^{1}H} NMR (100.7 MHz, CD_3CN): δ = 59.4 (t, 1C, $N\mathbf{C}H_2CH_2CH_2CH_3$, $^1J(^{15}N, ^{13}C)$ = 2.9 Hz), 24.4 (s, 1C, $NCH_2CH_2\mathbf{C}H_2CH_3$), 20.3 (t, 1C, $NCH_2\mathbf{C}H_2CH_2CH_3$, $^2J(^{15}N, ^{13}C)$ = 1.4 Hz), 13.8 (s, 1C, $NCH_2CH_2CH_2\mathbf{C}H_3$) ppm.

HRMS *m/z* (isotopic abundance >10) calculated for $[nBu_4N]_3[Cu(B_{11}H_{11})_2]^{2-}$: 282.8034 (100), 282.3052 (89), 283.3016 (85), 281.8070 (63), 283.7998 (51), 283.8025 (45), 283.3043 (40), 284.3007 (38), 281.3089 (36), 282.8061 (28), 284.7989 (23), 284.2980 (20), 283.3051 (17), 280.8107 (17), 282.3080 (16), 282.8069 (16), 283.8033 (15), 282.3087 (11).
found: 282.3049 (100), 283.8033 (83), 283.3015 (82), 281.8067 (56), 284.3011 (49), 283.7997 (45), 283.8028 (43), 281.3087 (40), 282.8063 (37), 283.3046 (35), 284.7989 (30), 282.3082 (17), 280.8105 (17), 284.2987 (15).

$[n\text{-}Bu_4N]_3[Ag(B_{11}H_{11})_2]$

^{1}H NMR (400.3 MHz, CD_3CN): δ = 4.33−1.07 (m, 22H $(B_{11}H_{11})_2$), 3.10 (m, 8H, $NC\mathbf{H_2}CH_2CH_2CH_3$), 1.61 (m, 8H, $NCH_2C\mathbf{H_2}CH_2CH_3$), 1.36 (m, 8H, $NCH_2CH_2C\mathbf{H_2}CH_3$), 0.97 (t, 12H, $NCH_2CH_2CH_2C\mathbf{H_3}$, $^3J(^1H, ^1H)$ = 7.4 Hz) ppm.

^{1}H{^{11}B} NMR (400.3 MHz, CD_3CN): δ = 3.69 (s, 2H, B_1−H), 3.10 (m, 8H, $NC\mathbf{H_2}CH_2CH_2CH_3$), 2.82 (s, 10H, B_{7-11}−H), 1.75 (s, 10H, B_{7-11}−H), 1.61 (m, 8H, $NCH_2C\mathbf{H_2}CH_2CH_3$), 1.36 (m, 8H, $NCH_2CH_2C\mathbf{H_2}CH_3$), 0.97 (t, 12H, $NCH_2CH_2CH_2C\mathbf{H_3}$, $^3J(^1H, ^1H)$ = 7.4 Hz) ppm.

^{11}B NMR (128.4 MHz, CD_3CN): δ = 16.6 (d, 2B, B_1−H, $^1J(^{11}B, ^1H)$ = 120.6 Hz), 11.7 (d, 10B, B_{2-6}−H, $^1J(^{11}B, ^1H)$ = 129.2 Hz), −9.0 (d, 10B, B_{7-11}−H, $^1J(^{11}B, ^1H)$ = 129.3 Hz) ppm.

^{11}B{^{1}H} NMR (128.4 MHz, CD_3CN): δ = 16.6 (s, 2B, B_1−H), 11.7 (s, 10B, B_{2-6}−H), −9.0 (s, 10B, B_{7-11}−H) ppm.

^{13}C NMR (100.7 MHz, CD_3CN): δ = 59.4 (t, 1C, $N\mathbf{C}H_2CH_2CH_2CH_3$, $^1J(^{13}C, ^1H)$ = 143.4 Hz), 24.4 (t, 1C, $NCH_2CH_2\mathbf{C}H_2CH_3$, $^1J(^{13}C, ^1H)$ = 127.6 Hz), 20.3 (t, 1C, $NCH_2\mathbf{C}H_2CH_2CH_3$, $^1J(^{13}C, ^1H)$ = 126.6 Hz), 13.8 (qtt, 1C, $NCH_2CH_2CH_2\mathbf{C}H_3$, $^1J(^{13}C, ^1H)$ = 125.3 Hz, $^2J(^{13}C, ^1H)$ = 7.7 Hz, $^3J(^{13}C, ^1H)$ = 3.8 Hz) ppm.

^{13}C{^{1}H} NMR (100.7 MHz, CD_3CN): δ = 59.4 (t, 1C, $N\mathbf{C}H_2CH_2CH_2CH_3$, $^1J(^{15}N, ^{13}C)$ = 2.8 Hz), 24.4 (s, 1C, $NCH_2CH_2\mathbf{C}H_2CH_3$), 20.3 (t, 1C, $NCH_2\mathbf{C}H_2CH_2CH_3$, $^2J(^{15}N, ^{13}C)$ = 1.4 Hz), 13.8 (s, 1C, $NCH_2CH_2CH_2\mathbf{C}H_3$) ppm.

HRMS *m/z* (isotopic abundance >10) calculated for $[nBu_4N]_2[Ag(B_{11}H_{11})_2]^-$: 851.8665 (100), 854.8626 (96), 853.8662 (93), 851.8735 (89), 850.8702 (89), 852.8629 (85), 852.8698 (83), 849.8738 (63), 853.8593 (51), 855.8589 (48), 854.8696 (37), 848.8774 (36), 852.8699 (35), 850.8771 (33), 853.8663 (29), 853.8732 (29), 855.8659 (27), 850.8772 (22), 852.8768 (20), 854.8556 (20), 856.8553 (18), 847.8811 (17).
found: 852.8685 (100), 851.8704 (97), 853.8668 (90), 850.8726 (79), 854.8641 (69), 849.8758 (52), 855.8612 (41), 848.8793 (28), 856.8585 (16), 847.8827 (12).

Results of the DSC, DTG and STA measurements.

Thermogravimetric (TG) and Differential Scanning Calorimetry (DSC) measurements were carried out simultaneously using a Netzsch STA 449 F5 Jupiter instrument. Experiments were conducted in 80 µL aluminium crucibles, closed with aluminium lids. Samples were heated from 20 to 600 °C with a heating rate of 5 K·min^{-1} in a nitrogen atmosphere applying a constant nitrogen flow of 25 ml·min^{-1} during the measurement.

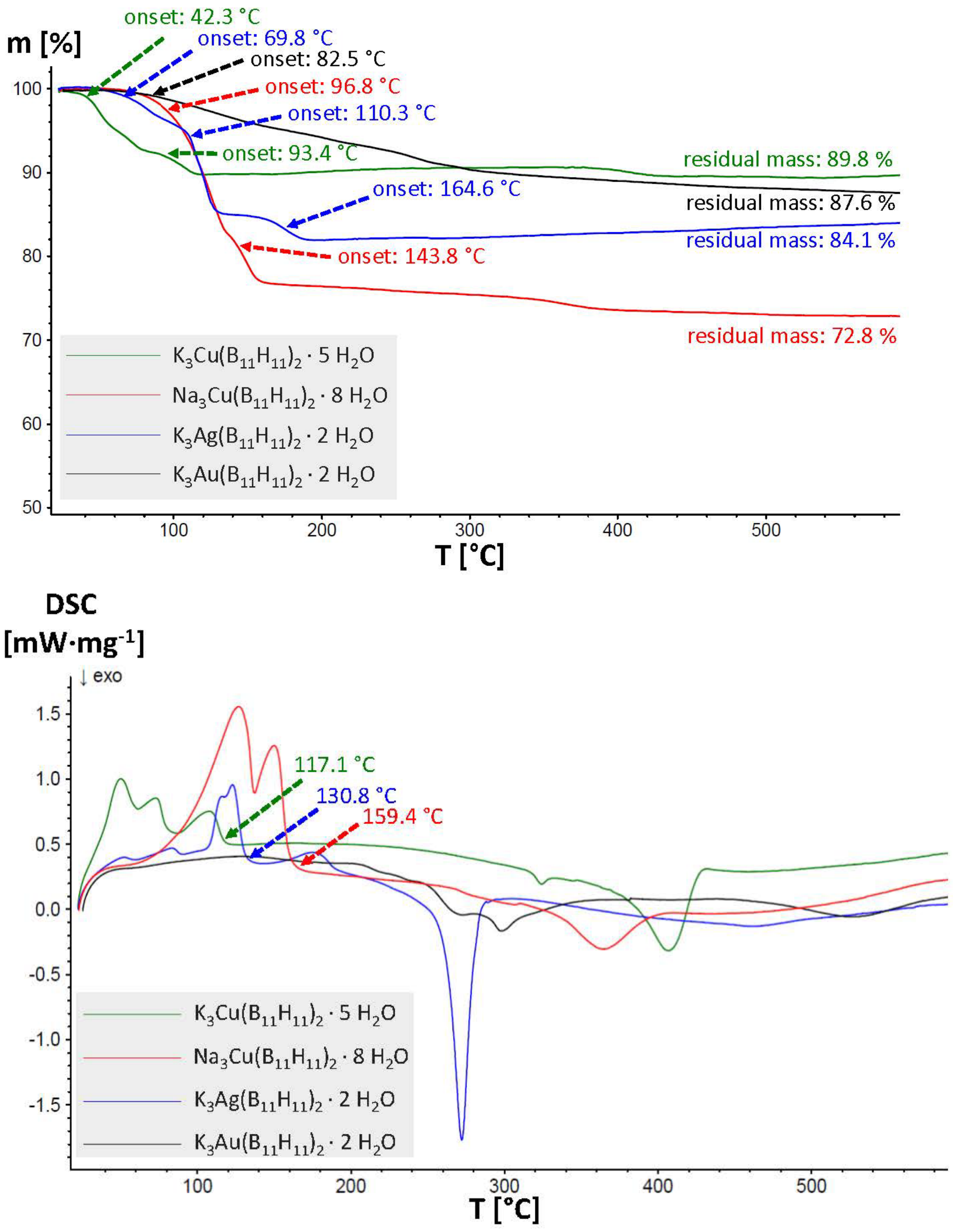

Figure S7. DSC/DTG of $Na_3[Cu(B_{11}H_{11})_2]\cdot 8H_2O$ and $K_3[M(B_{11}H_{11})_2]\cdot nH_2O$ (M = Cu, Ag, Au)

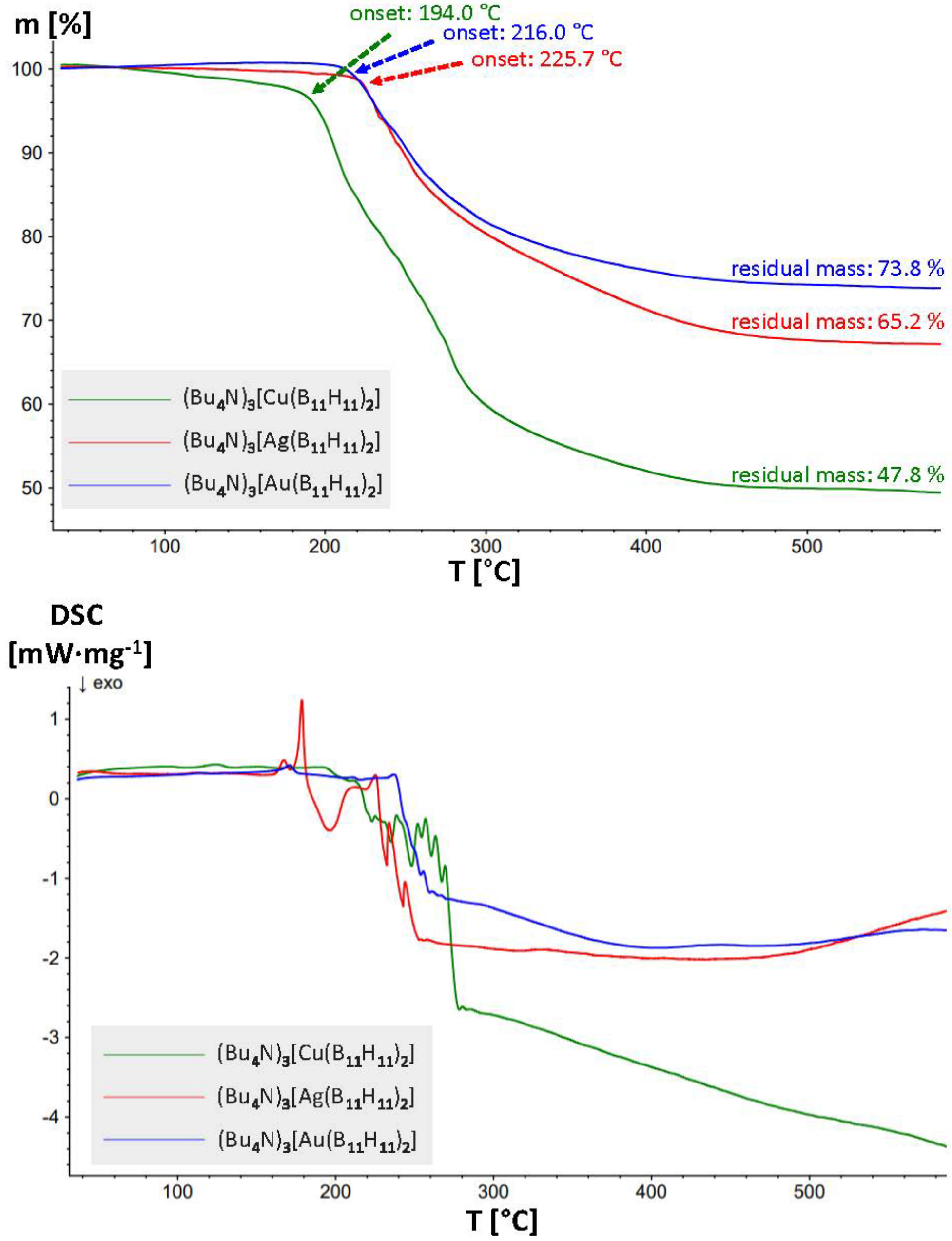

Figure S8. DSC/DTG of $(n\text{-}Bu_4N)_3[M(B_{11}H_{11})_2]$ (M = Cu, Ag, Au)

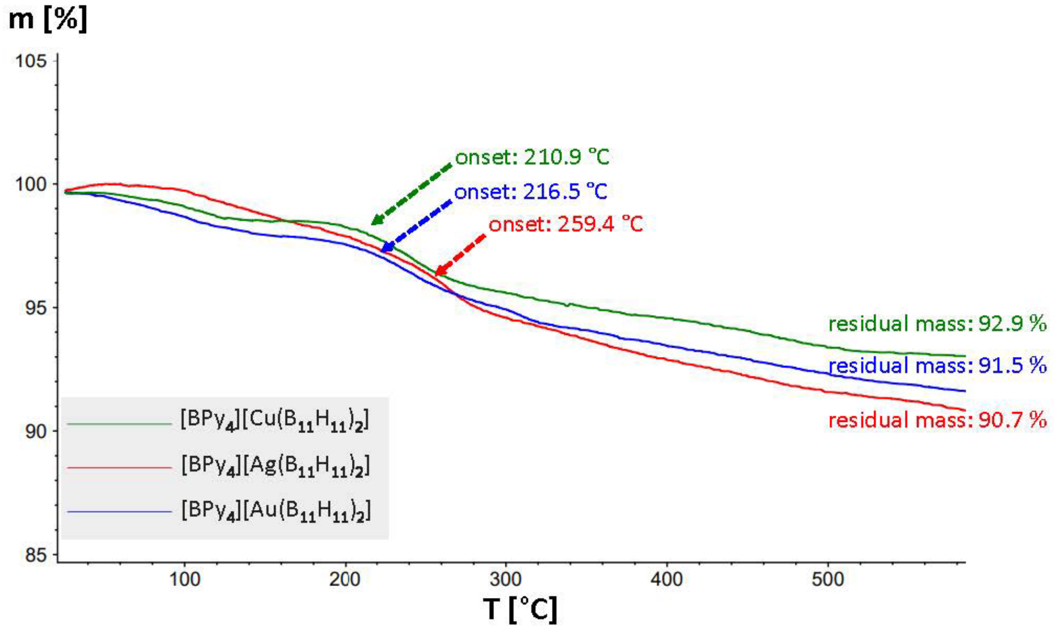

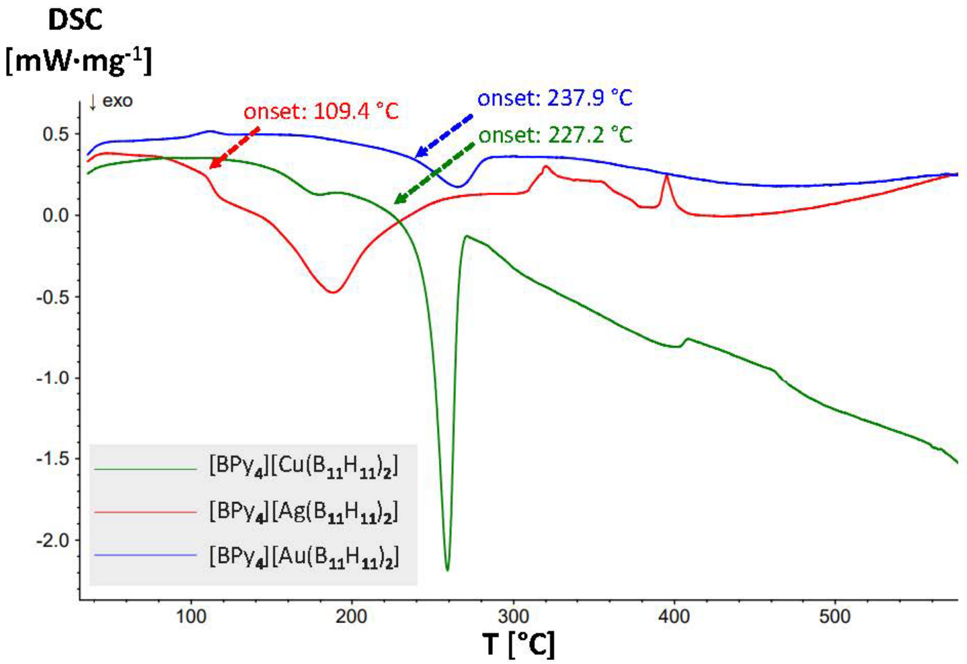

Figure S9. DSC/DTG of $BPy_4[M(B_{11}H_{11})_2]$ (M = Cu, Ag, Au)

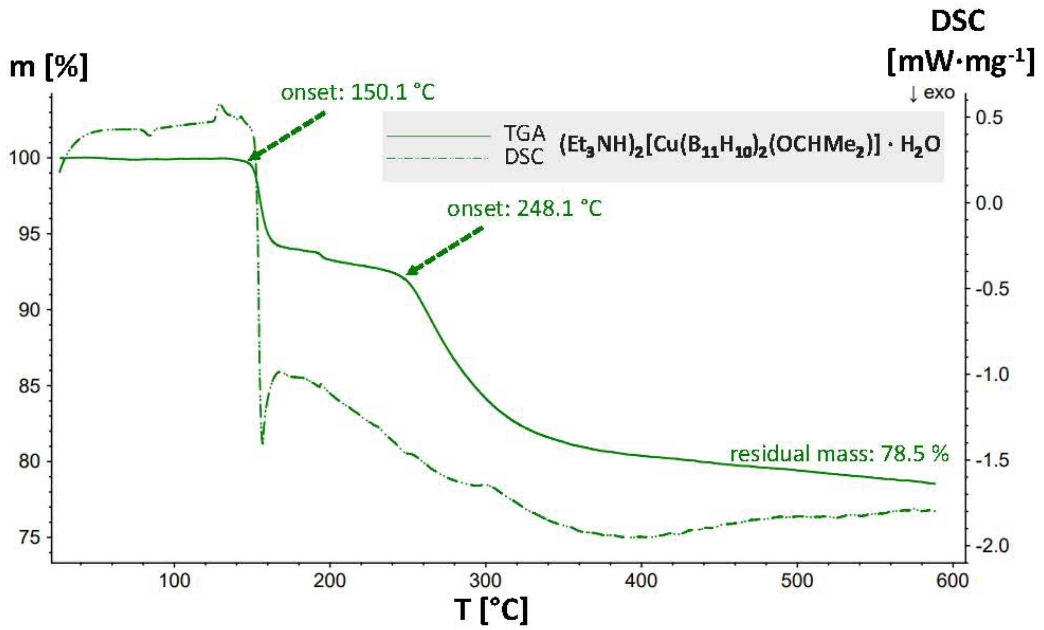

Figure S10. DSC/DTG of $(Et_3NH)_3[Cu(B_{11}H_{10})_2\,(OCHMe_2)]\cdot H_2O$

DSC/STA analyses were performed with a DSC 204 F1 Phoenix (Netzsch) in N_2 in the temperature range of −20 to 550 °C with a heating rate of 10 K·min^{-1} and with a STA 449 F3 Perseus (Netzsch), connected to an Alpha FTIR spectrometer (Bruker) and a QMS 403 Aeolos Quadro Mass Spectrometer for the analysis of the gaseous decomposition products in the temperature range of 30 °C to 700 °C with a heating rate of 10 K·min^{-1} in an Ar.

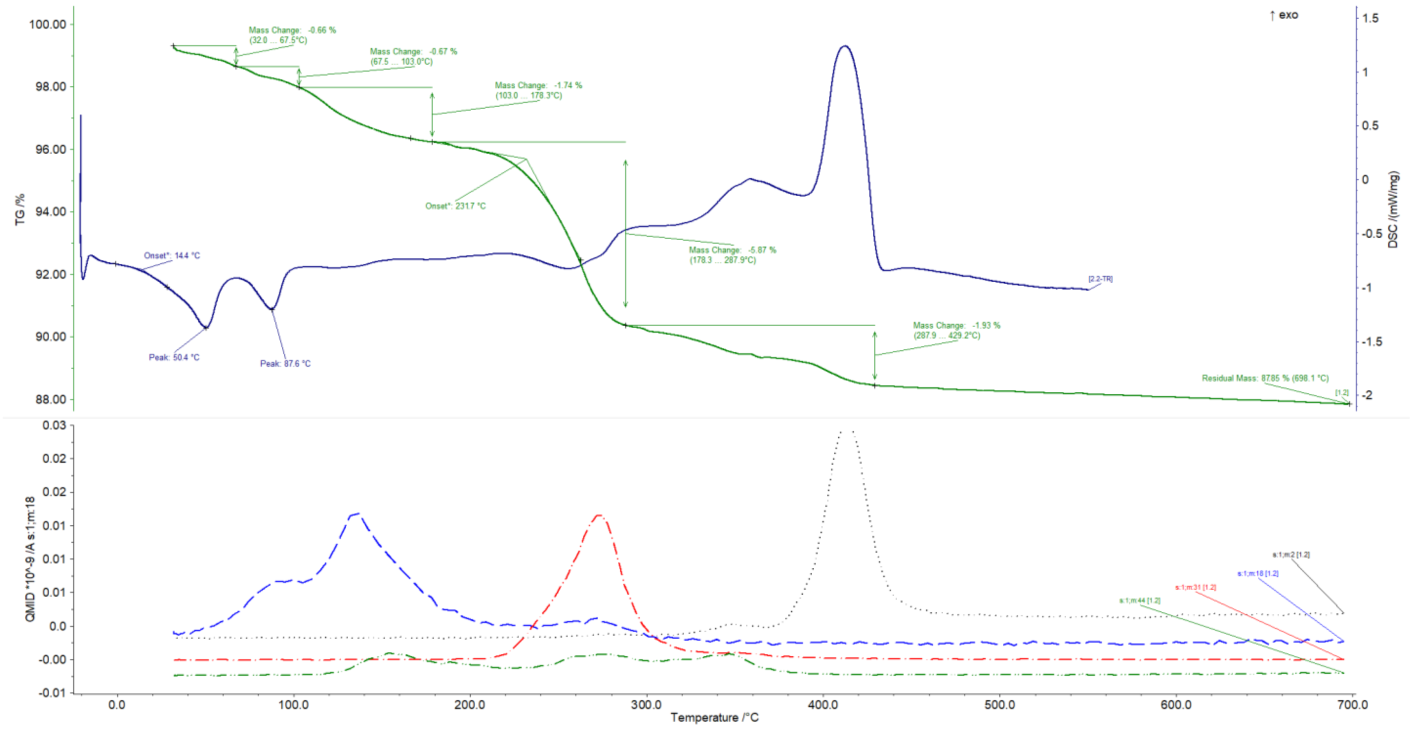

Figure S11. DSC/STA of $K_3[Cu(B_{11}H_{11})_2]$

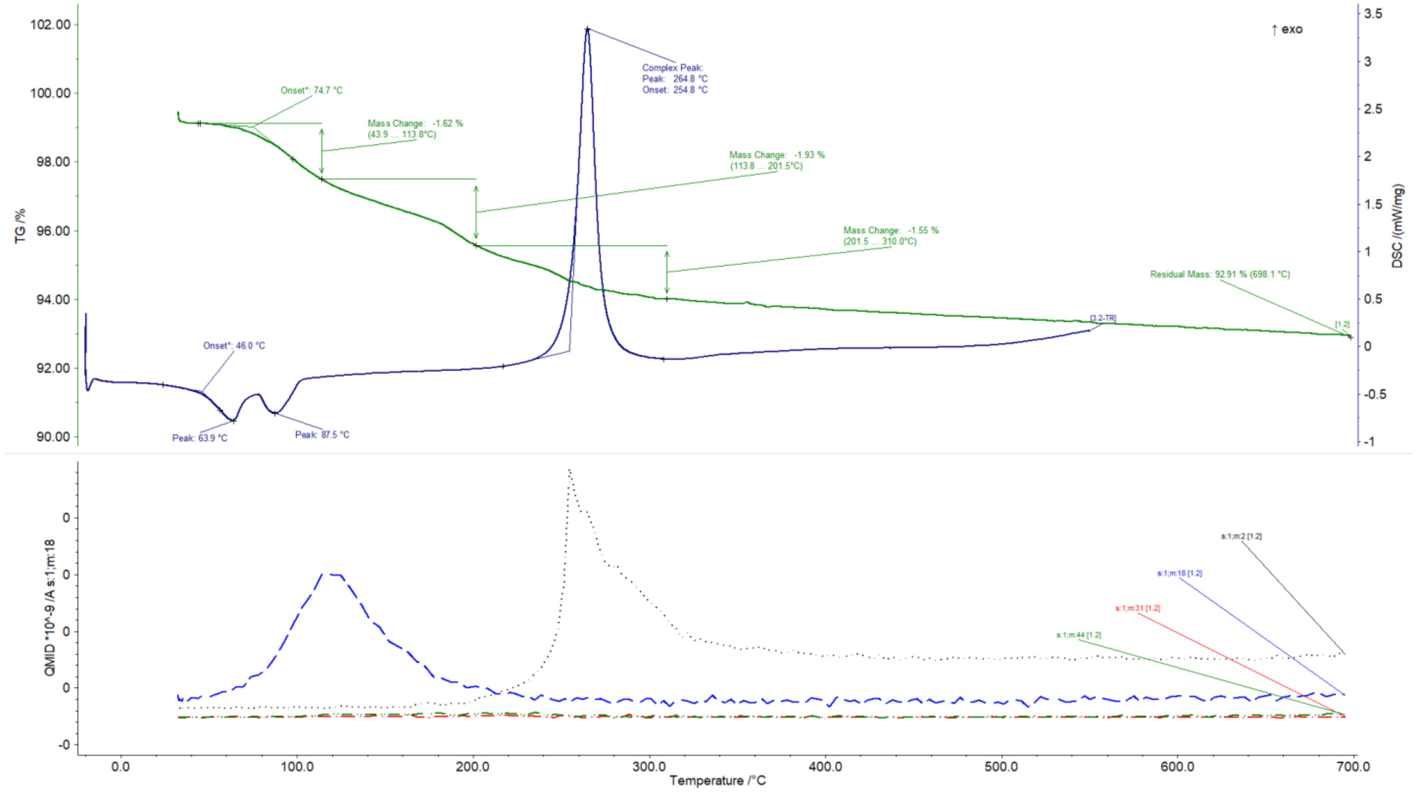

Figure S12. DSC/STA of $K_3[Ag(B_{11}H_{11})_2]$

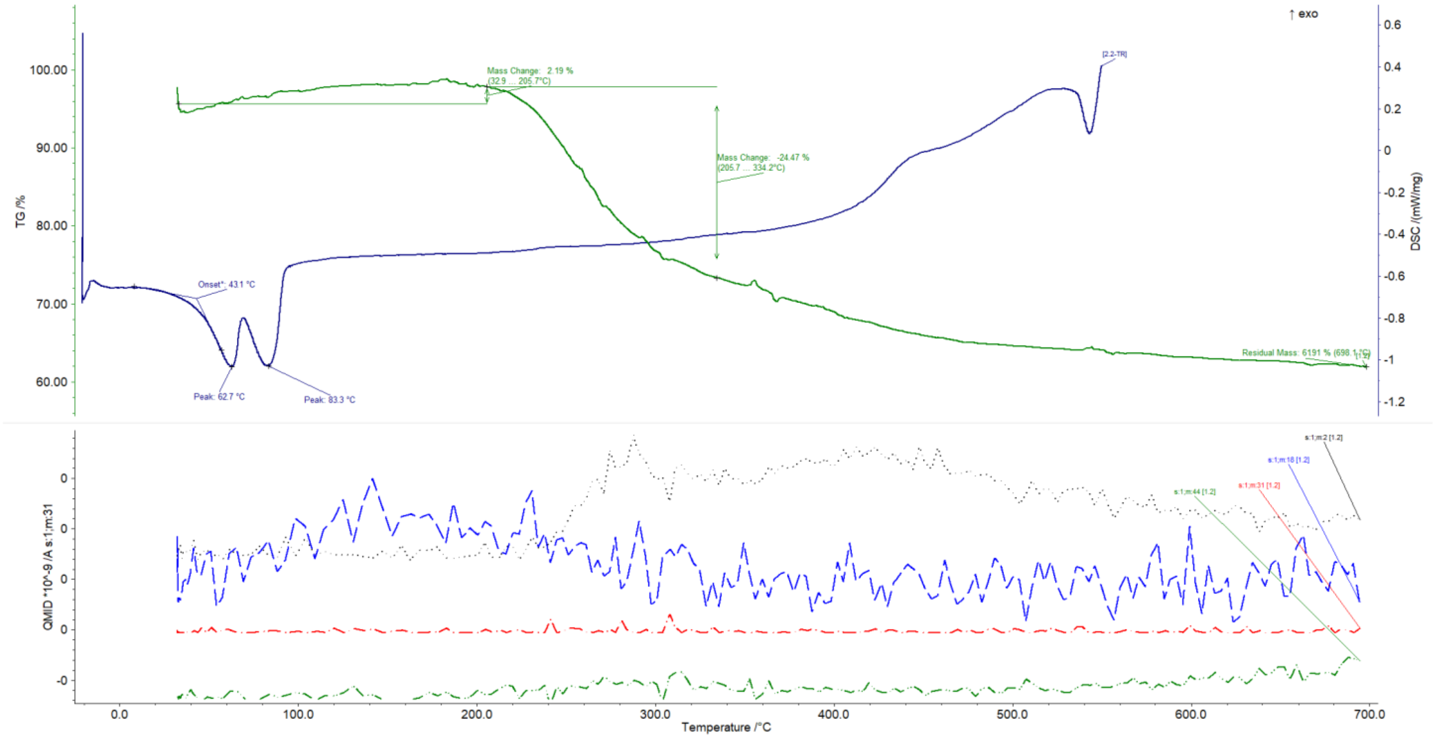

Figure S13. DSC/STA of $K_3[Au(B_{11}H_{11})_2]$

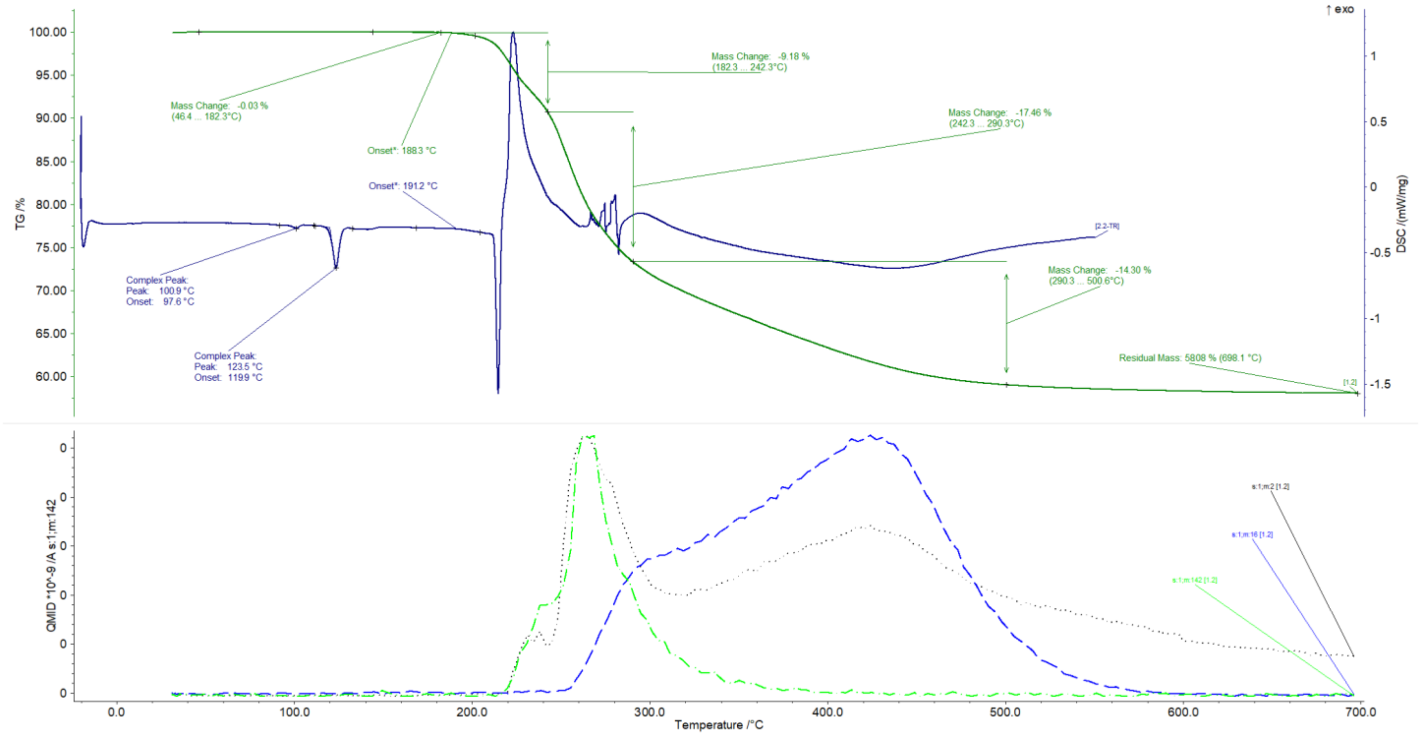

Figure S14. DSC/STA of $(n\text{-}Bu_4N)_3[Cu(B_{11}H_{11})_2]$

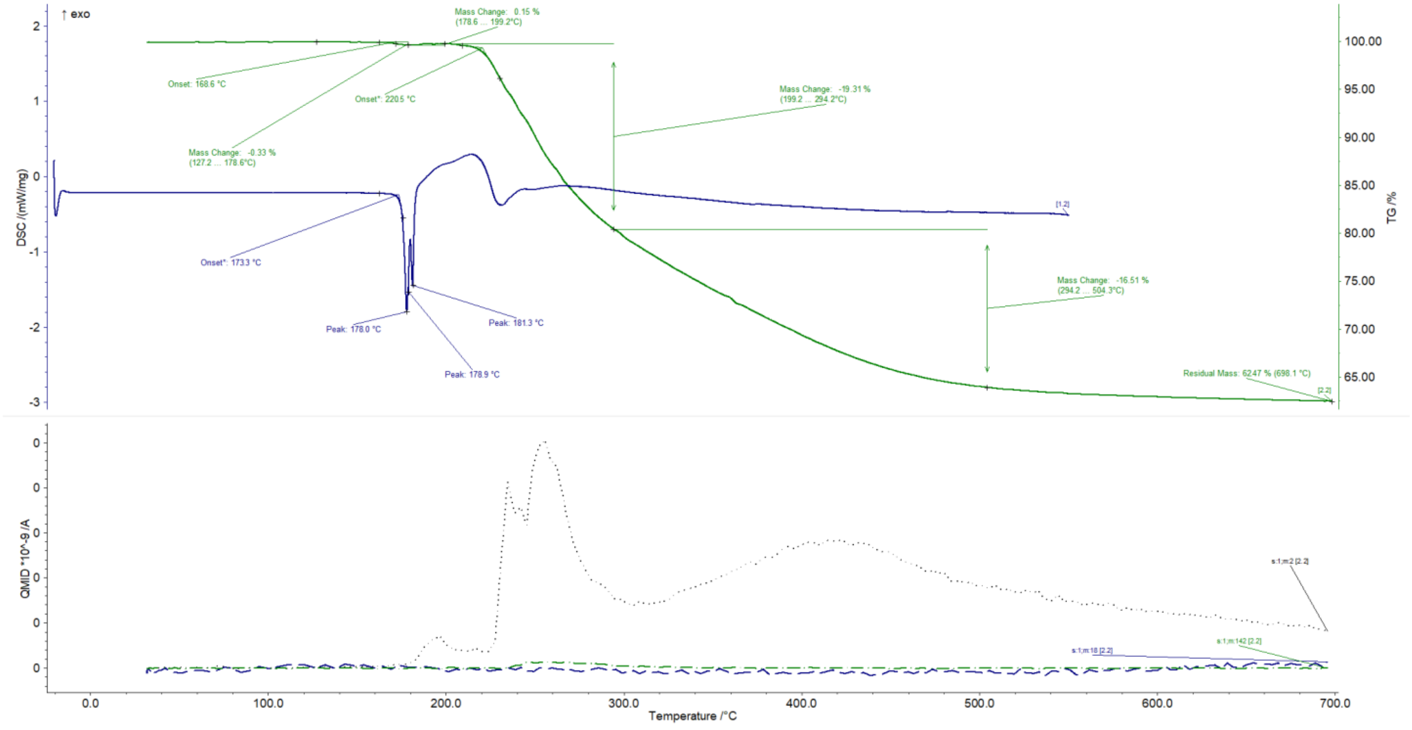

Figure S15. DSC/STA of $(n\text{-}Bu_4N)_3[Ag(B_{11}H_{11})_2]$

Cyclic Voltammetry.

Cyclic voltammetry was performed with a potentiostat (BioLogic SP−300) operating in a three−electrode setup. A carbon paper electrode, an Ag/AgCl electrode in a sat. KCl solution and a platinum wire were used as a working electrode, a reference electrode, and a counter electrode, respectively. Furthermore, additional experiments were carried out utilizing a two−electrode configuration comprising either two platinum electrodes or two carbon paper electrodes, with the Ag/AgCl electrode in sat. KCl solution consistently used as the reference. The potential window was set to range from −2.7 V to +1.9 V vs. Fc/Fc^+. The measurements were performed in an electrolyte consisting of 159 mg of $[(n\text{-Bu})_4N]_3[Cu(B_{11}H_{11})_2]$ dissolved in 10 mL of acetonitrile. Additionally, electrolytes containing 251 mg of $[(n\text{-Bu})_4N]_3[Ag(B_{11}H_{11})_2]$ or 156 mg of $[(n\text{-Bu})_4N]_3[Au(B_{11}H_{11})_2]$ dissolved in 10 mL of acetonitrile were also investigated.

We performed cyclic voltammetry measurements of $(n\text{-Bu}_4N)_3[M(B_{11}H_{11})_2]$ (M = Cu, Ag, Au) solutions in solution in the range −2.7 V to +1.9 V vs. Fc/Fc^+ (−2.3 V to 2.3 V vs. RHE, −2.55 V to 2.05 V vs. SCE, −2.52 V to 2.08 V vs. Ag/AgCl). In the case of M = Cu and Au, we observed neither oxidation nor reduction of $[M(B_{11}H_{11})_2]^{3-}$ in this range. Irreversible oxidations of $[Cu(B_{11}H_{11})_2]^{3-}$ at +0.78 and +1.14 V (vs. SCE) observed in a previous publication[23] could not be confirmed.

During the measurements, the solutions of $(n\text{-Bu}_4N)_3[Ag(B_{11}H_{11})_2]$ quickly turned brown due to decomposition, so we were not able to attribute any redox processes to the anion. The instability of $[Ag(B_{11}H_{11})_2]^{3-}$ is also noticeable during synthesis and crystallization and is likely due to the equilibrium between $[M^{+V}(nido\text{-}B_{11}X_{11})_2]^{3-}$ and $[M^{+I}(closo\text{-}B_{11}X_{11})_2]^{3-}$ isomers. The kinetically unstable $[M^{+I}(closo\text{-}B_{11}X_{11})_2]^{3-}$ isomer, present in small amounts, is reduced or undergoes reactions with compounds that typically react with Ag^+ (e.g., S^{2-}, CN^-, I^-, Br^-, Cl^-).

UV-Vis Spectroscopy.

UV-vis spectra of $K_3[Cu(B_{11}H_{11})_2]\cdot 5H_2O$, $K_3[Ag(B_{11}H_{11})_2]\cdot 5H_2O$ and $K_3[Au(B_{11}H_{11})_2]\cdot 2H_2O$ were recorded on a VWR UV-3100PC spectrophotometer. For each wavelength (λ) the extinction coefficient (*ε*) was calculated using Lambert-Beer's law, with the measured extinction (*E*), the concentration (*c*), and the thickness (*d*):

$$\varepsilon(\lambda) = \frac{E}{c \cdot d}$$

UV-Vis-Spectra

The energy splitting of the d-orbitals in the sandwich complexes $[ML_2]^{n\pm}$ can be compared to that of the octahedral complexes $[ML_6]^{n\pm}$. For a better comparison, the z-axis for the octahedral complexes $[ML_6]^{n\pm}$ should be considered along the threefold axis, and D_{3d} should be used as the point group instead of O_h. In this case, there are two types of d-orbitals: three $d_\pi(d_\varepsilon)$ (d_{z2}, $-\sin(\varepsilon)(d_{xz},d_{yz})+\cos(\varepsilon)(d_{xy},d_{x2-y2})$) and two $d_\sigma(d_\gamma)$ ($\cos(\varepsilon)(d_{xz},d_{yz})+\sin(\varepsilon)(d_{xy},d_{x2-y2})$). For octahedral complexes, ε = 35.3°, $\cos(\varepsilon) = (2/3)^{1/2}$ and $\sin(\varepsilon) = (1/3)^{1/2}$.[24] The strength of the ligand field is estimated by the energy difference (Δ_0) of the d_π and d_σ orbitals.[25] Typical values for Δ_0 are: $[Fe(CN)_6]^{4-}$ 33000 cm^{-1}, $[Co(NH_3)_6]^{3+}$ 23000 cm^{-1}, $[CoF_6]^{3-}$ 13000 cm^{-1} and $[Fe(H_2O)_6]^{2+}$ 10000 cm^{-1}.[26] In the point group D_{3d}, both d_{xz},d_{yz} and d_{xy},d_{x2-y2} orbitals have the same Mulliken symbol (E_g) and can be mixed with each other as necessary. This allows the d orbitals to be aligned with the ligand field. The situation is somewhat different for the $[M(B_{11}H_{11})_2]^{3-}$ (M = Cu, Ag, Au) complexes. The point group is D_{5d}, and the d_{xz},d_{yz} and d_{xy},d_{x2-y2} orbitals can not mix because they have different Mulliken symbols (E_{1g} for d_{xz},d_{yz} and E_{2g} for dxy,d_{x2-y2} orbitals). The d_{z2} orbital has the lowest energy, followed by the d_{xy}, d_{x2-y2} orbitals. Due to their stronger alignment with the ligands, the d_{xz},d_{yz} orbitals have the highest energy. The same applies to rotamers of $[M(B_{11}H_{11})_2]^{3-}$ (M = Cu, Ag, Au), which have

D_5 or D_{5h} symmetry. In contrast to the octahedral complexes, the orbitals in this case are less well adapted to the ligand field. We attempted to estimate the relative energies of the d orbitals in the $[M(B_{11}H_{11})_2]^{3-}$ (M = Cu, Ag, Au) complexes from DFT calculations (Tab. S4b, S12). The assignment is complicated by the strong mixing of the d orbitals with the cluster orbitals. The B-M bonds reveal a high degree of covalency, so ligand field theory is only of limited applicability in this case.

Table S4a. UV-Vis data for $K_3[M(B_{11}H_{11})_2]$ (M = Cu, Ag, Au).

M	Solvent	λ_1, (nm)	ν_1, (cm^{-1})	$\varepsilon_{\lambda 1}$, (cm^2/mmol)	λ_2, (nm)	ν_2, (cm^{-1})	$\varepsilon_{\lambda 2}$, (cm^2/mmol)	$\nu_2-\nu_1$, (cm^{-1})
Cu	H_2O	415	24100	1080, sh	305	32790	56600	8690
Cu	MeCN	418	23920	1060, sh	307.5	32520	56900	8600
Ag	H_2O	426	23470	540	317	31550	66200	8080
Ag	MeCN	429.5	23280	560	321.5	31100	64500	7820
Au	H_2O	333	30000	1700, sh	270.5	36970	45300	6970
Au	MeCN	336	29800	1800, sh	273.5	36560	45200	6760

Table S4b. Orbital energies (cm^{-1}) of the d-electrons in $[M(B_{11}H_{11})_2]^{3-}$ (M = Cu, Ag, Au). B3LYP/6-311++g(d,p) (B, H), /SDD (M); SCRF(Solvent=Water). There are a total of 50 MOs with valence electrons.

Orbitals	Cu	Ag	Au
$\Delta(d_{xz},d_{yz}/d_{xy},d_{x2-y2})$	37140	38306	42144
$\Delta(d_{xz},d_{yz}/d_{z2})$	52081	50990	54047
$\Delta(d_{xy},d_{x2-y2}/d_{z2})$	14941	12684	11903
d_{xz},d_{yz} [LUMO+x]	−16458[0,1]	−16873[0,1]	−12042[0,1]
d_{xy},d_{x2-y2} [HOMO−x]	−53598[3,4]	−55179[2,3]	−54186[2,3]
d_{z2} (+s) [HOMO−x]	−68539[17]	−67863[16]	−66089[15]

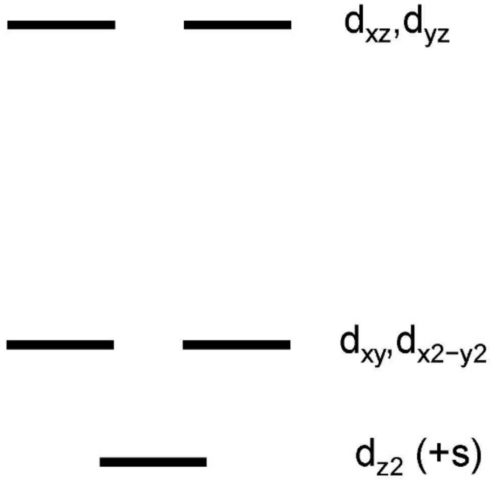

Figure S16a. MO diagram of $[M(B_{11}H_{11})_2]^{3-}$ (M = Cu, Ag, Au).

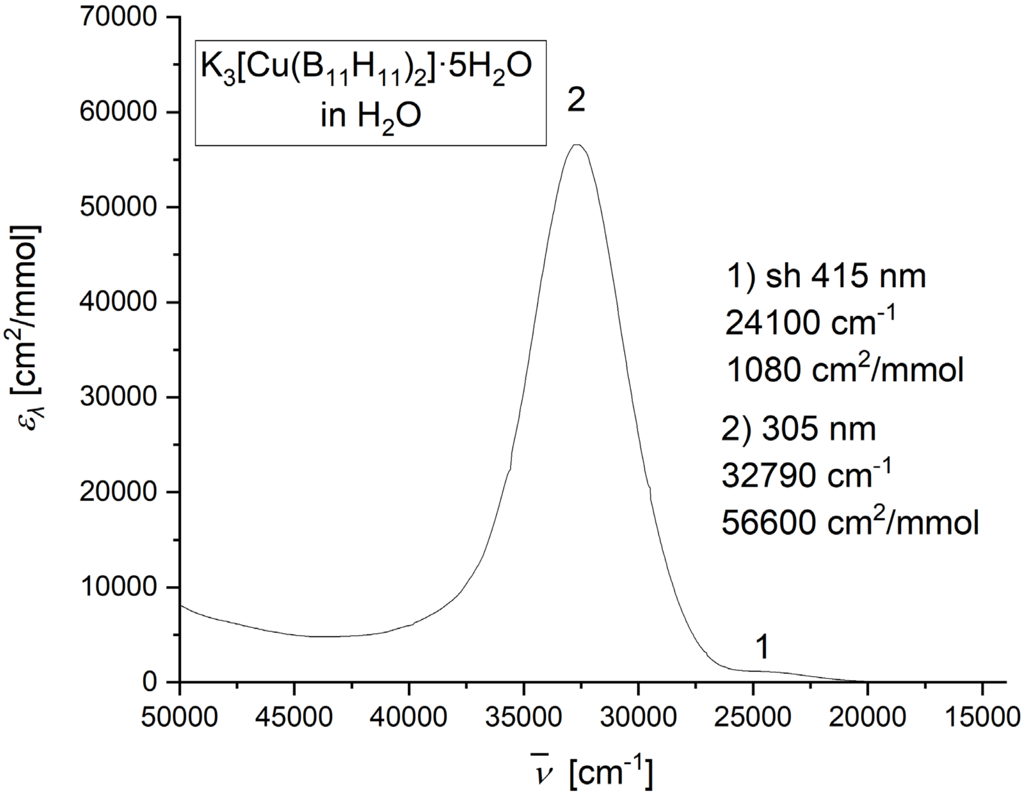

Figure S16. UV-Vis spectrum of $K_3[Cu(B_{11}H_{11})_2]$ in a water solution.

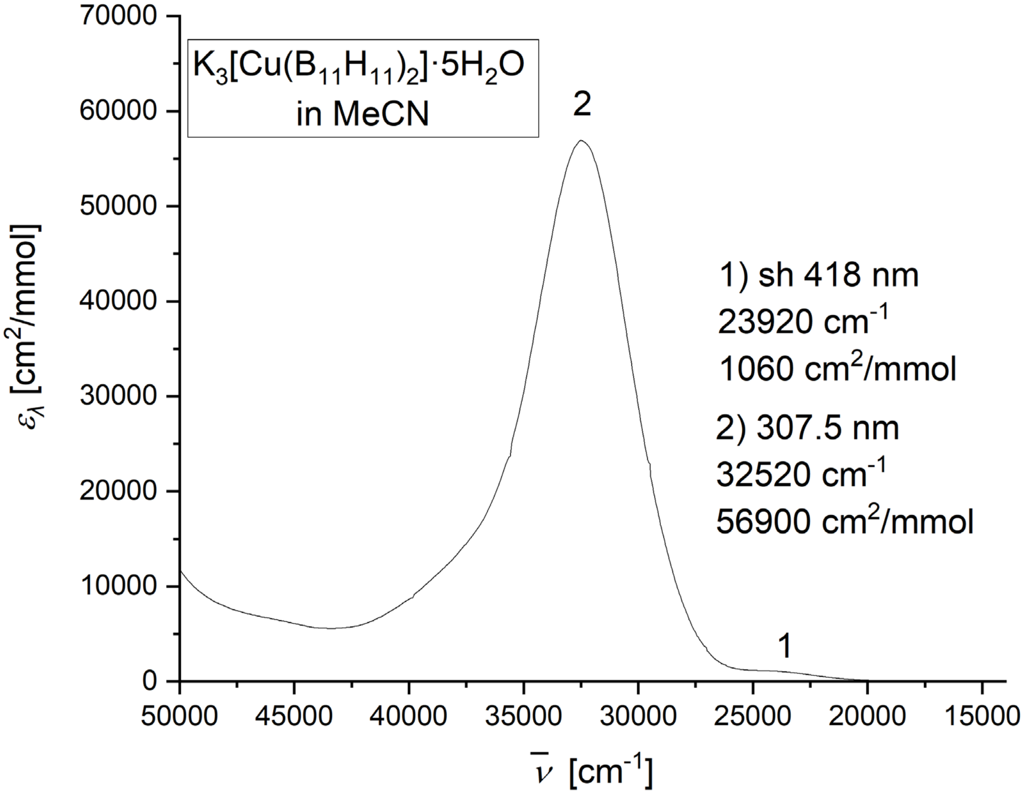

Figure S17. UV-Vis spectrum of $K_3[Cu(B_{11}H_{11})_2]$ in an acetonitrile solution.

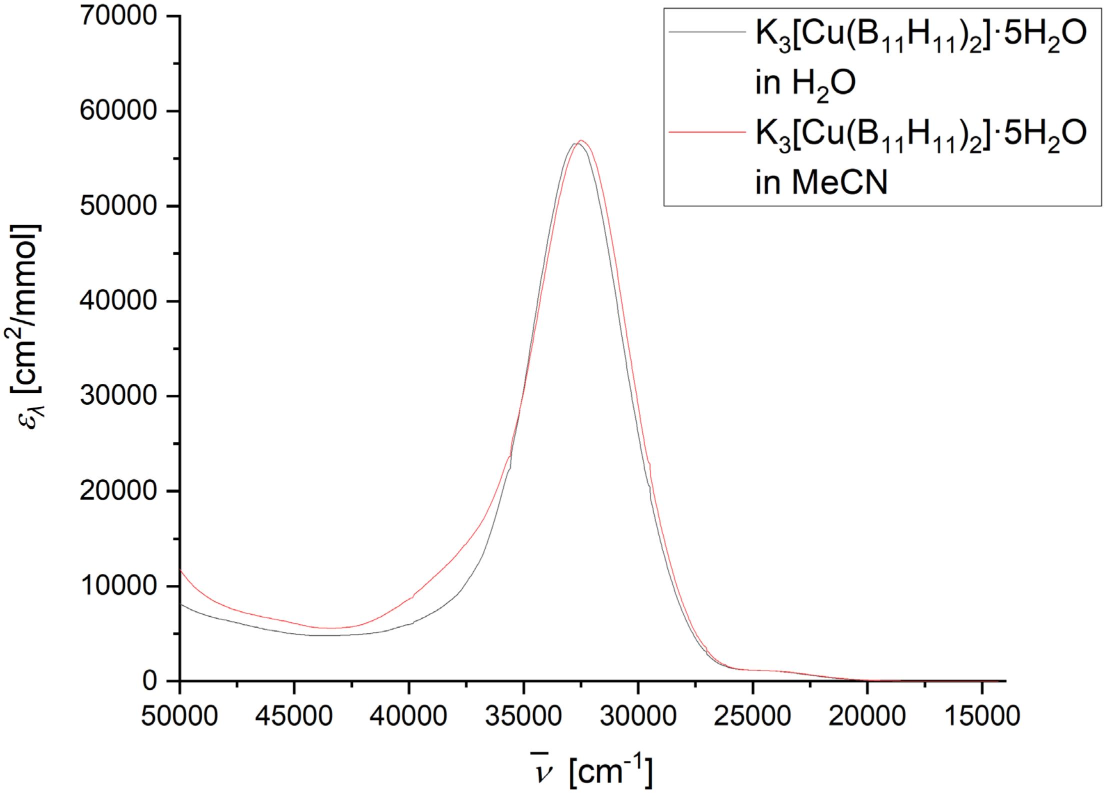

Figure S18. UV-Vis spectra of $K_3[Cu(B_{11}H_{11})_2]$

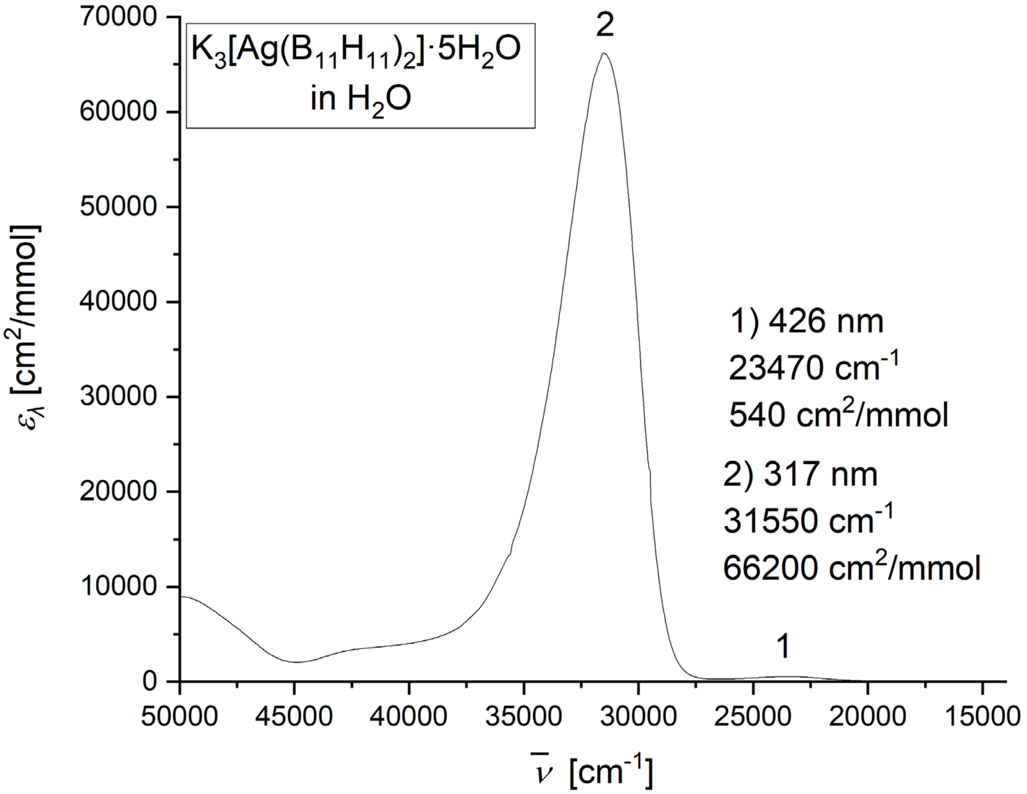

Figure S19. UV-Vis spectrum of $K_3[Ag(B_{11}H_{11})_2]$ in a water solution.

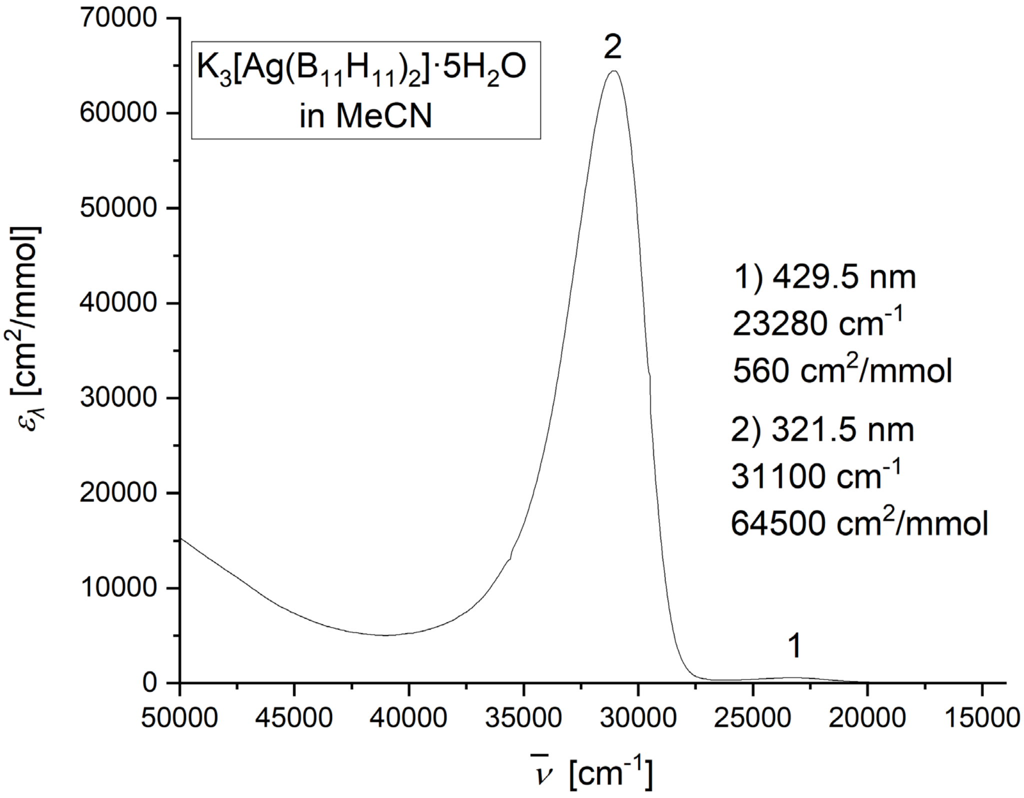

Figure S20. UV-Vis spectrum of $K_3[Ag(B_{11}H_{11})_2]$ in an acetonitrile solution.

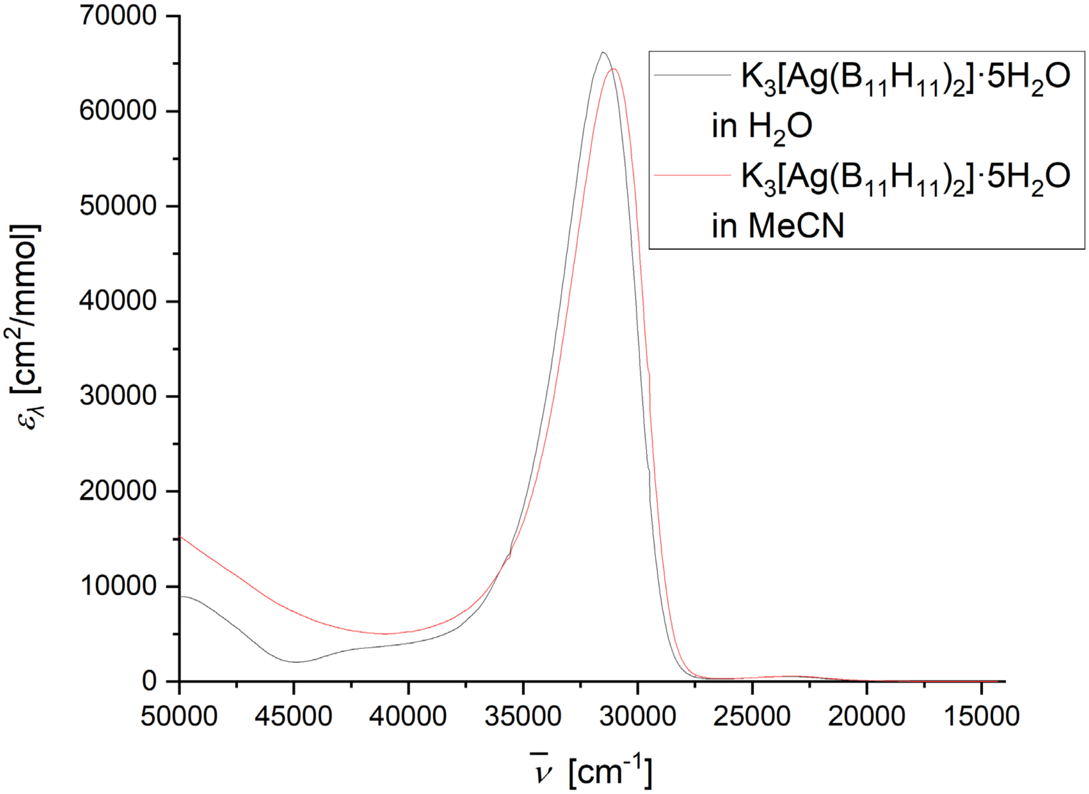

Figure S21. UV-Vis spectra of $K_3[Ag(B_{11}H_{11})_2]$.

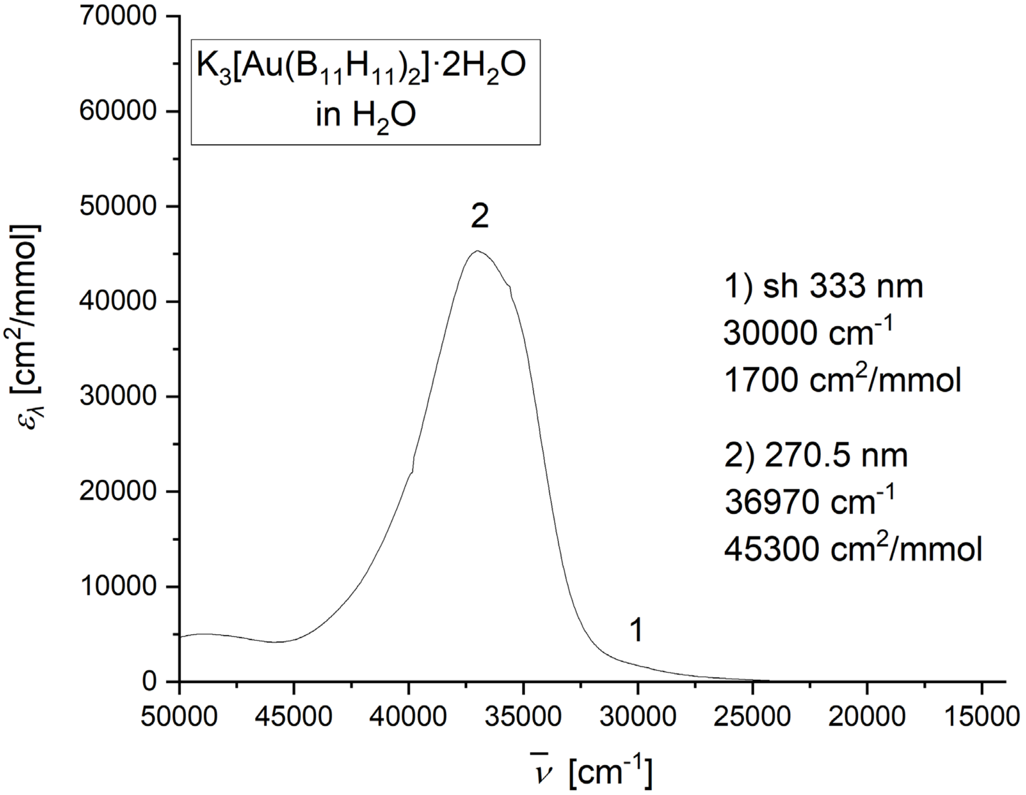

Figure S22. UV-Vis spectrum of $K_3[Au(B_{11}H_{11})_2]$ in a water solution.

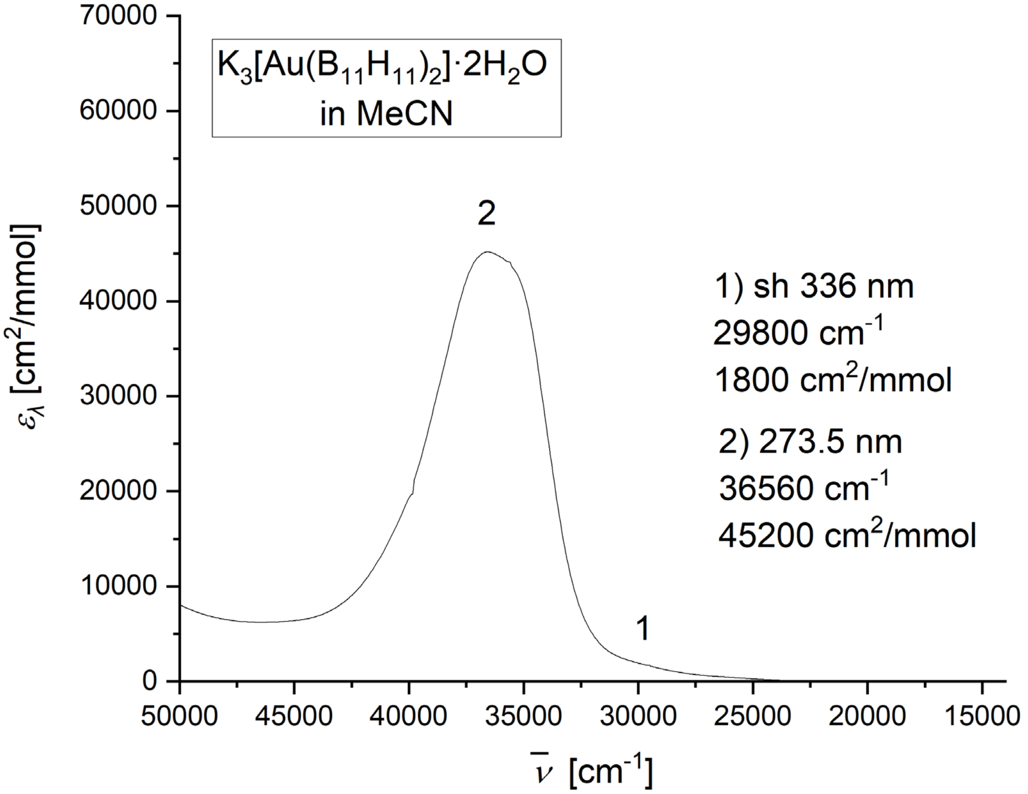

Figure S23. UV-Vis spectrum of $K_3[Au(B_{11}H_{11})_2]$ in an acetonitrile solution.

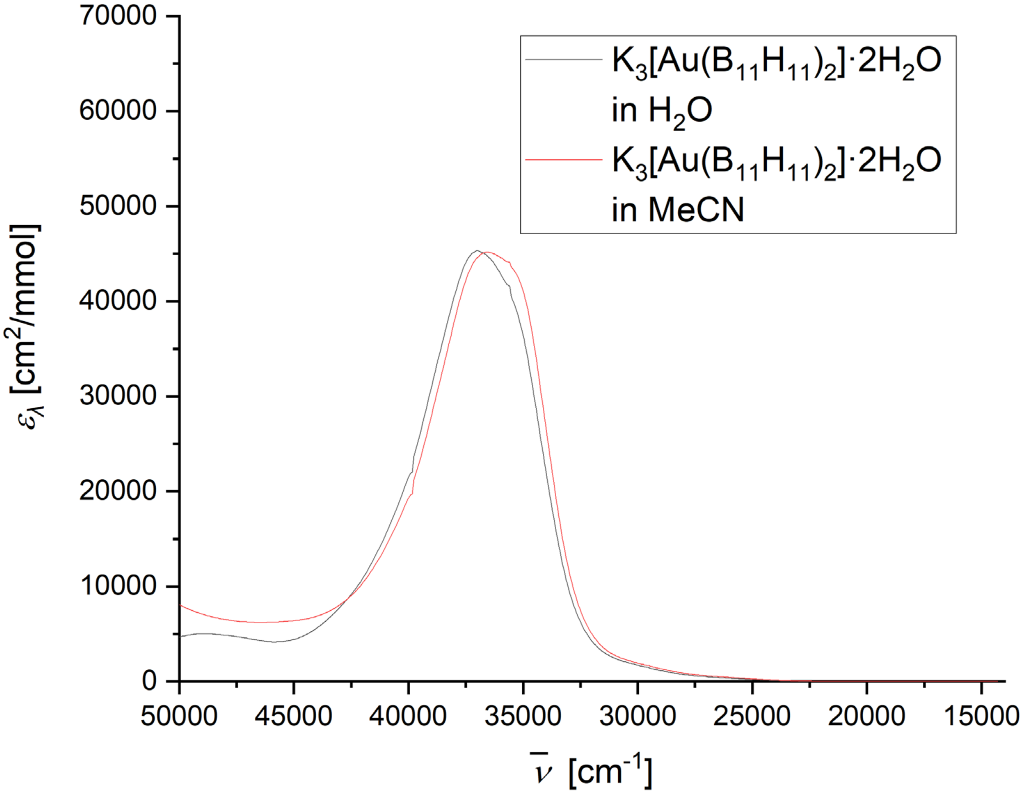

Figure S24. UV-Vis spectra of $K_3[Au(B_{11}H_{11})_2]$

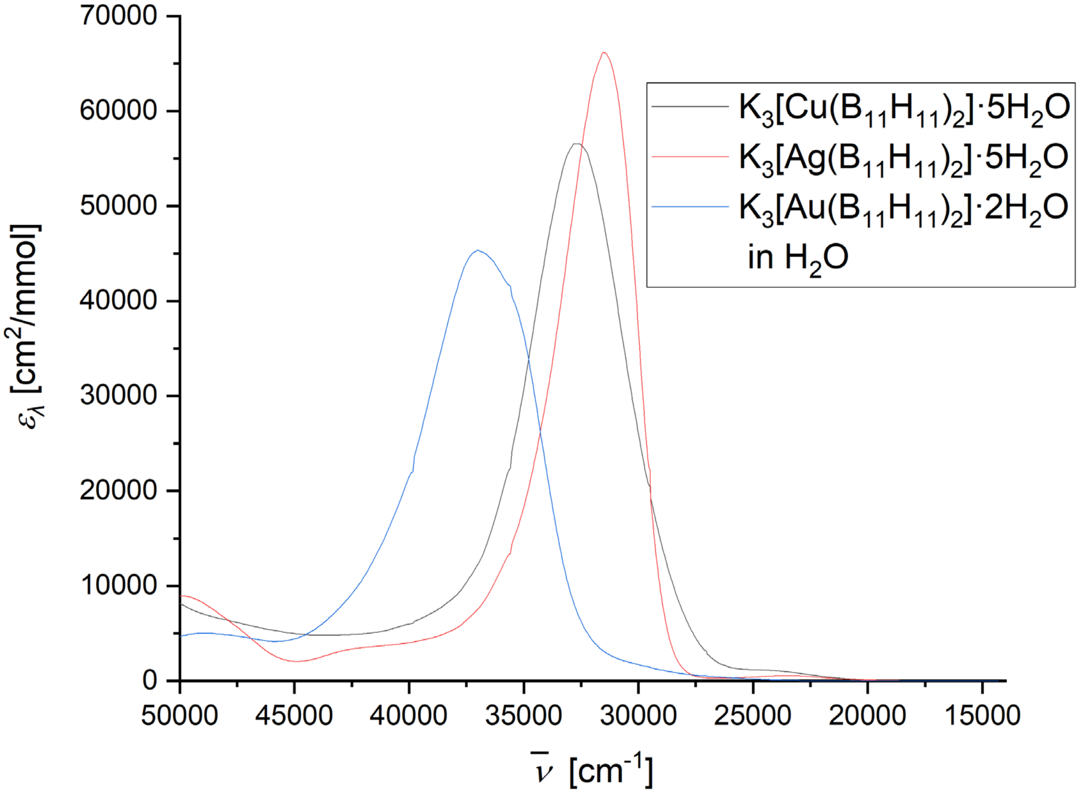

Figure S25. UV-Vis spectra of $K_3[M(B_{11}H_{11})_2]$ (M = Cu, Ag, Au) in a water solution.

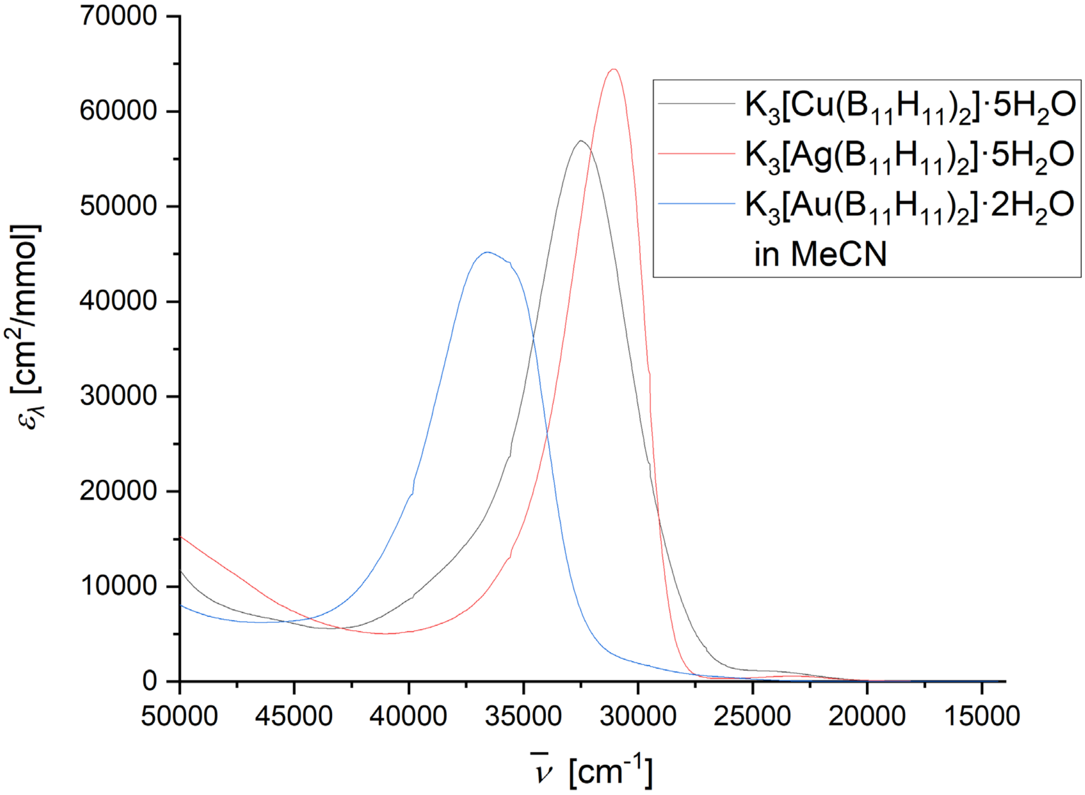

Figure S26. UV-Vis spectra of $K_3[M(B_{11}H_{11})_2]$ (M = Cu, Ag, Au) in an acetonitrile solution.

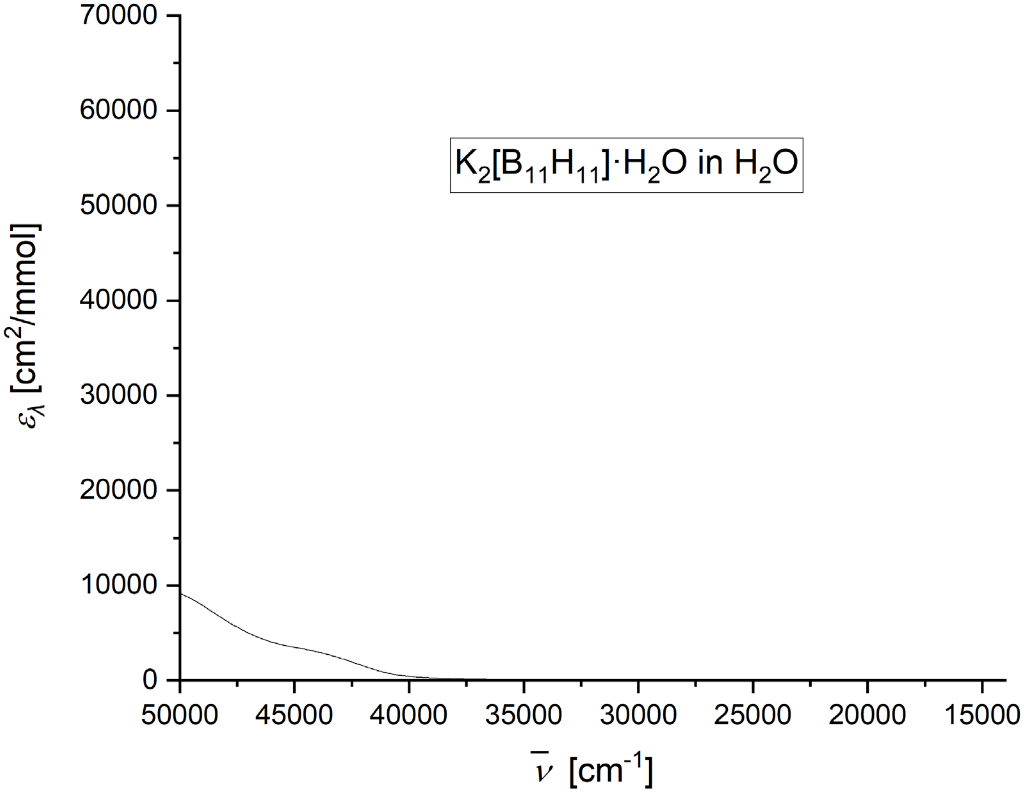

Figure S27. UV-Vis spectrum of $K_2[B_{11}H_{11}]\cdot H_2O$ in a water solution.

Computational details.

Density functional calculations (DFT)[27] were carried out using Becke's three-parameter hybrid functional and the Lee-Yang-Parr correlation functional (B3LYP)[28–30] using the Gaussian03 and Gaussian16 program suite. GaussView 4.1.2 was used for construction and visualization. Geometries were optimized, and energies were calculated with the 6-311++G(d,p),[31,32] SDD,[33–35] aug-cc-pvdz,[36,37] aug-cc-pvtz,[36,37] aug-cc-pvdz-PP[38] and aug-cc-pvtz-PP[38] basis sets. Diffuse functions were incorporated because improved energies are obtained for anions. Structures represent true minima with no imaginary frequency on the respective hypersurface.

Gaussian 03, Revision D.01,

M. J. Frisch, G. W. Trucks, H. B. Schlegel, G. E. Scuseria, M. A. Robb, J. R. Cheeseman, J. A. Montgomery, Jr., T. Vreven, K. N. Kudin, J. C. Burant, J. M. Millam, S. S. Iyengar, J. Tomasi, V. Barone, B. Mennucci, M. Cossi, G. Scalmani, N. Rega, G. A. Petersson, H. Nakatsuji, M. Hada, M. Ehara, K. Toyota, R. Fukuda, J. Hasegawa, M. Ishida, T. Nakajima, Y. Honda, O. Kitao, H. Nakai, M. Klene, X. Li, J. E. Knox, H. P. Hratchian, J. B. Cross, V. Bakken, C. Adamo, J. Jaramillo, R. Gomperts, R. E. Stratmann, O. Yazyev, A. J. Austin, R. Cammi, C. Pomelli, J. W. Ochterski, P. Y. Ayala, K. Morokuma, G. A. Voth, P. Salvador, J. J. Dannenberg, V. G. Zakrzewski, S. Dapprich, A. D. Daniels, M. C. Strain, O. Farkas, D. K. Malick, A. D. Rabuck, K. Raghavachari, J. B. Foresman, J. V. Ortiz, Q. Cui, A. G. Baboul, S. Clifford, J. Cioslowski, B. B. Stefanov, G. Liu, A. Liashenko, P. Piskorz, I. Komaromi, R. L. Martin, D. J. Fox, T. Keith, M. A. Al-Laham, C. Y. Peng, A. Nanayakkara, M. Challacombe, P. M. W. Gill, B. Johnson, W. Chen, M. W. Wong, C. Gonzalez, and J. A. Pople, Gaussian, Inc., Wallingford CT, 2004.

Gaussian 16, Revision A.03,

M. J. Frisch, G. W. Trucks, H. B. Schlegel, G. E. Scuseria, M. A. Robb, J. R. Cheeseman, G. Scalmani, V. Barone, G. A. Petersson, H. Nakatsuji, X. Li, M. Caricato, A. V. Marenich, J. Bloino, B. G. Janesko, R. Gomperts, B. Mennucci, H. P. Hratchian, J. V. Ortiz, A. F. Izmaylov, J. L. Sonnenberg, D. Williams-Young, F. Ding, F. Lipparini, F. Egidi, J. Goings, B. Peng, A. Petrone, T. Henderson, D. Ranasinghe, V. G. Zakrzewski, J. Gao, N. Rega, G. Zheng, W. Liang, M. Hada, M. Ehara, K. Toyota, R. Fukuda, J. Hasegawa, M. Ishida, T. Nakajima, Y. Honda, O. Kitao, H. Nakai, T. Vreven, K. Throssell, J. A. Montgomery, Jr., J. E. Peralta, F. Ogliaro, M. J. Bearpark, J. J. Heyd, E. N. Brothers, K. N. Kudin, V. N. Staroverov, T. A. Keith, R. Kobayashi, J. Normand, K. Raghavachari, A. P. Rendell, J. C. Burant, S. S. Iyengar, J. Tomasi, M. Cossi, J. M. Millam, M. Klene, C. Adamo, R. Cammi, J. W. Ochterski, R. L. Martin, K. Morokuma, O. Farkas, J. B. Foresman, and D. J. Fox, Gaussian, Inc., Wallingford CT, 2016.

GaussView Version 4.1.2,

Roy Dennington, Todd Keith, and John Millam, Semichem, Inc., Shawnee Mission, KS, 2007.
Web: www.gaussian.com, Gaussian, Inc.; 340 Quinnipiac St Bldg 40 Wallingford, CT o6492 USA
https://gaussview.software.informer.com/4.1/

Table S5. Orbital energies (eV) of the valence orbitals in Cu, Ag, and Au (B3LYP, energies of the α and β electrons are averaged). Basis set: SDD/aug-cc-pvdz-PP.

Orbitals	Cu, n = 4	Ag, n = 5	Au, n = 6	Cu^+, n = 4	Ag^+, n = 5	Au^+, n = 6
Δ(n−1)d/ns	−2.353/<br>−2.283	−4.404/<br>−4.372	−2.590/<br>−2.553	−4.606/<br>−4.547	−6.458/<br>−6.465	−4.416/<br>−4.420
(n−1)d	−6.828/<br>−6.767	−8.815/<br>−8.800	−8.206/<br>−8.178	−15.727/<br>−15.636	−17.143/<br>−17.104	−16.742/<br>−16.672
ns	−4.475/<br>−4.484	−4.411/<br>−4.428	−5.616/<br>−5.625	−11.121/<br>−11.089	−10.685/<br>−10.639	−12.326/<br>−12.252
np	−0.262/<br>−0.354	−0.403/<br>−0.453	−0.433/<br>−0.487	−5.965/<br>−6.061	−5.851/<br>−5.919	−6.217/<br>−6.254

Table S5a. Relative energy (ΔG_{298}, kJ/mol) of the isomer with oxidation number +I (d^{10} complex) compared to the isomer with oxidation number +V (d^6 complex) for $[M(B_{11}X_{11})_2]^{3-}$ (M = Cu, Ag, Au; X = H, F).

Functional	Basis set	Cu, H	Ag, H	Au, H	Cu, F	Ag, F	Au, F
B3LYP	B, H, F: 6-311++g(d,p)<br>M: SDD	−86.5	−122.6	48.6	68.8	−2.7	124.2
B3LYP	B, H, F: aug-cc-pvdz<br>M: aug-cc-pvdz-pp	−52.8	−93.3	85.7	95.6	21.7	145.5
B3LYP	B, H, F: aug-cc-pvtz<br>M: aug-cc-pvtz-pp	−69.8	−106.5	76.0	78.5	11.3	129.4
B3LYP, SCRF (Solvent=Water)	B, H, F: 6-311++g(d,p)<br>M: SDD	5.5	−33.9	122.4	118.3	47.8	(178)[a]
B3LYP, SCRF (Solvent=Water)	B, H, F: aug-cc-pvdz<br>M: aug-cc-pvdz-pp	36.9	−1.4	154.5	145.0	75.3	(208)[a]
B3LYP, SCRF (Solvent=Water)	B, H, F: aug-cc-pvtz<br>M: aug-cc-pvtz-pp	25.5	−9.6	146.6	130.3	64.2	(191)[a]

[a] Optimization of $[Au^{+I}(\textit{closo}\text{-}B_{11}H_{11})_2]^{3-}$ in this case resulted in $[Au^{+V}(\textit{nido}\text{-}B_{11}H_{11})_2]^{3-}$. The given values are for the C_{2h} rotamer, which has an imagined frequency.

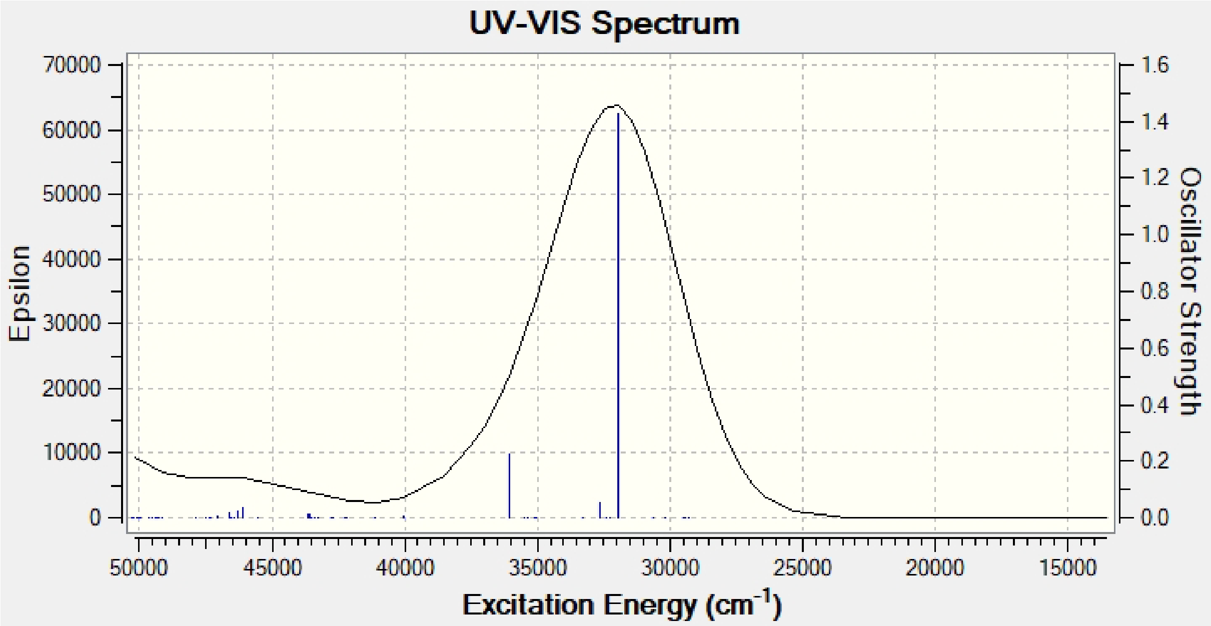

Figure S28. UV-Vis spectrum of $[Cu^{+V}(nido\text{-}B_{11}H_{11})_2]^{3-}$, B3LYP/aug-cc-pvdz (B, H), /aug-cc-pvdz-PP (Cu); SCRF(Solvent=Water), TD(NStates=400); UV-Vis peak half-width at half height 0.333 eV (2685.83 cm^{-1})

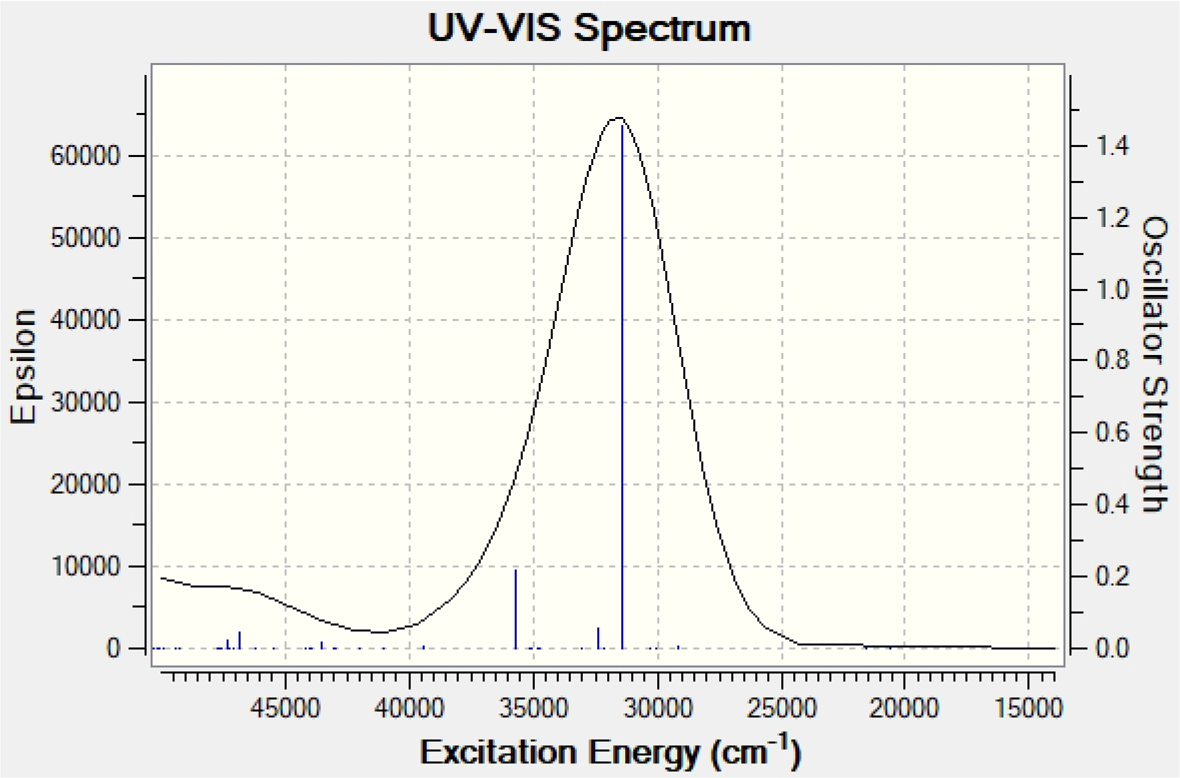

Figure S29. UV-Vis spectrum of $[Cu^{+V}(nido\text{-}B_{11}H_{11})_2]^{3-}$, B3LYP/6-311++g(d,p) (B, H), /SDD (Cu); SCRF(Solvent=Water), TD(NStates=400); UV-Vis peak half-width at half height 0.333 eV (2685.83 cm^{-1})

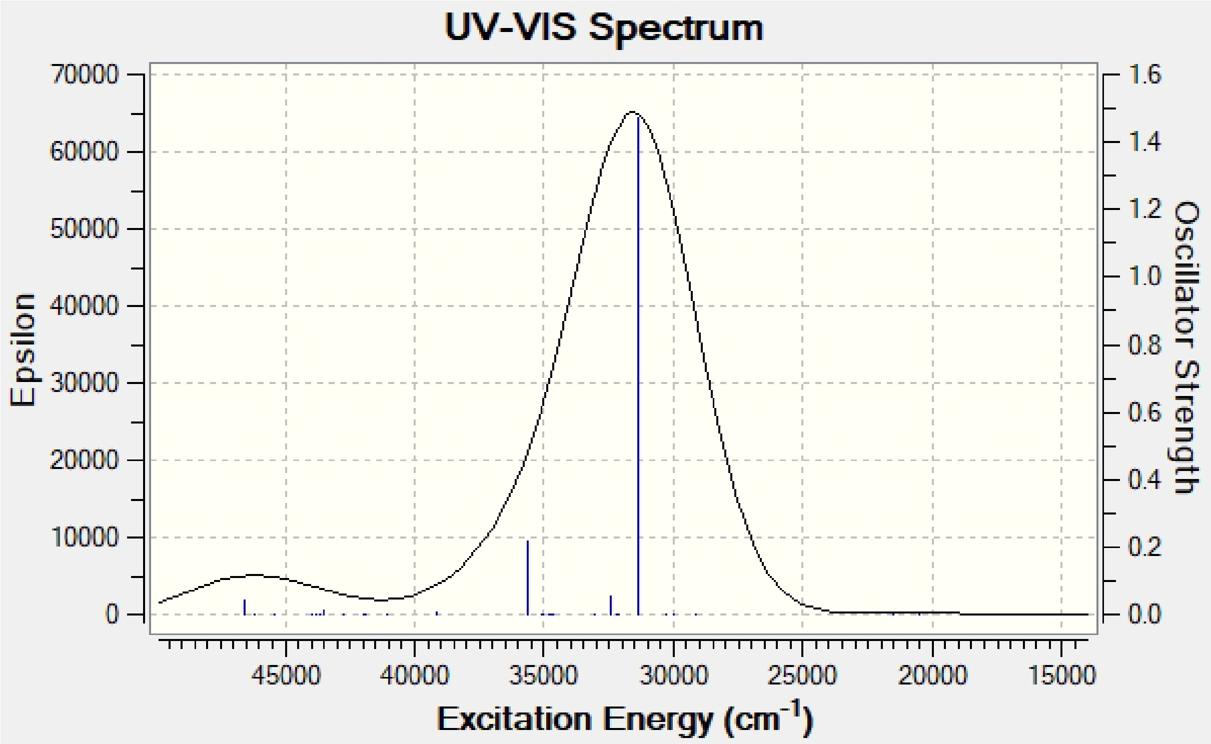

Figure S30. UV-Vis spectrum of $[Cu^{+V}(nido\text{-}B_{11}H_{11})_2]^{3-}$, B3LYP/6-311++g(d,p) (B, H), /SDD (Cu); SCRF(Solvent=CH3CN), TD(NStates=50); UV-Vis peak half-width at half height 0.333 eV (2685.83 cm^{-1})

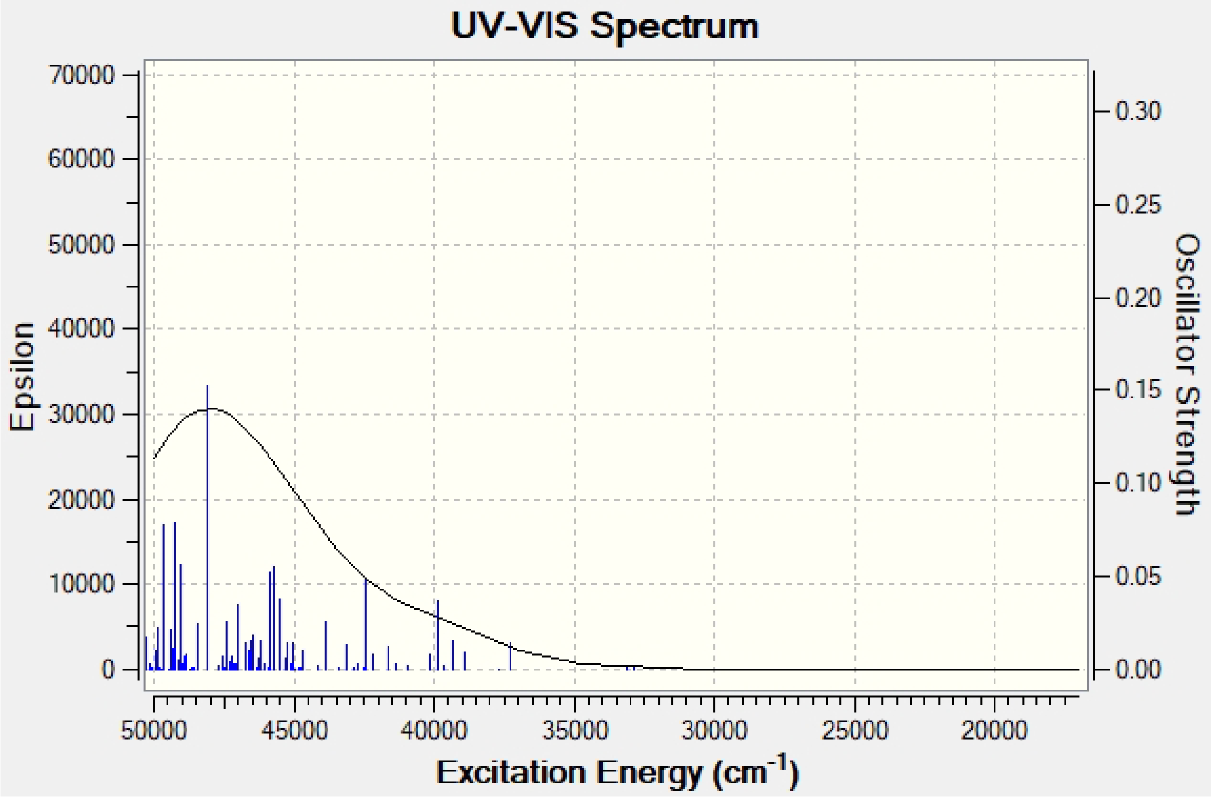

Figure S31. UV-Vis spectrum of $[Cu^{+I}(closo\text{-}B_{11}H_{11})_2]^{3-}$, B3LYP/6-311++g(d,p) (B, H), /SDD (Cu); SCRF(Solvent=Water), TD(NStates=100); UV-Vis peak half-width at half height 0.333 eV (2685.83 cm^{-1})

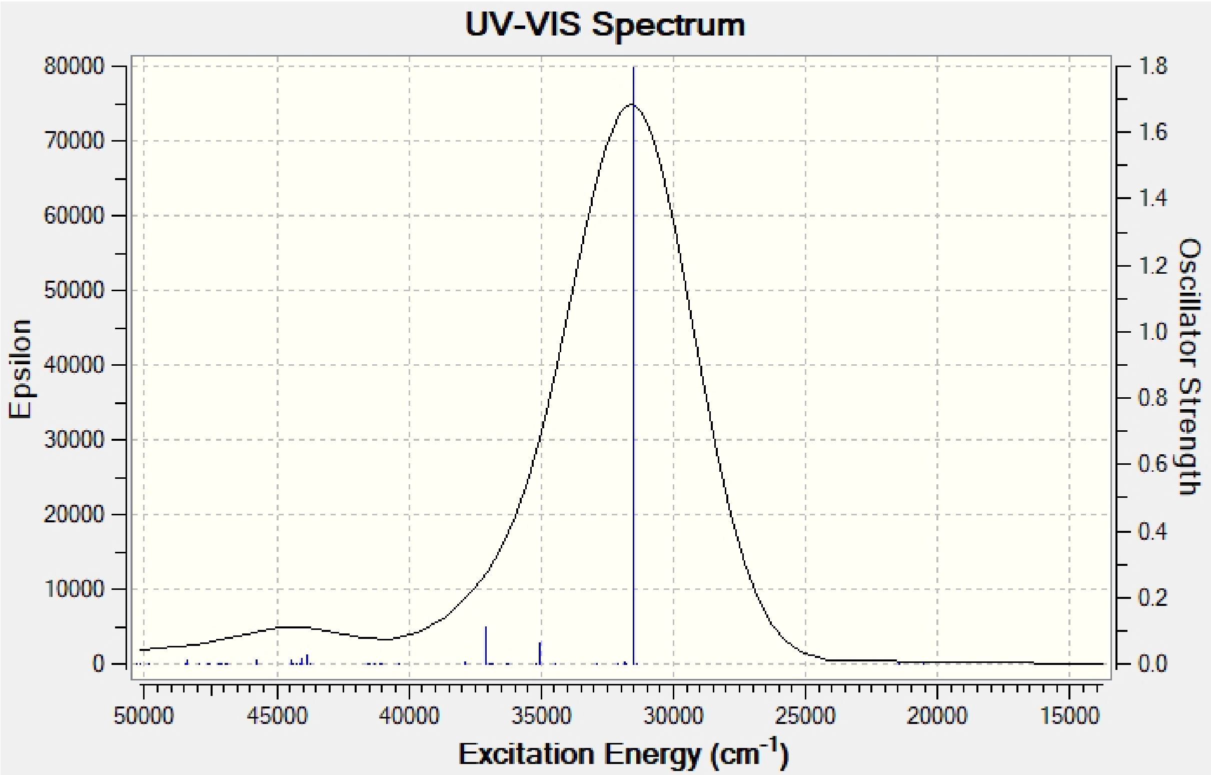

Figure S32. UV-Vis spectrum of $[Ag^{+V}(nido\text{-}B_{11}H_{11})_2]^{3-}$, B3LYP/aug-cc-pvdz (B, H), /aug-cc-pvdz-PP (Ag); SCRF(Solvent=Water), TD(NStates=100); UV-Vis peak half-width at half height 0.333 eV (2685.83 cm^{-1})

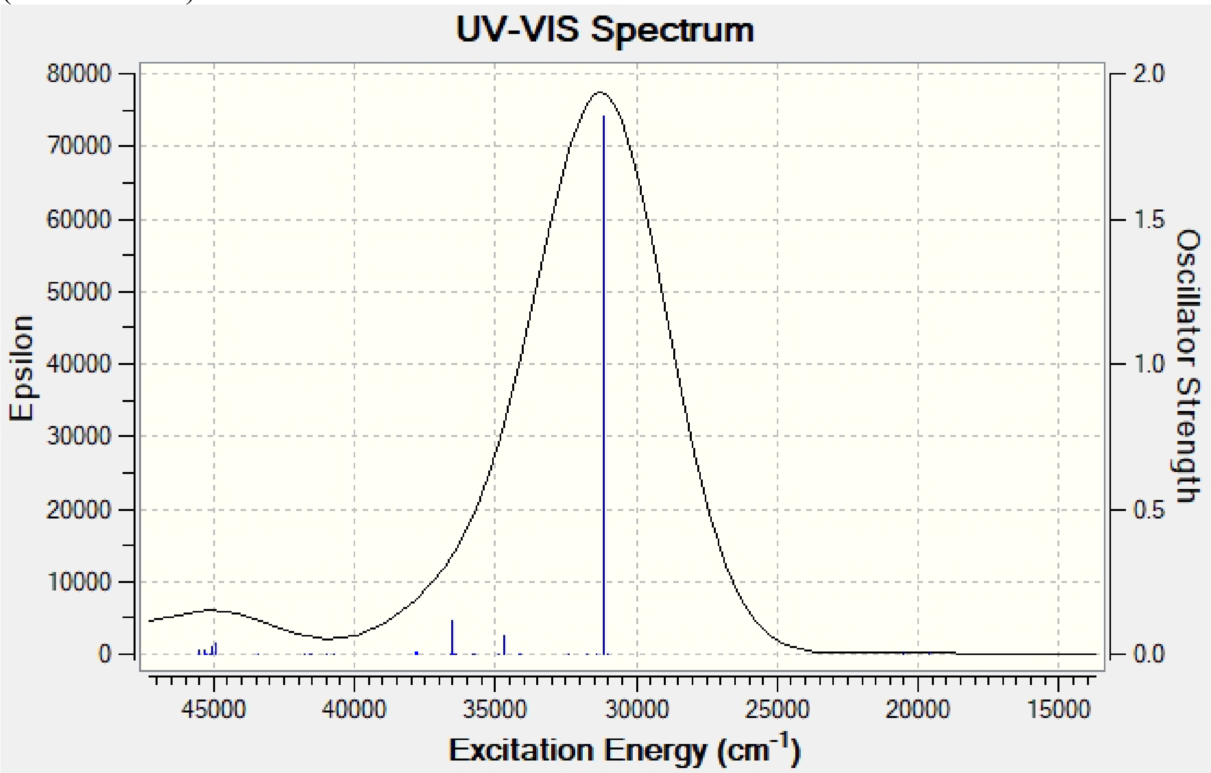

Figure S33. UV-Vis spectrum of $[Ag^{+V}(nido\text{-}B_{11}H_{11})_2]^{3-}$, B3LYP/6-311++g(d,p) (B, H), /SDD (Ag); SCRF(Solvent=Water), TD(NStates=400); UV-Vis peak half-width at half height 0.333 eV (2685.83 cm^{-1})

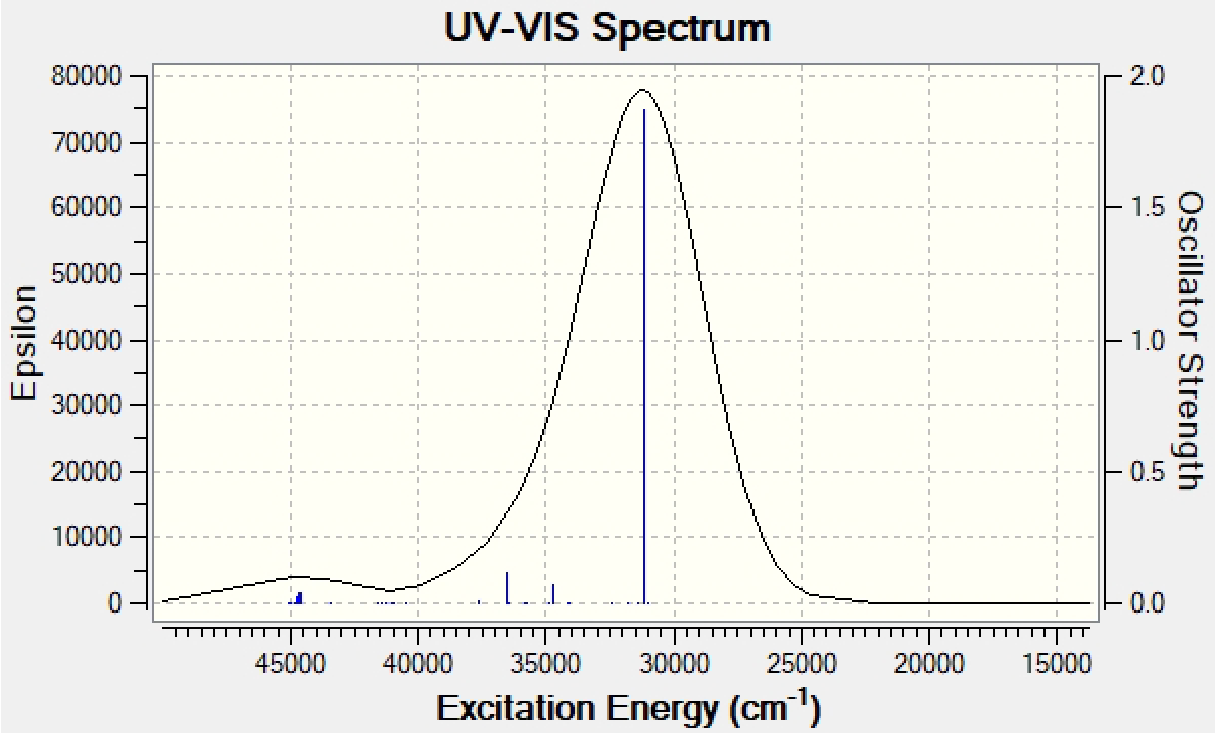

Figure S34. UV-Vis spectrum of $[Ag^{+V}(\textit{nido}\text{-}B_{11}H_{11})_2]^{3-}$, B3LYP/6-311++g(d,p) (B, H), /SDD (Ag); SCRF(Solvent=CH3CN), TD(NStates=50); UV-Vis peak half-width at half height 0.333 eV (2685.83 cm^{-1})

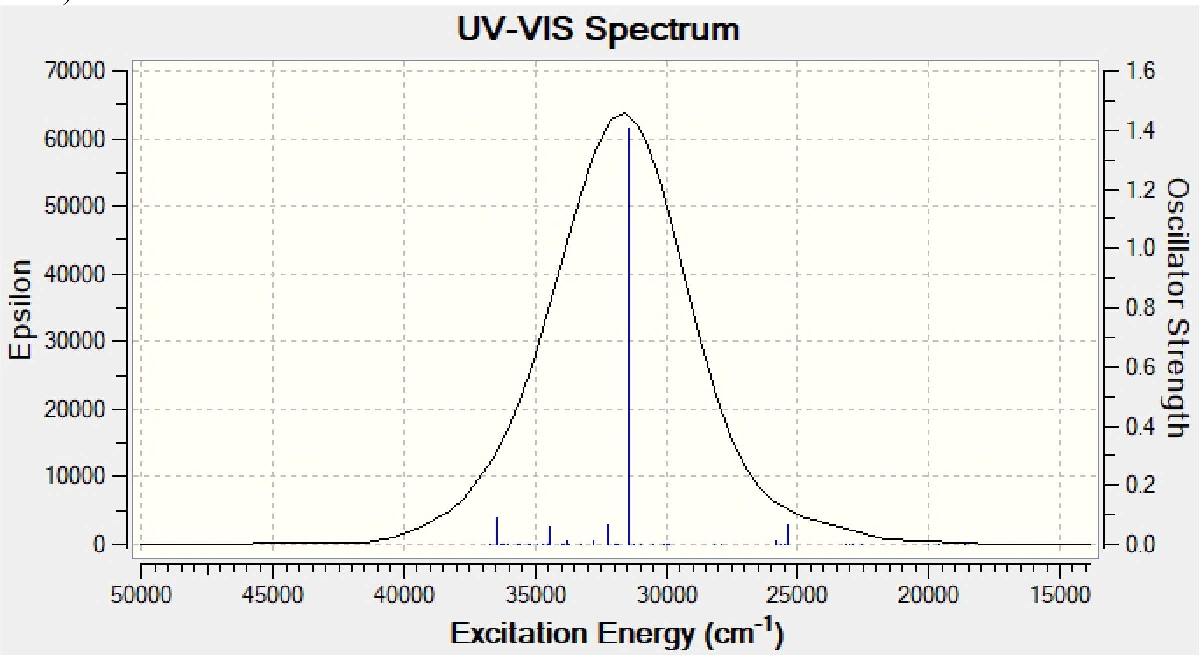

Figure S35. UV-Vis spectrum of $[Ag^{+V}(\textit{nido}\text{-}B_{11}H_{11})_2]^{3-}$, B3LYP/6-311++g(d,p) (B, H), /SDD (Ag); TD(NStates=100); UV-Vis peak half-width at half height 0.333 eV (2685.83 cm^{-1})

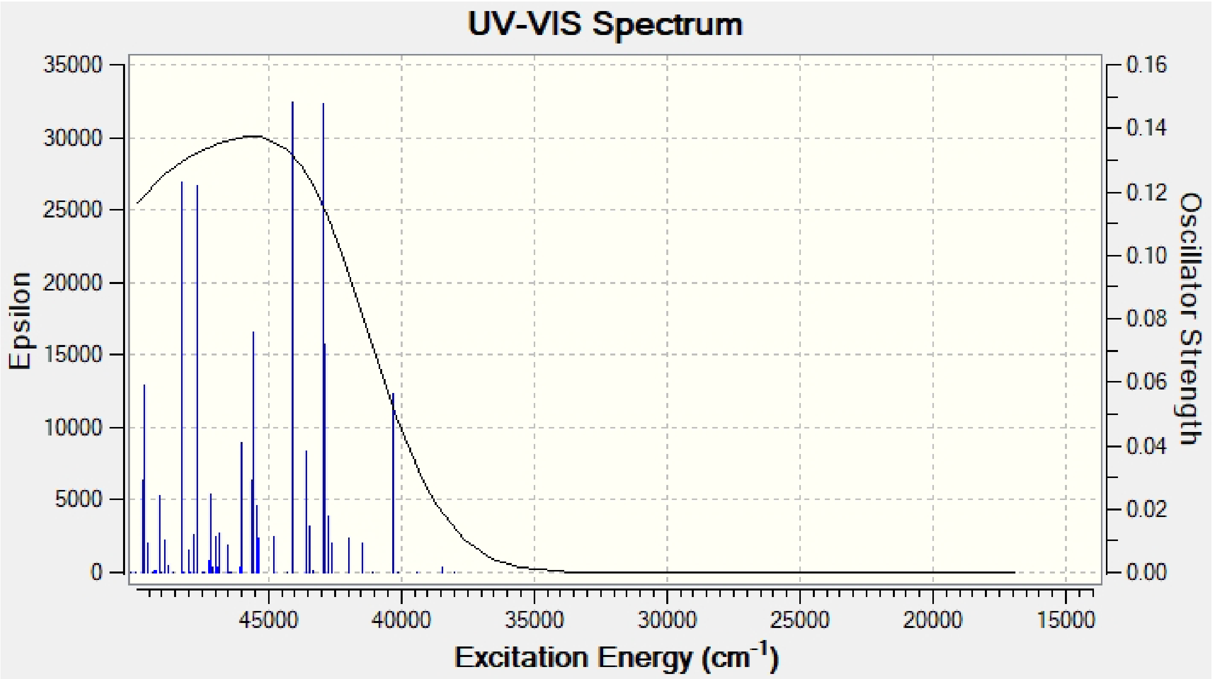

Figure S36. UV-Vis spectrum of $[Ag^{+I}(\textit{closo}\text{-}B_{11}H_{11})_2]^{3-}$ (C_{2v} rotamere), B3LYP/6-311++g(d,p) (B, H), /SDD (Ag); SCRF(Solvent=Water), TD(NStates=100); UV-Vis peak half-width at half height 0.333 eV (2685.83 cm^{-1})

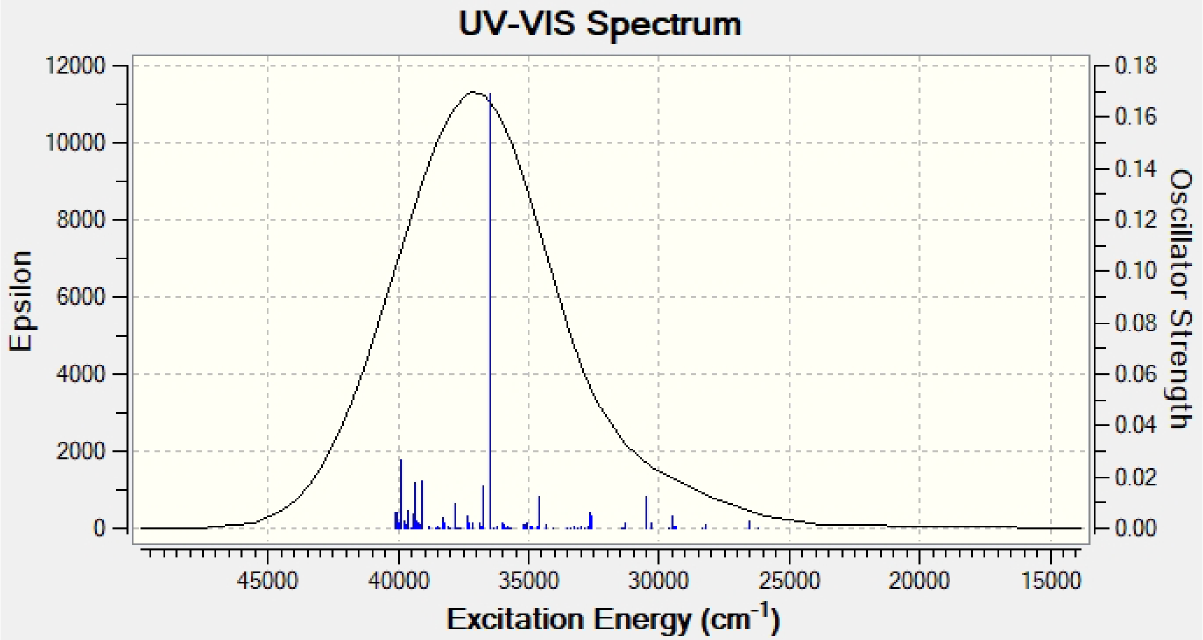

Figure S37. UV-Vis spectrum of $[Ag^{+I}(\textit{closo}\text{-}B_{11}H_{11})_2]^{3-}$ (C_{2v} rotamere), B3LYP/6-311++g(d,p) (B, H), /SDD (Ag); TD(NStates=100); UV-Vis peak half-width at half height 0.333 eV (2685.83 cm^{-1})

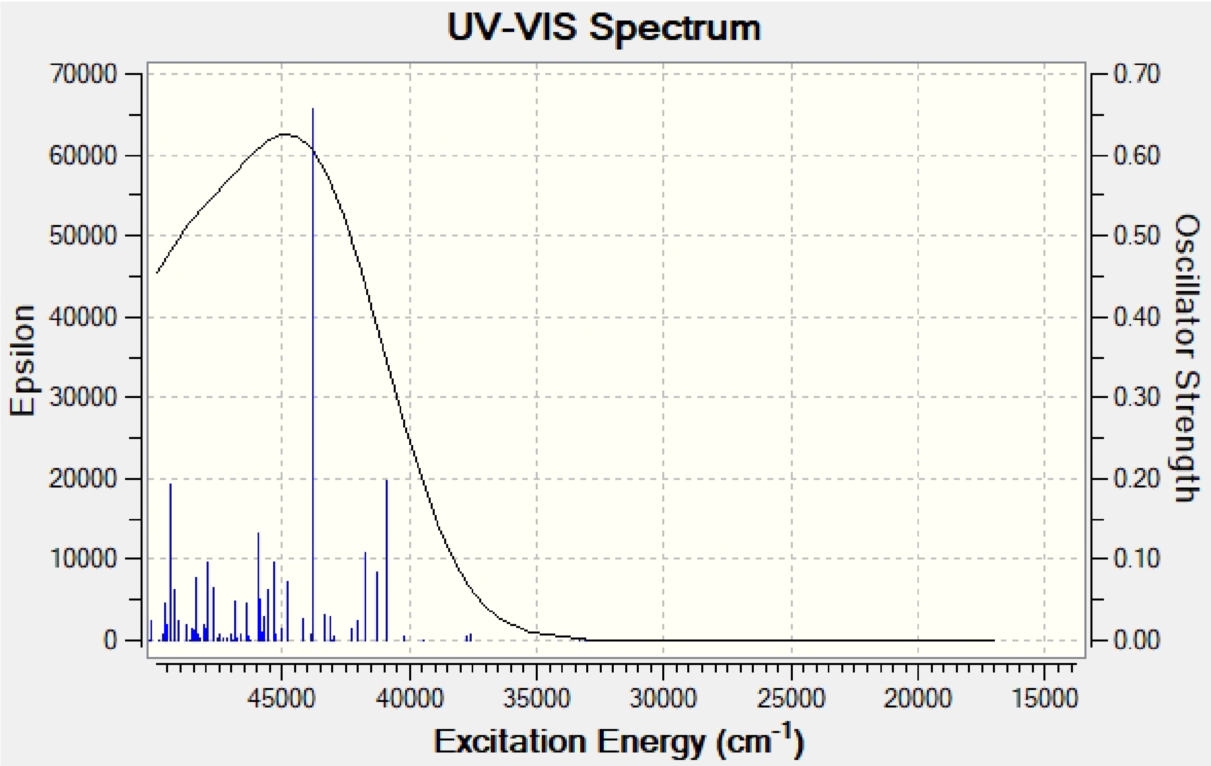

Figure S38. UV-Vis spectrum of $[Ag^{+I}(closo\text{-}B_{11}H_{11})_2]^{3-}$, B3LYP/6-311++g(d,p) (B, H), /SDD (Ag); SCRF(Solvent=Water), TD(NStates=100); UV-Vis peak half-width at half height 0.333 eV (2685.83 cm^{-1})

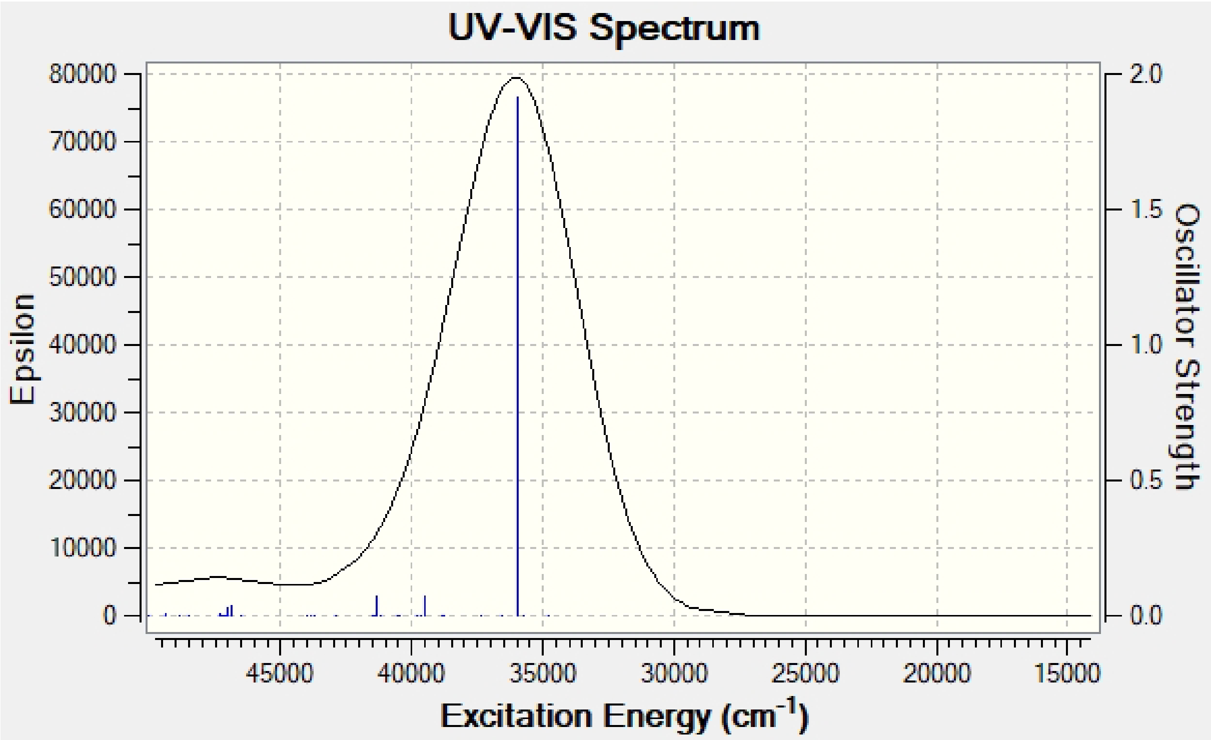

Figure S39. UV-Vis spectrum of $[Au^{+V}(nido\text{-}B_{11}H_{11})_2]^{3-}$, B3LYP/aug-cc-pvdz (B, H), /aug-cc-pvdz-PP (Au); SCRF(Solvent=Water), TD(NStates=100); UV-Vis peak half-width at half height 0.333 eV (2685.83 cm^{-1})

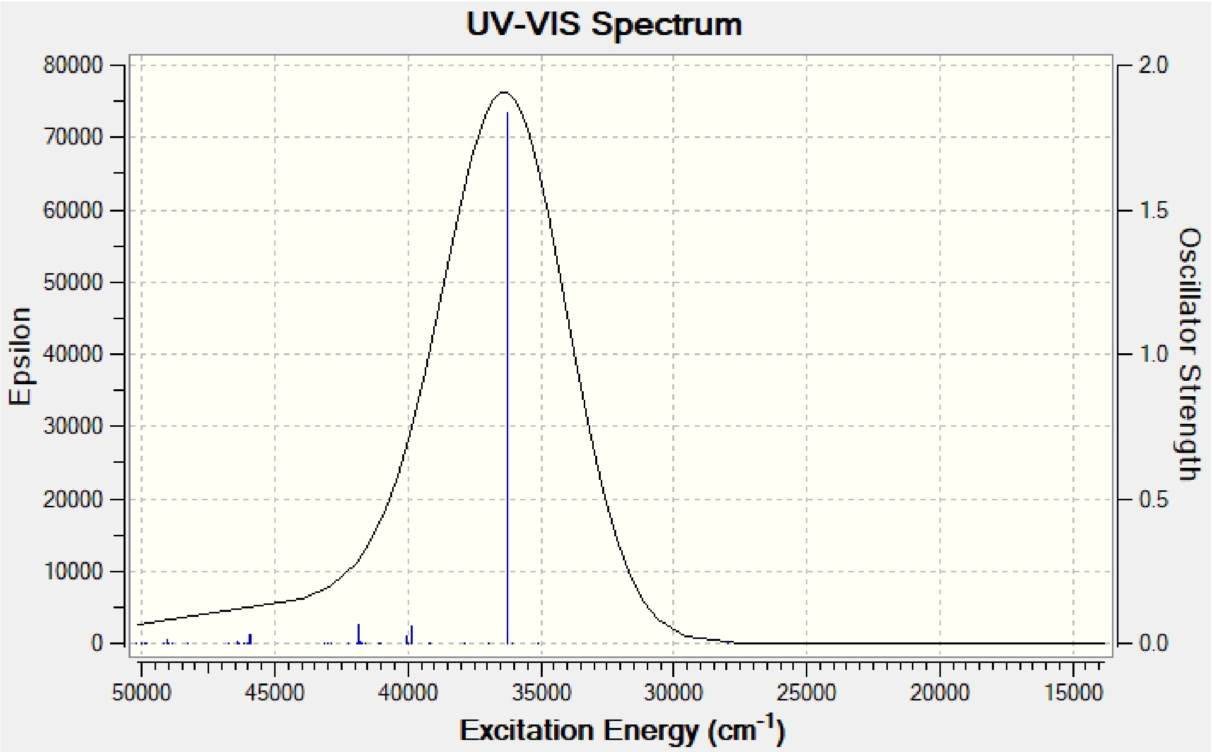

Figure S40. UV-Vis spectrum of $[Au^{+V}(\textit{nido}-B_{11}H_{11})_2]^{3-}$, B3LYP/6-311++g(d,p) (B, H), /SDD (Au); SCRF(Solvent=Water), TD(NStates=400); UV-Vis peak half-width at half height 0.333 eV (2685.83 cm^{-1})

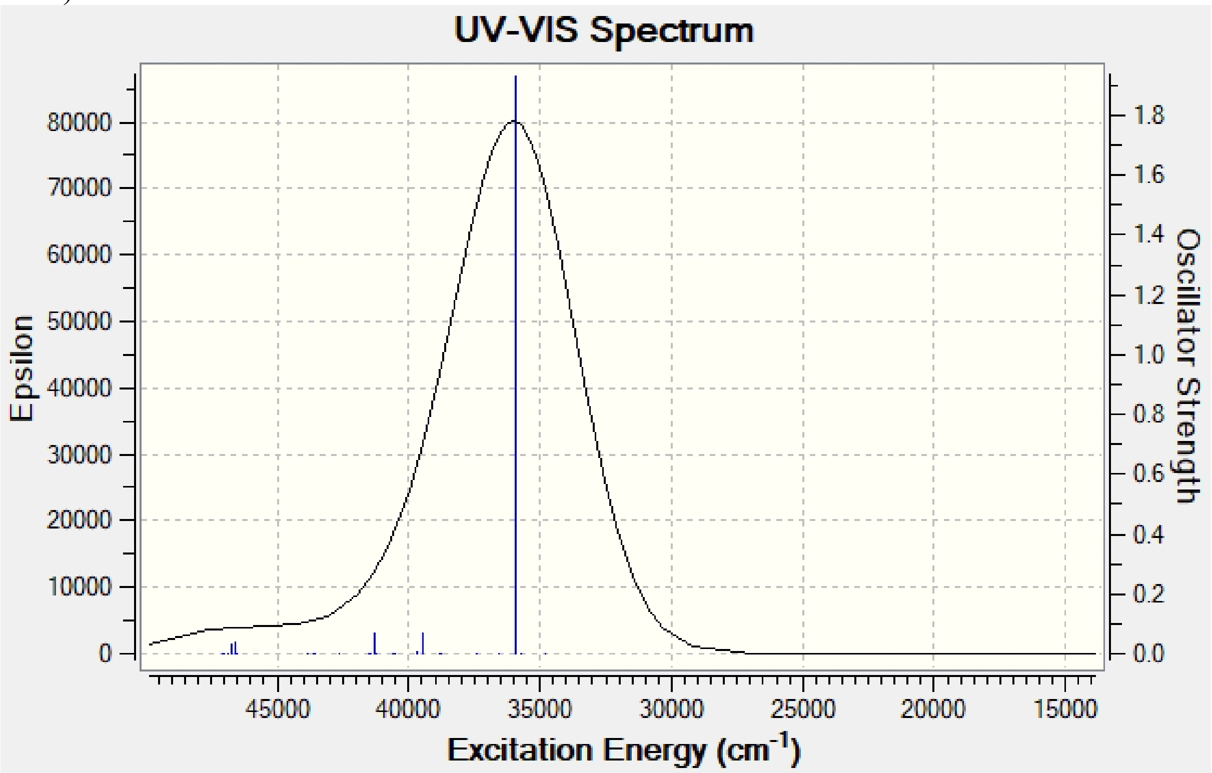

Figure S41. UV-Vis spectrum of $[Au^{+V}(\textit{nido}-B_{11}H_{11})_2]^{3-}$, B3LYP/6-311++g(d,p) (B, H), /SDD (Au); SCRF(Solvent=CH3CN), TD(NStates=50); UV-Vis peak half-width at half height 0.333 eV (2685.83 cm^{-1})

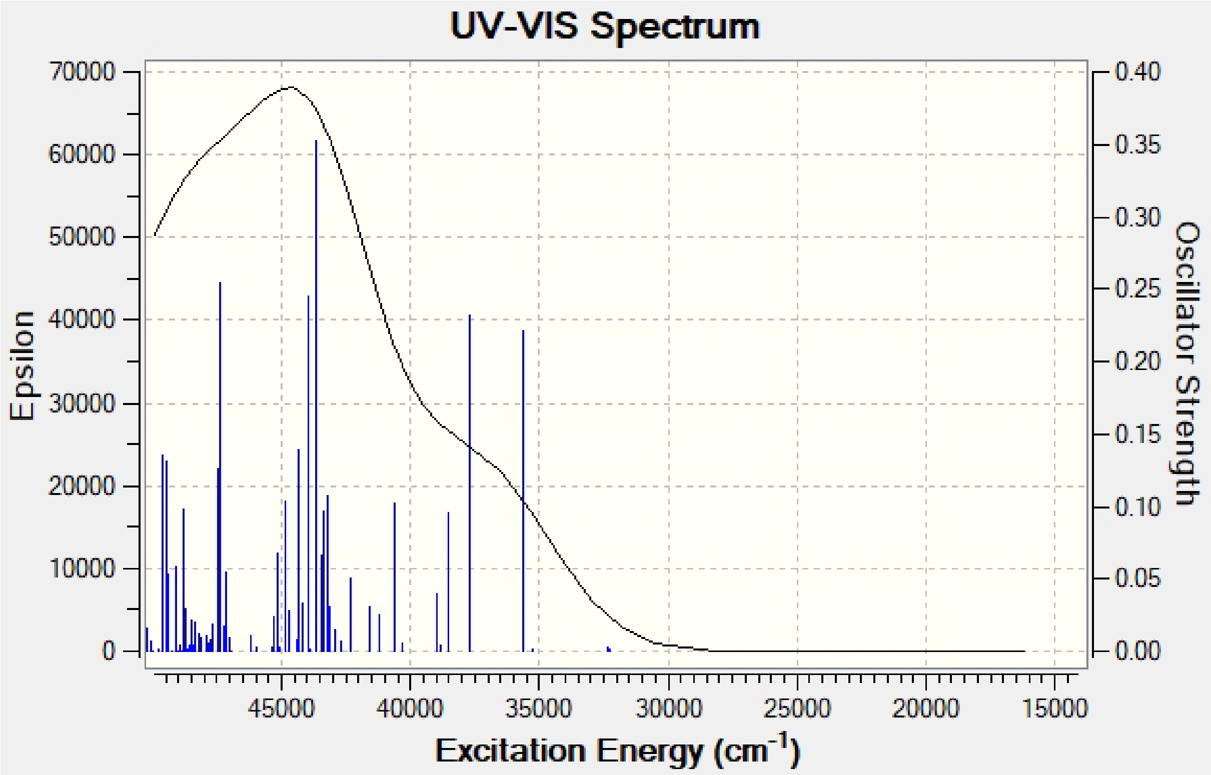

Figure S42. UV-Vis spectrum of $[Au^{+I}(closo\text{-}B_{11}H_{11})_2]^{3-}$, B3LYP/6-311++g(d,p) (B, H), /SDD (Au); SCRF(Solvent=Water), TD(NStates=100); UV-Vis peak half-width at half height 0.333 eV (2685.83 cm^{-1})

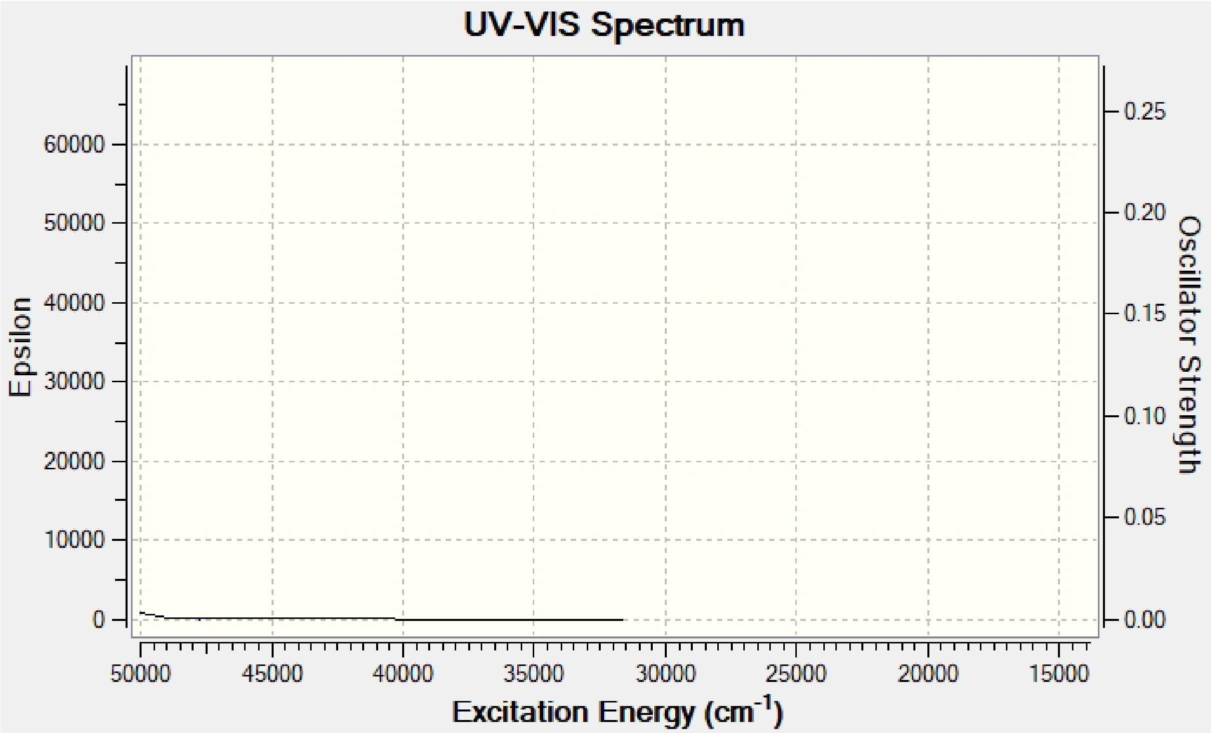

Figure S43. UV-Vis spectrum of *closo*-$[B_{12}H_{12}]^{2-}$, B3LYP/6-311++g(d,p); SCRF(Solvent=Water), TD(NStates=500); UV-Vis peak half-width at half height 0.333 eV (2685.83 cm^{-1})

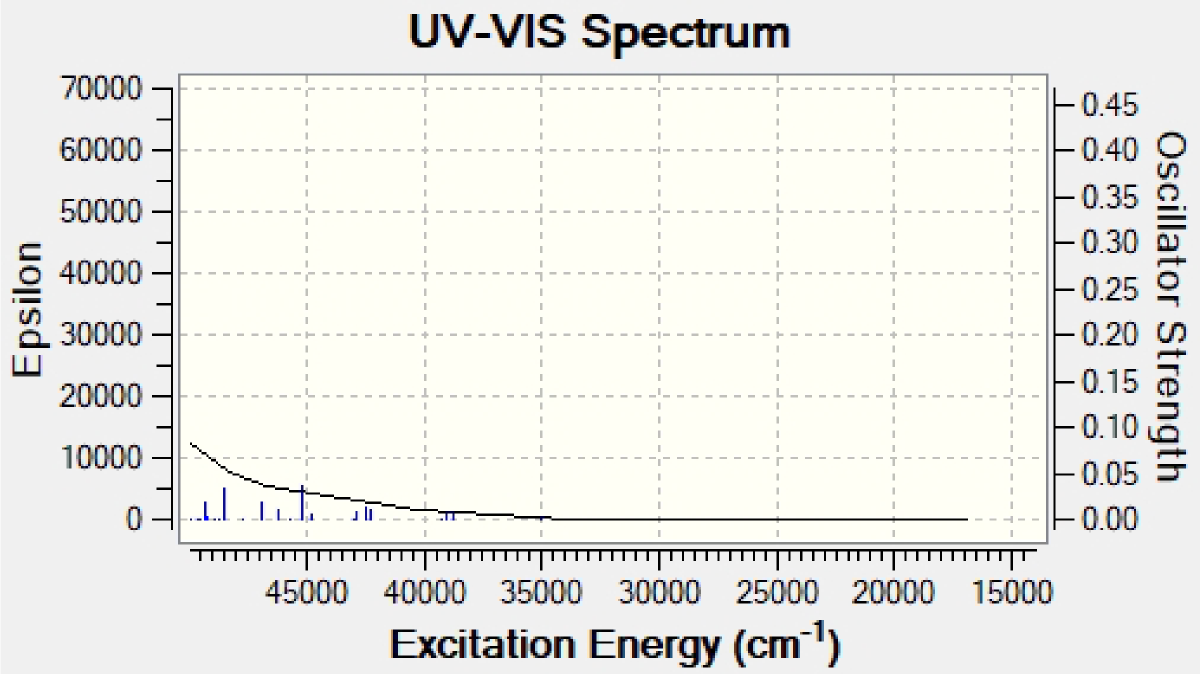

Figure S44. UV-Vis spectrum of *closo*-$[B_{11}H_{11}]^{2-}$, B3LYP/6-311++g(d,p); SCRF(Solvent=Water), TD(NStates=100); UV-Vis peak half-width at half height 0.333 eV (2685.83 cm^{-1})

Table S6. Calculated transitions in $[Cu^{+V}(nido\text{-}B_{11}H_{11})_2]^{3-}$, B3LYP/aug-cc-pvdz (B, H), /aug-cc-pvdz-PP (Cu); SCRF(Solvent=Water); TD(NStates=400); (HOMO-77; LUMO-78)

excited State Nr	excited State	symmery		ΔS	excitation energie, eV	excitation energie, nm	excitation energie, cm^{-1}	oscillator strength
1	76 → 78<br>77 → 79	$E_{1u} \rightarrow E_{1g}$<br>$E_{1u} \rightarrow E_{1g}$	A_{2u}[a)]	1	2.2697	546.25	18306	0.0000
2,3	76 → 78,79<br>77 → 79,78	$E_{1u} \rightarrow E_{1g}$<br>$E_{1u} \rightarrow E_{1g}$	E_{2u}[a)]	1	2.4345	509.28	19635	0.0000*2
4	76 → 79<br>77 → 78	$E_{1u} \rightarrow E_{1g}$<br>$E_{1u} \rightarrow E_{1g}$	A_{1u}[a)]	1	2.6744	463.60	21570	0.0000
1	76 → 79<br>77 → 78	$E_{1u} \rightarrow E_{1g}$<br>$E_{1u} \rightarrow E_{1g}$	A_{1u}[a)]	0	2.6798	462.66	21614	0.0000
2,3	76 → 78,79<br>77 → 79,78	$E_{1u} \rightarrow E_{1g}$<br>$E_{1u} \rightarrow E_{1g}$	E_{2u}[a)]	0	2.7939	443.77	22534	0.0000*2
6,7	71 → 79,78<br>72 → 78,79<br>75 → 78,79	$E_{2u} \rightarrow E_{1g}$<br>$E_{2u} \rightarrow E_{1g}$<br>$A_{2u} \rightarrow E_{1g}$	E_{1u}	0	3.6517	339.52	29453	0.0024*2
12	76 → 78<br>77 → 79	$E_{1u} \rightarrow E_{1g}$<br>$E_{1u} \rightarrow E_{1g}$	A_{2u}	0	3.9608	313.03	31946	1.4269
16,17	71 → 79,78<br>72 → 78,79<br>75 → 78,79	$E_{2u} \rightarrow E_{1g}$<br>$E_{2u} \rightarrow E_{1g}$<br>$A_{2u} \rightarrow E_{1g}$	E_{1u}	0	4.0475	306.33	32645	0.0516*2
30	67 → 78<br>68 → 79	$E_{1u} \rightarrow E_{1g}$<br>$E_{1u} \rightarrow E_{1g}$	A_{2u}	0	4.4665	277.59	36024	0.2226
31,32	76,77 → 80	$E_{1u} \rightarrow A_{1g}$	E_{1u}	0	4.9655	249.69	40049	0.0073*2

[a)] These transitions can gain intensity through spin-orbit coupling

Table S7. Calculated transitions in $[Cu^{+V}(nido\text{-}B_{11}H_{11})_2]^{3-}$, B3LYP/6-311++g(d,p) (B, H), /SDD (Cu); SCRF(Solvent=Water); TD(NStates=400); (HOMO-77; LUMO-78)

excited State Nr	excited State	symmery		ΔS	excitation energie, eV	excitation energie, nm	excitation energie, cm^{-1}	oscillator strength
1	76 → 78<br>77 → 79	$E_{1u} \rightarrow E_{1g}$<br>$E_{1u} \rightarrow E_{1g}$	A_{2u}[a)]	1	2.1243	583.64	17134	0.0000
2,3	76 → 78,79<br>77 → 79,78	$E_{1u} \rightarrow E_{1g}$<br>$E_{1u} \rightarrow E_{1g}$	E_{2u}[a)]	1	2.2947	540.32	18508	0.0000*2
4	76 → 79<br>77 → 78	$E_{1u} \rightarrow E_{1g}$<br>$E_{1u} \rightarrow E_{1g}$	A_{1u}[a)]	1	2.5419	487.77	20502	0.0000
1	76 → 79<br>77 → 78	$E_{1u} \rightarrow E_{1g}$<br>$E_{1u} \rightarrow E_{1g}$	A_{1u}[a)]	0	2.5498	486.25	20565	0.0000
2,3	76 → 78,79<br>77 → 79,78	$E_{1u} \rightarrow E_{1g}$<br>$E_{1u} \rightarrow E_{1g}$	E_{2u}[a)]	0	2.6709	464.21	21542	0.0000*2
4,5	71 → 79,78<br>72 → 78,79<br>75 → 78,79	$E_{2u} \rightarrow E_{1g}$<br>$E_{2u} \rightarrow E_{1g}$<br>$A_{2u} \rightarrow E_{1g}$	E_{1u}	0	3.6133	343.14	29143	0.0026*2
12	76 → 78<br>77 → 79	$E_{1u} \rightarrow E_{1g}$<br>$E_{1u} \rightarrow E_{1g}$	A_{2u}	0	3.8988	318.01	31446	1.4558
16,17	71 → 79,78<br>72 → 78,79<br>75 → 78,79	$E_{2u} \rightarrow E_{1g}$<br>$E_{2u} \rightarrow E_{1g}$<br>$A_{2u} \rightarrow E_{1g}$	E_{1u}	0	4.0197	308.44	32421	0.0525*2
30	67 → 78<br>68 → 79	$E_{1u} \rightarrow E_{1g}$<br>$E_{1u} \rightarrow E_{1g}$	A_{2u}	0	4.4216	280.41	35662	0.2173
31,32	76,77 → 80	$E_{1u} \rightarrow A_{1g}$	E_{1u}	0	4.8845	253.83	39396	0.0069*2

[a)] These transitions can gain intensity through spin-orbit coupling

Table S8. Calculated transitions in $[Ag^{+V}(\textit{nido}\text{-}B_{11}H_{11})_2]^{3-}$, B3LYP/aug-cc-pvdz (B, H), /aug-cc-pvdz-PP (Ag); SCRF(Solvent=Water); TD(NStates=100); (HOMO-77; LUMO-78)

excited State Nr	excited State	symmery		ΔS	excitation energie, eV	excitation energie, nm	excitation energie, cm^{-1}	oscillator strength
1	76 → 78<br>77 → 79	$E_{1u} \to E_{1g}$<br>$E_{1u} \to E_{1g}$	A_{2u}[a)]	1	2.1455	577.88	17305	0.0000
2,3	76 → 78,79<br>77 → 79,78	$E_{1u} \to E_{1g}$<br>$E_{1u} \to E_{1g}$	E_{2u}[a)]	1	2.3105	536.62	18635	0.0000*2
4	76 → 79<br>77 → 78	$E_{1u} \to E_{1g}$<br>$E_{1u} \to E_{1g}$	A_{1u}[a)]	1	2.5430	487.56	20511	0.0000
1	76 → 79<br>77 → 78	$E_{1u} \to E_{1g}$<br>$E_{1u} \to E_{1g}$	A_{1u}[a)]	0	2.5487	486.47	20557	0.0000
2,3	76 → 78,79<br>77 → 79,78	$E_{1u} \to E_{1g}$<br>$E_{1u} \to E_{1g}$	E_{2u}[a)]	0	2.6622	465.73	21472	0.0000*2
6	76 → 78<br>77 → 79	$E_{1u} \to E_{1g}$<br>$E_{1u} \to E_{1g}$	A_{2u}	0	3.9051	317.49	31497	1.7970
7,8	71 → 79,78<br>72 → 78,79<br>73 → 78,79	$E_{2u} \to E_{1g}$<br>$E_{2u} \to E_{1g}$<br>$A_{2u} \to E_{1g}$	E_{1u}	0	3.9444	314.34	31814	0.0025*2
16,17	71 → 79,78<br>72 → 78,79<br>73 → 78,79	$E_{2u} \to E_{1g}$<br>$E_{2u} \to E_{1g}$<br>$A_{2u} \to E_{1g}$	E_{1u}	0	4.3441	285.41	35037	0.0652*2
30	67 → 78<br>68 → 79	$E_{1u} \to E_{1g}$<br>$E_{1u} \to E_{1g}$	A_{2u}	0	4.5960	269.77	37069	0.1113
31,32	76,77 → 80	$E_{1u} \to A_{1g}$	E_{1u}	0	4.6958	264.04	37874	0.0045*2

a) These transitions can gain intensity through spin-orbit coupling

Table S9. Calculated transitions in $[Ag^{+V}(\textit{nido}\text{-}B_{11}H_{11})_2]^{3-}$, B3LYP/6-311++g(d,p) (B, H), /SDD (Ag); SCRF(Solvent=Water); TD(NStates=400); (HOMO-77; LUMO-78)

excited State Nr	excited State	symmery		ΔS	excitation energie, eV	excitation energie, nm	excitation energie, cm^{-1}	oscillator strength
1	76 → 78<br>77 → 79	$E_{1u} \to E_{1g}$<br>$E_{1u} \to E_{1g}$	A_{2u}[a)]	1	2.0130	615.91	16236	0.0000
2,3	76 → 78,79<br>77 → 79,78	$E_{1u} \to E_{1g}$<br>$E_{1u} \to E_{1g}$	E_{2u}[a)]	1	2.1834	567.85	17610	0.0000*2
4	76 → 79<br>77 → 78	$E_{1u} \to E_{1g}$<br>$E_{1u} \to E_{1g}$	A_{1u}[a)]	1	2.4230	511.70	19543	0.0000
1	76 → 79<br>77 → 78	$E_{1u} \to E_{1g}$<br>$E_{1u} \to E_{1g}$	A_{1u}[a)]	0	2.4284	510.56	19586	0.0000
2,3	76 → 78,79<br>77 → 79,78	$E_{1u} \to E_{1g}$<br>$E_{1u} \to E_{1g}$	E_{2u}[a)]	0	2.5487	486.47	20557	0.0000*2
6	76 → 78<br>77 → 79	$E_{1u} \to E_{1g}$<br>$E_{1u} \to E_{1g}$	A_{2u}	0	3.8649	320.79	31172	1.8566
7,8	71 → 79,78<br>72 → 78,79<br>73 → 78,79	$E_{2u} \to E_{1g}$<br>$E_{2u} \to E_{1g}$<br>$A_{2u} \to E_{1g}$	E_{1u}	0	3.8916	318.60	31388	0.0021*2
16,17	71 → 79,78<br>72 → 78,79<br>73 → 78,79	$E_{2u} \to E_{1g}$<br>$E_{2u} \to E_{1g}$<br>$A_{2u} \to E_{1g}$	E_{1u}	0	4.2985	288.43	34669	0.0655*2
28	67 → 78<br>68 → 79	$E_{1u} \to E_{1g}$<br>$E_{1u} \to E_{1g}$	A_{2u}	0	4.5333	273.50	36563	0.1121
31,32	76,77 → 80	$E_{1u} \to A_{1g}$	E_{1u}	0	4.6874	264.51	37806	0.0048*2

a) These transitions can gain intensity through spin-orbit coupling

Table S10. Calculated transitions in $[Au^{+V}(\textit{nido}\text{-}B_{11}H_{11})_2]^{3-}$, B3LYP/aug-cc-pvdz (B, H), /aug-cc-pvdz-PP (Au); SCRF(Solvent=Water); TD(NStates=400); (HOMO-77; LUMO-78)

excited State Nr	excited State	symmery		ΔS	excitation energie, eV	excitation energie, nm	excitation energie, cm^{-1}	oscillator strength
1	76 → 78<br>77 → 79	$E_{1u} \rightarrow E_{1g}$<br>$E_{1u} \rightarrow E_{1g}$	A_{2u}[a)]	1	3.0504	406.45	24603	0.0000
2,3	76 → 78,79<br>77 → 79,78	$E_{1u} \rightarrow E_{1g}$<br>$E_{1u} \rightarrow E_{1g}$	E_{2u}[a)]	1	3.1939	388.19	25760	0.0000*2
4	76 → 79<br>77 → 78	$E_{1u} \rightarrow E_{1g}$<br>$E_{1u} \rightarrow E_{1g}$	A_{1u}[a)]	1	3.3821	366.59	27278	0.0000
1	76 → 79<br>77 → 78	$E_{1u} \rightarrow E_{1g}$<br>$E_{1u} \rightarrow E_{1g}$	A_{1u}[a)]	0	3.3934	365.37	27369	0.0000
2,3	76 → 78,79<br>77 → 79,78	$E_{1u} \rightarrow E_{1g}$<br>$E_{1u} \rightarrow E_{1g}$	E_{2u}[a)]	0	3.4698	357.33	27986	0.0000*2
8	76 → 78<br>77 → 79	$E_{1u} \rightarrow E_{1g}$<br>$E_{1u} \rightarrow E_{1g}$	A_{2u}	0	4.4978	275.65	36277	1.8365
9,10	71 → 79,78<br>72 → 78,79<br>73 → 78,79	$E_{2u} \rightarrow E_{1g}$<br>$E_{2u} \rightarrow E_{1g}$<br>$A_{2u} \rightarrow E_{1g}$	E_{1u}	0	4.5844	270.45	36975	0.0007*2
16,17	71 → 79,78<br>72 → 78,79<br>73 → 78,79<br>76,77→ 80	$E_{2u} \rightarrow E_{1g}$<br>$E_{2u} \rightarrow E_{1g}$<br>$A_{2u} \rightarrow E_{1g}$<br>$E_{1u} \rightarrow A_{1g}$	E_{1u}	0	4.9411	250.92	39852	0.0557*2
19,20	71 → 79,78<br>72 → 78,79<br>73 → 78,79<br>76,77→ 80	$E_{2u} \rightarrow E_{1g}$<br>$E_{2u} \rightarrow E_{1g}$<br>$A_{2u} \rightarrow E_{1g}$<br>$E_{1u} \rightarrow A_{1g}$	E_{1u}	0	4.9634	249.80	40032	0.0179*2

a) These transitions can gain intensity through spin-orbit coupling

Table S11. Calculated transitions in $[Au^{+V}(\textit{nido}\text{-}B_{11}H_{11})_2]^{3-}$, B3LYP/6-311++g(d,p) (B, H), /SDD (Au); SCRF(Solvent=Water); TD(NStates=400); (HOMO-77; LUMO-78)

excited State Nr	excited State	symmery		ΔS	excitation energie, eV	excitation energie, nm	excitation energie, cm^{-1}	oscillator strength
1	76 → 78<br>77 → 79	$E_{1u} \rightarrow E_{1g}$<br>$E_{1u} \rightarrow E_{1g}$	A_{2u}[a)]	1	2.9254	423.82	23595	0.0000
2,3	76 → 78,79<br>77 → 79,78	$E_{1u} \rightarrow E_{1g}$<br>$E_{1u} \rightarrow E_{1g}$	E_{2u}[a)]	1	3.0726	403.52	24782	0.0000*2
4	76 → 79<br>77 → 78	$E_{1u} \rightarrow E_{1g}$<br>$E_{1u} \rightarrow E_{1g}$	A_{1u}[a)]	1	3.2663	379.59	26344	0.0000
1	76 → 79<br>77 → 78	$E_{1u} \rightarrow E_{1g}$<br>$E_{1u} \rightarrow E_{1g}$	A_{1u}[a)]	0	3.2785	378.17	26443	0.0000
2,3	76 → 78,79<br>77 → 79,78	$E_{1u} \rightarrow E_{1g}$<br>$E_{1u} \rightarrow E_{1g}$	E_{2u}[a)]	0	3.3609	368.90	27107	0.0000*2
8	76 → 78<br>77 → 79	$E_{1u} \rightarrow E_{1g}$<br>$E_{1u} \rightarrow E_{1g}$	A_{2u}	0	4.4576	278.14	35953	1.9152
9,10	71 → 79,78<br>72 → 78,79<br>73 → 78,79	$E_{2u} \rightarrow E_{1g}$<br>$E_{2u} \rightarrow E_{1g}$<br>$A_{2u} \rightarrow E_{1g}$	E_{1u}	0	4.5287	273.78	36526	0.0005*2
16,17	71 → 79,78<br>72 → 78,79<br>73 → 78,79<br>76,77→ 80	$E_{2u} \rightarrow E_{1g}$<br>$E_{2u} \rightarrow E_{1g}$<br>$A_{2u} \rightarrow E_{1g}$<br>$E_{1u} \rightarrow A_{1g}$	E_{1u}	0	4.8981	253.13	39506	0.0743*2
19,20	71 → 79,78<br>72 → 78,79<br>76,77→ 80	$E_{2u} \rightarrow E_{1g}$<br>$E_{2u} \rightarrow E_{1g}$<br>$E_{1u} \rightarrow A_{1g}$	E_{1u}	0	4.9366	251.16	39816	0.0002*2

a) These transitions can gain intensity through spin-orbit coupling

Table S12. Orbital energies (cm^{-1}) of the d-electrons in $[M(B_{11}H_{11})_2]^{3-}$ (M = Cu, Ag, Au). B3LYP/6-311++g(d,p) (B, H), /SDD (M); SCRF (solvent = water). There are a total of 50 MOs with valence electrons.

Orbitalle	Cu	Ag	Au
$\Delta(d_{xz},d_{yz}/d_{xy},d_{x2-y2})$	37140	38306	42144
$\Delta(d_{xz},d_{yz}/d_{z2})$	52081	50990	54047
$\Delta(d_{xy},d_{x2-y2}/d_{z2})$	14941	12684	11903
d_{xz},d_{yz} [LUMO+x]	−16458[0,1]	−16873[0,1]	−12042[0,1]
d_{xy},d_{x2-y2} [HOMO−x]	−53598[3,4]	−55179[2,3]	−54186[2,3]
d_{z2} (+s) [HOMO−x]	−68539[17]	−67863[16]	−66089[15]
Additional MO with large amounts of metal orbitals			
p_x,p_y [HOMO−x]	−45480[0,1]	−44568[0,1]	−47567[0,1]
p_z [HOMO−x]	−53121[2]	−55700[4]	−56187[4]
s (+d_{z2}) [HOMO−x]	−64227[15]<br>−88674[32]	−64600[15]	−67923[16]<br>−91841[32]
d_{xz},d_{yz} [HOMO−x]	−72180[18,19]<br>−76022[26,27]<br>−92501[34,35]	−81566[26,27]<br>−100801[41,42]<br>−129751[47,48]	−74566[18,19]<br>−80549[26,27]<br>−102815[41,42]<br>−130447[47,48]
d_{xy},d_{x2-y2} [HOMO−x]	−73565[20,21]<br>−80454[29,30]<br>−97761[41,42]	−75833[20,21]<br>−87253[30,31]<br>−99974[39,40]	−75096[20,21]<br>−86297[30,31]<br>−100692[39,40]
d_{z2} (+s) [HOMO−x]	−81615[31]<br>−94075[36]	−86172[29]<br>−89332[32]<br>−97586[38]	−85502[29]<br>−98557[38]

Table S13. Some thermal ellipsoids in $K_3[M(B_{11}H_{11})_2]\cdot 2H_2O$ (M = Cu, Ag und Au) at 100 K.

M/X	U_{max}, $Å^2$ [a]	U_{mid}, $Å^2$ [a]	U_{min}, $Å^2$ [a]	U_{eq}, $Å^2$	$U_{eq}(X)/U_{eq}(M)$
Cu/Cu	0.0144	0.0128	0.0110	0.01273	1
Ag/Ag	0.0071	0.0068	0.0063	0.00672	1
Au/Au	0.0091	0.0090	0.0086	0.00890	1
Cu/K1[b]	0.0316	0.0219	0.0189	0.02417	1.90
Cu/K3[b]	0.0846	0.0577	0.0187	0.05368	4.22
Ag/K1	0.0169	0.0168	0.0112	0.01494	2.22
Au/K1	0.0221	0.0211	0.0141	0.01909	2.14
Cu/K2	0.1470	0.1290	0.0167	0.09756	7.66
Ag/K2	0.0913	0.0628	0.0101	0.05472	8.14
Au/K2	0.1204	0.0852	0.0133	0.07297	8.20
Cu/O	0.1615	0.0256	0.0252	0.07077	5.56
Ag/O	0.0489	0.0155	0.0153	0.02658	3.96
Au/O	0.0675	0.0198	0.0192	0.03550	3.99

[a] Principal mean square atomic displacements U

[b] Occupancy ratio of K1 is 0.830(16) and of K3 is 0.170(16)

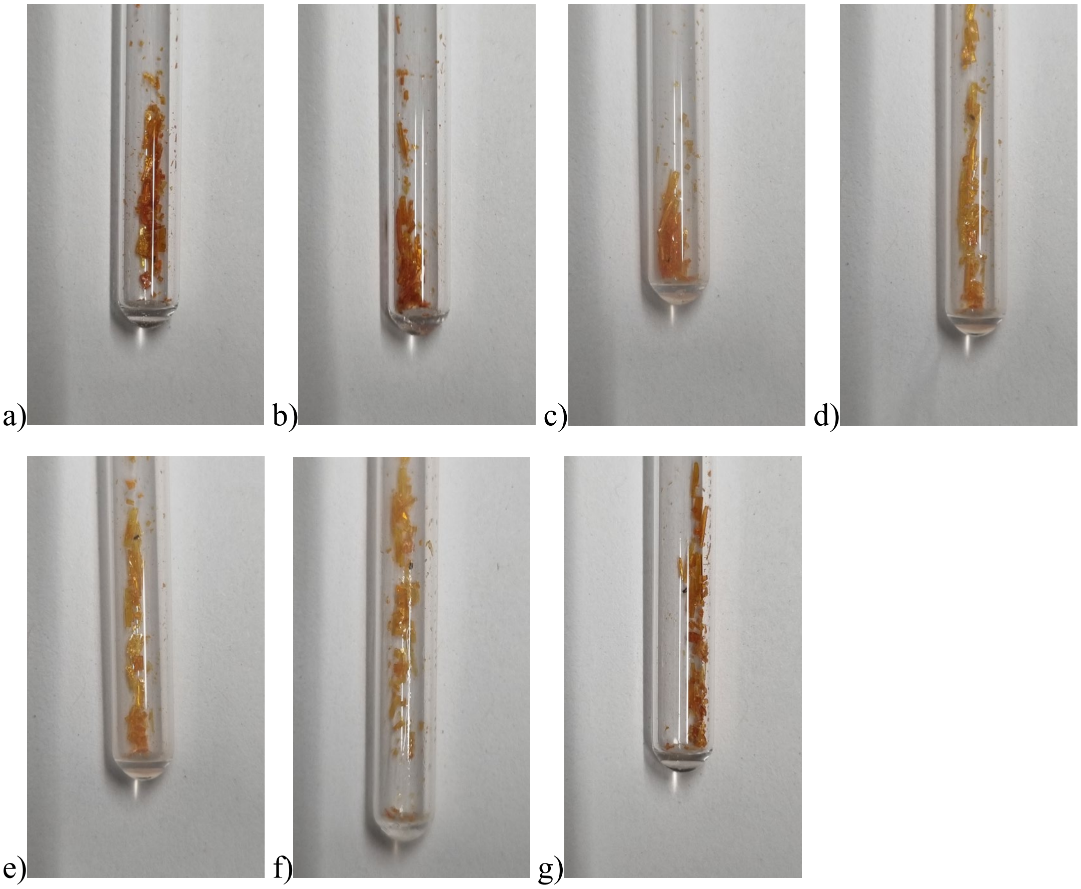

Figure S45. Color of $[n\text{-}Bu_4N]_3[Ag(B_{11}H_{11})_2]$ at different temperatures: a) at room temperature, b) liquid nitrogen for 1 s, c) liquid nitrogen for 2 s, d) liquid nitrogen for 3 s, e) liquid nitrogen for 5 s, f) liquid nitrogen for 300 s, g) at room temperature.

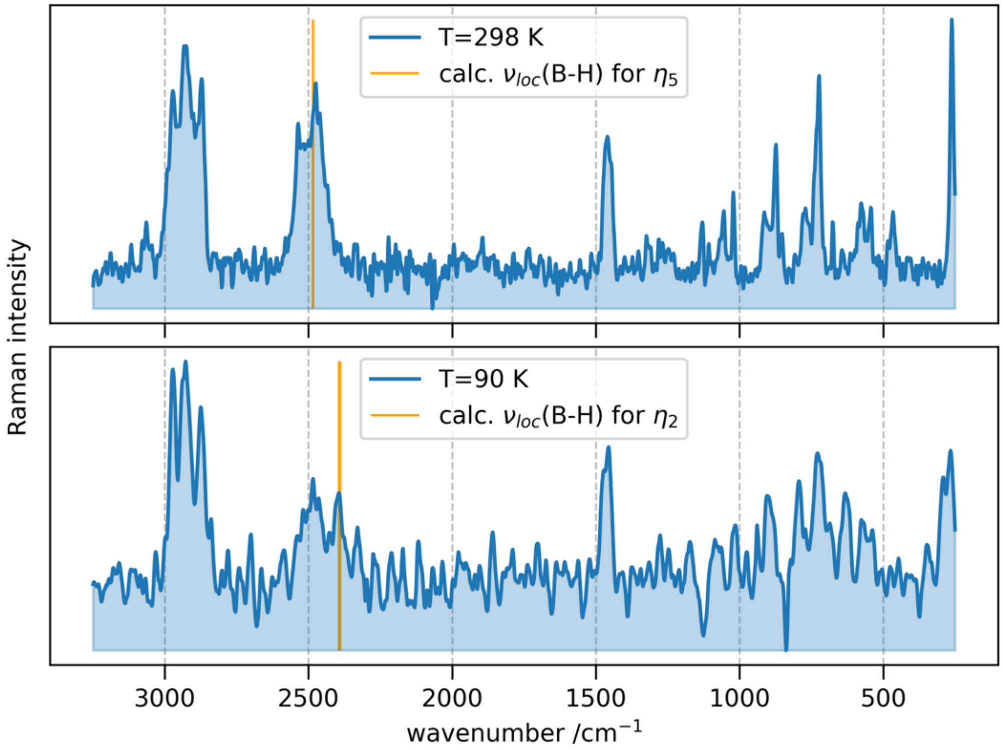

Figure S46. Raman spectra of $[n\text{-}Bu_4N]_3[Ag(B_{11}H_{11})_2]$ at different temperatures.

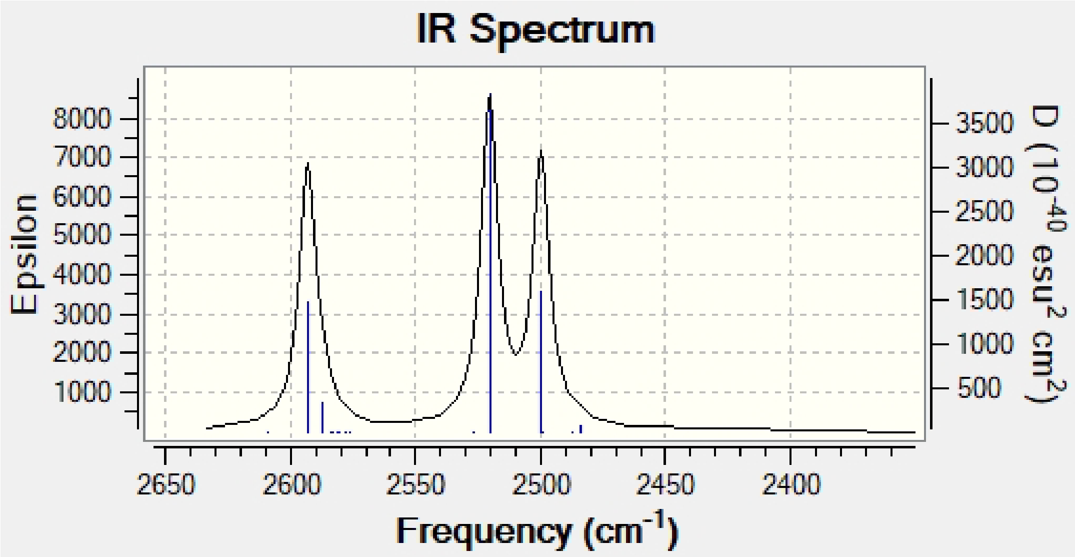

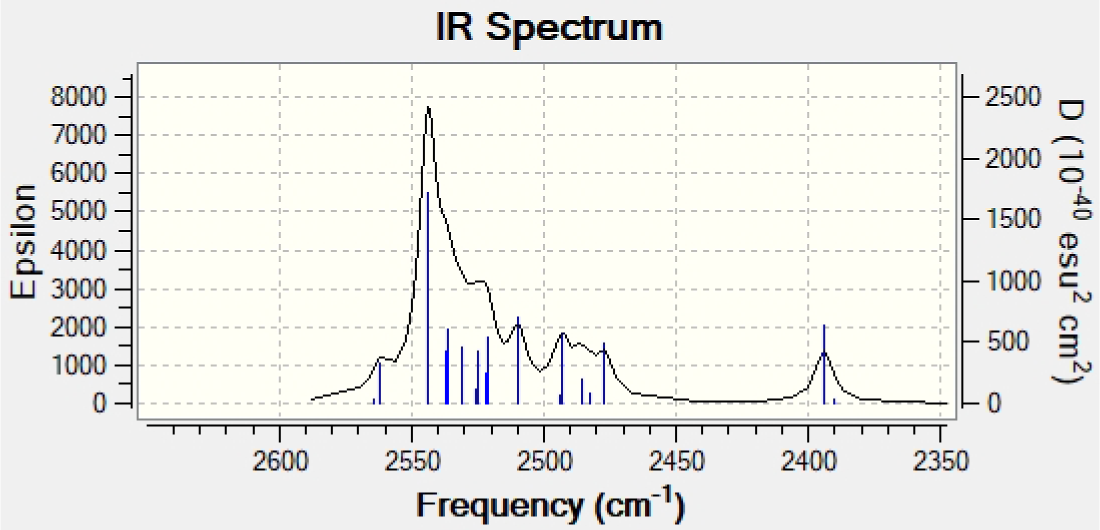

Figure S47. Calculated IR spectra of $[Ag(\eta^5\text{-}B_{11}H_{11})_2]^{3-}$ (up, ν(BH) 2484-2609 cm^{-1}) and $[Ag(\eta^2\text{-}B_{11}H_{11})_2]^{3-}$ (down, ν(BH) 2390-2564 cm^{-1}). B3LYP/aug-cc-pvtz (B, H), /aug-cc-pvtz-PP (Ag).

Table S14. Relative energy (ΔE, kJ/mol), dihedral angle τ (4-5-15-16, °) and average angle α_{av} (3-4-1 and 6-5-1, °) of the rotamers of the isomer with oxidation number +I (d^{10} complex) for $[M(B_{11}H_{11})_2]^{3-}$ (M = Cu, Ag, Au). Functional: B3LYP. Basis set: for B, H - 6-311++g(d,p) for M - SDD. Energies are without corrections due to imaginary frequencies of rotamers C_{2h} and C_{2v}.

	M	point group		
		C_{2h}	C_2	C_{2v}
ΔE, without solvent	Cu	14.1	0	19.7
	Ag	4.5	0	7.8
	Au	0.0	0	7.2
ΔE, SCRF(Solvent=Water)	Cu	3.9	0	7.5
	Ag	1.0	0	1.4
	Au	2.2	0	3.7
|τ|, without solvent	Cu	0	85.09	180
	Ag	0	79.78	180
	Au	0	37.27	180
|τ|, SCRF(Solvent=Water)	Cu	0	93.78	180
	Ag	0	65.92	180
	Au	0	81.42	180
α_{av}, without solvent	Cu	111.99	122.44	117.16
	Ag	109.65	117.44	114.22
	Au	99.25	99.89	103.86
α_{av}, SCRF(Solvent=Water)	Cu	94.03	113.88	97.76
	Ag	93.21	94.73	96.40
	Au	89.50	88.98	92.87

Experimental data for $(n\text{-}Bu_4N)_3[Ag\text{-}(\eta^2\text{-}B_{11}H_{11})_2]$ at 100 K are: |τ| = 139.0 °; α = 82.07(18) °, 86.42(17) °, 82.78(14) °, 80.28(14) °; α_{av} = 82.9(26) °.

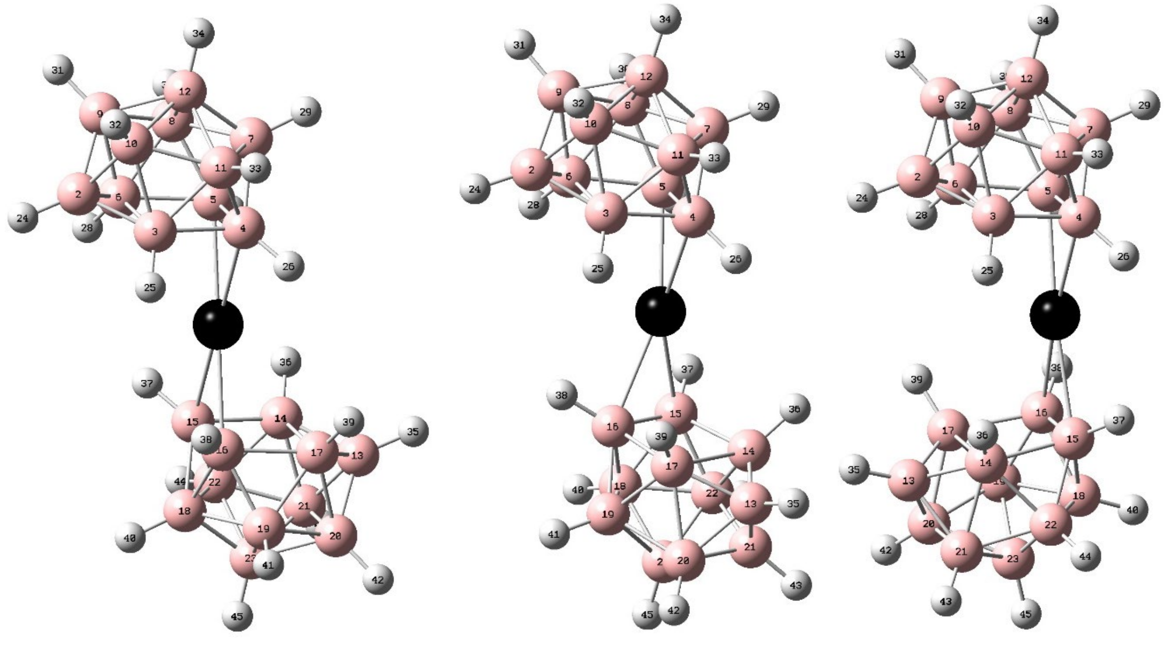

C_{2h} C_2 C_{2v}

Figure S48. The rotamers of the isomer with oxidation number +I (d^{10} complex) for $[M(B_{11}H_{11})_2]^{3-}$ (M = Cu, Ag, Au).

X-ray absorption spectroscopy

X-ray absorption spectra at the Cu K-edge were acquired at the ASTRA beamline of the SOLARIS synchrotron (Kraków, Poland). For measurements, sample pellets were prepared by mixing the accurately calculated mass of each compound (using the XAFSmass program) with 70 mg of ultra-pure microcrystalline cellulose (ACROS Organics, CAS 9004-34-6). The mixture was homogenized using a mortar and pestle and subsequently pressed at 2.5 metric tons. The incident photon beam was generated by a double bend achromatic 1.3 Tesla bending magnet with a critical energy of approximately 2 keV and monochromatized using a Ge(220) modified Lemonnier-type double crystal monochromator. Slits defined the monochromatized beam, resulting in a beam size of 7 × 1 mm at the sample position. Spectra were collected in transmission mode using ionization chambers filled with nitrogen gas at atmospheric pressure. A reference Cu foil (Exafs Company, USA) was positioned between the second and third ionization chambers for energy calibration and alignment of the collected spectra. The Demeter software package was used for data processing and analysis.

XANES data

The electronic structure and local geometry of $K_3[Cu(B_{11}H_{11})_2]\cdot 5H_2O$ ($B_{22}CuH_{32}K_3O_5$) were investigated by analyzing the XANES region of the Cu K-edge X-ray absorption spectra (Figure 3). The Cu K-edge absorption edge for $K_3[Cu(B_{11}H_{11})_2]\cdot 5H_2O$, determined at the half-height of the edge step, appears at 8991.1 eV—more than 5 eV higher than for Cu_2O (8984.6 eV) and CuO (8985.9 eV). Typically, the K-edge position is sensitive to the oxidation state and local electron density at the copper site, as it measures the energy needed to excite a 1s electron to unoccupied 4p states. A pronounced shift to higher energy suggests a significant decrease in electron density at copper, corresponding either to a formally higher oxidation state, strong ligand electron withdrawal, or both. A shift of more than 5 eV is unusual among copper compounds and is strong evidence of an exceptionally electron-deficient or high oxidation state Cu environment in this boron-rich cluster, consistent with its unique polyhedral cage structure.

Crystallographic and EXAFS analysis corroborates that the copper atom is encapsulated by twelve boron atoms within a polyhedral cage, locking the copper into an exceptionally electron-deficient coordination environment. As a result, the effective nuclear charge on copper is increased, raising the 1s electron binding energy and thus shifting the XANES absorption edge to higher energy. This prominent rightward edge shift is direct evidence of the unusual and highly oxidized local environment at the copper center in $K_3[Cu(B_{11}H_{11})_2]\cdot 5H_2O$.

These data are consistent with the X-ray photoelectron spectrum data.[23] The binding energy of $Cu(2p_{3/2})$ electrons is significantly larger in $(n\text{-}Bu_4N)_3[Cu(B_{11}H_{11})_2]$ (937.8 eV) than in $CuSO_4\cdot 5H_2O$ (935.9 eV), CuF_2 (935.8 eV), CuO (933.6 eV), CuCN (932.6 eV) and Cu (932.4 eV).[23]

In Cu K-edge XANES spectra, a shoulder feature commonly appears at the absorption edge. This shoulder is particularly evident in Cu-oxides like CuO and Cu_2O as well as in the $K_3[Cu(B_{11}H_{11})_2]\cdot 5H_2O$ polyborate compound (Figure 3). This feature arises due to transitions from the 1s core level of copper to specific unoccupied states (primarily the 4p orbitals) that are strongly influenced by the local electronic structure and coordination environment around the copper atom. In Cu-oxides, especially with planar (CuO) or linear (Cu_2O) geometry, the energy splitting of the Cu 4p states (originating from ligand-field effects) produces a clear shoulder on the edge.[39,40] This is because the 4p orbitals perpendicular to the bond axes (in-plane) remain at lower energy and are directly accessible by dipole-allowed transitions. The shoulder feature on the X-ray absorption edge is typically less pronounced or absent in highly centrosymmetric environments (such as octahedral coordination) or in systems with high covalency, both of which tend to either mix or shift the metal 4p states away from the edge region. In

$K_3[Cu(B_{11}H_{11})_2]\cdot 5H_2O$, although the formal site symmetry of copper is centrosymmetric, the distinct shoulder observed on its absorption edge is mainly due to the unique encapsulation of the copper ion by a polyborate cage. This encapsulation induces an unusual ligand field, amplifying the splitting of the 4p energy levels and thus enhancing the shoulder feature.

White line features in Cu K-edge XANES spectra are sensitive to both the oxidation state and coordination environment of copper. $K_3[Cu(B_{11}H_{11})_2]\cdot 5H_2O$ XANES exhibits its white line at the highest energy among all specimens, with the maximum just above 8990 eV, where Cu_2O has white line at 8995.3 eV and CuO at 8997.5 eV. Higher intensity and higher-energy white lines are characteristic of more covalent and often higher symmetry.[40] The sharp, intense, and blue-shifted white line for $K_3[Cu(B_{11}H_{11})_2]\cdot 5H_2O$ therefore indicates a more covalent, "rigid" and electronically distinct coordination with boron compared to oxygen, and possibly higher average oxidation state and/or lower ligand field splitting.

Based on the data obtained from EXAFS (SI) and XANES analysis, one could state that for $K_3[Cu(B_{11}H_{11})_2]\cdot 5H_2O$, the uniformity of bond distances and angles, absence of alternative donors, and the short Cu–B distances together justify describing the coordination as both covalent and rigid, with electronic distinctness deriving from this unique boron-only coordination shell.

EXAFS data of $K_3[Cu(B_{11}H_{11})_2]\cdot5H_2O$

To determine the local structure around Cu atoms, EXAFS (Extended X-ray Absorption Fine Structure) spectroscopy was employed.

The EXAFS signal, χ(k), can be described by the following equation:

$$\chi(k) = \sum_i \frac{N_i S_0^2 F_{eff\,i}(k)}{kR_i^2} \exp(-2k^2\sigma_i^2) \exp\left(-\frac{2R_i}{\lambda(k)}\right) \sin[2kR_i + \varphi_i(k)]$$

where:

N_i is the coordination number for shell *i* (number of atoms at a given distance)

S_o^2 – amplitude reduction factor

F_{effi} *(k)*- scattering amplitudę

R_i-path length (radial distance from shell *i*)

σ^2_i: Debye-Waller factor (measures atomic disorder)

ϕ_i(k) – phase shift

λ(k) – mean-free path of the photoelectrons.

F_{effi} *(k)*, ϕ_i(k), and λ(k) are calculated from theoretical models generated by the FEFF program integrated within Demeter. The parameters N_i, S_o^2, R_i, and σ^2_i are refined by fitting. ΔE_o (energy shift aligning theory and experiment) is also used as a fit parameter.

Because Ni and S_o^2 are correlated, at least one must be fixed during fitting. S_o^2 is a non-structural parameter determined for each element using a reference sample of known coordination. In this study, Cu foil was used as the reference: the model-generated N_i values were fixed, and S_o^2 was fitted, yielding a value of 0.95 used in subsequent analyses.

Data fitting included up to two coordination shells. The Fourier Transform of χ(k)·k² was performed over the k-range from 2.7 Å⁻¹ to 12.0 Å⁻¹ (Figure S49). The theoretical model predicted that, in R-space, the primary peak at 1.70 Å (not phase-corrected) corresponds to Cu–B interactions in the first shell, while the peak at 2.85 Å represents the second coordination shell. A feature at 2.30 Å is attributed to a multiple-scattering path forming an acute triangle (Figure S50).

Fitting was conducted from R = 1.0 Å to R = 3.3 Å. This range was chosen considering the low atomic number of boron, which fundamentally limits the precision of parameters for B-containing shells.[41,42] The EXAFS signal from boron is of low intensity and frequently overlaps with neighboring shells, complicating unambiguous assignment. To reduce parameter correlation during fitting, Ni values were fixed. Fitted parameters included interatomic distances (R), disorder factors (σ^2) for Cu–B bonds, and ΔE_o.

It should be noted that the absolute distances and Debye-Waller factors (σ^2) for boron scatterers exhibit increased uncertainty compared to those for heavier atoms such as copper or oxygen.

The results of the Cu K-edge EXAFS fitting are presented in Figures S49-S51 and Table S15. The R-factor of 1.3% indicates excellent agreement between the experimental data and the fitted model. The fitted bond lengths closely match those from the corresponding crystallographic information file (CIF), and the ΔR values are small, consistent with physically reasonable expectations. Disorder parameters (σ^2) are positive and fall within a realistic range for a crystalline solid at room temperature.

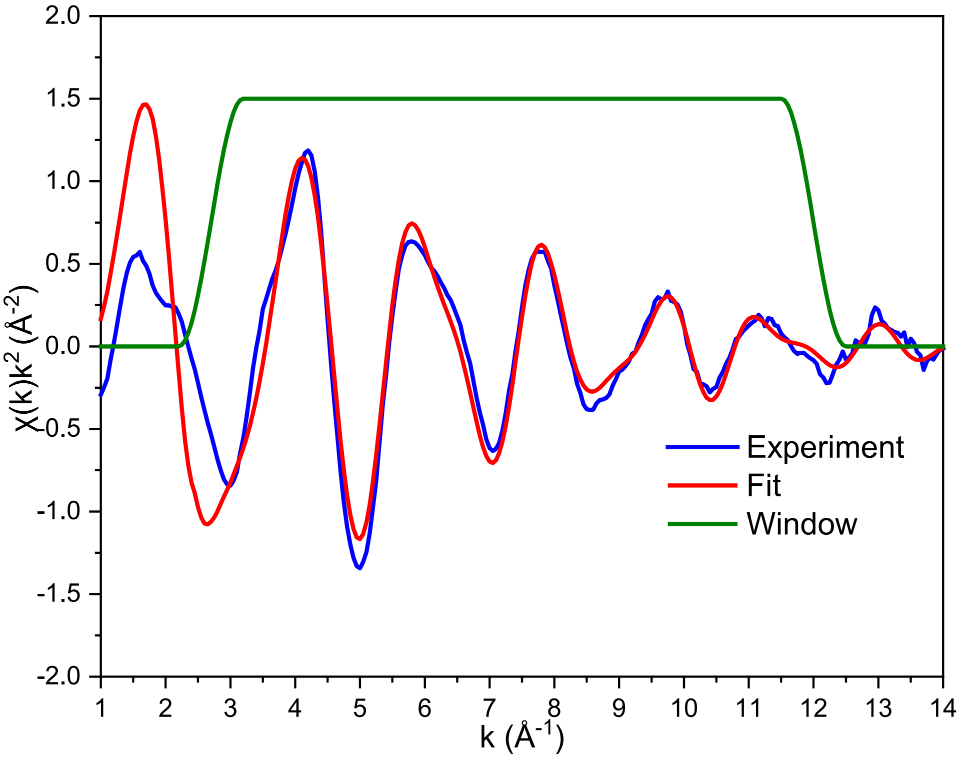

Figure S49. k^2-weighted χ(k) Cu K-edge EXAFS spectrum

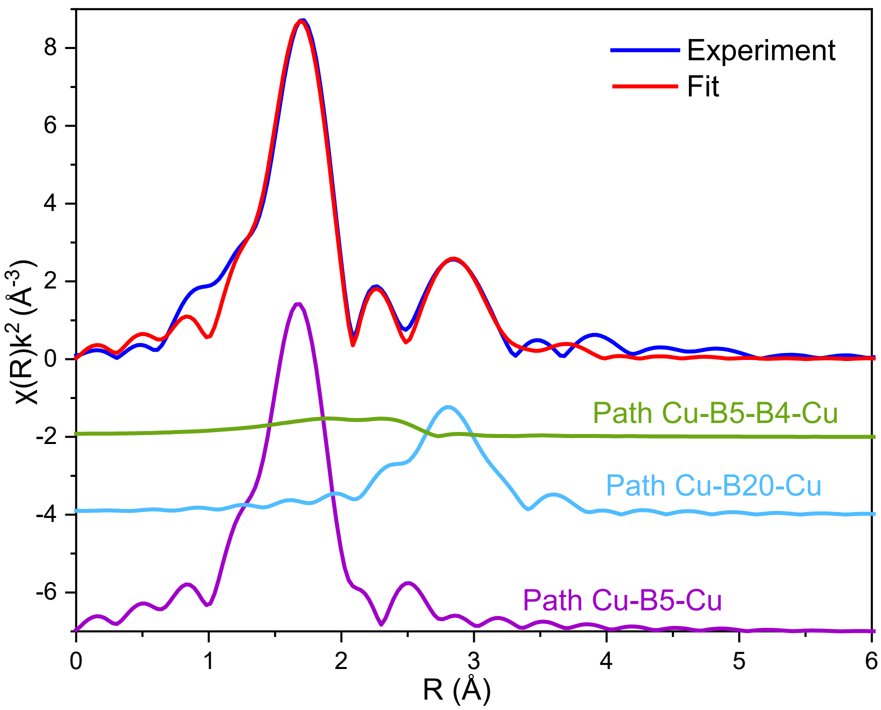

Figure S50. Fourier transform of k^2-weighted χ(k) (FT-EXAFS) with distinct scattering path contributions

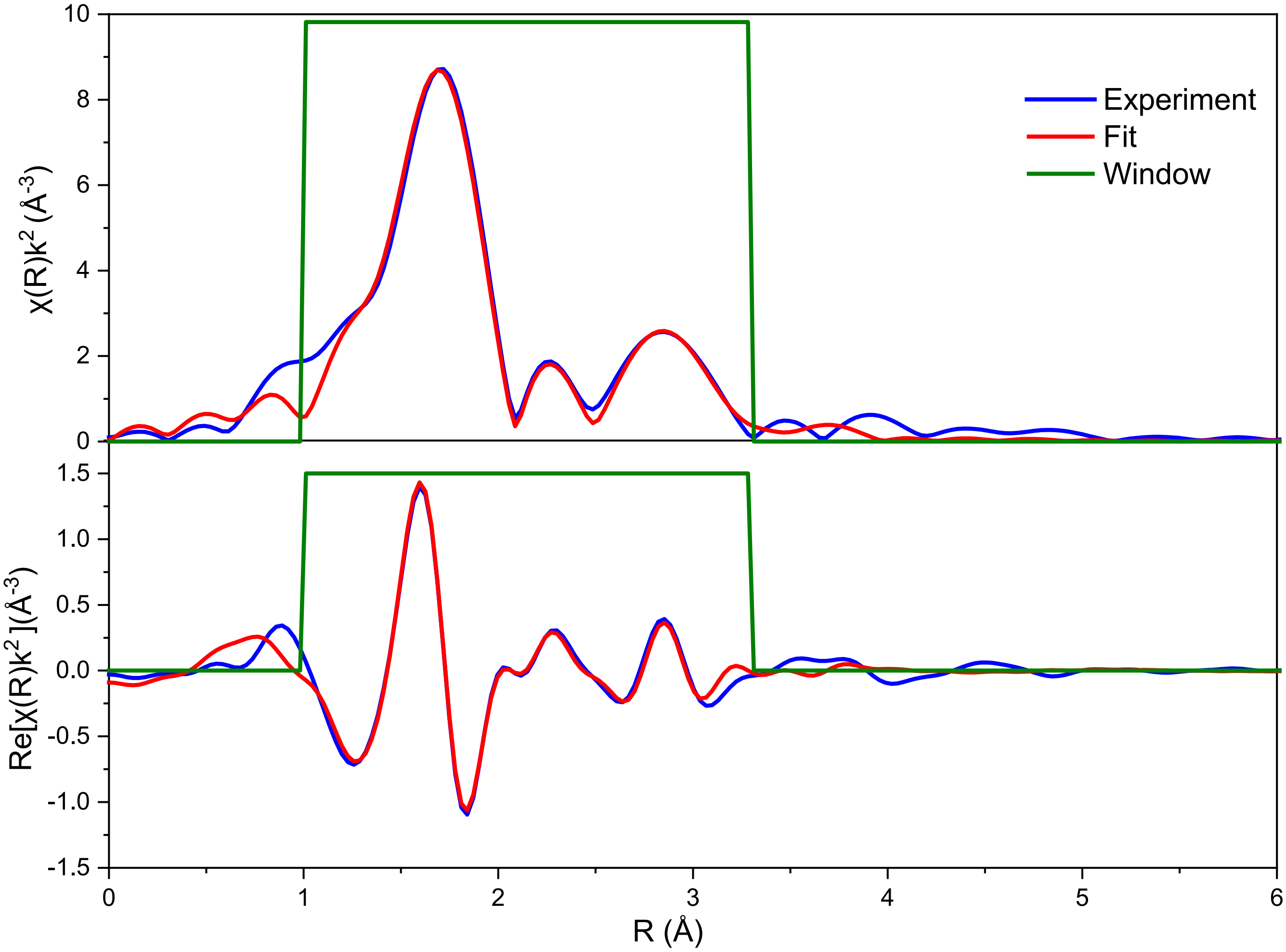

Figure S51. Fourier transformed k^2-weighted Cu K-edge EXAFS spectrum. Blue line is the FT-EXAFS, red line is the fitted spectrum and green is the window of the fitting.

Table S15. Best-fit parameters derived from the analysis of the Cu K-edge EXAFS.

Path*	*N* [–]	R_{eff} [Å]	S_0^2 [–]	*ΔE₀* [eV]	*ΔR* [Å]	σ^2 [-]	*R-factor [%]*
Cu-B5-Cu, *ss*	10	2.146			-0.0145±0.0106	0.0056±0.0007	
Cu-B5-B4-Cu, *at*	20	3.067	0.95	-2.8±1.5	-0.0807±0.0493	0.0086±0.0105	1.3
Cu-B20-Cu, *ss*	10	3.330			-0.0047±0.0180	0.0044±0.0018	

* *ss* – single scattering, *at* – acute triangle

[1] E. Bernhardt, A. Drichel, M. Krnel, E. Svanidze, A. Slabon, *Inorg. Chem.* **2024**, *63*, 5414.
[2] G. B. Dunks, K. P. Ordonez, *Inorg. Chem.* **1978**, *17*, 1514.
[3] G. B. Dunks, K. Barker, E. Hedaya, C. Hefner, K. Palmer-Ordonez, P. Remec, *Inorg. Chem.* **1981**, *20*, 1692.
[4] E. Bernhardt, H. Willner, DE102008004530A1, **2008**.
[5] G. E. Ryschkewitsch, *J. Am. Chem. Soc.* **1967**, *89*, 3145.
[6] E. Bernhardt, C. Bach, B. Bley, R. Wartchow, U. Westphal, I. H. T. Sham, B. von Ahsen, C. Wang, H. Willner, R. C. Thompson et al., *Inorg. Chem.* **2005**, *44*, 4189.
[7] M. Finze, E. Bernhardt, H. Willner, C. W. Lehmann, F. Aubke, *Inorg. Chem.* **2005**, *44*, 4206.
[8] W. C. Schumb, E. L. Gamble, M. D. Banus, *J. Am. Chem. Soc.* **1949**, *71*, 3225.
[9] H. C. Andersen, L. H. Belz, *J. Am. Chem. Soc.* **1953**, *75*, 4828.
[10] R. D. Bohl, G. L. Galloway, *J. Inorg. Nucl. Chem.* **1971**, *33*, 885.
[11] E. Haack, DE598879A.
[12] E. Haack, DE600499A.
[13] E. Koenigs, H. Greiner, *Ber. dtsch. Chem. Ges. A/B* **1931**, *64*, 1049.
[14] E. Haack, DE613402A.
[15] *CrysAlisPro 1.171.42.88a*, Rigaku OD, 2023.
[16] G. M. Sheldrick, *Acta Crystallogr. A* **2008**, *64*, 112.
[17] L. J. Farrugia, *J. Appl. Crystallogr.* **2012**, *45*, 849.
[18] G. M. Sheldrick, *Acta Crystallogr. C* **2015**, *71*, 3.
[19] G. M. Sheldrick, *SHELXL-2019/1*, Bruker AXS Inc., Madison, Wisconsin, USA, Madison, Wisconsin, USA, **2019**.
[20] K. Brandenburg, *Diamond, v.3.2f*, Crystal Impact GbR, **2001**.
[21] R. K. Harris, E. D. Becker, S. M. Cabral de Menezes, R. Goodfellow, P. Granger, *Pure Appl. Chem.* **2001**, *73*, 1795.
[22] G. R. Fulmer, A. J. M. Miller, N. H. Sherden, H. E. Gottlieb, A. Nudelman, B. M. Stoltz, J. E. Bercaw, K. I. Goldberg, *Organometallics* **2010**, *29*, 2176.
[23] J. G. Kester, D. Keller, J. C. Huffman, M. A. Benefiel, W. E. Geiger, C. Atwood, A. R. Siedle, G. A. Korba, L. J. Todd, *Inorg. Chem.* **1994**, *33*, 5438.
[24] E. A. Bernhardt, P. N. Komozin, *Russ. J. Inorg. Chem.* **1997**, *42*, 540-557; *Zurn. Neorg. Khim.*, **1997**, *42(4)*, 614-631.
[25] N. Wiberg, *Inorganic Chemistry*, De Gruyter Inc, Berlin/Boston, **2008**.
[26] A. F. Holleman, E. Wiberg, *Lehrbuch der anorganischen Chemie*, De Gruyter, Berlin, Boston, **1976**.
[27] W. Kohn, L. J. Sham, *Phys. Rev.* **1965**, *140*, A1133-A1138.
[28] A. D. Becke, *Phys. Rev. A* **1988**, *38*, 3098.
[29] A. D. Becke, *J. Chem. Phys.* **1993**, *98*, 5648.
[30] C. Lee, W. Yang, R. G. Parr, *Phys. Rev. B* **1988**, *37*, 785.
[31] R. Krishnan, J. S. Binkley, R. Seeger, J. A. Pople, *J. Chem. Phys.* **1980**, *72*, 650.
[32] T. Clark, J. Chandrasekhar, G. W. Spitznagel, P. V. R. Schleyer, *J. Comput. Chem.* **1983**, *4*, 294.
[33] M. Dolg, U. Wedig, H. Stoll, H. Preuss, *J. Chem. Phys.* **1987**, *86*, 866.
[34] D. Andrae, U. Huermann, M. Dolg, H. Stoll, H. Preu, *Theoret. Chim. Acta* **1990**, *77*, 123.
[35] P. Schwerdtfeger, M. Dolg, W. H. E. Schwarz, G. A. Bowmaker, P. D. W. Boyd, *J. Chem. Phys.* **1989**, *91*, 1762.
[36] T. H. Dunning, *J. Chem. Phys.* **1989**, *90*, 1007.
[37] R. A. Kendall, T. H. Dunning, R. J. Harrison, *The J. Chem. Phys.* **1992**, *96*, 6796.

[38] K. A. Peterson, C. Puzzarini, *Theoret. Chim. Acta* **2005**, *114*, 283.

[39] L. S. Kau, D. J. Spira-Solomon, J. E. Penner-Hahn, K. O. Hodgson, E. I. Solomon, *J. Am. Chem. Soc.* **1987**, *109*, 6433.

[40] A. A. Guda, S. A. Guda, A. Martini, A. N. Kravtsova, A. Algasov, A. Bugaev, S. P. Kubrin, L. V. Guda, P. Šot, J. A. van Bokhoven et al., *npj Comput. Mater.* **2021**, *7*.

[41] G. N. George, I. J. Pickering, *X-ray Absorption Spectroscopy*, De Gruyter, Berlin, Boston, **2024**.

[42] G. N. George, I. J. Pickering, *X-ray Absorption Spectroscopy*, De Gruyter, **2024**.
