# Supplementary material for: The Biicosahedral Complex Anions [M(B11H11)2]3− (M = Cu, Ag, Au): Synthesis and Unexpected Low‐Temperature Phase Transition of [Ag(η5‐B11H11)2]3− to [Ag(η2‐B11H11)2]3−
Source: Angew Chem Int Ed Engl. 2025 Nov 10;65(2):e19283. doi: 10.1002/anie.202519283 (PMC12790380; doi:10.1002/anie.202519283)
Supplement: Supplementary file 2 — Supporting Information [file ANIE-65-e19283-s001.zip › Ag_MO.pdf]

Functional: B3LYP, SCRF(Solvent=Water) Basis set: B, H, F: 6-311++g(d,p); Ag: SDD
Isovalue: 0.03

```
 Alpha  occ. eigenvalues --   -6.70264  -6.70257  -6.70257  -6.70254  -6.70250
 Alpha  occ. eigenvalues --   -6.70247  -6.70234  -6.70232  -6.70227  -6.70225
 Alpha  occ. eigenvalues --   -6.69157  -6.69154  -6.68978  -6.68974  -6.68971
 Alpha  occ. eigenvalues --   -6.68968  -6.68967  -6.68965  -6.68940  -6.68938
 Alpha  occ. eigenvalues --   -6.68936  -6.68934  -3.82026  -2.41251  -2.39737
 Alpha  occ. eigenvalues --   -2.39734  -0.72099  -0.71692  -0.59123  -0.59116
 Alpha  occ. eigenvalues --   -0.58939  -0.58932  -0.55829  -0.53460  -0.45935
 Alpha  occ. eigenvalues --   -0.45922  -0.45552  -0.45552  -0.44464  -0.44420
 Alpha  occ. eigenvalues --   -0.44419  -0.43186  -0.43168  -0.41951  -0.40885
 Alpha  occ. eigenvalues --   -0.39756  -0.39755  -0.39263  -0.37398  -0.37165
 Alpha  occ. eigenvalues --   -0.37164  -0.34873  -0.34863  -0.34844  -0.34843
 Alpha  occ. eigenvalues --   -0.34553  -0.34552  -0.34054  -0.34047  -0.31523
 Alpha  occ. eigenvalues --   -0.30921  -0.29434  -0.27343  -0.27339  -0.27325
 Alpha  occ. eigenvalues --   -0.27319  -0.27026  -0.27003  -0.26322  -0.26303
 Alpha  occ. eigenvalues --   -0.25612  -0.25609  -0.25379  -0.25142  -0.25141
 Alpha  occ. eigenvalues --   -0.20308  -0.20306
 Alpha virt. eigenvalues --  -0.07689  -0.07687  -0.00569   0.00588   0.01141
```

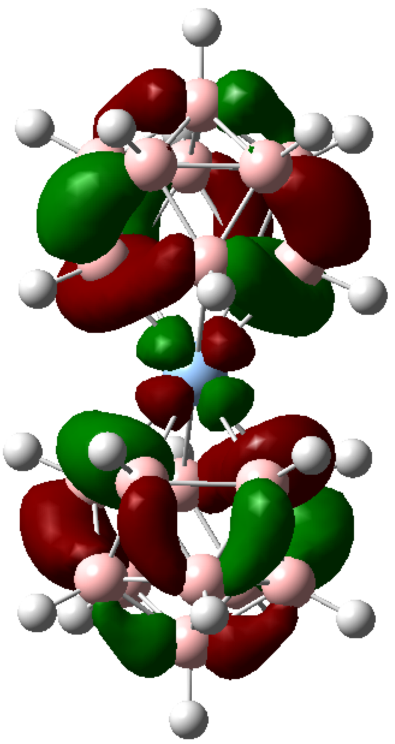
LUMO_00,01

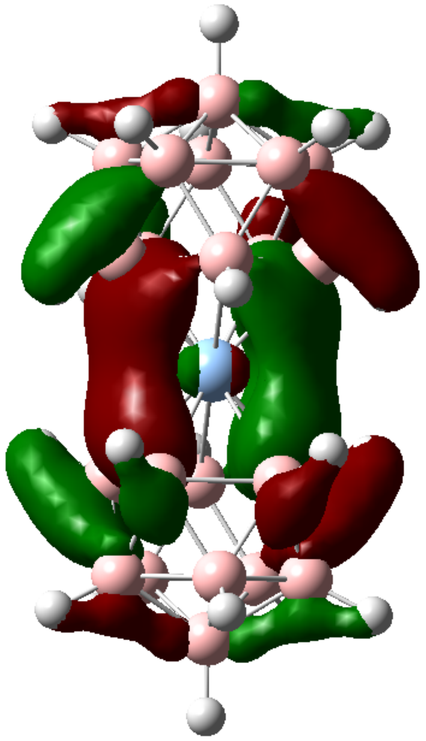
HOMO_00,01

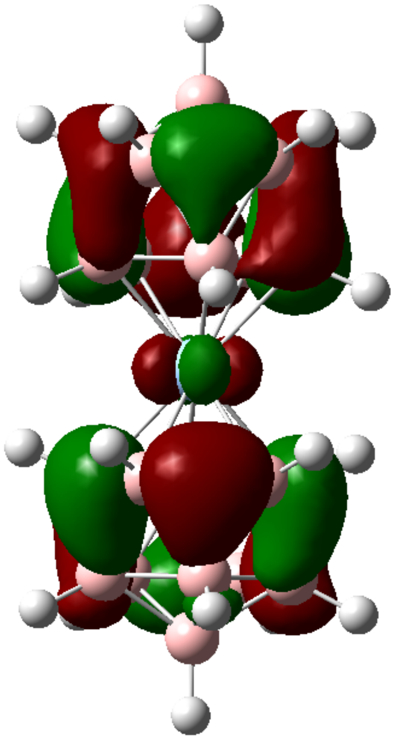
HOMO_02,03

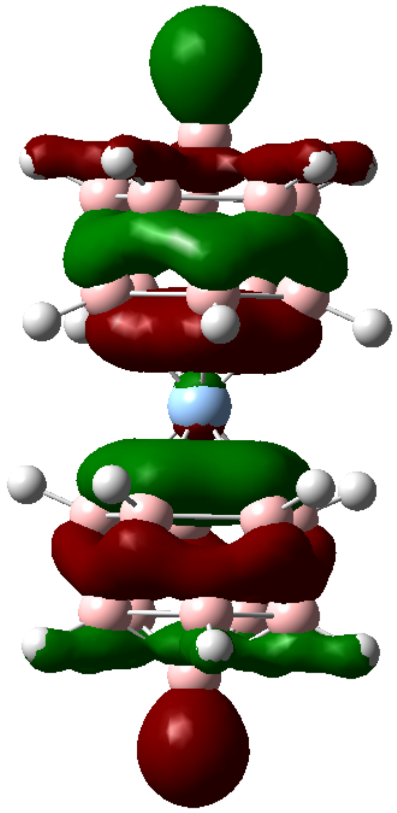
HOMO_04

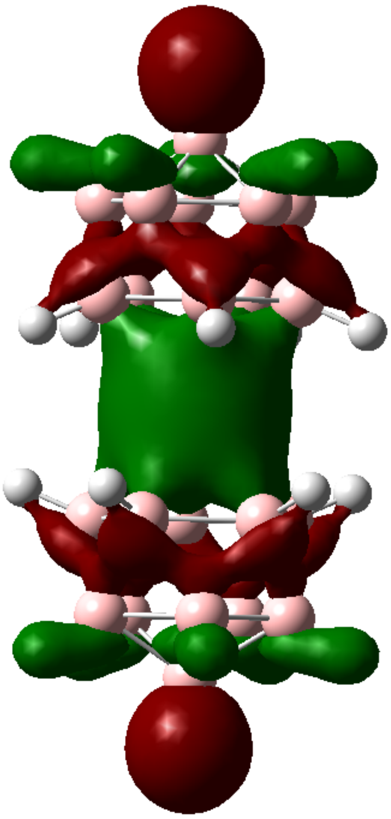

HOMO_15

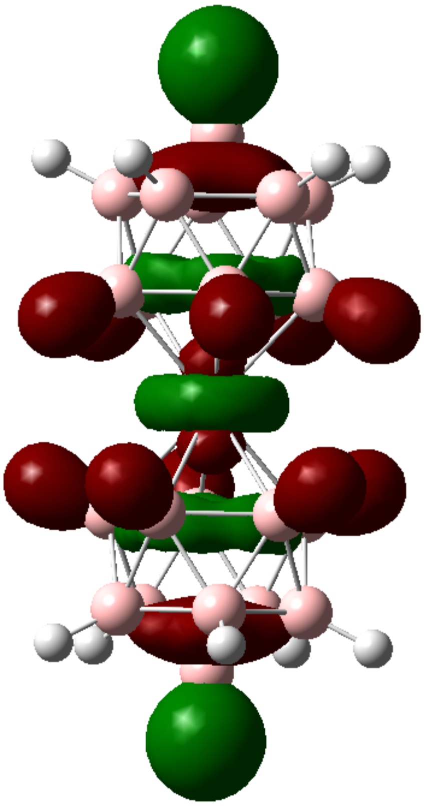

HOMO_16

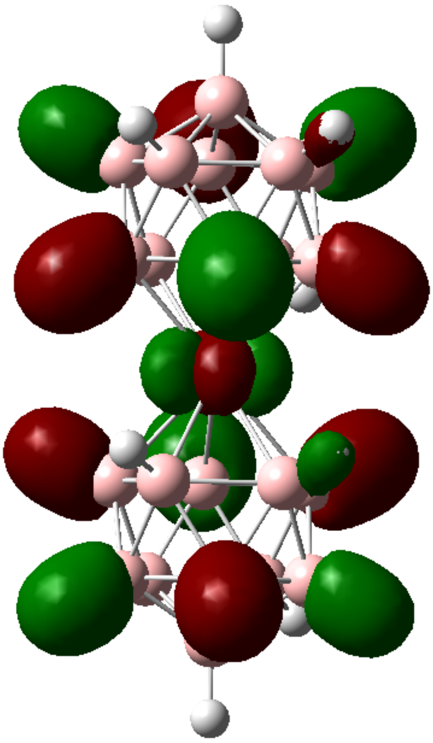

HOMO_20,21

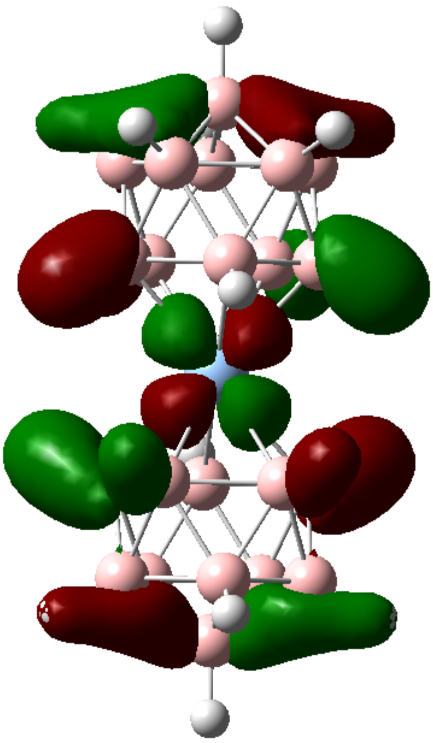

HOMO_26,27

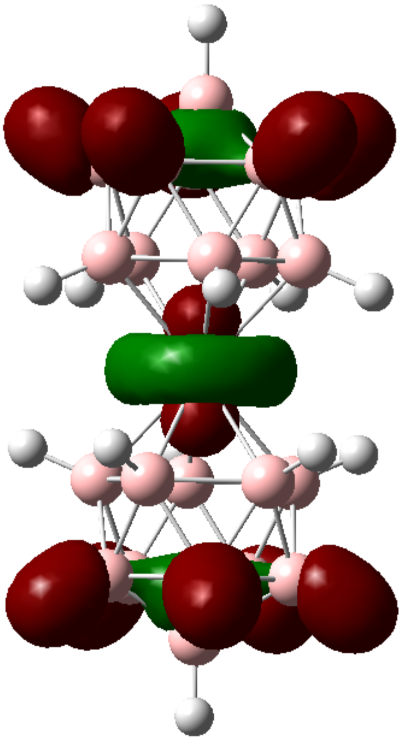

HOMO_29

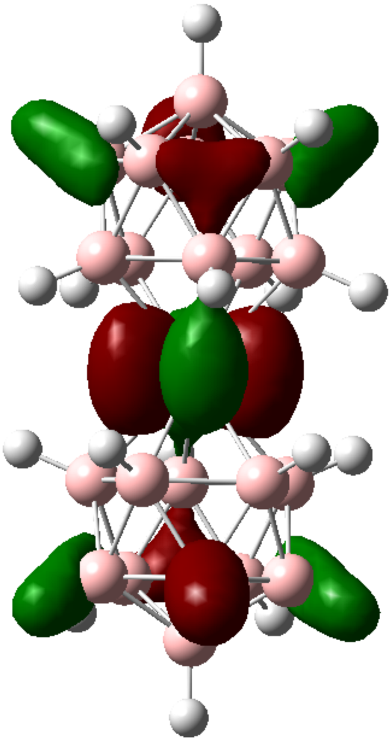

HOMO_30,31

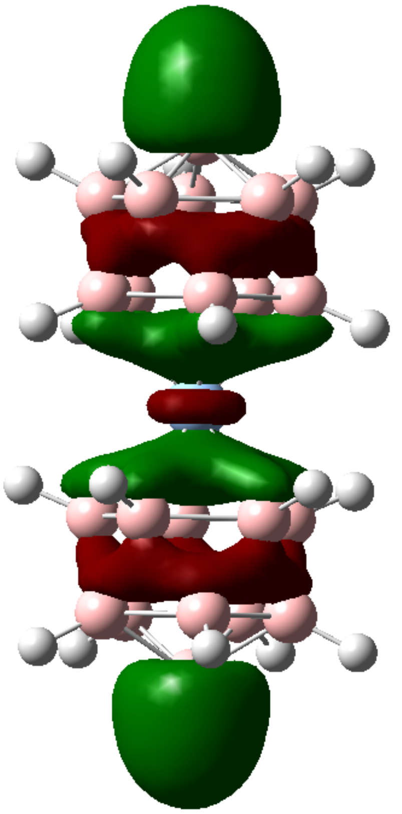

HOMO_32

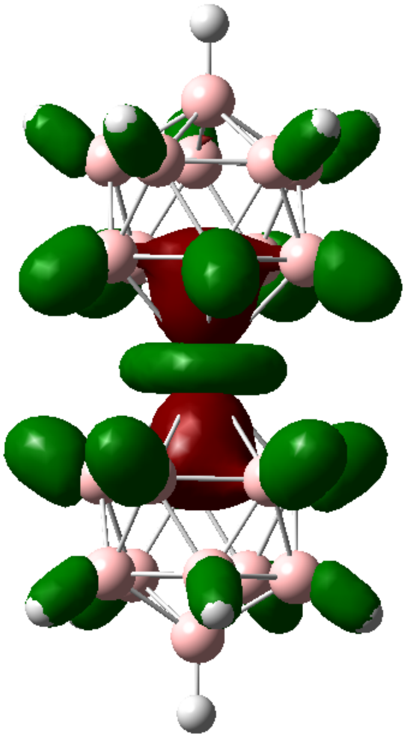

HOMO_38

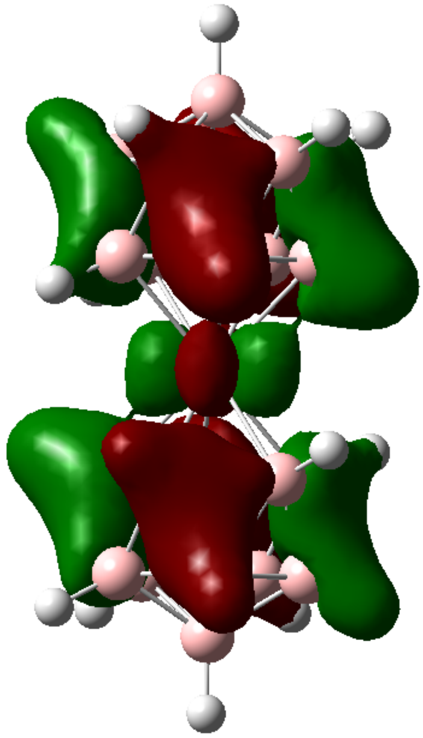

HOMO_39,40
