# Supplementary material for: The Biicosahedral Complex Anions [M(B11H11)2]3− (M = Cu, Ag, Au): Synthesis and Unexpected Low‐Temperature Phase Transition of [Ag(η5‐B11H11)2]3− to [Ag(η2‐B11H11)2]3−
Source: Angew Chem Int Ed Engl. 2025 Nov 10;65(2):e19283. doi: 10.1002/anie.202519283 (PMC12790380; doi:10.1002/anie.202519283)
Supplement: Supplementary file 2 — Supporting Information [file ANIE-65-e19283-s001.zip › AtomicCoordinats.pdf]

Atomic coordinates (Å), point groups, energies (a. u.) and first tree frequencies (cm^{-1}) for $[M(B_{11}X_{11})_2]^{3-}$ (M = Cu, Ag, Au; X = H, F; 45 atoms; charge −3).

Part 1: method – B3LYP, basis set – 6-311++g(d,p) for B, H, F and SDD for Cu, Ag, Au. Spin multiplicity: 1 (spin = 0).

Part 2: method – B3LYP, basis set – 6-311++g(d,p) for B, H, F and SDD for Cu, Ag, Au. SCRF(Solvent=Water). Spin multiplicity: 1 (spin = 0).

Part 3: method – B3LYP, basis set – aug-cc-pvdz for B, H, F and aug-cc-pvdz-PP for Cu, Ag, Au. Spin multiplicity: 1 (spin = 0).

Part 4: method – B3LYP, basis set – aug-cc-pvdz for B, H, F and aug-cc-pvdz-PP for Cu, Ag, Au. SCRF(Solvent=Water). Spin multiplicity: 1 (spin = 0).

Part 5: method – B3LYP, basis set – aug-cc-pvtz for B, H, F and aug-cc-pvtz-PP for Cu, Ag, Au. Spin multiplicity: 1 (spin = 0).

Part 6: method – B3LYP, basis set – aug-cc-pvtz for B, H, F and aug-cc-pvtz-PP for Cu, Ag, Au. SCRF(Solvent=Water). Spin multiplicity: 1 (spin = 0).

Part 1. $[Cu(B_{11}H_{11})_2]^{3-}$: η^5,η^5; PG D_{5d}

Cu	0.00000000	0.00000000	0.00000000
B	0.00000000	1.56791500	1.54117800
B	-1.49150500	0.48445400	1.54136600
B	-0.92189400	-1.26874700	1.54134600
B	0.92189400	-1.26874700	1.54134600
B	1.49150500	0.48445400	1.54136600
B	0.00000000	-1.51876200	3.03643600
B	1.44440000	-0.46929500	3.03654800
B	0.89264000	1.22862700	3.03644900
B	-0.89264000	1.22862700	3.03644900
B	-1.44440000	-0.46929500	3.03654800
B	0.00000000	-0.00003900	3.97295600
B	0.00000000	-1.56791500	-1.54117800
B	1.49150500	-0.48445400	-1.54136600
B	0.92189400	1.26874700	-1.54134600
B	-0.92189400	1.26874700	-1.54134600
B	-1.49150500	-0.48445400	-1.54136600
B	0.00000000	1.51876200	-3.03643600
B	-1.44440000	0.46929500	-3.03654800
B	-0.89264000	-1.22862700	-3.03644900
B	0.89264000	-1.22862700	-3.03644900
B	1.44440000	0.46929500	-3.03654800
B	0.00000000	0.00003900	-3.97295600
H	0.00000000	2.66463800	1.08000900
H	-2.53456400	0.82338300	1.08023800
H	-1.56655200	-2.15612100	1.08043000
H	1.56655200	-2.15612100	1.08043000
H	2.53456400	0.82338300	1.08023800
H	0.00000000	-2.59319400	3.57508500
H	2.46622000	-0.80128300	3.57526100
H	1.52410900	2.09792200	3.57506800
H	-1.52410900	2.09792200	3.57506800
H	-2.46622000	-0.80128300	3.57526100
H	0.00000000	-0.00000500	5.17603500
H	0.00000000	-2.66463800	-1.08000900
H	2.53456400	-0.82338300	-1.08023800
H	1.56655200	2.15612100	-1.08043000
H	-1.56655200	2.15612100	-1.08043000
H	-2.53456400	-0.82338300	-1.08023800
H	0.00000000	2.59319400	-3.57508500
H	-2.46622000	0.80128300	-3.57526100
H	-1.52410900	-2.09792200	-3.57506800
H	1.52410900	-2.09792200	-3.57506800
H	2.46622000	0.80128300	-3.57526100
H	0.00000000	0.00000500	-5.17603500

E(RB3LYP) = -757.710273054

Zero-point correction= 0.302169 (Hartree/Particle)
Thermal correction to Energy= 0.320522
Thermal correction to Enthalpy= 0.321466
Thermal correction to Gibbs Free Energy= 0.261069
Sum of electronic and zero-point Energies= -757.408104
Sum of electronic and thermal Energies= -757.389751
Sum of electronic and thermal Enthalpies= -757.388807
Sum of electronic and thermal Free Energies= -757.449204

	E (Thermal)	CV	S
	KCal/Mol	Cal/Mol-Kelvin	Cal/Mol-Kelvin
Total	201.131	95.059	127.116

	1	2	3
	A(A1U)	A(E1U)	A(E1U)
Frequencies --	68.5701	95.3928	95.9694

Part 1. $[Cu(B_{11}H_{11})_2]^{3-}$: η^2,η^2; PG C_2

Cu	0.00000000	0.00000000	0.10101700
B	-0.66673000	4.53796600	-1.26091900
B	-1.19818800	2.95609200	-1.46834500
B	-1.50770300	1.52407800	-0.31146800
B	-0.50640800	2.07967500	0.89180100
B	0.00585800	3.64637200	-0.00542300
B	-2.26882400	2.30070900	1.10477600
B	-1.09982600	3.60178000	1.45862600
B	-1.28388200	4.97289700	0.28005100
B	-2.35545300	4.36588700	-1.02243200
B	-2.77905500	2.65466800	-0.57142500
B	-2.69526200	3.96016100	0.68001700
B	0.66673000	-4.53796600	-1.26091900
B	-0.00585800	-3.64637200	-0.00542300
B	0.50640800	-2.07967500	0.89180100
B	1.50770300	-1.52407800	-0.31146800
B	1.19818800	-2.95609200	-1.46834500
B	2.26882400	-2.30070900	1.10477600
B	2.77905500	-2.65466800	-0.57142500
B	2.35545300	-4.36588700	-1.02243200
B	1.28388200	-4.97289700	0.28005100
B	1.09982600	-3.60178000	1.45862600
B	2.69526200	-3.96016100	0.68001700
H	-0.06737300	5.19346600	-2.06401700
H	-0.90877600	2.47219200	-2.52505900
H	-1.70135000	0.40912500	-0.76914200
H	0.28753800	1.52505700	1.61405100
H	1.20266400	3.67707800	0.04375800
H	-2.90684300	1.69343000	1.91533400
H	-0.76901900	3.95019200	2.55718700
H	-1.19237900	6.09024000	0.71574000
H	-3.16149200	4.97300200	-1.67659300
H	-3.78336700	2.25660000	-1.09025500
H	-3.64399200	4.43976300	1.23735700
H	0.06737300	-5.19346600	-2.06401700
H	-1.20266400	-3.67707800	0.04375800
H	-0.28753800	-1.52505700	1.61405100
H	1.70135000	-0.40912500	-0.76914200
H	0.90877600	-2.47219200	-2.52505900
H	2.90684300	-1.69343000	1.91533400
H	3.78336700	-2.25660000	-1.09025500
H	3.16149200	-4.97300200	-1.67659300
H	1.19237900	-6.09024000	0.71574000
H	0.76901900	-3.95019200	2.55718700
H	3.64399200	-4.43976300	1.23735700

E(RB3LYP) = -757.728974251

Zero-point correction= 0.296238 (Hartree/Particle)
Thermal correction to Energy= 0.317606
Thermal correction to Enthalpy= 0.318550
Thermal correction to Gibbs Free Energy= 0.246807
Sum of electronic and zero-point Energies= -757.432736
Sum of electronic and thermal Energies= -757.411369
Sum of electronic and thermal Enthalpies= -757.410424
Sum of electronic and thermal Free Energies= -757.482167

	E (Thermal)	CV	S
	KCal/Mol	Cal/Mol-Kelvin	Cal/Mol-Kelvin
Total	199.301	97.895	150.995

	1	2	3
	A(A)	A(A)	A(B)
Frequencies --	20.6272	31.1711	31.6604

Part 1. $[Cu(B_{11}H_{11})_2]^{3-}$: η^2,η^2; PG C_{2h}

Cu	0.00000000	0.00000000	0.00000000
B	-1.59061600	4.25514000	0.00000000
B	-0.85401900	3.13891700	1.02004200
B	0.62943100	1.98410000	0.83605500
B	0.62943100	1.98410000	-0.83605500
B	-0.85401900	3.13891700	-1.02004200
B	1.81610400	3.01968500	0.00000000
B	0.86942900	3.60793300	-1.39651300
B	-0.28375900	4.91618600	-0.89629900
B	-0.28375900	4.91618600	0.89629900
B	0.86942900	3.60793300	1.39651300
B	1.23992100	4.69227200	0.00000000
B	1.59061600	-4.25514000	0.00000000
B	0.85401900	-3.13891700	1.02004200
B	-0.62943100	-1.98410000	0.83605500
B	-0.62943100	-1.98410000	-0.83605500
B	0.85401900	-3.13891700	-1.02004200
B	-1.81610400	-3.01968500	0.00000000
B	-0.86942900	-3.60793300	-1.39651300
B	0.28375900	-4.91618600	-0.89629900
B	0.28375900	-4.91618600	0.89629900
B	-0.86942900	-3.60793300	1.39651300
B	-1.23992100	-4.69227200	0.00000000
H	-2.75018700	4.55321600	0.00000000
H	-1.60432700	2.59724400	1.77987900
H	0.84290100	1.14518200	1.67246400
H	0.84290100	1.14518200	-1.67246400
H	-1.60432700	2.59724400	-1.77987900
H	2.99231900	2.79148900	0.00000000
H	1.29946700	3.73498500	-2.50827500
H	-0.42329900	5.84642100	-1.64583300
H	-0.42329900	5.84642100	1.64583300
H	1.29946700	3.73498500	2.50827500
H	2.07726000	5.55239800	0.00000000
H	2.75018700	-4.55321600	0.00000000
H	1.60432700	-2.59724400	1.77987900
H	-0.84290100	-1.14518200	1.67246400
H	-0.84290100	-1.14518200	-1.67246400
H	1.60432700	-2.59724400	-1.77987900
H	-2.99231900	-2.79148900	0.00000000
H	-1.29946700	-3.73498500	-2.50827500
H	0.42329900	-5.84642100	-1.64583300
H	0.42329900	-5.84642100	1.64583300
H	-1.29946700	-3.73498500	2.50827500
H	-2.07726000	-5.55239800	0.00000000

E(RB3LYP) = -757.723590106

Zero-point correction= 0.296537 (Hartree/Particle)
Thermal correction to Energy= 0.316967
Thermal correction to Enthalpy= 0.317911
Thermal correction to Gibbs Free Energy= 0.248500
Sum of electronic and zero-point Energies= -757.427053
Sum of electronic and thermal Energies= -757.406623
Sum of electronic and thermal Enthalpies= -757.405679
Sum of electronic and thermal Free Energies= -757.475090

	E (Thermal)	CV	S
	KCal/Mol	Cal/Mol-Kelvin	Cal/Mol-Kelvin
Total	198.900	96.147	146.089

	1	2	3
	A(Au)	A(Bu)	A(Bg)
Frequencies --	-24.7748	20.0983	37.7459

Part 1. $[Cu(B_{11}H_{11})_2]^{3-}$: η^2,η^2; PG C_{2v}

Cu	0.37664700	-0.00051700	0.00000000
B	-1.94528500	4.09289700	0.00000000
B	-1.08145700	3.06784200	1.01507900
B	0.52312200	2.10186500	0.83438900
B	0.52312200	2.10186500	-0.83438900
B	-1.08145700	3.06784200	-1.01507900
B	1.58483500	3.27162500	0.00000000
B	0.57636500	3.74169800	-1.39588300
B	-0.72560500	4.90232700	-0.89659100
B	-0.72560500	4.90232700	0.89659100
B	0.57636500	3.74169800	1.39588300
B	0.81382200	4.86350000	0.00000000
H	-3.13154400	4.25221600	0.00000000
H	-1.76076300	2.43946900	1.77473700
H	0.82849300	1.29951000	1.67704700
H	0.82849300	1.29951000	-1.67704700
H	-1.76076300	2.43946900	-1.77473700
H	2.77996600	3.18098700	0.00000000
H	0.98658800	3.91877900	-2.50839400
H	-0.97485600	5.80834700	-1.64728500
H	-0.97485600	5.80834700	1.64728500
H	0.98658800	3.91877900	2.50839400
H	1.54214100	5.81778100	0.00000000
B	-1.94605300	-4.08989700	0.00000000
B	-1.08067900	-3.06619900	1.01516200
B	0.52539700	-2.10249500	0.83444400
B	0.52539700	-2.10249500	-0.83444400
B	-1.08067900	-3.06619900	-1.01516200
B	1.58525200	-3.27379600	0.00000000
B	0.57604400	-3.74245000	-1.39590000
B	-0.72755400	-4.90116700	-0.89659100
B	-0.72755400	-4.90116700	0.89659100
B	0.57604400	-3.74245000	1.39590000
B	0.81192000	-4.86454900	0.00000000
H	-3.13254800	-4.24739600	0.00000000
H	-1.75910800	-2.43678500	1.77473300
H	0.83219400	-1.30070700	1.67708100
H	0.83219400	-1.30070700	-1.67708100
H	-1.75910800	-2.43678500	-1.77473300
H	2.78053400	-3.18504400	0.00000000
H	0.98610500	-3.92014000	-2.50837200
H	-0.97814200	-5.80686800	-1.64722300
H	-0.97814200	-5.80686800	1.64722300
H	0.98610500	-3.92014000	2.50837200
H	1.53886600	-5.81987600	0.00000000

E(RB3LYP) = -757.721457229

Zero-point correction= 0.296511 (Hartree/Particle)
Thermal correction to Energy= 0.316158
Thermal correction to Enthalpy= 0.317102
Thermal correction to Gibbs Free Energy= 0.249793
Sum of electronic and zero-point Energies= -757.424946
Sum of electronic and thermal Energies= -757.405299
Sum of electronic and thermal Enthalpies= -757.404355
Sum of electronic and thermal Free Energies= -757.471664

	E (Thermal)	CV	S
	KCal/Mol	Cal/Mol-Kelvin	Cal/Mol-Kelvin
Total	198.392	94.148	141.663

	1	2	3
	A"(A2)	A"(A2)	A'(A1)
Frequencies --	-54.1906	-23.7355	25.8969

Part 1. $[Cu(B_{11}H_{11})_2]^{3-}$: η^5,η^2; PG C_s

Au	0.15605800	-0.19373400	0.00000000
B	-1.96329500	3.18552800	0.00000000
B	-0.89208000	2.45148800	1.08591000
B	0.96183200	1.86266400	0.86624600
B	0.96183200	1.86266400	-0.86624600
B	-0.89208000	2.45148800	-1.08591000
B	1.63301300	3.26691500	0.00000000
B	0.54223000	3.48982500	-1.39485600
B	-0.99717900	4.29208800	-0.89510300
B	-0.99717900	4.29208800	0.89510300
B	0.54223000	3.48982500	1.39485600
B	0.50209700	4.62711400	0.00000000
H	-3.15235600	3.05130600	0.00000000
H	-1.41688900	1.72823900	1.87667800
H	1.56954700	1.30375500	1.72864100
H	1.56954700	1.30375500	-1.72864100
H	-1.41688900	1.72823900	-1.87667800
H	2.81148300	3.49441100	0.00000000
H	0.93079100	3.76841800	-2.49418500
H	-1.47024300	5.11006100	-1.63933500
H	-1.47024300	5.11006100	1.63933500
H	0.93079100	3.76841800	2.49418500
H	0.99066500	5.72404800	0.00000000
B	-1.40918200	-1.86566700	0.00000000
B	-0.52257300	-2.11586200	1.64586900
B	1.09041400	-2.15110500	1.04917100
B	1.09041400	-2.15110500	-1.04917100
B	-0.52257300	-2.11586200	-1.64586900
B	1.30492500	-3.56731100	0.00000000
B	0.25394400	-3.67789400	-1.47022900
B	-1.39385100	-3.42467300	-0.91769100
B	-1.39385100	-3.42467300	0.91769100
B	0.25394400	-3.67789400	1.47022900
B	-0.27041000	-4.48521500	0.00000000
H	-2.33024600	-1.10156900	0.00000000
H	-0.91610600	-1.63256900	2.66451200
H	2.06069400	-1.67625500	1.55736200
H	2.06069400	-1.67625500	-1.55736200
H	-0.91610600	-1.63256900	-2.66451200
H	2.37757200	-4.10945400	0.00000000
H	0.59309300	-4.32489700	-2.42348800
H	-2.38625300	-3.84858100	-1.44397600
H	-2.38625300	-3.84858100	1.44397600
H	0.59309300	-4.32489700	2.42348800
H	-0.36808700	-5.68221300	0.00000000

E(RB3LYP) = -696.109891802

Zero-point correction= 0.296884 (Hartree/Particle)
Thermal correction to Energy= 0.316989
Thermal correction to Enthalpy= 0.317933
Thermal correction to Gibbs Free Energy= 0.250164
Sum of electronic and zero-point Energies= -695.813008
Sum of electronic and thermal Energies= -695.792903
Sum of electronic and thermal Enthalpies= -695.791959
Sum of electronic and thermal Free Energies= -695.859728

	E (Thermal)	CV	S
	KCal/Mol	Cal/Mol-Kelvin	Cal/Mol-Kelvin
Total	198.913	96.493	142.631

	1	2	3
	A"	A"	A'
Frequencies --	-44.1022	31.1515	33.2911

Part 1. $[Cu(B_{11}H_{11})_2]^{3-}$: η^5,η^2; PG C_s

Au	-0.19753600	-0.22127000	0.00000000
B	1.97984800	3.30587600	0.00000000
B	0.92463600	2.53273500	1.06887600
B	-0.90181700	1.90174400	0.86254800
B	-0.90181700	1.90174400	-0.86254800
B	0.92463600	2.53273500	-1.06887600
B	-1.62161700	3.28304500	0.00000000
B	-0.53512100	3.53668800	-1.39421700
B	0.98050800	4.38246000	-0.89559800
B	0.98050800	4.38246000	0.89559800
B	-0.53512100	3.53668800	1.39421700
B	-0.52695800	4.67533900	0.00000000
H	3.17169700	3.20147800	0.00000000
H	1.46763700	1.80914200	1.84695600
H	-1.48048600	1.31818500	1.73044000
H	-1.48048600	1.31818500	-1.73044000
H	1.46763700	1.80914200	-1.84695600
H	-2.80627200	3.47550400	0.00000000
H	-0.92516100	3.80100600	-2.49642400
H	1.42955600	5.21335900	-1.64047800
H	1.42955600	5.21335900	1.64047800
H	-0.92516100	3.80100600	2.49642400
H	-1.04711700	5.75763200	0.00000000
B	-1.32141900	-2.21700700	0.00000000
B	-0.39418800	-2.26119100	1.65390400
B	1.16998400	-1.91724100	1.03829200
B	1.16998400	-1.91724100	-1.03829200
B	-0.39418800	-2.26119100	-1.65390400
B	1.71380000	-3.25235400	0.00000000
B	0.72112300	-3.59988100	-1.46898900
B	-0.94328300	-3.72821200	-0.92173600
B	-0.94328300	-3.72821200	0.92173600
B	0.72112300	-3.59988100	1.46898900
B	0.38916100	-4.50610700	0.00000000
H	-2.39693600	-1.69264100	0.00000000
H	-0.88992900	-1.87668700	2.67049500
H	2.00182500	-1.22167000	1.53787600
H	2.00182500	-1.22167000	-1.53787600
H	-0.88992900	-1.87668700	-2.67049500
H	2.88190500	-3.53214900	0.00000000
H	1.19666500	-4.15269100	-2.42288500
H	-1.81953100	-4.36533600	-1.44027100
H	-1.81953100	-4.36533600	1.44027100
H	1.19666500	-4.15269100	2.42288500
H	0.55840700	-5.69504800	0.00000000

E(RB3LYP) = -696.108917304

Zero-point correction= 0.296571 (Hartree/Particle)
Thermal correction to Energy= 0.316872
Thermal correction to Enthalpy= 0.317816
Thermal correction to Gibbs Free Energy= 0.248569
Sum of electronic and zero-point Energies= -695.812346
Sum of electronic and thermal Energies= -695.792046
Sum of electronic and thermal Enthalpies= -695.791101
Sum of electronic and thermal Free Energies= -695.860348

	E (Thermal)	CV	S
	KCal/Mol	Cal/Mol-Kelvin	Cal/Mol-Kelvin
Total	198.840	96.628	145.741

	1	2	3
	A"	A"	A'
Frequencies --	-130.1970	13.5039	35.5037

Part 1. $[Ag(B_{11}H_{11})_2]^{3-}$: η⁵,η⁵; PG D_{5d}

Ag	0.00000000	0.00000000	0.00000000
B	-0.57021600	2.26114000	0.00000000
B	0.38944700	1.73948100	1.50358600
B	1.94262700	0.89543100	0.92960200
B	1.94262700	0.89543100	-0.92960200
B	0.38944700	1.73948100	-1.50358600
B	2.86592300	2.08801000	0.00000000
B	1.94262700	2.59002200	-1.44634300
B	0.44889200	3.40194500	-0.89380300
B	0.44889200	3.40194500	0.89380300
B	1.94262700	2.59002200	1.44634300
B	1.97571900	3.63457300	0.00000000
B	0.57021600	-2.26114000	0.00000000
B	-0.38944700	-1.73948100	-1.50358600
B	-1.94262700	-0.89543100	-0.92960200
B	-1.94262700	-0.89543100	0.92960200
B	-0.38944700	-1.73948100	1.50358600
B	-2.86592300	-2.08801000	0.00000000
B	-1.94262700	-2.59002200	1.44634300
B	-0.44889200	-3.40194500	0.89380300
B	-0.44889200	-3.40194500	-0.89380300
B	-1.94262700	-2.59002200	-1.44634300
B	-1.97571900	-3.63457300	0.00000000
H	-1.75615800	2.37415300	0.00000000
H	-0.13134100	1.49080500	2.54577900
H	2.49847200	0.06163900	1.57391100
H	2.49847200	0.06163900	-1.57391100
H	-0.13134100	1.49080500	-2.54577900
H	4.06700000	2.05719400	0.00000000
H	2.49413100	2.91229900	-2.46394100
H	-0.05069100	4.29549500	-1.52269000
H	-0.05069100	4.29549500	1.52269000
H	2.49413100	2.91229900	2.46394100
H	2.54983300	4.69077900	0.00000000
H	1.75615800	-2.37415300	0.00000000
H	0.13134100	-1.49080500	-2.54577900
H	-2.49847200	-0.06163900	-1.57391100
H	-2.49847200	-0.06163900	1.57391100
H	0.13134100	-1.49080500	2.54577900
H	-4.06700000	-2.05719400	0.00000000
H	-2.49413100	-2.91229900	2.46394100
H	0.05069100	-4.29549500	1.52269000
H	0.05069100	-4.29549500	-1.52269000
H	-2.49413100	-2.91229900	-2.46394100
H	-2.54983300	-4.69077900	0.00000000

E(RB3LYP) = -707.340895712
Zero-point correction= 0.300733 (Hartree/Particle)
Thermal correction to Energy= 0.319696
Thermal correction to Enthalpy= 0.320640
Thermal correction to Gibbs Free Energy= 0.258638
Sum of electronic and zero-point Energies= -707.040163
Sum of electronic and thermal Energies= -707.021200
Sum of electronic and thermal Enthalpies= -707.020256
Sum of electronic and thermal Free Energies= -707.082258

	E (Thermal)	CV	S
	KCal/Mol	Cal/Mol-Kelvin	Cal/Mol-Kelvin
Total	200.612	96.030	130.495

	1	2	3
	AU(A1U)	BU(E1U)	AU(E1U)
Frequencies --	34.4032	80.5953	81.0367

Part 1. $[Ag(B_{11}H_{11})_2]^{3-}$: η^2,η^2; PG C_2

Ag	0.00000000	0.00000000	0.11395800
B	0.50634200	4.69099200	-1.23947500
B	1.12258400	3.13758300	-1.44281300
B	1.63503900	1.75953500	-0.29311900
B	0.68919600	2.29476200	0.97488800
B	0.00000000	3.81190900	0.09840900
B	2.44052500	2.63278300	1.03456400
B	1.22625700	3.87571600	1.45074500
B	1.22117900	5.21836700	0.23313100
B	2.21594800	4.64203300	-1.14113100
B	2.79159000	2.97876600	-0.68517000
B	2.72301400	4.30851100	0.53407700
B	-0.50634200	-4.69099200	-1.23947500
B	0.00000000	-3.81190900	0.09840900
B	-0.68919600	-2.29476200	0.97488800
B	-1.63503900	-1.75953500	-0.29311900
B	-1.12258400	-3.13758300	-1.44281300
B	-2.44052500	-2.63278300	1.03456400
B	-2.79159000	-2.97876600	-0.68517000
B	-2.21594800	-4.64203300	-1.14113100
B	-1.22117900	-5.21836700	0.23313100
B	-1.22625700	-3.87571600	1.45074500
B	-2.72301400	-4.30851100	0.53407700
H	-0.19956700	5.27786700	-2.00760300
H	0.76941400	2.60759900	-2.45723800
H	1.93072100	0.67292700	-0.74820300
H	0.00582000	1.74648100	1.80195600
H	-1.18775200	3.77193800	0.25239200
H	3.18489000	2.10026000	1.80724100
H	0.96458900	4.22402800	2.56764000
H	1.09133600	6.34144300	0.64258900
H	2.92076900	5.27748100	-1.87930100
H	3.77366600	2.63195300	-1.27747600
H	3.68332900	4.85740400	0.99946600
H	0.19956700	-5.27786700	-2.00760300
H	1.18775200	-3.77193800	0.25239200
H	-0.00582000	-1.74648100	1.80195600
H	-1.93072100	-0.67292700	-0.74820300
H	-0.76941400	-2.60759900	-2.45723800
H	-3.18489000	-2.10026000	1.80724100
H	-3.77366600	-2.63195300	-1.27747600
H	-2.92076900	-5.27748100	-1.87930100
H	-1.09133600	-6.34144300	0.64258900
H	-0.96458900	-4.22402800	2.56764000
H	-3.68332900	-4.85740400	0.99946600

E(RB3LYP) = -707.374045157

Zero-point correction= 0.296155 (Hartree/Particle)
Thermal correction to Energy= 0.317713
Thermal correction to Enthalpy= 0.318657
Thermal correction to Gibbs Free Energy= 0.245107
Sum of electronic and zero-point Energies= -707.077890
Sum of electronic and thermal Energies= -707.056333
Sum of electronic and thermal Enthalpies= -707.055388
Sum of electronic and thermal Free Energies= -707.128938

	E (Thermal)	CV	S
	KCal/Mol	Cal/Mol-Kelvin	Cal/Mol-Kelvin
Total	199.368	98.017	154.798

	1	2	3
	A	A	B
Frequencies --	17.8924	25.2896	26.1185

Part 1. $[Ag(B_{11}H_{11})_2]^{3-}$: η^2,η^2; PG C_{2h}

Ag	0.00000000	0.00000000	0.00000000
B	-1.63165400	4.42548000	0.00000000
B	-0.85089200	3.33783300	1.01783000
B	0.67390100	2.24403100	0.83822300
B	0.67390100	2.24403100	-0.83822300
B	-0.85089200	3.33783300	-1.01783000
B	1.81864900	3.31980100	0.00000000
B	0.85089200	3.87799800	-1.39669500
B	-0.35170400	5.13921500	-0.89588700
B	-0.35170400	5.13921500	0.89588700
B	0.85089200	3.87799800	1.39669500
B	1.17842000	4.97365400	0.00000000
B	1.63165400	-4.42548000	0.00000000
B	0.85089200	-3.33783300	1.01783000
B	-0.67390100	-2.24403100	0.83822300
B	-0.67390100	-2.24403100	-0.83822300
B	0.85089200	-3.33783300	-1.01783000
B	-1.81864900	-3.31980100	0.00000000
B	-0.85089200	-3.87799800	-1.39669500
B	0.35170400	-5.13921500	-0.89588700
B	0.35170400	-5.13921500	0.89588700
B	-0.85089200	-3.87799800	1.39669500
B	-1.17842000	-4.97365400	0.00000000
H	-2.80189100	4.67643600	0.00000000
H	-1.58236000	2.76705300	1.77540400
H	0.92994000	1.43299100	1.69070400
H	0.92994000	1.43299100	-1.69070400
H	-1.58236000	2.76705300	-1.77540400
H	3.00316100	3.13863600	0.00000000
H	1.27483000	4.02089000	-2.50861900
H	-0.52917600	6.06263500	-1.64523300
H	-0.52917600	6.06263500	1.64523300
H	1.27483000	4.02089000	2.50861900
H	1.98520700	5.86202700	0.00000000
H	2.80189100	-4.67643600	0.00000000
H	1.58236000	-2.76705300	1.77540400
H	-0.92994000	-1.43299100	1.69070400
H	-0.92994000	-1.43299100	-1.69070400
H	1.58236000	-2.76705300	-1.77540400
H	-3.00316100	-3.13863600	0.00000000
H	-1.27483000	-4.02089000	-2.50861900
H	0.52917600	-6.06263500	-1.64523300
H	0.52917600	-6.06263500	1.64523300
H	-1.27483000	-4.02089000	2.50861900
H	-1.98520700	-5.86202700	0.00000000

E(RB3LYP) = -707.372314004

Zero-point correction= 0.296472 (Hartree/Particle)
Thermal correction to Energy= 0.317025
Thermal correction to Enthalpy= 0.317969
Thermal correction to Gibbs Free Energy= 0.248175
Sum of electronic and zero-point Energies= -707.075842
Sum of electronic and thermal Energies= -707.055289
Sum of electronic and thermal Enthalpies= -707.054345
Sum of electronic and thermal Free Energies= -707.124139

	E (Thermal)	CV	S
	KCal/Mol	Cal/Mol-Kelvin	Cal/Mol-Kelvin
Total	198.936	96.075	146.893

	1	2	3
	AU	BU	AU
Frequencies --	-15.1724	20.7628	35.0708

Part 1. $[Ag(B_{11}H_{11})_2]^{3-}$: η^2,η^2; PG C_{2v}

Ag	0.00000000	0.00000000	0.31612300
B	0.00000000	4.25995400	-1.98817100
B	-1.01515100	3.26277000	-1.09223100
B	-0.83765500	2.34794500	0.54052100
B	0.83765500	2.34794500	0.54052100
B	1.01515100	3.26277000	-1.09223100
B	0.00000000	3.54318800	1.56348200
B	1.39629700	3.99070900	0.54107100
B	0.89604600	5.10994000	-0.79477900
B	-0.89604600	5.10994000	-0.79477900
B	-1.39629700	3.99070900	0.54107100
B	0.00000000	5.11598600	0.74411300
H	0.00000000	4.38075600	-3.17863300
H	-1.77292100	2.61378100	-1.75473000
H	-1.69379000	1.57220800	0.88135700
H	1.69379000	1.57220800	0.88135700
H	1.77292100	2.61378100	-1.75473000
H	0.00000000	3.49201500	2.76105400
H	2.50850400	4.17988600	0.94595900
H	1.64580700	6.00769100	-1.07328200
H	-1.64580700	6.00769100	-1.07328200
H	-2.50850400	4.17988600	0.94595900
H	0.00000000	6.08867200	1.44715000
B	0.00000000	-4.25995400	-1.98817100
B	-1.01515100	-3.26277000	-1.09223100
B	-0.83765500	-2.34794500	0.54052100
B	0.83765500	-2.34794500	0.54052100
B	1.01515100	-3.26277000	-1.09223100
B	0.00000000	-3.54318800	1.56348200
B	1.39629700	-3.99070900	0.54107100
B	0.89604600	-5.10994000	-0.79477900
B	-0.89604600	-5.10994000	-0.79477900
B	-1.39629700	-3.99070900	0.54107100
B	0.00000000	-5.11598600	0.74411300
H	0.00000000	-4.38075600	-3.17863300
H	-1.77292100	-2.61378100	-1.75473000
H	-1.69379000	-1.57220800	0.88135700
H	1.69379000	-1.57220800	0.88135700
H	1.77292100	-2.61378100	-1.75473000
H	0.00000000	-3.49201500	2.76105400
H	2.50850400	-4.17988600	0.94595900
H	1.64580700	-6.00769100	-1.07328200
H	-1.64580700	-6.00769100	-1.07328200
H	-2.50850400	-4.17988600	0.94595900
H	0.00000000	-6.08867200	1.44715000

E(RB3LYP) = -707.371080360

Zero-point correction= 0.296474 (Hartree/Particle)
Thermal correction to Energy= 0.317076
Thermal correction to Enthalpy= 0.318020
Thermal correction to Gibbs Free Energy= 0.247783
Sum of electronic and zero-point Energies= -707.074606
Sum of electronic and thermal Energies= -707.054004
Sum of electronic and thermal Enthalpies= -707.053060
Sum of electronic and thermal Free Energies= -707.123298

	E (Thermal)	CV	S
	KCal/Mol	Cal/Mol-Kelvin	Cal/Mol-Kelvin
Total	198.968	96.033	147.827

	1	2	3
	A2	A1	B2
Frequencies --	-13.1947	23.8175	30.7932

Part 1. $[Au(B_{11}H_{11})_2]^{3-}$: η^5,η^5; PG D_{5d}

Au	0.00000000	0.00000000	0.00000000
B	-0.58196700	2.24298800	0.00000000
B	0.38081700	1.71457700	1.51182200
B	1.93887200	0.85953700	0.93461900
B	1.93887200	0.85953700	-0.93461900
B	0.38081700	1.71457700	-1.51182200
B	2.85879600	2.05204100	0.00000000
B	1.93887200	2.55710700	-1.44431300
B	0.45056600	3.37398700	-0.89253900
B	0.45056600	3.37398700	0.89253900
B	1.93887200	2.55710700	1.44431300
B	1.97699800	3.60153800	0.00000000
B	0.58196700	-2.24298800	0.00000000
B	-0.38081700	-1.71457700	-1.51182200
B	-1.93887200	-0.85953700	-0.93461900
B	-1.93887200	-0.85953700	0.93461900
B	-0.38081700	-1.71457700	1.51182200
B	-2.85879600	-2.05204100	0.00000000
B	-1.93887200	-2.55710700	1.44431300
B	-0.45056600	-3.37398700	0.89253900
B	-0.45056600	-3.37398700	-0.89253900
B	-1.93887200	-2.55710700	-1.44431300
B	-1.97699800	-3.60153800	0.00000000
H	-1.76466500	2.38203300	0.00000000
H	-0.13355100	1.48660900	2.56137300
H	2.50632600	0.03787200	1.58346500
H	2.50632600	0.03787200	-1.58346500
H	-0.13355100	1.48660900	-2.56137300
H	4.05970600	2.01989600	0.00000000
H	2.49275200	2.88016600	-2.46021500
H	-0.04253000	4.27171100	-1.52035300
H	-0.04253000	4.27171100	1.52035300
H	2.49275200	2.88016600	2.46021500
H	2.55544000	4.65539600	0.00000000
H	1.76466500	-2.38203300	0.00000000
H	0.13355100	-1.48660900	-2.56137300
H	-2.50632600	-0.03787200	-1.58346500
H	-2.50632600	-0.03787200	1.58346500
H	0.13355100	-1.48660900	2.56137300
H	-4.05970600	-2.01989600	0.00000000
H	-2.49275200	-2.88016600	2.46021500
H	0.04253000	-4.27171100	1.52035300
H	0.04253000	-4.27171100	-1.52035300
H	-2.49275200	-2.88016600	-2.46021500
H	-2.55544000	-4.65539600	0.00000000

E(RB3LYP) = -696.164254083

Zero-point correction= 0.302715 (Hartree/Particle)
Thermal correction to Energy= 0.321197
Thermal correction to Enthalpy= 0.322142
Thermal correction to Gibbs Free Energy= 0.260857
Sum of electronic and zero-point Energies= -695.861539
Sum of electronic and thermal Energies= -695.843057
Sum of electronic and thermal Enthalpies= -695.842112
Sum of electronic and thermal Free Energies= -695.903397

	E (Thermal)	CV	S
	KCal/Mol	Cal/Mol-Kelvin	Cal/Mol-Kelvin
Total	201.554	94.668	128.984

	1	2	3
	AU(A1U)	BU(E1U)	AU(E1U)
Frequencies --	36.6101	83.2242	83.6799

Part 1. $[Au(B_{11}H_{11})_2]^{3-}$: η^2,η^2; PG C_2

Au	0.00000000	0.00000000	0.16447200
B	-1.44790300	4.02395800	-0.70713900
B	-0.33190200	2.92898600	-1.33533400
B	1.14225000	1.95884600	-0.56720800
B	0.62439700	2.13420600	1.05275200
B	-0.96292200	3.14423600	0.64406600
B	1.88690100	3.22630000	0.43820800
B	0.51376200	3.84831800	1.40375100
B	-0.53908500	4.94305300	0.42895700
B	0.00000000	4.76071800	-1.27006700
B	1.35425300	3.56499000	-1.23671900
B	1.19547700	4.81357200	0.04811200
B	1.44790300	-4.02395800	-0.70713900
B	0.96292200	-3.14423600	0.64406600
B	-0.62439700	-2.13420600	1.05275200
B	-1.14225000	-1.95884600	-0.56720800
B	0.33190200	-2.92898600	-1.33533400
B	-1.88690100	-3.22630000	0.43820800
B	-1.35425300	-3.56499000	-1.23671900
B	0.00000000	-4.76071800	-1.27006700
B	0.53908500	-4.94305300	0.42895700
B	-0.51376200	-3.84831800	1.40375100
B	-1.19547700	-4.81357200	0.04811200
H	-2.57477800	4.17079600	-1.08133700
H	-0.77170100	2.23716600	-2.20470600
H	1.76494000	1.16736300	-1.21638800
H	0.70927300	1.51976800	2.07715200
H	-1.86092300	2.60833700	1.22442200
H	3.02585600	3.16749000	0.80804900
H	0.59110300	4.12654800	2.56685600
H	-0.98268700	5.92046400	0.97094500
H	0.00694000	5.58558800	-2.14467600
H	2.09790800	3.62315300	-2.17394400
H	1.92806400	5.75487500	0.18009300
H	2.57477800	-4.17079600	-1.08133700
H	1.86092300	-2.60833700	1.22442200
H	-0.70927300	-1.51976800	2.07715200
H	-1.76494000	-1.16736300	-1.21638800
H	0.77170100	-2.23716600	-2.20470600
H	-3.02585600	-3.16749000	0.80804900
H	-2.09790800	-3.62315300	-2.17394400
H	-0.00694000	-5.58558800	-2.14467600
H	0.98268700	-5.92046400	0.97094500
H	-0.59110300	-4.12654800	2.56685600
H	-1.92806400	-5.75487500	0.18009300

E(RB3LYP) = -696.131807979

Zero-point correction= 0.297309 (Hartree/Particle)
Thermal correction to Energy= 0.318332
Thermal correction to Enthalpy= 0.319276
Thermal correction to Gibbs Free Energy= 0.246928
Sum of electronic and zero-point Energies= -695.834499
Sum of electronic and thermal Energies= -695.813476
Sum of electronic and thermal Enthalpies= -695.812532
Sum of electronic and thermal Free Energies= -695.884880

	E (Thermal)	CV	S
	KCal/Mol	Cal/Mol-Kelvin	Cal/Mol-Kelvin
Total	199.756	98.041	152.270

	1	2	3
	A	B	A
Frequencies --	3.8033	23.6084	34.4808

Part 1. $[Au(B_{11}H_{11})_2]^{3-}$: η^2,η^2; PG C_{2h}

Au	0.00000000	0.00000000	0.00000000
B	-1.73272500	3.98651200	0.00000000
B	-0.83289100	3.01977200	1.04547100
B	0.83289100	2.07076500	0.85510700
B	0.83289100	2.07076500	-0.85510700
B	-0.83289100	3.01977200	-1.04547100
B	1.81306300	3.28489200	0.00000000
B	0.78451600	3.73911100	-1.39258900
B	-0.54726300	4.85314900	-0.89588700
B	-0.54726300	4.85314900	0.89588700
B	0.78451600	3.73911100	1.39258900
B	0.99126600	4.85820900	0.00000000
B	1.73272500	-3.98651200	0.00000000
B	0.83289100	-3.01977200	1.04547100
B	-0.83289100	-2.07076500	0.85510700
B	-0.83289100	-2.07076500	-0.85510700
B	0.83289100	-3.01977200	-1.04547100
B	-1.81306300	-3.28489200	0.00000000
B	-0.78451600	-3.73911100	-1.39258900
B	0.54726300	-4.85314900	-0.89588700
B	0.54726300	-4.85314900	0.89588700
B	-0.78451600	-3.73911100	1.39258900
B	-0.99126600	-4.85820900	0.00000000
H	-2.92385400	4.10020600	0.00000000
H	-1.49668800	2.38431200	1.81006300
H	1.24804300	1.37624000	1.73796800
H	1.24804300	1.37624000	-1.73796800
H	-1.49668800	2.38431200	-1.81006300
H	3.01166900	3.26019100	0.00000000
H	1.20511200	3.92043500	-2.49974300
H	-0.83144100	5.75130600	-1.64301000
H	-0.83144100	5.75130600	1.64301000
H	1.20511200	3.92043500	2.49974300
H	1.70255200	5.82471100	0.00000000
H	2.92385400	-4.10020600	0.00000000
H	1.49668800	-2.38431200	1.81006300
H	-1.24804300	-1.37624000	1.73796800
H	-1.24804300	-1.37624000	-1.73796800
H	1.49668800	-2.38431200	-1.81006300
H	-3.01166900	-3.26019100	0.00000000
H	-1.20511200	-3.92043500	-2.49974300
H	0.83144100	-5.75130600	-1.64301000
H	0.83144100	-5.75130600	1.64301000
H	-1.20511200	-3.92043500	2.49974300
H	-1.70255200	-5.82471100	0.00000000

E(RB3LYP) = -696.131807168
Zero-point correction= 0.297350 (Hartree/Particle)
Thermal correction to Energy= 0.318358
Thermal correction to Enthalpy= 0.319302
Thermal correction to Gibbs Free Energy= 0.248214
Sum of electronic and zero-point Energies= -695.834457
Sum of electronic and thermal Energies= -695.813450
Sum of electronic and thermal Enthalpies= -695.812505
Sum of electronic and thermal Free Energies= -695.883593

	E (Thermal)	CV	S
	KCal/Mol	Cal/Mol-Kelvin	Cal/Mol-Kelvin
Total	199.772	98.038	149.616

	1	2	3
	AU	BU	AU
Frequencies --	16.0301	21.8122	37.5037

Part 1. $[Au(B_{11}H_{11})_2]^{3-}$: η^2,η^2; PG C_{2v}

Au	0.00000000	0.00000000	0.45084800
B	0.00000000	3.62669200	-2.18945300
B	-1.03916400	2.84295100	-1.12124700
B	-0.85404200	2.23134300	0.68429900
B	0.85404200	2.23134300	0.68429900
B	1.03916400	2.84295100	-1.12124700
B	0.00000000	3.60764600	1.42564700
B	1.39227200	3.86062000	0.33058800
B	0.89623900	4.70008700	-1.18809600
B	-0.89623900	4.70008700	-1.18809600
B	-1.39227200	3.86062000	0.33058800
B	0.00000000	4.99764400	0.32172400
H	0.00000000	3.51391400	-3.37970900
H	-1.80048300	2.08938200	-1.65092900
H	-1.74347500	1.63216400	1.21929900
H	1.74347500	1.63216400	1.21929900
H	1.80048300	2.08938200	-1.65092900
H	0.00000000	3.80594100	2.60876800
H	2.50007600	4.11684400	0.70864300
H	1.64398000	5.52764400	-1.63700400
H	-1.64398000	5.52764400	-1.63700400
H	-2.50007600	4.11684400	0.70864300
H	0.00000000	6.08141700	0.83738400
B	0.00000000	-3.62669200	-2.18945300
B	-1.03916400	-2.84295100	-1.12124700
B	-0.85404200	-2.23134300	0.68429900
B	0.85404200	-2.23134300	0.68429900
B	1.03916400	-2.84295100	-1.12124700
B	0.00000000	-3.60764600	1.42564700
B	1.39227200	-3.86062000	0.33058800
B	0.89623900	-4.70008700	-1.18809600
B	-0.89623900	-4.70008700	-1.18809600
B	-1.39227200	-3.86062000	0.33058800
B	0.00000000	-4.99764400	0.32172400
H	0.00000000	-3.51391400	-3.37970900
H	-1.80048300	-2.08938200	-1.65092900
H	-1.74347500	-1.63216400	1.21929900
H	1.74347500	-1.63216400	1.21929900
H	1.80048300	-2.08938200	-1.65092900
H	0.00000000	-3.80594100	2.60876800
H	2.50007600	-4.11684400	0.70864300
H	1.64398000	-5.52764400	-1.63700400
H	-1.64398000	-5.52764400	-1.63700400
H	-2.50007600	-4.11684400	0.70864300
H	0.00000000	-6.08141700	0.83738400

E(RB3LYP) = -696.129064626

Zero-point correction= 0.297249 (Hartree/Particle)
Thermal correction to Energy= 0.318345
Thermal correction to Enthalpy= 0.319289
Thermal correction to Gibbs Free Energy= 0.246887
Sum of electronic and zero-point Energies= -695.831816
Sum of electronic and thermal Energies= -695.810720
Sum of electronic and thermal Enthalpies= -695.809775
Sum of electronic and thermal Free Energies= -695.882178

	E (Thermal)	CV	S
	KCal/Mol	Cal/Mol-Kelvin	Cal/Mol-Kelvin
Total	199.765	98.042	152.383

	1	2	3
	A2	B2	A1
Frequencies --	9.4206	17.5787	25.6450

Part 1. $[Au(B_{11}H_{11})_2]^{3-}$: η^5,η^2; PG C_s

Au	0.15605800	-0.19373400	0.00000000
B	-1.96329500	3.18552800	0.00000000
B	-0.89208000	2.45148800	1.08591000
B	0.96183200	1.86266400	0.86624600
B	0.96183200	1.86266400	-0.86624600
B	-0.89208000	2.45148800	-1.08591000
B	1.63301300	3.26691500	0.00000000
B	0.54223000	3.48982500	-1.39485600
B	-0.99717900	4.29208800	-0.89510300
B	-0.99717900	4.29208800	0.89510300
B	0.54223000	3.48982500	1.39485600
B	0.50209700	4.62711400	0.00000000
H	-3.15235600	3.05130600	0.00000000
H	-1.41688900	1.72823900	1.87667800
H	1.56954700	1.30375500	1.72864100
H	1.56954700	1.30375500	-1.72864100
H	-1.41688900	1.72823900	-1.87667800
H	2.81148300	3.49441100	0.00000000
H	0.93079100	3.76841800	-2.49418500
H	-1.47024300	5.11006100	-1.63933500
H	-1.47024300	5.11006100	1.63933500
H	0.93079100	3.76841800	2.49418500
H	0.99066500	5.72404800	0.00000000
B	-1.40918200	-1.86566700	0.00000000
B	-0.52257300	-2.11586200	1.64586900
B	1.09041400	-2.15110500	1.04917100
B	1.09041400	-2.15110500	-1.04917100
B	-0.52257300	-2.11586200	-1.64586900
B	1.30492500	-3.56731100	0.00000000
B	0.25394400	-3.67789400	-1.47022900
B	-1.39385100	-3.42467300	-0.91769100
B	-1.39385100	-3.42467300	0.91769100
B	0.25394400	-3.67789400	1.47022900
B	-0.27041000	-4.48521500	0.00000000
H	-2.33024600	-1.10156900	0.00000000
H	-0.91610600	-1.63256900	2.66451200
H	2.06069400	-1.67625500	1.55736200
H	2.06069400	-1.67625500	-1.55736200
H	-0.91610600	-1.63256900	-2.66451200
H	2.37757200	-4.10945400	0.00000000
H	0.59309300	-4.32489700	-2.42348800
H	-2.38625300	-3.84858100	-1.44397600
H	-2.38625300	-3.84858100	1.44397600
H	0.59309300	-4.32489700	2.42348800
H	-0.36808700	-5.68221300	0.00000000

E(RB3LYP) = -696.109891802

Zero-point correction= 0.296884 (Hartree/Particle)
Thermal correction to Energy= 0.316989
Thermal correction to Enthalpy= 0.317933
Thermal correction to Gibbs Free Energy= 0.250164
Sum of electronic and zero-point Energies= -695.813008
Sum of electronic and thermal Energies= -695.792903
Sum of electronic and thermal Enthalpies= -695.791959
Sum of electronic and thermal Free Energies= -695.859728

	E (Thermal)	CV	S
	KCal/Mol	Cal/Mol-Kelvin	Cal/Mol-Kelvin
Total	198.913	96.493	142.631

	1	2	3
	A"	A"	A'
Frequencies --	-44.1022	31.1515	33.2911

Part 1. $[Au(B_{11}H_{11})_2]^{3-}$: η^5,η^2; PG C_s

Au	-0.19753600	-0.22127000	0.00000000
B	1.97984800	3.30587600	0.00000000
B	0.92463600	2.53273500	1.06887600
B	-0.90181700	1.90174400	0.86254800
B	-0.90181700	1.90174400	-0.86254800
B	0.92463600	2.53273500	-1.06887600
B	-1.62161700	3.28304500	0.00000000
B	-0.53512100	3.53668800	-1.39421700
B	0.98050800	4.38246000	-0.89559800
B	0.98050800	4.38246000	0.89559800
B	-0.53512100	3.53668800	1.39421700
B	-0.52695800	4.67533900	0.00000000
H	3.17169700	3.20147800	0.00000000
H	1.46763700	1.80914200	1.84695600
H	-1.48048600	1.31818500	1.73044000
H	-1.48048600	1.31818500	-1.73044000
H	1.46763700	1.80914200	-1.84695600
H	-2.80627200	3.47550400	0.00000000
H	-0.92516100	3.80100600	-2.49642400
H	1.42955600	5.21335900	-1.64047800
H	1.42955600	5.21335900	1.64047800
H	-0.92516100	3.80100600	2.49642400
H	-1.04711700	5.75763200	0.00000000
B	-1.32141900	-2.21700700	0.00000000
B	-0.39418800	-2.26119100	1.65390400
B	1.16998400	-1.91724100	1.03829200
B	1.16998400	-1.91724100	-1.03829200
B	-0.39418800	-2.26119100	-1.65390400
B	1.71380000	-3.25235400	0.00000000
B	0.72112300	-3.59988100	-1.46898900
B	-0.94328300	-3.72821200	-0.92173600
B	-0.94328300	-3.72821200	0.92173600
B	0.72112300	-3.59988100	1.46898900
B	0.38916100	-4.50610700	0.00000000
H	-2.39693600	-1.69264100	0.00000000
H	-0.88992900	-1.87668700	2.67049500
H	2.00182500	-1.22167000	1.53787600
H	2.00182500	-1.22167000	-1.53787600
H	-0.88992900	-1.87668700	-2.67049500
H	2.88190500	-3.53214900	0.00000000
H	1.19666500	-4.15269100	-2.42288500
H	-1.81953100	-4.36533600	-1.44027100
H	-1.81953100	-4.36533600	1.44027100
H	1.19666500	-4.15269100	2.42288500
H	0.55840700	-5.69504800	0.00000000

E(RB3LYP) = -696.108917304
Zero-point correction= 0.296571 (Hartree/Particle)
Thermal correction to Energy= 0.316872
Thermal correction to Enthalpy= 0.317816
Thermal correction to Gibbs Free Energy= 0.248569
Sum of electronic and zero-point Energies= -695.812346
Sum of electronic and thermal Energies= -695.792046
Sum of electronic and thermal Enthalpies= -695.791101
Sum of electronic and thermal Free Energies= -695.860348

	E (Thermal)	CV	S
	KCal/Mol	Cal/Mol-Kelvin	Cal/Mol-Kelvin
Total	198.840	96.628	145.741

	1	2	3
	A"	A"	A'
Frequencies --	-130.1970	13.5039	35.5037

Part 1. $[Cu(B_{11}F_{11})_2]^{3-}$: η^5,η^5; PG D_{5d}

Cu	0.00000000	0.00000000	0.00000000
B	0.00000000	1.58581400	1.53635000
B	-1.50840900	0.49009100	1.53645200
B	-0.93236500	-1.28290900	1.53633900
B	0.93236500	-1.28290900	1.53633900
B	1.50840900	0.49009100	1.53645200
B	0.00000000	-1.52363500	3.04475400
B	1.44912500	-0.47077900	3.04495000
B	0.89555000	1.23269100	3.04493400
B	-0.89555000	1.23269100	3.04493400
B	-1.44912500	-0.47077900	3.04495000
B	0.00000000	-0.00005600	3.99035800
B	0.00000000	-1.58581400	-1.53635000
B	1.50840900	-0.49009100	-1.53645200
B	0.93236500	1.28290900	-1.53633900
B	-0.93236500	1.28290900	-1.53633900
B	-1.50840900	-0.49009100	-1.53645200
B	0.00000000	1.52363500	-3.04475400
B	-1.44912500	0.47077900	-3.04495000
B	-0.89555000	-1.23269100	-3.04493400
B	0.89555000	-1.23269100	-3.04493400
B	1.44912500	0.47077900	-3.04495000
B	0.00000000	0.00005600	-3.99035800
F	0.00000000	2.89801200	1.13269000
F	-2.75634400	0.89552500	1.13271700
F	-1.70353700	-2.34454400	1.13271900
F	1.70353700	-2.34454400	1.13271900
F	2.75634400	0.89552500	1.13271700
F	0.00000000	-2.75592200	3.68491600
F	2.62100000	-0.85156900	3.68527000
F	1.61978300	2.22963900	3.68515700
F	-1.61978300	2.22963900	3.68515700
F	-2.62100000	-0.85156900	3.68527000
F	0.00000000	-0.00010100	5.37372500
F	0.00000000	-2.89801200	-1.13269000
F	2.75634400	-0.89552500	-1.13271700
F	1.70353700	2.34454400	-1.13271900
F	-1.70353700	2.34454400	-1.13271900
F	-2.75634400	-0.89552500	-1.13271700
F	0.00000000	2.75592200	-3.68491600
F	-2.62100000	0.85156900	-3.68527000
F	-1.61978300	-2.22963900	-3.68515700
F	1.61978300	-2.22963900	-3.68515700
F	2.62100000	0.85156900	-3.68527000
F	0.00000000	0.00010100	-5.37372500

E(RB3LYP) = -2942.93555960
Zero-point correction= 0.160797 (Hartree/Particle)
Thermal correction to Energy= 0.202385
Thermal correction to Enthalpy= 0.203330
Thermal correction to Gibbs Free Energy= 0.095228
Sum of electronic and zero-point Energies= -2942.774763
Sum of electronic and thermal Energies= -2942.733174
Sum of electronic and thermal Enthalpies= -2942.732230
Sum of electronic and thermal Free Energies= -2942.840331

	E (Thermal)	CV	S
	KCal/Mol	Cal/Mol-Kelvin	Cal/Mol-Kelvin
Total	126.999	160.514	227.519

	1	2	3
	A(A1U)	A(E1U)	A(E1U)
Frequencies --	48.4139	53.8745	54.1120

Part 1. $[Cu(B_{11}F_{11})_2]^{3-}$: η^2,η^2; PG C_2

Cu	-0.00009200	0.00006200	0.24187000
B	1.52362700	3.30426500	-0.70635300
B	0.21824100	2.44488200	-1.39959100
B	-1.38220400	1.66121700	-0.32459000
B	-0.78959500	1.83559400	1.24052100
B	1.06469100	2.68751900	0.81737600
B	-1.83011800	3.13599300	0.58744700
B	-0.33860300	3.56125000	1.48357000
B	0.85625300	4.44895400	0.38152100
B	0.22408600	4.26578800	-1.27809400
B	-1.33482100	3.27531800	-1.12587000
B	-0.91149200	4.56196300	0.08217500
B	-1.52358900	-3.30450300	-0.70651600
B	-1.06476000	-2.68769400	0.81721200
B	0.78937100	-1.83556700	1.24046700
B	1.38202600	-1.66109300	-0.32460700
B	-0.21828300	-2.44491000	-1.39963500
B	1.83009900	-3.13582400	0.58742100
B	1.33487700	-3.27518000	-1.12591700
B	-0.22390400	-4.26583200	-1.27823300
B	-0.85611500	-4.44911400	0.38136500
B	0.33859800	-3.56128700	1.48346400
B	0.91165600	-4.56190000	0.08207500
F	2.82690300	3.26521400	-1.19807400
F	0.46898700	1.64984800	-2.48062200
F	-2.31180000	0.86449900	-0.96048200
F	-1.01625900	1.25488100	2.46978400
F	2.02302700	2.09366400	1.59271300
F	-3.12328700	3.34403800	1.05878000
F	-0.38944500	3.99673000	2.80249600
F	1.51901700	5.54842600	0.91601400
F	0.29158400	5.19262800	-2.31217900
F	-2.27351800	3.45628500	-2.13348200
F	-1.53617800	5.79611700	0.18597400
F	-2.82684800	-3.26556800	-1.19828900
F	-2.02319600	-2.09392600	1.59249700
F	1.01585800	-1.25485200	2.46976200
F	2.31151100	-0.86423300	-0.96048500
F	-0.46912000	-1.64983700	-2.48062100
F	3.12327100	-3.34373200	1.05880400
F	2.27362200	-3.45599700	-2.13350700
F	-0.29123900	-5.19265400	-2.31234600
F	-1.51875600	-5.54867900	0.91582100
F	0.38943000	-3.99677300	2.80238700
F	1.53648900	-5.79598000	0.18587500

E(RB3LYP) = -2942.89781621

Zero-point correction= 0.158077 (Hartree/Particle)
Thermal correction to Energy= 0.202089
Thermal correction to Enthalpy= 0.203034
Thermal correction to Gibbs Free Energy= 0.083693
Sum of electronic and zero-point Energies= -2942.739739
Sum of electronic and thermal Energies= -2942.695727
Sum of electronic and thermal Enthalpies= -2942.694783
Sum of electronic and thermal Free Energies= -2942.814123

	E (Thermal)	CV	S
	KCal/Mol	Cal/Mol-Kelvin	Cal/Mol-Kelvin
Total	126.813	162.219	251.174

	1	2	3
	A(A)	A(B)	A(A)
Frequencies --	13.6134	17.8894	25.8507

Part 1. $[Cu(B_{11}F_{11})_2]^{3-}$: η^2,η^2; PG C_{2h}

Cu	0.00000000	0.00000000	0.00000000
B	-1.64924700	3.54279100	0.00000000
B	-0.72839200	2.73536000	1.18820100
B	1.08673000	1.78665500	0.84037900
B	1.08673000	1.78665500	-0.84037900
B	-0.72839200	2.73536000	-1.18820100
B	1.92562000	3.12832400	0.00000000
B	0.87212700	3.48609900	-1.40185000
B	-0.57028700	4.52991600	-0.89333700
B	-0.57028700	4.52991600	0.89333700
B	0.87212700	3.48609900	1.40185000
B	0.98611400	4.63030300	0.00000000
B	1.64924700	-3.54279200	0.00000000
B	0.72839200	-2.73536000	1.18820100
B	-1.08673000	-1.78665500	0.84037900
B	-1.08673000	-1.78665500	-0.84037900
B	0.72839200	-2.73536000	-1.18820100
B	-1.92562000	-3.12832400	0.00000000
B	-0.87212700	-3.48609900	-1.40185000
B	0.57028700	-4.52991600	-0.89333700
B	0.57028700	-4.52991600	0.89333700
B	-0.87212700	-3.48609900	1.40185000
B	-0.98611400	-4.63030300	0.00000000
F	-3.04338500	3.58793200	0.00000000
F	-1.39479400	2.07984000	2.18462000
F	1.67222100	1.05753200	1.85450000
F	1.67222100	1.05753200	-1.85450000
F	-1.39479300	2.07984000	-2.18462000
F	3.31246200	3.24528300	0.00000000
F	1.40813800	3.75565700	-2.65467900
F	-0.93064100	5.57464800	-1.73715300
F	-0.93064100	5.57464800	1.73715300
F	1.40813800	3.75565700	2.65467900
F	1.69027000	5.82501600	0.00000000
F	3.04338500	-3.58793200	0.00000000
F	1.39479400	-2.07984000	2.18462000
F	-1.67222100	-1.05753200	1.85450000
F	-1.67222100	-1.05753200	-1.85450000
F	1.39479300	-2.07984000	-2.18462000
F	-3.31246200	-3.24528300	0.00000000
F	-1.40813800	-3.75565700	-2.65467900
F	0.93064100	-5.57464800	-1.73715300
F	0.93064100	-5.57464800	1.73715300
F	-1.40813800	-3.75565700	2.65467900
F	-1.69027000	-5.82501600	0.00000000

E(RB3LYP) = -2942.89582825

Zero-point correction= 0.158066 (Hartree/Particle)
Thermal correction to Energy= 0.201194
Thermal correction to Enthalpy= 0.202138
Thermal correction to Gibbs Free Energy= 0.086337
Sum of electronic and zero-point Energies= -2942.737762
Sum of electronic and thermal Energies= -2942.694635
Sum of electronic and thermal Enthalpies= -2942.693690
Sum of electronic and thermal Free Energies= -2942.809491

	E (Thermal)	CV	S
	KCal/Mol	Cal/Mol-Kelvin	Cal/Mol-Kelvin
Total	126.251	160.209	243.723

	1	2	3
	A(Au)	A(Bu)	A(Ag)
Frequencies --	-10.2865	20.3793	30.8975

Part 1. $[Ag(B_{11}F_{11})_2]^{3-}$: η^5,η^5; PG D_{5d}

Ag	0.00000000	0.00000000	0.00000000
B	0.00000000	1.59966200	1.71773200
B	1.52153500	0.49436400	1.71774600
B	0.94062400	-1.29437900	1.71792700
B	-0.94062400	-1.29437900	1.71792700
B	-1.52153500	0.49436400	1.71774600
B	0.00000000	-1.52583400	3.22006200
B	-1.45111700	-0.47145300	3.22015200
B	-0.89673100	1.23428700	3.22011100
B	0.89673100	1.23428700	3.22011100
B	1.45111700	-0.47145300	3.22015200
B	0.00000000	-0.00004800	4.16339000
B	0.00000000	-1.59966200	-1.71773200
B	-1.52153500	-0.49436400	-1.71774600
B	-0.94062400	1.29437900	-1.71792700
B	0.94062400	1.29437900	-1.71792700
B	1.52153500	-0.49436400	-1.71774600
B	0.00000000	1.52583400	-3.22006200
B	1.45111700	0.47145300	-3.22015200
B	0.89673100	-1.23428700	-3.22011100
B	-0.89673100	-1.23428700	-3.22011100
B	-1.45111700	0.47145300	-3.22015200
B	0.00000000	0.00004800	-4.16339000
F	0.00000000	2.90806500	1.29773500
F	2.76573500	0.89901700	1.29757200
F	1.70963600	-2.35270800	1.29721000
F	-1.70963600	-2.35270800	1.29721000
F	-2.76573500	0.89901700	1.29757200
F	0.00000000	-2.75469100	3.86792900
F	-2.61984600	-0.85106700	3.86803100
F	-1.61907100	2.22857400	3.86772200
F	1.61907100	2.22857400	3.86772200
F	2.61984600	-0.85106700	3.86803100
F	0.00000000	-0.00004600	5.54702700
F	0.00000000	-2.90806500	-1.29773500
F	-2.76573500	-0.89901700	-1.29757200
F	-1.70963600	2.35270800	-1.29721000
F	1.70963600	2.35270800	-1.29721000
F	2.76573500	-0.89901700	-1.29757200
F	0.00000000	2.75469100	-3.86792900
F	2.61984600	0.85106700	-3.86803100
F	1.61907100	-2.22857400	-3.86772200
F	-1.61907100	-2.22857400	-3.86772200
F	-2.61984600	0.85106700	-3.86803100
F	0.00000000	0.00004600	-5.54702600

E(RB3LYP) = -2892.55717441

Zero-point correction= 0.159149 (Hartree/Particle)
Thermal correction to Energy= 0.201624
Thermal correction to Enthalpy= 0.202568
Thermal correction to Gibbs Free Energy= 0.091151
Sum of electronic and zero-point Energies= -2892.398025
Sum of electronic and thermal Energies= -2892.355551
Sum of electronic and thermal Enthalpies= -2892.354607
Sum of electronic and thermal Free Energies= -2892.466024

	E (Thermal)	CV	S
	KCal/Mol	Cal/Mol-Kelvin	Cal/Mol-Kelvin
Total	126.521	161.565	234.497

	1	2	3
	A(A1U)	A(E1U)	A(E1U)
Frequencies --	26.9246	45.1234	45.7038

Part 1. $[Ag(B_{11}F_{11})_2]^{3-}$: η^2,η^2; PG C_2

Ag	0.00000000	0.00000000	-0.05298200
B	1.76150900	3.61610400	-0.92993900
B	0.41466400	2.83210400	-1.62069700
B	-1.23469300	2.13880600	-0.61697000
B	-0.65382300	2.23339700	0.94505500
B	1.22322900	2.96040100	0.54728200
B	-1.61881400	3.61390800	0.33013800
B	-0.11317400	3.91799500	1.24746200
B	1.15368200	4.75543600	0.18872100
B	0.52745000	4.65585000	-1.49112900
B	-1.09089200	3.76218000	-1.37195200
B	-0.60021400	4.99104900	-0.13117100
B	-1.76150900	-3.61610400	-0.92993900
B	-1.22322900	-2.96040100	0.54728200
B	0.65382300	-2.23339700	0.94505500
B	1.23469300	-2.13880600	-0.61697000
B	-0.41466400	-2.83210400	-1.62069700
B	1.61881400	-3.61390800	0.33013800
B	1.09089200	-3.76218000	-1.37195200
B	-0.52745000	-4.65585000	-1.49112900
B	-1.15368200	-4.75543600	0.18872100
B	0.11317400	-3.91799500	1.24746200
B	0.60021400	-4.99104900	-0.13117100
F	3.06307300	3.48065800	-1.40586300
F	0.64455100	2.00685900	-2.69023000
F	-2.18118700	1.40055400	-1.30255000
F	-0.89484300	1.61294600	2.15584700
F	2.12844800	2.24059500	1.28586900
F	-2.90242200	3.88504500	0.79344600
F	-0.13916300	4.31086700	2.57977600
F	1.87381700	5.79520700	0.76552200
F	0.65590800	5.60182500	-2.50125300
F	-1.99575900	4.01585900	-2.39445700
F	-1.14276200	6.26107400	-0.00372500
F	-3.06307300	-3.48065800	-1.40586300
F	-2.12844800	-2.24059500	1.28586900
F	0.89484300	-1.61294600	2.15584700
F	2.18118700	-1.40055400	-1.30255000
F	-0.64455100	-2.00685900	-2.69023000
F	2.90242200	-3.88504500	0.79344600
F	1.99575900	-4.01585900	-2.39445700
F	-0.65590800	-5.60182500	-2.50125300
F	-1.87381700	-5.79520700	0.76552200
F	0.13916300	-4.31086700	2.57977600
F	1.14276200	-6.26107400	-0.00372500

E(RB3LYP) = -2892.54804548

Zero-point correction= 0.157421 (Hartree/Particle)
Thermal correction to Energy= 0.201867
Thermal correction to Enthalpy= 0.202812
Thermal correction to Gibbs Free Energy= 0.081002
Sum of electronic and zero-point Energies= -2892.390624
Sum of electronic and thermal Energies= -2892.346178
Sum of electronic and thermal Enthalpies= -2892.345234
Sum of electronic and thermal Free Energies= -2892.467044

	E (Thermal)	CV	S
	KCal/Mol	Cal/Mol-Kelvin	Cal/Mol-Kelvin
Total	126.674	162.532	256.370

	1	2	3
	A(A)	A(B)	A(A)
Frequencies --	7.7051	15.8909	19.5283

Part 1. $[Ag(B_{11}F_{11})_2]^{3-}$: η^2,η^2; PG C_{2h}

Ag	0.00000000	0.00000000	0.00000000
B	-1.88121200	3.70872900	0.00000000
B	-0.88135800	2.95664800	1.15633400
B	0.99063200	2.19021400	0.83412100
B	0.99063200	2.19021400	-0.83412100
B	-0.88135800	2.95664800	-1.15633400
B	1.72476600	3.59943500	0.00000000
B	0.64202300	3.85865200	-1.39957900
B	-0.88978000	4.77052800	-0.89810800
B	-0.88978000	4.77052800	0.89810800
B	0.64202300	3.85865200	1.39957900
B	0.64966100	5.01145200	0.00000000
B	1.88121200	-3.70872900	0.00000000
B	0.88135800	-2.95664800	1.15633400
B	-0.99063200	-2.19021400	0.83412100
B	-0.99063200	-2.19021400	-0.83412100
B	0.88135800	-2.95664800	-1.15633400
B	-1.72476600	-3.59943500	0.00000000
B	-0.64202300	-3.85865200	-1.39957900
B	0.88978000	-4.77052800	-0.89810800
B	0.88978000	-4.77052800	0.89810800
B	-0.64202300	-3.85865200	1.39957900
B	-0.64966100	-5.01145200	0.00000000
F	-3.27063400	3.60916600	0.00000000
F	-1.49426600	2.19937200	2.12105200
F	1.61272100	1.49035500	1.85069300
F	1.61272100	1.49035500	-1.85069300
F	-1.49426600	2.19937200	-2.12105200
F	3.09658600	3.83111900	0.00000000
F	1.14080500	4.16652200	-2.65868900
F	-1.33349000	5.77800000	-1.74681200
F	-1.33349000	5.77800000	1.74681200
F	1.14080500	4.16652200	2.65868900
F	1.23966500	6.26660600	0.00000000
F	3.27063400	-3.60916600	0.00000000
F	1.49426600	-2.19937200	2.12105200
F	-1.61272100	-1.49035500	1.85069300
F	-1.61272100	-1.49035500	-1.85069300
F	1.49426600	-2.19937200	-2.12105200
F	-3.09658600	-3.83111900	0.00000000
F	-1.14080500	-4.16652200	-2.65868900
F	1.33349000	-5.77800000	-1.74681200
F	1.33349000	-5.77800000	1.74681200
F	-1.14080500	-4.16652200	2.65868900
F	-1.23966400	-6.26660700	0.00000000

E(RB3LYP) = -2892.54773841

Zero-point correction= 0.157405 (Hartree/Particle)
Thermal correction to Energy= 0.200936
Thermal correction to Enthalpy= 0.201881
Thermal correction to Gibbs Free Energy= 0.083530
Sum of electronic and zero-point Energies= -2892.390333
Sum of electronic and thermal Energies= -2892.346802
Sum of electronic and thermal Enthalpies= -2892.345858
Sum of electronic and thermal Free Energies= -2892.464208

	E (Thermal)	CV	S
	KCal/Mol	Cal/Mol-Kelvin	Cal/Mol-Kelvin
Total	126.089	160.545	249.089

	1	2	3
	A(Au)	A(Bu)	A(Au)
Frequencies --	-3.8694	17.6083	22.8001

Part 1. $[Au(B_{11}F_{11})_2]^{3-}$: η^5,η^5; PG D_{5d}

Au	0.00000000	0.00000000	0.00000000
B	0.00000000	1.60970200	1.69005800
B	1.53111400	0.49746700	1.69019600
B	0.94649700	-1.30248300	1.69032100
B	-0.94649700	-1.30248300	1.69032100
B	-1.53111400	0.49746700	1.69019600
B	0.00000000	-1.52509600	3.18859700
B	-1.45041500	-0.47123300	3.18867400
B	-0.89628300	1.23368900	3.18860600
B	0.89628300	1.23368900	3.18860600
B	1.45041500	-0.47123300	3.18867400
B	0.00000000	-0.00004500	4.12794300
B	0.00000000	-1.60970200	-1.69005800
B	-1.53111400	-0.49746700	-1.69019600
B	-0.94649700	1.30248300	-1.69032100
B	0.94649700	1.30248300	-1.69032100
B	1.53111400	-0.49746700	-1.69019600
B	0.00000000	1.52509600	-3.18859600
B	1.45041500	0.47123300	-3.18867400
B	0.89628300	-1.23368900	-3.18860600
B	-0.89628300	-1.23368900	-3.18860600
B	-1.45041500	0.47123300	-3.18867400
B	0.00000000	0.00004500	-4.12794300
F	0.00000000	2.91975000	1.27885600
F	2.77689900	0.90266800	1.27891600
F	1.71637100	-2.36219400	1.27832200
F	-1.71637100	-2.36219400	1.27832200
F	-2.77689900	0.90266800	1.27891600
F	0.00000000	-2.75337400	3.83881900
F	-2.61858600	-0.85066000	3.83893500
F	-1.61825200	2.22751000	3.83861800
F	1.61825200	2.22751000	3.83861800
F	2.61858600	-0.85066000	3.83893500
F	0.00000000	-0.00000200	5.51354400
F	0.00000000	-2.91975000	-1.27885600
F	-2.77689900	-0.90266800	-1.27891600
F	-1.71637100	2.36219400	-1.27832200
F	1.71637100	2.36219400	-1.27832200
F	2.77689900	-0.90266800	-1.27891600
F	0.00000000	2.75337400	-3.83881900
F	2.61858600	0.85066000	-3.83893500
F	1.61825200	-2.22751000	-3.83861800
F	-1.61825200	-2.22751000	-3.83861800
F	-2.61858600	0.85066000	-3.83893500
F	0.00000000	0.00000200	-5.51354400

E(RB3LYP) = -2881.37710911

Zero-point correction=	0.160251 (Hartree/Particle)
Thermal correction to Energy=	0.202345
Thermal correction to Enthalpy=	0.203289
Thermal correction to Gibbs Free Energy=	0.092936
Sum of electronic and zero-point Energies=	-2881.216858
Sum of electronic and thermal Energies=	-2881.174764
Sum of electronic and thermal Enthalpies=	-2881.173820
Sum of electronic and thermal Free Energies=	-2881.284173

	E (Thermal)	CV	S
	KCal/Mol	Cal/Mol-Kelvin	Cal/Mol-Kelvin
Total	126.973	160.814	232.257

	1	2	3
	A(A1U)	A(E1U)	A(E1U)
Frequencies --	30.4546	48.7097	49.1820

Part 1. $[Au(B_{11}F_{11})_2]^{3-}$: η^2,η^2; PG C_2

Au	0.00000000	0.00000000	0.06599200
B	1.68339000	3.16908500	-1.04327700
B	0.26383400	2.45867800	-1.69721400
B	-1.37065600	1.82524400	-0.46656000
B	-0.65986000	1.99320200	1.09597200
B	1.29860400	2.69392700	0.55359800
B	-1.58016800	3.36083300	0.42059600
B	-0.00464800	3.67849800	1.23176500
B	1.19158400	4.42579900	0.02867200
B	0.45583700	4.25472500	-1.58020100
B	-1.17641200	3.41643600	-1.32857200
B	-0.57381700	4.68290100	-0.17187700
B	-1.68339000	-3.16908500	-1.04327700
B	-1.29860400	-2.69392700	0.55359800
B	0.65986000	-1.99320200	1.09597200
B	1.37065600	-1.82524400	-0.46656000
B	-0.26383400	-2.45867800	-1.69721400
B	1.58016800	-3.36083300	0.42059600
B	1.17641200	-3.41643600	-1.32857200
B	-0.45583700	-4.25472500	-1.58020100
B	-1.19158400	-4.42579900	0.02867200
B	0.00464800	-3.67849800	1.23176500
B	0.57381700	-4.68290100	-0.17187700
F	2.94835800	3.00929000	-1.60912800
F	0.37214100	1.66312500	-2.79696000
F	-2.47455700	1.19461700	-0.99228400
F	-0.94139200	1.54566300	2.36563500
F	2.25014900	2.09509900	1.32654100
F	-2.81807100	3.70673900	0.95183200
F	0.03484800	4.17261400	2.53080900
F	1.97787400	5.47963500	0.47691900
F	0.56159000	5.14946600	-2.63756900
F	-2.16242500	3.68199600	-2.26984900
F	-1.08437000	5.96641300	-0.06915100
F	-2.94835800	-3.00929000	-1.60912800
F	-2.25014900	-2.09509900	1.32654100
F	0.94139200	-1.54566300	2.36563500
F	2.47455700	-1.19461700	-0.99228400
F	-0.37214100	-1.66312500	-2.79696000
F	2.81807100	-3.70673900	0.95183200
F	2.16242500	-3.68199600	-2.26984900
F	-0.56159000	-5.14946600	-2.63756900
F	-1.97787400	-5.47963500	0.47691900
F	-0.03484800	-4.17261400	2.53080900
F	1.08437000	-5.96641300	-0.06915100

E(RB3LYP) = -2881.32066006

Zero-point correction= 0.157727 (Hartree/Particle)
Thermal correction to Energy= 0.201735
Thermal correction to Enthalpy= 0.202679
Thermal correction to Gibbs Free Energy= 0.083786
Sum of electronic and zero-point Energies= -2881.162933
Sum of electronic and thermal Energies= -2881.118925
Sum of electronic and thermal Enthalpies= -2881.117981
Sum of electronic and thermal Free Energies= -2881.236875

	E (Thermal)	CV	S
	KCal/Mol	Cal/Mol-Kelvin	Cal/Mol-Kelvin
Total	126.591	162.414	250.233

	1	2	3
	A(A)	A(B)	A(A)
Frequencies --	11.0132	20.3028	23.7476

Part 1. $[Au(B_{11}F_{11})_2]^{3-}$: η^2,η^2; PG C_{2h}

Au	0.00000000	0.00000000	0.00000000
B	-1.72126500	3.41806600	0.00000000
B	-0.76290400	2.72491200	1.23594600
B	1.16679900	1.87100100	0.85933100
B	1.16679900	1.87100100	-0.85933200
B	-0.76290400	2.72491200	-1.23594600
B	1.86044700	3.27081800	0.00000000
B	0.78453300	3.55974200	-1.41222600
B	-0.72172700	4.50272700	-0.88998900
B	-0.72172700	4.50272700	0.88998900
B	0.78453300	3.55974200	1.41222600
B	0.82588200	4.70197500	0.00000000
B	1.72126500	-3.41806600	0.00000000
B	0.76290400	-2.72491200	1.23594600
B	-1.16679900	-1.87100100	0.85933100
B	-1.16679900	-1.87100100	-0.85933200
B	0.76290400	-2.72491200	-1.23594600
B	-1.86044700	-3.27081800	0.00000000
B	-0.78453300	-3.55974200	-1.41222600
B	0.72172700	-4.50272700	-0.88998900
B	0.72172700	-4.50272700	0.88998900
B	-0.78453300	-3.55974200	1.41222600
B	-0.82588200	-4.70197500	0.00000000
F	-3.11661800	3.37873100	0.00000000
F	-1.37519300	2.06926900	2.26174300
F	1.88482800	1.25886800	1.85817600
F	1.88482800	1.25886800	-1.85817600
F	-1.37519300	2.06926900	-2.26174300
F	3.23285900	3.49431700	0.00000000
F	1.32006000	3.88740900	-2.65168900
F	-1.15791300	5.52812200	-1.71974100
F	-1.15791300	5.52812200	1.71974100
F	1.32006000	3.88740900	2.65168900
F	1.45192300	5.93721400	0.00000000
F	3.11661800	-3.37873100	0.00000000
F	1.37519300	-2.06926900	2.26174300
F	-1.88482800	-1.25886800	1.85817600
F	-1.88482800	-1.25886800	-1.85817600
F	1.37519300	-2.06926900	-2.26174300
F	-3.23285900	-3.49431700	0.00000000
F	-1.32006000	-3.88740900	-2.65168900
F	1.15791300	-5.52812200	-1.71974100
F	1.15791300	-5.52812200	1.71974100
F	-1.32006000	-3.88740800	2.65168900
F	-1.45192300	-5.93721400	0.00000000

E(RB3LYP) = -2881.31853040

Zero-point correction= 0.157724 (Hartree/Particle)
Thermal correction to Energy= 0.200865
Thermal correction to Enthalpy= 0.201809
Thermal correction to Gibbs Free Energy= 0.085846
Sum of electronic and zero-point Energies= -2881.160806
Sum of electronic and thermal Energies= -2881.117665
Sum of electronic and thermal Enthalpies= -2881.116721
Sum of electronic and thermal Free Energies= -2881.232684

	E (Thermal)	CV	S
	KCal/Mol	Cal/Mol-Kelvin	Cal/Mol-Kelvin
Total	126.045	160.430	244.065

	1	2	3
	A(Au)	A(Bu)	A(Au)
Frequencies --	-9.5088	21.9083	26.3665

Part 2. $[Cu(B_{11}H_{11})_2]^{3-}$: η^5,η^5; PG D_{5d}

Cu	0.00000000	0.00000000	0.00000000
B	0.50268700	2.12567400	0.00000000
B	1.05773700	1.19890000	1.48694700
B	1.95645400	-0.30108800	0.91942700
B	1.95645400	-0.30108800	-0.91942700
B	1.05773700	1.19890000	-1.48694700
B	3.36490100	0.25063300	0.00000000
B	2.82681500	1.14919900	-1.44129600
B	1.95626100	2.60276000	-0.89075900
B	1.95626100	2.60276000	0.89075900
B	2.82681500	1.14919900	1.44129600
B	3.38849000	2.03132700	0.00000000
B	-0.50268700	-2.12567400	0.00000000
B	-1.05773700	-1.19890000	-1.48694700
B	-1.95645400	0.30108800	-0.91942700
B	-1.95645400	0.30108800	0.91942700
B	-1.05773700	-1.19890000	1.48694700
B	-3.36490100	-0.25063300	0.00000000
B	-2.82681500	-1.14919900	1.44129600
B	-1.95626100	-2.60276000	0.89075900
B	-1.95626100	-2.60276000	-0.89075900
B	-2.82681500	-1.14919900	-1.44129600
B	-3.38849000	-2.03132700	0.00000000
H	-0.45060000	2.83677800	0.00000000
H	0.49375900	1.25732300	2.53201700
H	2.02466000	-1.29610100	1.56684300
H	2.02466000	-1.29610100	-1.56684300
H	0.49375900	1.25732300	-2.53201700
H	4.37112600	-0.39660000	0.00000000
H	3.45317200	1.13807500	-2.46060000
H	1.96585800	3.61926200	-1.52164800
H	1.96585800	3.61926200	1.52164800
H	3.45317200	1.13807500	2.46060000
H	4.41410800	2.64623100	0.00000000
H	0.45060000	-2.83677800	0.00000000
H	-0.49375900	-1.25732300	-2.53201700
H	-2.02466000	1.29610100	-1.56684300
H	-2.02466000	1.29610100	1.56684300
H	-0.49375900	-1.25732300	2.53201700
H	-4.37112600	0.39660000	0.00000000
H	-3.45317200	-1.13807500	2.46060000
H	-1.96585800	-3.61926200	1.52164800
H	-1.96585800	-3.61926200	-1.52164800
H	-3.45317200	-1.13807500	-2.46060000
H	-4.41410800	-2.64623100	0.00000000

E(RB3LYP) = -758.184536388
Zero-point correction= 0.304941 (Hartree/Particle)
Thermal correction to Energy= 0.323097
Thermal correction to Enthalpy= 0.324042
Thermal correction to Gibbs Free Energy= 0.264337
Sum of electronic and zero-point Energies= -757.879596
Sum of electronic and thermal Energies= -757.861439
Sum of electronic and thermal Enthalpies= -757.860495
Sum of electronic and thermal Free Energies= -757.920199

	E (Thermal)	CV	S
	KCal/Mol	Cal/Mol-Kelvin	Cal/Mol-Kelvin
Total	202.747	94.091	125.658

	1	2	3
	A(A1U)	A(E1U)	A(E1U)
Frequencies --	47.6837	87.4268	98.4695

Part 2. $[Cu(B_{11}H_{11})_2]^{3-}$: η^2,η^2; PG C_2

Cu	0.00000000	0.00000000	0.45132200
B	0.40230800	4.07549000	-1.51267000
B	-0.60553600	2.73551400	-1.46498300
B	-1.27871400	1.67154400	-0.08079000
B	-0.10831900	2.09260400	1.03363400
B	0.81580400	3.24224000	-0.11595300
B	-1.70597500	2.86157900	1.17156600
B	-0.18568800	3.78739800	1.31476600
B	0.00985500	4.92851800	-0.07875200
B	-1.24652900	4.47885600	-1.26888300
B	-2.14689300	3.08324900	-0.54533100
B	-1.62249700	4.47915000	0.47106200
B	-0.40230800	-4.07549000	-1.51267000
B	-0.81580400	-3.24224000	-0.11595300
B	0.10831900	-2.09260400	1.03363400
B	1.27871400	-1.67154400	-0.08079000
B	0.60553600	-2.73551400	-1.46498300
B	1.70597500	-2.86157900	1.17156600
B	2.14689300	-3.08324900	-0.54533100
B	1.24652900	-4.47885600	-1.26888300
B	-0.00985500	-4.92851800	-0.07875200
B	0.18568800	-3.78739800	1.31476600
B	1.62249700	-4.47915000	0.47106200
H	1.14078200	4.36904900	-2.40130800
H	-0.52486700	2.02114400	-2.42172000
H	-1.82229300	0.62297200	-0.34867400
H	0.50380700	1.45912800	1.86491700
H	1.96374000	2.90831000	-0.06378800
H	-2.46616200	2.62280400	2.06230300
H	0.27991700	4.18981900	2.33952200
H	0.45749900	6.01174200	0.16337000
H	-1.84961700	5.18624400	-2.02261700
H	-3.24220300	2.92413500	-0.99658200
H	-2.35356400	5.30630000	0.92976800
H	-1.14078200	-4.36904900	-2.40130800
H	-1.96374000	-2.90831000	-0.06378800
H	-0.50380700	-1.45912800	1.86491700
H	1.82229300	-0.62297200	-0.34867400
H	0.52486700	-2.02114400	-2.42172000
H	2.46616200	-2.62280400	2.06230300
H	3.24220300	-2.92413500	-0.99658200
H	1.84961700	-5.18624400	-2.02261700
H	-0.45749900	-6.01174200	0.16337000
H	-0.27991700	-4.18981900	2.33952200
H	2.35356400	-5.30630000	0.92976800

E(RB3LYP) = -758.168381597

Zero-point correction= 0.299052 (Hartree/Particle)
Thermal correction to Energy= 0.320033
Thermal correction to Enthalpy= 0.320978
Thermal correction to Gibbs Free Energy= 0.250259
Sum of electronic and zero-point Energies= -757.869329
Sum of electronic and thermal Energies= -757.848348
Sum of electronic and thermal Enthalpies= -757.847404
Sum of electronic and thermal Free Energies= -757.918123

	E (Thermal)	CV	S
	KCal/Mol	Cal/Mol-Kelvin	Cal/Mol-Kelvin
Total	200.824	97.010	148.841

	1	2	3
	A(A)	A(B)	A(A)
Frequencies --	19.6987	21.9757	29.4209

Part 2. $[Cu(B_{11}H_{11})_2]^{3-}$: η^2,η^2; PG C_{2h}

Cu	0.00000000	0.00000000	0.00000000
B	-1.77813300	3.61347000	0.00000000
B	-0.84340800	2.67522300	1.03433400
B	0.84340800	1.82387600	0.84351600
B	0.84340800	1.82387600	-0.84351600
B	-0.84340800	2.67522300	-1.03433400
B	1.79236000	3.05838800	0.00000000
B	0.74906900	3.46221100	-1.39412000
B	-0.62675300	4.52241700	-0.89266200
B	-0.62675300	4.52241700	0.89266200
B	0.74906900	3.46221100	1.39412000
B	0.90789100	4.59167700	0.00000000
B	1.77813300	-3.61347000	0.00000000
B	0.84340800	-2.67522300	1.03433400
B	-0.84340800	-1.82387600	0.84351600
B	-0.84340800	-1.82387600	-0.84351600
B	0.84340800	-2.67522300	-1.03433400
B	-1.79236000	-3.05838800	0.00000000
B	-0.74906900	-3.46221100	-1.39412000
B	0.62675300	-4.52241700	-0.89266200
B	0.62675300	-4.52241700	0.89266200
B	-0.74906900	-3.46221100	1.39412000
B	-0.90789100	-4.59167700	0.00000000
H	-2.96870600	3.67937400	0.00000000
H	-1.48373600	2.01582700	1.79906900
H	1.22605700	1.04575000	1.67376100
H	1.22605700	1.04575000	-1.67376100
H	-1.48373600	2.01582700	-1.79906900
H	2.98752600	3.06244700	0.00000000
H	1.15021200	3.66434000	-2.50175500
H	-0.94332400	5.40338400	-1.63794900
H	-0.94332400	5.40338400	1.63794900
H	1.15021200	3.66434000	2.50175500
H	1.56711200	5.58873700	0.00000000
H	2.96870600	-3.67937400	0.00000000
H	1.48373600	-2.01582700	1.79906900
H	-1.22605700	-1.04575000	1.67376100
H	-1.22605700	-1.04575000	-1.67376100
H	1.48373600	-2.01582700	-1.79906900
H	-2.98752600	-3.06244700	0.00000000
H	-1.15021200	-3.66434000	-2.50175500
H	0.94332400	-5.40338400	-1.63794900
H	0.94332400	-5.40338400	1.63794900
H	-1.15021200	-3.66434000	2.50175500
H	-1.56711200	-5.58873700	0.00000000

E(RB3LYP) = -758.166906217
Zero-point correction= 0.299557 (Hartree/Particle)
Thermal correction to Energy= 0.319480
Thermal correction to Enthalpy= 0.320424
Thermal correction to Gibbs Free Energy= 0.253315
Sum of electronic and zero-point Energies= -757.867349
Sum of electronic and thermal Energies= -757.847426
Sum of electronic and thermal Enthalpies= -757.846482
Sum of electronic and thermal Free Energies= -757.913591

	E (Thermal)	CV	S
	KCal/Mol	Cal/Mol-Kelvin	Cal/Mol-Kelvin
Total	200.477	95.223	141.243

	1	2	3
	BU	AU	AG
Frequencies --	-21.5292	14.8403	41.3223

Part 2. $[Cu(B_{11}H_{11})_2]^{3-}$: η^2,η^2; PG C_{2v}

Cu	0.00000000	0.00000000	0.84365200
B	0.00000000	2.93042100	-2.08137400
B	-1.03063100	2.31748600	-0.90491700
B	-0.84203000	2.01529600	0.95571100
B	0.84203000	2.01529600	0.95571100
B	1.03063100	2.31748600	-0.90491700
B	0.00000000	3.48115000	1.49015200
B	1.39375600	3.54997400	0.37409300
B	0.89316200	4.14416100	-1.25764000
B	-0.89316200	4.14416100	-1.25764000
B	-1.39375600	3.54997400	0.37409300
B	0.00000000	4.67479200	0.18424300
B	0.00000000	-2.93042100	-2.08137400
B	-1.03063100	-2.31748600	-0.90491700
B	-0.84203000	-2.01529600	0.95571100
B	0.84203000	-2.01529600	0.95571100
B	1.03063100	-2.31748600	-0.90491700
B	0.00000000	-3.48115000	1.49015200
B	1.39375600	-3.54997400	0.37409300
B	0.89316200	-4.14416100	-1.25764000
B	-0.89316200	-4.14416100	-1.25764000
B	-1.39375600	-3.54997400	0.37409300
B	0.00000000	-4.67479200	0.18424300
H	0.00000000	2.63270900	-3.23594100
H	-1.79421800	1.49314200	-1.31267400
H	-1.67578500	1.39544000	1.55698500
H	1.67578500	1.39544000	1.55698500
H	1.79421800	1.49314200	-1.31267400
H	0.00000000	3.84471500	2.62902500
H	2.50221500	3.86237200	0.69474700
H	1.63879900	4.88787900	-1.82597900
H	-1.63879900	4.88787900	-1.82597900
H	-2.50221500	3.86237200	0.69474700
H	0.00000000	5.82480500	0.51022600
H	0.00000000	-2.63270900	-3.23594100
H	-1.79421800	-1.49314200	-1.31267400
H	-1.67578500	-1.39544000	1.55698500
H	1.67578500	-1.39544000	1.55698500
H	1.79421800	-1.49314200	-1.31267400
H	0.00000000	-3.84471500	2.62902500
H	2.50221500	-3.86237200	0.69474700
H	1.63879900	-4.88787900	-1.82597900
H	-1.63879900	-4.88787900	-1.82597900
H	-2.50221500	-3.86237200	0.69474700
H	0.00000000	-5.82480500	0.51022600

E(RB3LYP) = -758.165517525

Zero-point correction= 0.299547 (Hartree/Particle)
Thermal correction to Energy= 0.318536
Thermal correction to Enthalpy= 0.319480
Thermal correction to Gibbs Free Energy= 0.255996
Sum of electronic and zero-point Energies= -757.865971
Sum of electronic and thermal Energies= -757.846982
Sum of electronic and thermal Enthalpies= -757.846038
Sum of electronic and thermal Free Energies= -757.909522

	E (Thermal)	CV	S
	KCal/Mol	Cal/Mol-Kelvin	Cal/Mol-Kelvin
Total	199.884	93.180	133.614

	1	2	3
	B2	A2	A2
Frequencies --	-52.6909	-48.9627	49.7828

Part 2. $[Cu(B_{11}H_{11})_2]^{3-}$: η^5,η^2; PG C_s

Cu	0.00000000	0.36438700	-0.12210400
B	0.00000000	-1.81028000	4.08896100
B	1.00714800	-1.01217900	3.01098700
B	0.82504200	0.52167500	1.97539600
B	-0.82504200	0.52167500	1.97539600
B	-1.00714800	-1.01217900	3.01098700
B	0.00000000	1.66435000	3.05984900
B	-1.39912300	0.68708900	3.58526200
B	-0.89429600	-0.54343800	4.81805000
B	0.89429600	-0.54343800	4.81805000
B	1.39912300	0.68708900	3.58526200
B	0.00000000	0.99098100	4.68785900
H	0.00000000	-2.98014100	4.31834900
H	1.76463100	-1.72467200	2.41911100
H	1.61351800	0.75607600	1.09167600
H	-1.61351800	0.75607600	1.09167600
H	-1.76463100	-1.72467200	2.41911100
H	0.00000000	2.84562700	2.88003000
H	-2.51003700	1.10028500	3.73919000
H	-1.64209500	-0.73449300	5.73272800
H	1.64209500	-0.73449300	5.73272800
H	2.51003700	1.10028500	3.73919000
H	0.00000000	1.76396400	5.59984000
B	0.00000000	-1.59732200	-1.71382100
B	1.29479100	-0.41725900	-1.64858000
B	0.84556200	1.49837000	-1.86772200
B	-0.84556200	1.49837000	-1.86772200
B	-1.29479100	-0.41725900	-1.64858000
B	0.00000000	1.52493200	-3.42730200
B	-1.41509800	0.48078100	-3.18174900
B	-0.87637600	-1.26384200	-3.17523000
B	0.87637600	-1.26384200	-3.17523000
B	1.41509800	0.48078100	-3.18174900
B	0.00000000	-0.07102200	-4.15829400
H	0.00000000	-2.65546500	-1.15926100
H	2.23117900	-0.59612000	-0.92999000
H	1.61277300	2.31449800	-1.44955300
H	-1.61277300	2.31449800	-1.44955300
H	-2.23117900	-0.59612000	-0.92999000
H	0.00000000	2.49583700	-4.12488900
H	-2.49230300	0.71409500	-3.64809700
H	-1.59263300	-2.07655400	-3.68143700
H	1.59263300	-2.07655400	-3.68143700
H	2.49230300	0.71409500	-3.64809700
H	0.00000000	-0.15464000	-5.35056300

E(RB3LYP) = -758.164451413

Zero-point correction= 0.299778 (Hartree/Particle)
Thermal correction to Energy= 0.319616
Thermal correction to Enthalpy= 0.320560
Thermal correction to Gibbs Free Energy= 0.253455
Sum of electronic and zero-point Energies= -757.864673
Sum of electronic and thermal Energies= -757.844836
Sum of electronic and thermal Enthalpies= -757.843891
Sum of electronic and thermal Free Energies= -757.910996

	E (Thermal)	CV	S
	KCal/Mol	Cal/Mol-Kelvin	Cal/Mol-Kelvin
Total	200.562	94.995	141.233

	1	2	3
	A(A")	A(A')	A(A")
Frequencies --	-83.5102	29.7507	29.8255

Part 2. $[Cu(B_{11}H_{11})_2]^{3-}$: η^5,η^2; PG C_s

Cu	0.00000000	-0.28193800	-0.13347900
B	0.00000000	1.82980500	4.19318400
B	1.00371500	1.08214800	3.07690900
B	0.82345200	-0.40196500	1.97770200
B	-0.82345200	-0.40196500	1.97770200
B	-1.00371500	1.08214800	3.07690900
B	0.00000000	-1.59680500	3.00972500
B	-1.39926900	-0.64483100	3.57520900
B	-0.89430000	0.53086500	4.86229100
B	0.89430000	0.53086500	4.86229100
B	1.39926900	-0.64483100	3.57520900
B	0.00000000	-0.99642100	4.66500700
H	0.00000000	2.98779800	4.47660700
H	1.76217200	1.81893600	2.51699400
H	1.61010800	-0.57981100	1.07952200
H	-1.61010800	-0.57981100	1.07952200
H	-1.76217200	1.81893600	2.51699300
H	0.00000000	-2.76811900	2.77432000
H	-2.51064900	-1.06279800	3.71274700
H	-1.64303600	0.67932300	5.78421200
H	1.64303600	0.67932300	5.78421200
H	2.51064900	-1.06279800	3.71274700
H	0.00000000	-1.80790200	5.54296000
B	0.00000000	-1.87799400	-2.05659800
B	1.30795700	-0.73739700	-1.77807400
B	0.84525100	1.18094200	-1.62002800
B	-0.84525100	1.18094200	-1.62002800
B	-1.30795700	-0.73739700	-1.77807400
B	0.00000000	1.51072500	-3.14567600
B	-1.41588600	0.44093400	-3.10804600
B	-0.87501100	-1.27399900	-3.43226400
B	0.87501100	-1.27399900	-3.43226400
B	1.41588600	0.44093400	-3.10804600
B	0.00000000	0.08510800	-4.17026900
H	0.00000000	-3.02649600	-1.72642800
H	2.25881500	-1.04990100	-1.12968900
H	1.60989800	1.89934500	-1.04645000
H	-1.60989800	1.89934500	-1.04645000
H	-2.25881500	-1.04990100	-1.12968900
H	0.00000000	2.59817900	-3.64221400
H	-2.49195300	0.75972700	-3.52386600
H	-1.58893800	-1.97843600	-4.08313900
H	1.58893800	-1.97843600	-4.08313900
H	2.49195300	0.75972700	-3.52386600
H	0.00000000	0.23192800	-5.35646100

E(RB3LYP) = -758.163029939

Zero-point correction= 0.299356 (Hartree/Particle)
Thermal correction to Energy= 0.318461
Thermal correction to Enthalpy= 0.319405
Thermal correction to Gibbs Free Energy= 0.254108
Sum of electronic and zero-point Energies= -757.863674
Sum of electronic and thermal Energies= -757.844569
Sum of electronic and thermal Enthalpies= -757.843624
Sum of electronic and thermal Free Energies= -757.908922

	E (Thermal)	CV	S
	KCal/Mol	Cal/Mol-Kelvin	Cal/Mol-Kelvin
	1	2	3
	A(A")	A(A")	A(A')
Frequencies --	-89.7978	-20.4077	27.3481

Part 2. $[Ag(B_{11}H_{11})_2]^{3-}$: η^5,η^5; PG D_{5d}

Ag	0.00000000	0.00000000	0.00000000
B	-0.57672100	2.24343100	0.00000000
B	0.38136300	1.72444300	1.50030200
B	1.93126900	0.88164100	0.92689900
B	1.93126900	0.88164100	-0.92689900
B	0.38136300	1.72444300	-1.50030200
B	2.85282000	2.07210300	0.00000000
B	1.93126900	2.57244400	-1.44321400
B	0.43992000	3.38289700	-0.89189700
B	0.43992000	3.38289700	0.89189700
B	1.93126900	2.57244400	1.44321400
B	1.96373300	3.61507300	0.00000000
B	0.57672100	-2.24343100	0.00000000
B	-0.38136300	-1.72444300	-1.50030200
B	-1.93126900	-0.88164100	-0.92689900
B	-1.93126900	-0.88164100	0.92689900
B	-0.38136300	-1.72444300	1.50030200
B	-2.85282000	-2.07210300	0.00000000
B	-1.93126900	-2.57244400	1.44321400
B	-0.43992000	-3.38289700	0.89189700
B	-0.43992000	-3.38289700	-0.89189700
B	-1.93126900	-2.57244400	-1.44321400
B	-1.96373300	-3.61507300	0.00000000
H	-1.76145900	2.36451700	0.00000000
H	-0.13471800	1.48622400	2.54689700
H	2.49359400	0.05443000	1.57335200
H	2.49359400	0.05443000	-1.57335200
H	-0.13471800	1.48622400	-2.54689700
H	4.04805300	2.03581700	0.00000000
H	2.47816200	2.88926100	-2.45856600
H	-0.06142500	4.26900900	-1.51935000
H	-0.06142500	4.26900900	1.51935000
H	2.47816200	2.88926100	2.45856600
H	2.53433000	4.66530900	0.00000000
H	1.76145900	-2.36451700	0.00000000
H	0.13471800	-1.48622400	-2.54689700
H	-2.49359400	-0.05443000	-1.57335200
H	-2.49359400	-0.05443000	1.57335200
H	0.13471800	-1.48622400	2.54689700
H	-4.04805300	-2.03581700	0.00000000
H	-2.47816200	-2.88926100	2.45856600
H	0.06142500	-4.26900900	1.51935000
H	0.06142500	-4.26900900	-1.51935000
H	-2.47816200	-2.88926100	-2.45856600
H	-2.53433000	-4.66530900	0.00000000

E(RB3LYP) = -707.810091113
Zero-point correction= 0.304020 (Hartree/Particle)
Thermal correction to Energy= 0.322610
Thermal correction to Enthalpy= 0.323554
Thermal correction to Gibbs Free Energy= 0.262502
Sum of electronic and zero-point Energies= -707.506072
Sum of electronic and thermal Energies= -707.487481
Sum of electronic and thermal Enthalpies= -707.486537
Sum of electronic and thermal Free Energies= -707.547589

	E (Thermal)	CV	S
	KCal/Mol	Cal/Mol-Kelvin	Cal/Mol-Kelvin
Total	202.441	94.756	128.495

	1	2	3
	AU(A1U)	BU(E1U)	AU(E1U)
Frequencies --	48.9466	77.3640	82.9839

Part 2. $[Ag(B_{11}H_{11})_2]^{3-}$: η^2,η^2; PG C_2

Ag	0.00000000	0.00000000	0.45392700
B	1.45163100	3.60658700	-1.19079200
B	0.11983600	2.59996100	-1.37877500
B	-1.21861800	1.97413100	-0.19383600
B	-0.33881600	2.26940000	1.21544000
B	1.19085600	2.96040600	0.33771100
B	-1.59390800	3.42633500	0.74634400
B	0.02133500	3.96133900	1.29604000
B	0.92996600	4.77106700	-0.04080800
B	0.00000000	4.45832200	-1.53269600
B	-1.43209200	3.47277100	-1.03410100
B	-0.84388000	4.83896700	-0.01806300
B	-1.45163100	-3.60658700	-1.19079200
B	-1.19085600	-2.96040600	0.33771100
B	0.33881600	-2.26940000	1.21544000
B	1.21861800	-1.97413100	-0.19383600
B	-0.11983600	-2.59996100	-1.37877500
B	1.59390800	-3.42633500	0.74634400
B	1.43209200	-3.47277100	-1.03410100
B	0.00000000	-4.45832200	-1.53269600
B	-0.92996600	-4.77106700	-0.04080800
B	-0.02133500	-3.96133900	1.29604000
B	0.84388000	-4.83896700	-0.01806300
H	2.46736400	3.54709800	-1.81271100
H	0.27339300	1.74978900	-2.20667400
H	-1.98819400	1.11861900	-0.53779600
H	-0.23408700	1.70707400	2.27087400
H	2.13586000	2.37473900	0.78113600
H	-2.61421200	3.55467500	1.35637000
H	0.25730400	4.39672000	2.38419900
H	1.58571600	5.73199900	0.24022000
H	-0.11972700	5.15817400	-2.49620200
H	-2.35124900	3.52043800	-1.79710100
H	-1.41217700	5.88175500	0.11807500
H	-2.46736400	-3.54709800	-1.81271100
H	-2.13586000	-2.37473900	0.78113600
H	0.23408700	-1.70707400	2.27087400
H	1.98819400	-1.11861900	-0.53779600
H	-0.27339300	-1.74978900	-2.20667400
H	2.61421200	-3.55467500	1.35637000
H	2.35124900	-3.52043800	-1.79710100
H	0.11972700	-5.15817400	-2.49620200
H	-1.58571600	-5.73199900	0.24022000
H	-0.25730400	-4.39672000	2.38419900
H	1.41217700	-5.88175500	0.11807500

E(RB3LYP) = -707.809902956

Zero-point correction= 0.299137 (Hartree/Particle)
Thermal correction to Energy= 0.320166
Thermal correction to Enthalpy= 0.321110
Thermal correction to Gibbs Free Energy= 0.249411
Sum of electronic and zero-point Energies= -707.510766
Sum of electronic and thermal Energies= -707.489737
Sum of electronic and thermal Enthalpies= -707.488793
Sum of electronic and thermal Free Energies= -707.560492

	E (Thermal)	CV	S
	KCal/Mol	Cal/Mol-Kelvin	Cal/Mol-Kelvin
Total	200.907	97.255	150.903

	1	2	3
	A	A	B
Frequencies --	16.3554	22.4586	23.4899

Part 2. $[Ag(B_{11}H_{11})_2]^{3-}$: η[2],η[2]; PG C_{2h}

Ag	0.00000000	0.00000000	0.00000000
B	-1.82037200	3.75809600	0.00000000
B	-0.84230100	2.86023300	1.02971400
B	0.87932800	2.08683000	0.84432200
B	0.87932800	2.08683000	-0.84432200
B	-0.84230100	2.86023300	-1.02971400
B	1.77063000	3.36104000	0.00000000
B	0.71087400	3.72053000	-1.39449400
B	-0.71087400	4.71869300	-0.89260900
B	-0.71087400	4.71869300	0.89260900
B	0.71087400	3.72053000	1.39449400
B	0.81683300	4.85558500	0.00000000
B	1.82037200	-3.75809600	0.00000000
B	0.84230100	-2.86023300	1.02971400
B	-0.87932800	-2.08683000	0.84432200
B	-0.87932800	-2.08683000	-0.84432200
B	0.84230100	-2.86023300	-1.02971400
B	-1.77063000	-3.36104000	0.00000000
B	-0.71087400	-3.72053000	-1.39449400
B	0.71087400	-4.71869300	-0.89260900
B	0.71087400	-4.71869300	0.89260900
B	-0.71087400	-3.72053000	1.39449400
B	-0.81683300	-4.85558500	0.00000000
H	-3.01267400	3.76800200	0.00000000
H	-1.45413300	2.16863200	1.79085000
H	1.30934400	1.34139500	1.68066700
H	1.30934400	1.34139500	-1.68066700
H	-1.45413300	2.16863200	-1.79085000
H	2.96470100	3.42188000	0.00000000
H	1.10108700	3.94326100	-2.50232800
H	-1.06757100	5.58482900	-1.63744900
H	-1.06757100	5.58482900	1.63744900
H	1.10108700	3.94326100	2.50232800
H	1.43398400	5.87940000	0.00000000
H	3.01267400	-3.76800200	0.00000000
H	1.45413300	-2.16863200	1.79085000
H	-1.30934400	-1.34139500	1.68066700
H	-1.30934400	-1.34139500	-1.68066700
H	1.45413300	-2.16863200	-1.79085000
H	-2.96470100	-3.42188000	0.00000000
H	-1.10108700	-3.94326100	-2.50232800
H	1.06757100	-5.58482900	-1.63744900
H	1.06757100	-5.58482900	1.63744900
H	-1.10108700	-3.94326100	2.50232800
H	-1.43398400	-5.87940000	0.00000000

E(RB3LYP) = -707.809541023
Zero-point correction= 0.298981 (Hartree/Particle)
Thermal correction to Energy= 0.319169
Thermal correction to Enthalpy= 0.320113
Thermal correction to Gibbs Free Energy= 0.251523
Sum of electronic and zero-point Energies= -707.510560
Sum of electronic and thermal Energies= -707.490372
Sum of electronic and thermal Enthalpies= -707.489428
Sum of electronic and thermal Free Energies= -707.558018

	E (Thermal)	CV	S
	KCal/Mol	Cal/Mol-Kelvin	Cal/Mol-Kelvin
Total	200.282	95.399	144.360

	1	2	3
	AU	BU	AU
Frequencies --	-20.6509	23.1541	27.3938

Part 2. $[Ag(B_{11}H_{11})_2]^{3-}$: η^2,η^2; PG C_{2v}

Ag	0.77740700	-0.00000200	0.00000000
B	-2.16556900	3.10179500	0.00000000
B	-0.97285500	2.51667100	1.02758900
B	0.89486900	2.26303600	0.84323900
B	0.89486900	2.26303600	-0.84323900
B	-0.97285500	2.51667100	-1.02758900
B	1.39109800	3.73887000	0.00000000
B	0.27302000	3.78302000	-1.39425600
B	-1.37324200	4.33611200	-0.89276400
B	-1.37324200	4.33611200	0.89276400
B	0.27302000	3.78302000	1.39425600
B	0.05331900	4.90130500	0.00000000
H	-3.31266200	2.77591500	0.00000000
H	-1.35896100	1.67793000	1.78835400
H	1.51511000	1.67153500	1.68412200
H	1.51511000	1.67153500	-1.68412200
H	-1.35896100	1.67793000	-1.78835400
H	2.52007700	4.13298600	0.00000000
H	0.58429300	4.10697500	-2.50232500
H	-1.96137800	5.06512000	-1.63783800
H	-1.96137800	5.06512000	1.63783800
H	0.58429300	4.10697500	2.50232500
H	0.35327100	6.05856600	0.00000000
B	-2.16556800	-3.10178100	0.00000000
B	-0.97285000	-2.51666600	1.02758900
B	0.89487600	-2.26304400	0.84323900
B	0.89487600	-2.26304400	-0.84323900
B	-0.97285000	-2.51666600	-1.02758900
B	1.39109600	-3.73888200	0.00000000
B	0.27301600	-3.78302200	-1.39425600
B	-1.37325000	-4.33610200	-0.89276500
B	-1.37325000	-4.33610200	0.89276500
B	0.27301600	-3.78302200	1.39425600
B	0.05330700	-4.90130700	0.00000000
H	-3.31265800	-2.77589000	0.00000000
H	-1.35894900	-1.67792100	1.78835400
H	1.51512100	-1.67154500	1.68412200
H	1.51512100	-1.67154500	-1.68412200
H	-1.35894900	-1.67792100	-1.78835400
H	2.52007000	-4.13300600	0.00000000
H	0.58428600	-4.10697800	-2.50232600
H	-1.96139200	-5.06510700	-1.63783800
H	-1.96139200	-5.06510700	1.63783800
H	0.58428600	-4.10697800	2.50232600
H	0.35325000	-6.05857000	0.00000000

E(RB3LYP) = -707.809352700

Zero-point correction= 0.299252 (Hartree/Particle)
Thermal correction to Energy= 0.318506
Thermal correction to Enthalpy= 0.319450
Thermal correction to Gibbs Free Energy= 0.252788
Sum of electronic and zero-point Energies= -707.510101
Sum of electronic and thermal Energies= -707.490846
Sum of electronic and thermal Enthalpies= -707.489902
Sum of electronic and thermal Free Energies= -707.556565

	E (Thermal)	CV	S
	KCal/Mol	Cal/Mol-Kelvin	Cal/Mol-Kelvin
Total	199.866	93.186	140.303

	1	2	3
	A'(A1)	A"(B1)	A"(A2)
Frequencies --	-49.8865	-47.0231	16.0248

Part 2. $[Au(B_{11}H_{11})_2]^{3-}$: η[5],η[5]; PG D_{5d}

Au	0.00000000	0.00000000	0.00000000
B	-0.58595600	2.23022400	0.00000000
B	0.37497500	1.70456600	1.50787600
B	1.92955700	0.85144100	0.93166500
B	1.92955700	0.85144100	-0.93166500
B	0.37497500	1.70456600	-1.50787600
B	2.84818900	2.04154200	0.00000000
B	1.92955700	2.54495300	-1.44165000
B	0.44306300	3.36039600	-0.89096600
B	0.44306300	3.36039600	0.89096600
B	1.92955700	2.54495300	1.44165000
B	1.96648600	3.58693600	0.00000000
B	0.58595600	-2.23022400	0.00000000
B	-0.37497500	-1.70456600	-1.50787600
B	-1.92955700	-0.85144100	-0.93166500
B	-1.92955700	-0.85144100	0.93166500
B	-0.37497500	-1.70456600	1.50787600
B	-2.84818900	-2.04154200	0.00000000
B	-1.92955700	-2.54495300	1.44165000
B	-0.44306300	-3.36039600	0.89096600
B	-0.44306300	-3.36039600	-0.89096600
B	-1.92955700	-2.54495300	-1.44165000
B	-1.96648600	-3.58693600	0.00000000
H	-1.76770600	2.37397200	0.00000000
H	-0.13556800	1.48425600	2.56057400
H	2.50138500	0.03465400	1.58216400
H	2.50138500	0.03465400	-1.58216400
H	-0.13556800	1.48425600	-2.56057400
H	4.04329800	2.00478500	0.00000000
H	2.47888900	2.86312300	-2.45513500
H	-0.05157200	4.25094000	-1.51725900
H	-0.05157200	4.25094000	1.51725900
H	2.47888900	2.86312300	2.45513500
H	2.54126500	4.63506800	0.00000000
H	1.76770600	-2.37397200	0.00000000
H	0.13556800	-1.48425600	-2.56057400
H	-2.50138500	-0.03465400	-1.58216400
H	-2.50138500	-0.03465400	1.58216400
H	0.13556800	-1.48425600	2.56057400
H	-4.04329800	-2.00478500	0.00000000
H	-2.47888900	-2.86312300	2.45513500
H	0.05157200	-4.25094000	1.51725900
H	0.05157200	-4.25094000	-1.51725900
H	-2.47888900	-2.86312300	-2.45513500
H	-2.54126500	-4.63506800	0.00000000

E(RB3LYP) = -696.633759472

Zero-point correction= 0.305650 (Hartree/Particle)
Thermal correction to Energy= 0.323893
Thermal correction to Enthalpy= 0.324837
Thermal correction to Gibbs Free Energy= 0.264065
Sum of electronic and zero-point Energies= -696.328110
Sum of electronic and thermal Energies= -696.309867
Sum of electronic and thermal Enthalpies= -696.308923
Sum of electronic and thermal Free Energies= -696.369695

	E (Thermal)	CV	S
	KCal/Mol	Cal/Mol-Kelvin	Cal/Mol-Kelvin
Total	203.246	93.629	127.906

	1	2	3
	AU(A1U)	AU(E1U)	BU(E1U)
Frequencies --	45.5284	77.5146	82.5539

Part 2. $[Au(B_{11}H_{11})_2]^{3-}$: η^2,η^2; PG C_2

Au	0.00000000	0.00000000	0.45508000
B	1.39375800	3.16931800	-1.48685200
B	-0.05625700	2.31133900	-1.48069000
B	-1.30016100	1.87587600	-0.05454600
B	-0.20754100	2.18420800	1.24348100
B	1.30070800	2.69092700	0.12538900
B	-1.38156700	3.42889200	0.79296900
B	0.33063400	3.84098600	1.10279200
B	1.13248000	4.46537000	-0.38578700
B	0.00000000	4.14905000	-1.72767300
B	-1.43638400	3.34661600	-0.99125500
B	-0.61023400	4.70790800	-0.15875600
B	-1.39375800	-3.16931800	-1.48685200
B	-1.30070800	-2.69092700	0.12538900
B	0.20754100	-2.18420800	1.24348100
B	1.30016100	-1.87587600	-0.05454600
B	0.05625700	-2.31133900	-1.48069000
B	1.38156700	-3.42889200	0.79296900
B	1.43638400	-3.34661600	-0.99125500
B	0.00000000	-4.14905000	-1.72767300
B	-1.13248000	-4.46537000	-0.38578700
B	-0.33063400	-3.84098600	1.10279200
B	0.61023400	-4.70790800	-0.15875600
H	2.31475800	2.96877300	-2.21745800
H	-0.09275600	1.42240100	-2.27643600
H	-2.25002900	1.17028900	-0.23238600
H	-0.06410900	1.78588700	2.36306700
H	2.25320200	2.07660800	0.50338500
H	-2.30110200	3.71016000	1.50285800
H	0.71961800	4.31848900	2.12645900
H	1.90365500	5.37143900	-0.26316900
H	-0.17083600	4.79155500	-2.72200900
H	-2.44552400	3.43185900	-1.62475200
H	-1.06719700	5.80424400	-0.03057600
H	-2.31475800	-2.96877300	-2.21745800
H	-2.25320200	-2.07660800	0.50338500
H	0.06410900	-1.78588700	2.36306700
H	2.25002900	-1.17028900	-0.23238600
H	0.09275600	-1.42240100	-2.27643600
H	2.30110200	-3.71016000	1.50285800
H	2.44552400	-3.43185900	-1.62475200
H	0.17083600	-4.79155500	-2.72200900
H	-1.90365500	-5.37143900	-0.26316900
H	-0.71961800	-4.31848900	2.12645900
H	1.06719700	-5.80424400	-0.03057600

E(RB3LYP) = -696.575689785
Zero-point correction= 0.300157 (Hartree/Particle)
Thermal correction to Energy= 0.320713
Thermal correction to Enthalpy= 0.321657
Thermal correction to Gibbs Free Energy= 0.252609
Sum of electronic and zero-point Energies= -696.275533
Sum of electronic and thermal Energies= -696.254976
Sum of electronic and thermal Enthalpies= -696.254032
Sum of electronic and thermal Free Energies= -696.323080

	E (Thermal)	CV	S
	KCal/Mol	Cal/Mol-Kelvin	Cal/Mol-Kelvin
Total	201.251	97.096	145.324

	1	2	3
	A	B	A
Frequencies --	27.6837	29.7554	30.1217

Part 2. $[Au(B_{11}H_{11})_2]^{3-}$: η^2,η^2; PG C_{2h}

Au	0.00000000	0.00000000	0.00000000
B	-1.79973600	3.56771600	0.00000000
B	-0.80682500	2.72106000	1.06415600
B	0.96532000	1.95828700	0.86035000
B	0.96532000	1.95828700	-0.86035000
B	-0.80682500	2.72106000	-1.06415600
B	1.79035000	3.26489500	0.00000000
B	0.71967600	3.60456000	-1.39192400
B	-0.71967600	4.56725500	-0.89201100
B	-0.71967600	4.56725500	0.89201100
B	0.71967600	3.60456000	1.39192400
B	0.80436700	4.73740500	0.00000000
B	1.79973600	-3.56771600	0.00000000
B	0.80682500	-2.72106000	1.06415600
B	-0.96532000	-1.95828700	0.86035000
B	-0.96532000	-1.95828700	-0.86035000
B	0.80682500	-2.72106000	-1.06415600
B	-1.79035000	-3.26489500	0.00000000
B	-0.71967600	-3.60456000	-1.39192400
B	0.71967600	-4.56725500	-0.89201100
B	0.71967600	-4.56725500	0.89201100
B	-0.71967600	-3.60456000	1.39192400
B	-0.80436700	-4.73740500	0.00000000
H	-2.99187800	3.54895500	0.00000000
H	-1.40560400	2.03861600	1.84066100
H	1.47597600	1.30409100	1.72222800
H	1.47597600	1.30409100	-1.72222800
H	-1.40560400	2.03861600	-1.84066100
H	2.98110700	3.36862600	0.00000000
H	1.12128600	3.82882400	-2.49443400
H	-1.10015900	5.42434400	-1.63438600
H	-1.10015900	5.42434400	1.63438600
H	1.12128600	3.82882400	2.49443400
H	1.40609800	5.76947900	0.00000000
H	2.99187800	-3.54895500	0.00000000
H	1.40560400	-2.03861600	1.84066100
H	-1.47597600	-1.30409100	1.72222800
H	-1.47597600	-1.30409100	-1.72222800
H	1.40560400	-2.03861600	-1.84066100
H	-2.98110700	-3.36862600	0.00000000
H	-1.12128600	-3.82882400	-2.49443400
H	1.10015900	-5.42434400	-1.63438600
H	1.10015900	-5.42434400	1.63438600
H	-1.12128600	-3.82882400	2.49443400
H	-1.40609800	-5.76947900	0.00000000

E(RB3LYP) = -696.574845702

Zero-point correction= 0.299916 (Hartree/Particle)
Thermal correction to Energy= 0.319655
Thermal correction to Enthalpy= 0.320599
Thermal correction to Gibbs Free Energy= 0.254234
Sum of electronic and zero-point Energies= -696.274930
Sum of electronic and thermal Energies= -696.255191
Sum of electronic and thermal Enthalpies= -696.254247
Sum of electronic and thermal Free Energies= -696.320612

	E (Thermal)	CV	S
	KCal/Mol	Cal/Mol-Kelvin	Cal/Mol-Kelvin
Total	200.586	95.232	139.677

	1	2	3
	AU	BU	AU
Frequencies --	-22.6296	27.7970	40.4153

Part 2. $[Au(B_{11}H_{11})_2]^{3-}$: η^2,η^2; PG C_{2v}

Au	0.68683800	0.00000000	0.00000000
B	-2.27619800	2.84020900	0.00000000
B	-1.06475900	2.34554400	1.05821700
B	0.84874600	2.17758800	0.85961300
B	0.84874600	2.17758800	-0.85961300
B	-1.06475900	2.34554400	-1.05821700
B	1.22964600	3.67793900	0.00000000
B	0.10547600	3.66589400	-1.39169900
B	-1.56311300	4.12630500	-0.89242200
B	-1.56311300	4.12630500	0.89242200
B	0.10547600	3.66589400	1.39169900
B	-0.16881300	4.76716500	0.00000000
H	-3.40213600	2.44839000	0.00000000
H	-1.41582500	1.50527300	1.83072600
H	1.53378800	1.71882700	1.72786300
H	1.53378800	1.71882700	-1.72786300
H	-1.41582500	1.50527300	-1.83072600
H	2.32883200	4.14760700	0.00000000
H	0.41527200	4.00287000	-2.49517900
H	-2.19377200	4.82016800	-1.63516700
H	-2.19377200	4.82016800	1.63516700
H	0.41527200	4.00287000	2.49517900
H	0.07761500	5.93616900	0.00000000
B	-2.27619800	-2.84020900	0.00000000
B	-1.06475900	-2.34554400	1.05821700
B	0.84874600	-2.17758800	0.85961400
B	0.84874600	-2.17758800	-0.85961400
B	-1.06475900	-2.34554400	-1.05821700
B	1.22964600	-3.67793900	0.00000000
B	0.10547600	-3.66589400	-1.39169900
B	-1.56311400	-4.12630500	-0.89242200
B	-1.56311400	-4.12630500	0.89242200
B	0.10547600	-3.66589400	1.39169900
B	-0.16881300	-4.76716500	0.00000000
H	-3.40213600	-2.44839000	0.00000000
H	-1.41582500	-1.50527300	1.83072500
H	1.53378800	-1.71882700	1.72786300
H	1.53378800	-1.71882700	-1.72786300
H	-1.41582500	-1.50527300	-1.83072500
H	2.32883200	-4.14760700	0.00000000
H	0.41527200	-4.00287000	-2.49517900
H	-2.19377200	-4.82016700	-1.63516700
H	-2.19377200	-4.82016700	1.63516700
H	0.41527200	-4.00287000	2.49517900
H	0.07761400	-5.93616900	0.00000000

E(RB3LYP) = -696.574280959
Zero-point correction= 0.299713 (Hartree/Particle)
Thermal correction to Energy= 0.319545
Thermal correction to Enthalpy= 0.320489
Thermal correction to Gibbs Free Energy= 0.251829
Sum of electronic and zero-point Energies= -696.274568
Sum of electronic and thermal Energies= -696.254736
Sum of electronic and thermal Enthalpies= -696.253792
Sum of electronic and thermal Free Energies= -696.322452

	E (Thermal)	CV	S
	KCal/Mol	Cal/Mol-Kelvin	Cal/Mol-Kelvin
Total	200.517	95.335	144.507

	1	2	3
	A"(A2)	A'(B2)	A"(B1)
Frequencies --	-51.6157	5.4432	41.7389

Part 2. $[Au(B_{11}H_{11})_2]^{3-}$: η[5],η[2]; PG C_s

Au	0.00000000	0.45116100	-0.14011900
B	0.00000000	-1.83193800	4.16905300
B	1.00703500	-0.99006100	3.12434600
B	0.83142700	0.58522000	2.14401200
B	-0.83142700	0.58522000	2.14401200
B	-1.00703500	-0.99006100	3.12434600
B	0.00000000	1.68124700	3.27305000
B	-1.39780800	0.68304800	3.76674700
B	-0.89430400	-0.59501200	4.94721000
B	0.89430400	-0.59501200	4.94721000
B	1.39780800	0.68304800	3.76674700
B	0.00000000	0.94495900	4.87542900
H	0.00000000	-3.00941600	4.35219900
H	1.76405500	-1.67910800	2.50542300
H	1.66996000	0.87408200	1.31724800
H	-1.66996000	0.87408200	1.31724800
H	-1.76405500	-1.67910800	2.50542300
H	0.00000000	2.86852600	3.14274100
H	-2.50810500	1.09091500	3.93241300
H	-1.64371100	-0.82187900	5.85166800
H	1.64371100	-0.82187900	5.85166800
H	2.50810500	1.09091400	3.93241300
H	0.00000000	1.68571300	5.81314700
B	0.00000000	-1.58675800	-1.80051600
B	1.35040700	-0.40791300	-1.76643100
B	0.86082600	1.55193700	-2.07438100
B	-0.86082600	1.55193700	-2.07438100
B	-1.35040700	-0.40791300	-1.76643100
B	0.00000000	1.48736700	-3.61618800
B	-1.42211500	0.46028400	-3.32840900
B	-0.87369300	-1.28029600	-3.26356100
B	0.87369300	-1.28029600	-3.26356100
B	1.42211500	0.46028400	-3.32840900
B	0.00000000	-0.12486500	-4.28519900
H	0.00000000	-2.64499800	-1.24737300
H	2.35012200	-0.59471400	-1.14075800
H	1.62453900	2.39814800	-1.72111400
H	-1.62453900	2.39814800	-1.72111400
H	-2.35012200	-0.59471400	-1.14075800
H	0.00000000	2.43367600	-4.34644800
H	-2.48726200	0.67635000	-3.82919100
H	-1.58880500	-2.10949100	-3.74374400
H	1.58880500	-2.10949100	-3.74374400
H	2.48726200	0.67635000	-3.82919100
H	0.00000000	-0.24786000	-5.47399300

E(RB3LYP) = -696.565629184

Zero-point correction= 0.299071 (Hartree/Particle)
Thermal correction to Energy= 0.319119
Thermal correction to Enthalpy= 0.320063
Thermal correction to Gibbs Free Energy= 0.251270
Sum of electronic and zero-point Energies= -696.266558
Sum of electronic and thermal Energies= -696.246511
Sum of electronic and thermal Enthalpies= -696.245566
Sum of electronic and thermal Free Energies= -696.314359

	E (Thermal)	CV	S
	KCal/Mol	Cal/Mol-Kelvin	Cal/Mol-Kelvin
Total	200.250	95.424	144.787

	1	2	3
	A(A")	A(A')	A(A")
Frequencies --	-105.8563	19.3375	20.8128

Part 2. $[Au(B_{11}H_{11})_2]^{3-}$: η^5,η^2; PG C_s

Au	0.00000000	-0.44920700	-0.15443400
B	0.00000000	1.84381400	4.22111100
B	1.00328100	1.02821700	3.15278100
B	0.82911600	-0.52106900	2.14036300
B	-0.82911700	-0.52106900	2.14036400
B	-1.00328200	1.02821700	3.15278100
B	0.00000000	-1.64878400	3.24247600
B	-1.39858400	-0.66358300	3.75603500
B	-0.89440900	0.58792300	4.96679100
B	0.89440900	0.58792300	4.96679100
B	1.39858400	-0.66358300	3.75603500
B	0.00000000	-0.95009600	4.85997400
H	0.00000000	3.01638100	4.43347800
H	1.76096700	1.73003400	2.54917300
H	1.66226000	-0.77489500	1.29467100
H	-1.66226000	-0.77489500	1.29467100
H	-1.76096800	1.73003400	2.54917300
H	0.00000000	-2.83191700	3.07963300
H	-2.50933200	-1.07334100	3.91426300
H	-1.64461700	0.79205200	5.87608200
H	1.64461700	0.79205200	5.87608200
H	2.50933200	-1.07334000	3.91426200
H	0.00000000	-1.71034000	5.78205400
B	0.00000000	-1.87686900	-2.19809600
B	1.37616300	-0.72695100	-1.90711900
B	0.85258000	1.23526800	-1.74406500
B	-0.85258000	1.23526800	-1.74406500
B	-1.37616300	-0.72695100	-1.90711900
B	0.00000000	1.54240400	-3.26752900
B	-1.42198800	0.48214900	-3.22208500
B	-0.87439200	-1.23248600	-3.54841800
B	0.87439200	-1.23248700	-3.54841800
B	1.42198800	0.48214900	-3.22208500
B	0.00000000	0.12429400	-4.28398000
H	0.00000000	-3.04111000	-1.93755500
H	2.39457100	-1.04587900	-1.37263800
H	1.60712300	1.96630000	-1.17664700
H	-1.60712300	1.96630000	-1.17664700
H	-2.39457100	-1.04587900	-1.37263800
H	0.00000000	2.63026600	-3.76334000
H	-2.48737200	0.80178300	-3.66406200
H	-1.58135200	-1.93597700	-4.20729200
H	1.58135200	-1.93597700	-4.20729200
H	2.48737200	0.80178300	-3.66406200
H	0.00000000	0.26610900	-5.47071900

E(RB3LYP) = -696.564686286
Zero-point correction= 0.298813 (Hartree/Particle)
Thermal correction to Energy= 0.318047
Thermal correction to Enthalpy= 0.318991
Thermal correction to Gibbs Free Energy= 0.252965
Sum of electronic and zero-point Energies= -696.265873
Sum of electronic and thermal Energies= -696.246639
Sum of electronic and thermal Enthalpies= -696.245695
Sum of electronic and thermal Free Energies= -696.311721

	E (Thermal)	CV	S
	KCal/Mol	Cal/Mol-Kelvin	Cal/Mol-Kelvin
Total	199.577	93.522	138.964

	1	2	3
	A(A")	A(A")	A(A')
Frequencies --	-107.1259	-29.7904	19.7685

Part 2. $[Cu(B_{11}F_{11})_2]^{3-}$: η^5,η^5; PG D_{5d}

Cu	0.00000000	0.00000000	0.00000000
B	-1.51991200	1.58061100	0.00000000
B	-1.51888500	0.48944900	-1.50222200
B	-1.51835400	-1.27727300	-0.92911200
B	-1.51835400	-1.27727300	0.92911200
B	-1.51888500	0.48944900	1.50222200
B	-3.02310200	-1.52136000	0.00000000
B	-3.02378300	-0.46967400	1.44714300
B	-3.02468600	1.23109400	0.89429500
B	-3.02468600	1.23109400	-0.89429500
B	-3.02378300	-0.46967400	-1.44714300
B	-3.97345700	-0.00039400	0.00000000
B	1.51991200	-1.58061100	0.00000000
B	1.51888500	-0.48944900	1.50222200
B	1.51835400	1.27727300	0.92911200
B	1.51835400	1.27727300	-0.92911200
B	1.51888500	-0.48944900	-1.50222200
B	3.02310200	1.52136000	0.00000000
B	3.02378300	0.46967400	-1.44714300
B	3.02468600	-1.23109400	-0.89429500
B	3.02468600	-1.23109400	0.89429500
B	3.02378300	0.46967400	1.44714300
B	3.97345700	0.00039400	0.00000000
F	-1.11811200	2.89655800	0.00000000
F	-1.11413700	0.89788900	-2.75219200
F	-1.11445800	-2.34068500	-1.70293900
F	-1.11445800	-2.34068500	1.70293900
F	-1.11413700	0.89788900	2.75219200
F	-3.64370100	-2.75746100	0.00000000
F	-3.64484700	-0.85157800	2.62249600
F	-3.64555000	2.23076200	1.62104900
F	-3.64555000	2.23076200	-1.62104900
F	-3.64484600	-0.85157800	-2.62249600
F	-5.34655000	-0.00118800	0.00000000
F	1.11811200	-2.89655800	0.00000000
F	1.11413700	-0.89788900	2.75219200
F	1.11445800	2.34068500	1.70293900
F	1.11445800	2.34068500	-1.70293900
F	1.11413700	-0.89788900	-2.75219200
F	3.64370100	2.75746100	0.00000000
F	3.64484600	0.85157800	-2.62249600
F	3.64555000	-2.23076200	-1.62104900
F	3.64555000	-2.23076200	1.62104900
F	3.64484700	0.85157800	2.62249600
F	5.34655000	0.00118800	0.00000000

E(RB3LYP) = -2943.37420983
Zero-point correction= 0.160872 (Hartree/Particle)
Thermal correction to Energy= 0.202367
Thermal correction to Enthalpy= 0.203312
Thermal correction to Gibbs Free Energy= 0.096114
Sum of electronic and zero-point Energies= -2943.213338
Sum of electronic and thermal Energies= -2943.171842
Sum of electronic and thermal Enthalpies= -2943.170898
Sum of electronic and thermal Free Energies= -2943.278096

	E (Thermal)	CV	S
	KCal/Mol	Cal/Mol-Kelvin	Cal/Mol-Kelvin
Total	126.987	160.230	225.616

	1	2	3
	A(A1U)	A(E1U)	A(E1U)
Frequencies --	53.0250	54.6444	57.2847

Part 2. $[Cu(B_{11}F_{11})_2]^{3-}$: η^2,η^2; PG C_2

Cu	0.00000000	0.00000000	0.19188900
B	1.64833500	2.87479200	-0.73876000
B	0.23938700	2.18537900	-1.42151000
B	-1.40277200	1.60976400	-0.28720800
B	-0.77043500	1.74771800	1.26852500
B	1.16570600	2.36890900	0.82026400
B	-1.66667300	3.13583900	0.60032200
B	-0.12830300	3.40529700	1.47049600
B	1.14442200	4.12420600	0.32365000
B	0.46766700	3.98388500	-1.31185600
B	-1.19755200	3.18641400	-1.12042500
B	-0.60172900	4.43739900	0.05486300
B	-1.64833500	-2.87479200	-0.73876000
B	-1.16570600	-2.36890900	0.82026400
B	0.77043500	-1.74771800	1.26852500
B	1.40277200	-1.60976400	-0.28720800
B	-0.23938700	-2.18537900	-1.42151000
B	1.66667300	-3.13583900	0.60032200
B	1.19755200	-3.18641400	-1.12042500
B	-0.46766700	-3.98388500	-1.31185600
B	-1.14442200	-4.12420600	0.32365000
B	0.12830300	-3.40529700	1.47049600
B	0.60172900	-4.43739900	0.05486300
F	2.92451300	2.65898200	-1.25127400
F	0.37624200	1.38274300	-2.51891800
F	-2.43322000	0.89576800	-0.87282700
F	-1.05703200	1.20532400	2.50427900
F	2.07357600	1.72204000	1.61275300
F	-2.91984700	3.48416100	1.08536900
F	-0.10763200	3.86990600	2.77643900
F	1.93620100	5.13787400	0.82979200
F	0.62171700	4.86289200	-2.36813100
F	-2.12887200	3.45424400	-2.11120800
F	-1.07674800	5.73015500	0.14351300
F	-2.92451300	-2.65898200	-1.25127400
F	-2.07357600	-1.72204000	1.61275300
F	1.05703200	-1.20532400	2.50427900
F	2.43322000	-0.89576800	-0.87282700
F	-0.37624200	-1.38274300	-2.51891800
F	2.91984700	-3.48416100	1.08536900
F	2.12887200	-3.45424400	-2.11120800
F	-0.62171700	-4.86289200	-2.36813100
F	-1.93620100	-5.13787400	0.82979200
F	0.10763200	-3.86990600	2.77643900
F	1.07674800	-5.73015500	0.14351300

E(RB3LYP) = -2943.31835822
Zero-point correction= 0.157918 (Hartree/Particle)
Thermal correction to Energy= 0.201874
Thermal correction to Enthalpy= 0.202818
Thermal correction to Gibbs Free Energy= 0.085334
Sum of electronic and zero-point Energies= -2943.160440
Sum of electronic and thermal Energies= -2943.116484
Sum of electronic and thermal Enthalpies= -2943.115540
Sum of electronic and thermal Free Energies= -2943.233025

	E (Thermal)	CV	S
	KCal/Mol	Cal/Mol-Kelvin	Cal/Mol-Kelvin
Total	126.678	162.219	247.267

	1	2	3
	A(B)	A(A)	A(A)
Frequencies --	21.6131	25.0724	27.0810

Part 2. $[Ag(B_{11}F_{11})_2]^{3-}$: η^5,η^5; PG D_{5d}

Ag	0.00000000	0.00000000	0.00000000
B	-1.69920000	1.59400000	0.00000000
B	-1.69867600	0.49242500	1.51573400
B	-1.69904900	-1.28917300	0.93719800
B	-1.69904900	-1.28917300	-0.93719800
B	-1.69867600	0.49242500	-1.51573400
B	-3.19784300	-1.52439900	0.00000000
B	-3.19818900	-0.47088800	-1.44961300
B	-3.19807300	1.23302300	-0.89599700
B	-3.19807300	1.23302300	0.89599700
B	-3.19818900	-0.47088800	1.44961300
B	-4.14396500	-0.00014100	0.00000000
B	1.69920000	-1.59400000	0.00000000
B	1.69867600	-0.49242500	-1.51573400
B	1.69904900	1.28917300	-0.93719800
B	1.69904900	1.28917300	0.93719800
B	1.69867600	-0.49242500	1.51573400
B	3.19784300	1.52439900	0.00000000
B	3.19818900	0.47088800	1.44961300
B	3.19807300	-1.23302300	0.89599700
B	3.19807300	-1.23302300	-0.89599700
B	3.19818900	0.47088800	-1.44961300
B	4.14396500	0.00014100	0.00000000
F	-1.27669800	2.90469600	0.00000000
F	-1.27283000	0.89666300	2.76134200
F	-1.27381300	-2.34806700	1.70793400
F	-1.27381300	-2.34806700	-1.70793400
F	-1.27283000	0.89666300	-2.76134200
F	-3.82717200	-2.75659300	0.00000000
F	-3.82778900	-0.85149600	-2.62137200
F	-3.82709600	2.22984000	-1.62055200
F	-3.82709600	2.22984000	1.62055200
F	-3.82778900	-0.85149600	2.62137200
F	-5.51829700	-0.00030600	0.00000000
F	1.27669800	-2.90469600	0.00000000
F	1.27283000	-0.89666300	-2.76134200
F	1.27381300	2.34806700	-1.70793400
F	1.27381300	2.34806700	1.70793400
F	1.27283000	-0.89666300	2.76134200
F	3.82717200	2.75659300	0.00000000
F	3.82778900	0.85149600	2.62137200
F	3.82709600	-2.22984000	1.62055200
F	3.82709600	-2.22984000	-1.62055200
F	3.82778900	0.85149600	-2.62137200
F	5.51829700	0.00030600	0.00000000

E(RB3LYP) = -2892.98974482

Zero-point correction= 0.159074 (Hartree/Particle)

Thermal correction to Energy= 0.201523

Thermal correction to Enthalpy= 0.202467

Thermal correction to Gibbs Free Energy= 0.091089

Sum of electronic and zero-point Energies= -2892.830671

Sum of electronic and thermal Energies= -2892.788222

Sum of electronic and thermal Enthalpies= -2892.787278

Sum of electronic and thermal Free Energies= -2892.898655

	E (Thermal)	CV	S
	KCal/Mol	Cal/Mol-Kelvin	Cal/Mol-Kelvin
Total	126.458	161.380	234.414

	1	2	3
	A(A1U)	A(E1U)	A(E1U)
Frequencies --	26.4174	46.2312	47.4524

Part 2. $[Ag(B_{11}F_{11})_2]^{3-}$: η^2,η^2; PG C_2

Ag	0.00000000	0.00000000	0.17642100
B	1.69798800	3.28375400	-0.68167400
B	0.32384800	2.57444900	-1.39780500
B	-1.35705200	1.97113200	-0.36644800
B	-0.78834300	2.07996000	1.20309600
B	1.14049300	2.72358800	0.82714400
B	-1.67427100	3.48073300	0.53868100
B	-0.17095300	3.73864800	1.46758500
B	1.14099800	4.49429300	0.39221200
B	0.52738800	4.38066900	-1.28026500
B	-1.13334500	3.56201000	-1.15990100
B	-0.59622000	4.79701300	0.05649800
B	-1.69798800	-3.28375400	-0.68167400
B	-1.14049300	-2.72358800	0.82714400
B	0.78834300	-2.07996000	1.20309600
B	1.35705200	-1.97113200	-0.36644800
B	-0.32384800	-2.57444900	-1.39780500
B	1.67427100	-3.48073300	0.53868100
B	1.13334500	-3.56201000	-1.15990100
B	-0.52738800	-4.38066900	-1.28026500
B	-1.14099800	-4.49429300	0.39221200
B	0.17095300	-3.73864800	1.46758500
B	0.59622000	-4.79701300	0.05649800
F	2.99276500	3.06031800	-1.14291500
F	0.51887400	1.74867700	-2.47584000
F	-2.34895900	1.26565300	-1.02621200
F	-1.09603200	1.50281300	2.42286800
F	2.01720500	2.02412900	1.61683500
F	-2.94853000	3.81203400	0.98123200
F	-0.19828400	4.16889200	2.78679500
F	1.89569900	5.51015800	0.95156700
F	0.70219900	5.28842700	-2.31008600
F	-2.02361700	3.83274700	-2.18921700
F	-1.08262400	6.08710100	0.14859500
F	-2.99276500	-3.06031800	-1.14291500
F	-2.01720500	-2.02412900	1.61683500
F	1.09603200	-1.50281300	2.42286800
F	2.34895900	-1.26565300	-1.02621200
F	-0.51887400	-1.74867700	-2.47584000
F	2.94853000	-3.81203400	0.98123200
F	2.02361700	-3.83274700	-2.18921700
F	-0.70219900	-5.28842700	-2.31008600
F	-1.89569900	-5.51015800	0.95156700
F	0.19828400	-4.16889200	2.78679500
F	1.08262400	-6.08710100	0.14859500

E(RB3LYP) = -2892.96235732

Zero-point correction= 0.157264 (Hartree/Particle)
Thermal correction to Energy= 0.201566
Thermal correction to Enthalpy= 0.202510
Thermal correction to Gibbs Free Energy= 0.081899
Sum of electronic and zero-point Energies= -2892.805093
Sum of electronic and thermal Energies= -2892.760792
Sum of electronic and thermal Enthalpies= -2892.759848
Sum of electronic and thermal Free Energies= -2892.880458

	E (Thermal)	CV	S
	KCal/Mol	Cal/Mol-Kelvin	Cal/Mol-Kelvin
Total	126.484	162.505	253.847

	1	2	3
	A(A)	A(B)	A(A)
Frequencies --	7.9174	15.1402	20.2865

Part 2. $[Au(B_{11}F_{11})_2]^{3-}$: η^5,η^5; PG D_{5d}

Au	0.00000000	0.00000000	0.00000000
B	-1.67682400	1.60504200	0.00000000
B	-1.67633300	0.49589500	1.52617000
B	-1.67656100	-1.29782500	0.94352400
B	-1.67656100	-1.29782500	-0.94352400
B	-1.67633300	0.49589500	-1.52617000
B	-3.17086500	-1.52337600	0.00000000
B	-3.17120200	-0.47058700	-1.44873300
B	-3.17114400	1.23228300	-0.89542800
B	-3.17114400	1.23228300	0.89542800
B	-3.17120200	-0.47058700	1.44873300
B	-4.11278600	-0.00015100	0.00000000
B	1.67682400	-1.60504200	0.00000000
B	1.67633300	-0.49589500	-1.52617000
B	1.67656100	1.29782500	-0.94352400
B	1.67656100	1.29782500	0.94352400
B	1.67633300	-0.49589500	1.52617000
B	3.17086500	1.52337600	0.00000000
B	3.17120200	0.47058700	1.44873300
B	3.17114400	-1.23228300	0.89542800
B	3.17114400	-1.23228300	-0.89542800
B	3.17120200	0.47058700	-1.44873300
B	4.11278600	0.00015100	0.00000000
F	-1.26201600	2.91669000	0.00000000
F	-1.25822700	0.90050600	2.77268000
F	-1.25858500	-2.35752700	1.71447100
F	-1.25858500	-2.35752700	-1.71447100
F	-1.25822700	0.90050600	-2.77268000
F	-3.80520100	-2.75419000	0.00000000
F	-3.80591600	-0.85078300	-2.61913400
F	-3.80530200	2.22794300	-1.61915100
F	-3.80530200	2.22794300	1.61915100
F	-3.80591600	-0.85078300	2.61913400
F	-5.48961200	-0.00036300	0.00000000
F	1.26201600	-2.91669000	0.00000000
F	1.25822700	-0.90050600	-2.77268000
F	1.25858500	2.35752700	-1.71447100
F	1.25858500	2.35752700	1.71447100
F	1.25822700	-0.90050600	2.77268000
F	3.80520100	2.75419000	0.00000000
F	3.80591600	0.85078300	2.61913400
F	3.80530200	-2.22794300	1.61915100
F	3.80530200	-2.22794300	-1.61915100
F	3.80591600	0.85078300	-2.61913400
F	5.48961200	0.00036300	0.00000000

E(RB3LYP) = -2881.80958488

Zero-point correction= 0.160073 (Hartree/Particle)
Thermal correction to Energy= 0.202227
Thermal correction to Enthalpy= 0.203171
Thermal correction to Gibbs Free Energy= 0.092523
Sum of electronic and zero-point Energies= -2881.649512
Sum of electronic and thermal Energies= -2881.607358
Sum of electronic and thermal Enthalpies= -2881.606414
Sum of electronic and thermal Free Energies= -2881.717062

	E (Thermal)	CV	S
	KCal/Mol	Cal/Mol-Kelvin	Cal/Mol-Kelvin
Total	126.899	160.701	232.878

	1	2	3
	A(A1U)	A(E1U)	A(E1U)
Frequencies --	29.1005	49.0846	49.2795

Part 2. $[Au(B_{11}F_{11})_2]^{3-}$: η^2,η^2; PG C_{2h}

Au	0.00000000	0.00000000	0.00000000
B	-1.81884100	3.17410800	0.00000000
B	-0.81920400	2.56412000	1.24456400
B	1.15165000	1.85081300	0.85808800
B	1.15165000	1.85081300	-0.85808900
B	-0.81920400	2.56412000	-1.24456400
B	1.75149300	3.28639800	0.00000000
B	0.66521900	3.50044100	-1.41196900
B	-0.90611900	4.33532600	-0.88780200
B	-0.90611900	4.33532600	0.88780200
B	0.66522000	3.50044100	1.41196800
B	0.62286100	4.64207500	0.00000000
B	1.81884100	-3.17410800	0.00000000
B	0.81920400	-2.56412000	1.24456400
B	-1.15165000	-1.85081300	0.85808800
B	-1.15165000	-1.85081300	-0.85808900
B	0.81920400	-2.56412000	-1.24456400
B	-1.75149300	-3.28639800	0.00000000
B	-0.66521900	-3.50044100	-1.41196900
B	0.90611900	-4.33532600	-0.88780200
B	0.90611900	-4.33532600	0.88780200
B	-0.66522000	-3.50044100	1.41196800
B	-0.62286100	-4.64207500	0.00000000
F	-3.20355100	3.00806100	0.00000100
F	-1.38526900	1.88184100	2.28191900
F	1.91600000	1.27171400	1.84743900
F	1.91599900	1.27171400	-1.84744000
F	-1.38526900	1.88184200	-2.28191900
F	3.10579400	3.59007900	-0.00000100
F	1.18085900	3.85876900	-2.64862300
F	-1.40561600	5.31981300	-1.71961100
F	-1.40561600	5.31981300	1.71961200
F	1.18086000	3.85876800	2.64862300
F	1.16067700	5.91175700	0.00000000
F	3.20355100	-3.00806100	0.00000100
F	1.38526900	-1.88184100	2.28191900
F	-1.91600000	-1.27171400	1.84743900
F	-1.91599900	-1.27171400	-1.84744000
F	1.38526900	-1.88184200	-2.28191900
F	-3.10579400	-3.59007900	-0.00000100
F	-1.18085900	-3.85876900	-2.64862300
F	1.40561600	-5.31981300	-1.71961100
F	1.40561600	-5.31981300	1.71961200
F	-1.18086000	-3.85876800	2.64862300
F	-1.16067700	-5.91175700	0.00000000

E(RB3LYP) = -2881.73370164
Zero-point correction= 0.157130 (Hartree/Particle)
Thermal correction to Energy= 0.200478
Thermal correction to Enthalpy= 0.201422
Thermal correction to Gibbs Free Energy= 0.084378
Sum of electronic and zero-point Energies= -2881.576572
Sum of electronic and thermal Energies= -2881.533224
Sum of electronic and thermal Enthalpies= -2881.532280
Sum of electronic and thermal Free Energies= -2881.649324

	E (Thermal)	CV	S
	KCal/Mol	Cal/Mol-Kelvin	Cal/Mol-Kelvin
Total	125.802	160.595	246.341

	1	2	3
	A(Au)	A(Bu)	A(Au)
Frequencies --	-29.3025	18.4047	20.8039

Part 3. $[Cu(B_{11}H_{11})_2]^{3-}$: η^5,η^5; PG D_{5d}

Cu	0.00000000	0.00000000	0.00000000
B	0.00000000	1.57010600	-1.52564400
B	1.49363900	0.48514600	-1.52591100
B	0.92319800	-1.27055000	-1.52596100
B	-0.92319800	-1.27055000	-1.52596100
B	-1.49363900	0.48514600	-1.52591100
B	0.00000000	-1.52336200	-3.02633000
B	-1.44890400	-0.47065400	-3.02637400
B	-0.89543500	1.23259600	-3.02616600
B	0.89543500	1.23259600	-3.02616600
B	1.44890400	-0.47065400	-3.02637400
B	0.00000000	0.00016100	-3.96666700
B	0.00000000	-1.57010600	1.52564400
B	-1.49363900	-0.48514600	1.52591100
B	-0.92319800	1.27055000	1.52596100
B	0.92319800	1.27055000	1.52596100
B	1.49363900	-0.48514600	1.52591100
B	0.00000000	1.52336200	3.02633000
B	1.44890400	0.47065400	3.02637400
B	0.89543500	-1.23259600	3.02616600
B	-0.89543500	-1.23259600	3.02616600
B	-1.44890400	0.47065400	3.02637400
B	0.00000000	-0.00016100	3.96666700
H	0.00000000	2.67406800	-1.06151500
H	2.54358700	0.82625900	-1.06179000
H	1.57206800	-2.16388500	-1.06214900
H	-1.57206800	-2.16388500	-1.06214900
H	-2.54358700	0.82625900	-1.06179000
H	0.00000000	-2.60676900	-3.56476900
H	-2.47930400	-0.80545300	-3.56477000
H	-1.53220000	2.10927000	-3.56435500
H	1.53220000	2.10927000	-3.56435400
H	2.47930400	-0.80545300	-3.56477000
H	0.00000000	0.00026900	-5.17871100
H	0.00000000	-2.67406800	1.06151500
H	-2.54358700	-0.82625900	1.06179000
H	-1.57206800	2.16388500	1.06214900
H	1.57206800	2.16388500	1.06214900
H	2.54358700	-0.82625900	1.06179000
H	0.00000000	2.60676900	3.56476900
H	2.47930400	0.80545300	3.56477000
H	1.53220000	-2.10927000	3.56435500
H	-1.53220000	-2.10927000	3.56435400
H	-2.47930400	0.80545300	3.56477000
H	0.00000000	-0.00026900	5.17871100

E(RB3LYP) = -757.634765261

Zero-point correction= 0.300895 (Hartree/Particle)
Thermal correction to Energy= 0.319167
Thermal correction to Enthalpy= 0.320111
Thermal correction to Gibbs Free Energy= 0.260121
Sum of electronic and zero-point Energies= -757.333870
Sum of electronic and thermal Energies= -757.315599
Sum of electronic and thermal Enthalpies= -757.314654
Sum of electronic and thermal Free Energies= -757.374644

E (Thermal)	CV	S
KCal/Mol	Cal/Mol-Kelvin	Cal/Mol-Kelvin

	1	2	3
	AU(A1U)	AU(E1U)	AU(E1U)
Frequencies --	85.2211	98.5584	99.1379

Part 3. $[Cu(B_{11}H_{11})_2]^{3-}$: η^2,η^2; PG C_2

Cu	0.00000000	0.00000000	0.09184700
B	0.69109200	4.54551400	-1.26498500
B	1.21470900	2.95616700	-1.48047800
B	1.50236400	1.50955100	-0.33186500
B	0.49149100	2.06280000	0.87117200
B	0.00000000	3.63815800	-0.02248000
B	2.26013500	2.26724800	1.10495100
B	1.09160600	3.57648900	1.46042700
B	1.29735100	4.96073600	0.29249800
B	2.38262200	4.35508000	-1.01013100
B	2.78934300	2.63250900	-0.56959600
B	2.70261300	3.93148700	0.69788700
B	-0.69109200	-4.54551400	-1.26498500
B	0.00000000	-3.63815800	-0.02248000
B	-0.49149100	-2.06280000	0.87117200
B	-1.50236400	-1.50955100	-0.33186500
B	-1.21470900	-2.95616700	-1.48047800
B	-2.26013500	-2.26724800	1.10495100
B	-2.78934300	-2.63250900	-0.56959600
B	-2.38262200	-4.35508000	-1.01013100
B	-1.29735100	-4.96073600	0.29249800
B	-1.09160600	-3.57648900	1.46042700
B	-2.70261300	-3.93148700	0.69788700
H	0.09863900	5.21554800	-2.07317000
H	0.93150000	2.47726300	-2.55012800
H	1.68092200	0.38817400	-0.80109100
H	-0.32128200	1.49814200	1.57889500
H	-1.20499900	3.67368900	0.01490500
H	2.88871200	1.64165900	1.92035800
H	0.74819100	3.92144900	2.56484600
H	1.20889900	6.08011300	0.74527200
H	3.20817100	4.96350000	-1.65332600
H	3.80103200	2.22810900	-1.08792500
H	3.65542100	4.40282400	1.27257900
H	-0.09863900	-5.21554800	-2.07317000
H	1.20499900	-3.67368900	0.01490500
H	0.32128200	-1.49814200	1.57889500
H	-1.68092200	-0.38817400	-0.80109100
H	-0.93150000	-2.47726300	-2.55012800
H	-2.88871200	-1.64165900	1.92035800
H	-3.80103200	-2.22810900	-1.08792500
H	-3.20817100	-4.96350000	-1.65332600
H	-1.20889900	-6.08011300	0.74527200
H	-0.74819100	-3.92144900	2.56484600
H	-3.65542100	-4.40282400	1.27257900

E(RB3LYP) = -757.639742676
Zero-point correction= 0.294333 (Hartree/Particle)
Thermal correction to Energy= 0.315811
Thermal correction to Enthalpy= 0.316756
Thermal correction to Gibbs Free Energy= 0.245002
Sum of electronic and zero-point Energies= -757.345409
Sum of electronic and thermal Energies= -757.323931
Sum of electronic and thermal Enthalpies= -757.322987
Sum of electronic and thermal Free Energies= -757.394741

	E (Thermal)	CV	S
	KCal/Mol	Cal/Mol-Kelvin	Cal/Mol-Kelvin
Total	198.175	98.597	151.019

	1	2	3
	A	A	B
Frequencies --	22.8836	31.7320	31.9000

Part 3. $[Cu(B_{11}H_{11})_2]^{3-}$: η^2,η^2; PG C_{2h}

Cu	0.00000000	0.00000000	0.00000000
B	-1.61226100	4.22126500	0.00000000
B	-0.86150900	3.10676500	1.02113600
B	0.63447400	1.96285500	0.83913500
B	0.63447400	1.96285500	-0.83913500
B	-0.86150900	3.10676500	-1.02113600
B	1.81729200	3.01108400	0.00000000
B	0.86150900	3.59205200	-1.40112900
B	-0.30528600	4.89429600	-0.90027800
B	-0.30528600	4.89429600	0.90027800
B	0.86150900	3.59205200	1.40112900
B	1.22466600	4.68306500	0.00000000
B	1.61226100	-4.22126500	0.00000000
B	0.86150900	-3.10676500	1.02113600
B	-0.63447400	-1.96285500	0.83913500
B	-0.63447400	-1.96285500	-0.83913500
B	0.86150900	-3.10676500	-1.02113600
B	-1.81729200	-3.01108400	0.00000000
B	-0.86150900	-3.59205200	-1.40112900
B	0.30528600	-4.89429600	-0.90027800
B	0.30528600	-4.89429600	0.90027800
B	-0.86150900	-3.59205200	1.40112900
B	-1.22466600	-4.68306500	0.00000000
H	-2.78289700	4.50775900	0.00000000
H	-1.61091800	2.55280100	1.78534100
H	0.85323600	1.11764600	1.67844100
H	0.85323600	1.11764600	-1.67844100
H	-1.61091800	2.55280100	-1.78534100
H	3.00294900	2.79081700	0.00000000
H	1.29181500	3.72225800	-2.52110600
H	-0.45049900	5.82815800	-1.65699100
H	-0.45049900	5.82815800	1.65699100
H	1.29181500	3.72225800	2.52110600
H	2.06067900	5.55582700	0.00000000
H	2.78289700	-4.50775900	0.00000000
H	1.61091800	-2.55280100	1.78534100
H	-0.85323600	-1.11764600	1.67844100
H	-0.85323600	-1.11764600	-1.67844100
H	1.61091800	-2.55280100	-1.78534100
H	-3.00294900	-2.79081700	0.00000000
H	-1.29181500	-3.72225800	-2.52110600
H	0.45049900	-5.82815800	-1.65699100
H	0.45049900	-5.82815800	1.65699100
H	-1.29181500	-3.72225800	2.52110600
H	-2.06067900	-5.55582700	0.00000000

E(RB3LYP) = -757.63370368

Zero-point correction= 0.294632 (Hartree/Particle)
Thermal correction to Energy= 0.315172
Thermal correction to Enthalpy= 0.316116
Thermal correction to Gibbs Free Energy= 0.247150
Sum of electronic and zero-point Energies= -757.339072
Sum of electronic and thermal Energies= -757.318532
Sum of electronic and thermal Enthalpies= -757.317588
Sum of electronic and thermal Free Energies= -757.386554

	E (Thermal)	CV	S
	KCal/Mol	Cal/Mol-Kelvin	Cal/Mol-Kelvin
Total	197.773	96.905	145.151

	1	2	3
	AU	BU	BG
Frequencies --	-24.3379	17.3539	34.6945

Part 3. $[Ag(B_{11}H_{11})_2]^{3-}$: η[5],η[5]; PG D_{5d}

Ag	0.00000000	0.00000000	0.00000000
B	0.00000000	1.58302100	-1.69401900
B	1.50564100	0.48923600	-1.69409300
B	0.93074700	-1.28075900	-1.69434300
B	-0.93074700	-1.28075900	-1.69434300
B	-1.50564100	0.48923600	-1.69409300
B	0.00000000	-1.52487700	-3.18885400
B	-1.45034400	-0.47106600	-3.18889300
B	-0.89628600	1.23379700	-3.18876700
B	0.89628700	1.23379700	-3.18876700
B	1.45034400	-0.47106600	-3.18889300
B	0.00000000	0.00010000	-4.12692500
B	0.00000000	-1.58302100	1.69401900
B	-1.50564100	-0.48923600	1.69409300
B	-0.93074700	1.28075900	1.69434300
B	0.93074700	1.28075900	1.69434300
B	1.50564100	-0.48923600	1.69409300
B	0.00000000	1.52487700	3.18885400
B	1.45034400	0.47106600	3.18889300
B	0.89628600	-1.23379700	3.18876700
B	-0.89628700	-1.23379700	3.18876700
B	-1.45034400	0.47106600	3.18889300
B	0.00000000	-0.00010000	4.12692500
H	0.00000000	2.68434900	-1.21972800
H	2.55298700	0.82968300	-1.21972200
H	1.57819300	-2.17172900	-1.22019100
H	-1.57819300	-2.17172900	-1.22019100
H	-2.55298700	0.82968300	-1.21972200
H	0.00000000	-2.60363300	-3.73639600
H	-2.47629000	-0.80441600	-3.73645400
H	-1.53032000	2.10658000	-3.73626200
H	1.53032000	2.10658000	-3.73626200
H	2.47629000	-0.80441600	-3.73645400
H	0.00000000	0.00015500	-5.33774400
H	0.00000000	-2.68434900	1.21972800
H	-2.55298700	-0.82968300	1.21972200
H	-1.57819300	2.17172900	1.22019100
H	1.57819300	2.17172900	1.22019100
H	2.55298700	-0.82968300	1.21972200
H	0.00000000	2.60363300	3.73639600
H	2.47629000	0.80441600	3.73645400
H	1.53032000	-2.10658000	3.73626200
H	-1.53032000	-2.10658000	3.73626200
H	-2.47629000	0.80441600	3.73645400
H	0.00000000	-0.00015500	5.33774400

E(RB3LYP) = -707.246415695

Zero-point correction= 0.298956 (Hartree/Particle)
Thermal correction to Energy= 0.317875
Thermal correction to Enthalpy= 0.318819
Thermal correction to Gibbs Free Energy= 0.256680
Sum of electronic and zero-point Energies= -706.947460
Sum of electronic and thermal Energies= -706.928540
Sum of electronic and thermal Enthalpies= -706.927596
Sum of electronic and thermal Free Energies= -706.989736

	E (Thermal)	CV	S
	KCal/Mol	Cal/Mol-Kelvin	Cal/Mol-Kelvin
Total	199.470	96.594	130.784

	1	2	3
	AU(A1U)	AU(E1U)	AU(E1U)
Frequencies --	48.3988	82.8881	83.1366

Part 3. $[Ag(B_{11}H_{11})_2]^{3-}$: η^2,η^2; PG C_2

Ag	0.00000000	0.00000000	0.10646000
B	0.49398400	4.68879400	-1.22127200
B	1.10296100	3.12854300	-1.44031000
B	1.62560300	1.73610700	-0.30797700
B	0.69225100	2.26650300	0.97876800
B	0.00000000	3.79493800	0.11891300
B	2.45265700	2.59762600	1.02330500
B	1.24306500	3.84524700	1.46313200
B	1.22967700	5.20155700	0.25412900
B	2.21084500	4.62955700	-1.14194900
B	2.78580700	2.95647600	-0.70365600
B	2.73624300	4.28062600	0.53078500
B	-0.49398400	-4.68879400	-1.22127200
B	0.00000000	-3.79493800	0.11891300
B	-0.69225100	-2.26650300	0.97876800
B	-1.62560300	-1.73610700	-0.30797700
B	-1.10296100	-3.12854300	-1.44031000
B	-2.45265700	-2.59762600	1.02330500
B	-2.78580700	-2.95647600	-0.70365600
B	-2.21084500	-4.62955700	-1.14194900
B	-1.22967700	-5.20155700	0.25412900
B	-1.24306500	-3.84524700	1.46313200
B	-2.73624300	-4.28062600	0.53078500
H	-0.22485300	5.28601500	-1.98212000
H	0.73336600	2.60077800	-2.45942900
H	1.90880500	0.64509600	-0.78044800
H	0.00867700	1.71092800	1.81148900
H	-1.19382700	3.75585800	0.28752300
H	3.20799800	2.05202500	1.78839800
H	0.99125500	4.18768500	2.59265800
H	1.10875800	6.32771900	0.68085800
H	2.91774200	5.26908500	-1.88749800
H	3.76514900	2.60753200	-1.31550300
H	3.71031500	4.82564500	0.99293400
H	0.22485300	-5.28601500	-1.98212000
H	1.19382700	-3.75585800	0.28752300
H	-0.00867700	-1.71092800	1.81148900
H	-1.90880500	-0.64509600	-0.78044800
H	-0.73336600	-2.60077800	-2.45942900
H	-3.20799800	-2.05202500	1.78839800
H	-3.76514900	-2.60753200	-1.31550300
H	-2.91774200	-5.26908500	-1.88749800
H	-1.10875800	-6.32771900	0.68085800
H	-0.99125500	-4.18768500	2.59265800
H	-3.71031500	-4.82564500	0.99293400

E(RB3LYP) = -707.268625311

Zero-point correction= 0.294287 (Hartree/Particle)
Thermal correction to Energy= 0.315950
Thermal correction to Enthalpy= 0.316894
Thermal correction to Gibbs Free Energy= 0.243355
Sum of electronic and zero-point Energies= -706.974338
Sum of electronic and thermal Energies= -706.952675
Sum of electronic and thermal Enthalpies= -706.951731
Sum of electronic and thermal Free Energies= -707.025270

	E (Thermal)	CV	S
	KCal/Mol	Cal/Mol-Kelvin	Cal/Mol-Kelvin
Total	198.262	98.734	154.777

	1	2	3
	A	A	B
Frequencies --	19.8043	24.0095	25.7227

Part 3. $[Ag(B_{11}H_{11})_2]^{3-}$: η²,η²; PG C_{2h}

Ag	0.00000000	0.00000000	0.00000000
B	-1.63684800	4.41149300	0.00000000
B	-0.85339700	3.31727600	1.01800000
B	0.67211700	2.21935400	0.84149800
B	0.67211700	2.21935400	-0.84149800
B	-0.85339700	3.31727600	-1.01800000
B	1.82435500	3.29567900	0.00000000
B	0.85339700	3.85667500	-1.40100800
B	-0.34978600	5.12351800	-0.89984300
B	-0.34978600	5.12351800	0.89984300
B	0.85339700	3.85667500	1.40100800
B	1.18437500	4.95500200	0.00000000
B	1.63684800	-4.41149300	0.00000000
B	0.85339700	-3.31727600	1.01800000
B	-0.67211700	-2.21935400	0.84149800
B	-0.67211700	-2.21935400	-0.84149800
B	0.85339700	-3.31727600	-1.01800000
B	-1.82435500	-3.29567900	0.00000000
B	-0.85339700	-3.85667500	-1.40100800
B	0.34978600	-5.12351800	-0.89984300
B	0.34978600	-5.12351800	0.89984300
B	-0.85339700	-3.85667500	1.40100800
B	-1.18437500	-4.95500200	0.00000000
H	-2.81508300	4.66355500	0.00000000
H	-1.58873900	2.74117300	1.78043500
H	0.92368200	1.40072600	1.69846400
H	0.92368200	1.40072600	-1.69846400
H	-1.58873900	2.74117300	-1.78043500
H	3.01636300	3.11090300	0.00000000
H	1.27899800	3.99908700	-2.52114100
H	-0.52349300	6.05209100	-1.65645500
H	-0.52349300	6.05209100	1.65645500
H	1.27899800	3.99908700	2.52114100
H	1.99850400	5.84764800	0.00000000
H	2.81508300	-4.66355500	0.00000000
H	1.58873900	-2.74117300	1.78043500
H	-0.92368200	-1.40072600	1.69846400
H	-0.92368200	-1.40072600	-1.69846400
H	1.58873900	-2.74117300	-1.78043500
H	-3.01636300	-3.11090300	0.00000000
H	-1.27899800	-3.99908700	-2.52114100
H	0.52349300	-6.05209100	-1.65645500
H	0.52349300	-6.05209100	1.65645500
H	-1.27899800	-3.99908700	2.52114100
H	-1.99850400	-5.84764800	0.00000000

E(RB3LYP) = -707.266799284

Zero-point correction= 0.294653 (Hartree/Particle)
Thermal correction to Energy= 0.315303
Thermal correction to Enthalpy= 0.316248
Thermal correction to Gibbs Free Energy= 0.246412
Sum of electronic and zero-point Energies= -706.972146
Sum of electronic and thermal Energies= -706.951496
Sum of electronic and thermal Enthalpies= -706.950552
Sum of electronic and thermal Free Energies= -707.020387

	E (Thermal)	CV	S
	KCal/Mol	Cal/Mol-Kelvin	Cal/Mol-Kelvin
Total	197.856	96.778	146.981

	1	2	3
	AU	BU	AU
Frequencies --	-16.8046	21.7315	32.4568

Part 3. $[Au(B_{11}H_{11})_2]^{3-}$: η^5,η^5; PG D_{5d}

Au	0.00000000	0.00000000	0.00000000
B	0.00000000	1.59022000	-1.67033300
B	1.51252200	0.49142600	-1.67046700
B	0.93494100	-1.28654500	-1.67069200
B	-0.93494000	-1.28654500	-1.67069200
B	-1.51252200	0.49142600	-1.67046700
B	0.00000000	-1.52313900	-3.16489300
B	-1.44869400	-0.47054000	-3.16493000
B	-0.89526800	1.23239200	-3.16478200
B	0.89526800	1.23239200	-3.16478200
B	1.44869400	-0.47054000	-3.16493000
B	0.00000000	0.00010300	-4.10219500
B	0.00000000	-1.59022000	1.67033300
B	-1.51252200	-0.49142600	1.67046700
B	-0.93494100	1.28654500	1.67069200
B	0.93494000	1.28654500	1.67069200
B	1.51252200	-0.49142600	1.67046700
B	0.00000000	1.52313900	3.16489300
B	1.44869400	0.47054000	3.16493000
B	0.89526800	-1.23239200	3.16478200
B	-0.89526800	-1.23239200	3.16478200
B	-1.44869400	0.47054000	3.16493000
B	0.00000000	-0.00010300	4.10219500
H	0.00000000	2.69917100	-1.21459600
H	2.56711600	0.83422700	-1.21463900
H	1.58685300	-2.18368700	-1.21511200
H	-1.58685300	-2.18368700	-1.21511200
H	-2.56711600	0.83422700	-1.21463900
H	0.00000000	-2.60014800	-3.71538500
H	-2.47296400	-0.80336300	-3.71545800
H	-1.52825600	2.10375400	-3.71526100
H	1.52825600	2.10375400	-3.71526100
H	2.47296400	-0.80336300	-3.71545800
H	0.00000000	0.00015500	-5.31326000
H	0.00000000	-2.69917100	1.21459600
H	-2.56711600	-0.83422700	1.21463900
H	-1.58685300	2.18368700	1.21511200
H	1.58685300	2.18368700	1.21511200
H	2.56711600	-0.83422700	1.21463900
H	0.00000000	2.60014800	3.71538500
H	2.47296400	0.80336300	3.71545800
H	1.52825600	-2.10375400	3.71526100
H	-1.52825600	-2.10375400	3.71526100
H	-2.47296400	0.80336300	3.71545800
H	0.00000000	-0.00015500	5.31326000

E(RB3LYP) = -696.089591388

Zero-point correction= 0.300683 (Hartree/Particle)
Thermal correction to Energy= 0.319187
Thermal correction to Enthalpy= 0.320131
Thermal correction to Gibbs Free Energy= 0.258542
Sum of electronic and zero-point Energies= -695.788909
Sum of electronic and thermal Energies= -695.770404
Sum of electronic and thermal Enthalpies= -695.769460
Sum of electronic and thermal Free Energies= -695.831049

	E (Thermal)	CV	S
	KCal/Mol	Cal/Mol-Kelvin	Cal/Mol-Kelvin
Total	200.293	95.362	129.625

	1	2	3
	AU(A1U)	AU(E1U)	AU(E1U)
Frequencies --	52.6586	84.1280	84.2334

Part 3. $[Au(B_{11}H_{11})_2]^{3-}$: η^2,η^2; PG C_2

Au	0.00000000	0.00000000	0.19439700
B	-1.47244600	3.98180400	-0.81877800
B	-0.31224400	2.88338900	-1.37121800
B	1.13705400	1.94020600	-0.52084000
B	0.53753900	2.13681000	1.07489500
B	-1.03924000	3.12401000	0.57035800
B	1.82364900	3.23624300	0.50264100
B	0.39455200	3.85954400	1.39309000
B	-0.62439800	4.93024800	0.34969500
B	0.00000000	4.72519300	-1.32576500
B	1.36632700	3.54163700	-1.20649400
B	1.13359400	4.81330600	0.05204200
B	1.47244600	-3.98180400	-0.81877800
B	1.03924000	-3.12401000	0.57035800
B	-0.53753900	-2.13681000	1.07489500
B	-1.13705400	-1.94020600	-0.52084000
B	0.31224400	-2.88338900	-1.37121800
B	-1.82364900	-3.23624300	0.50264100
B	-1.36632700	-3.54163700	-1.20649400
B	0.00000000	-4.72519300	-1.32576500
B	0.62439800	-4.93024800	0.34969500
B	-0.39455200	-3.85954400	1.39309000
B	-1.13359400	-4.81330600	0.05204200
H	-2.58922400	4.10851400	-1.25155300
H	-0.70435200	2.16669000	-2.25407200
H	1.79877700	1.13918600	-1.13391300
H	0.57403700	1.53598200	2.11957800
H	-1.96335800	2.58260500	1.12045300
H	2.95150000	3.19471400	0.93061300
H	0.41124800	4.15785100	2.56205500
H	-1.10422100	5.91712200	0.86077800
H	0.04489800	5.53954900	-2.21993900
H	2.15734600	3.59195200	-2.11526000
H	1.85483700	5.77068700	0.20444600
H	2.58922400	-4.10851400	-1.25155300
H	1.96335800	-2.58260500	1.12045300
H	-0.57403700	-1.53598200	2.11957800
H	-1.79877700	-1.13918600	-1.13391300
H	0.70435200	-2.16669000	-2.25407200
H	-2.95150000	-3.19471400	0.93061300
H	-2.15734600	-3.59195200	-2.11526000
H	-0.04489800	-5.53954900	-2.21993900
H	1.10422100	-5.91712200	0.86077800
H	-0.41124800	-4.15785100	2.56205500
H	-1.85483700	-5.77068700	0.20444600

E(RB3LYP) = -696.044568497

Zero-point correction= 0.295360 (Hartree/Particle)
Thermal correction to Energy= 0.316466
Thermal correction to Enthalpy= 0.317410
Thermal correction to Gibbs Free Energy= 0.246166
Sum of electronic and zero-point Energies= -695.749209
Sum of electronic and thermal Energies= -695.728103
Sum of electronic and thermal Enthalpies= -695.727158
Sum of electronic and thermal Free Energies= -695.798402

	E (Thermal)	CV	S
	KCal/Mol	Cal/Mol-Kelvin	Cal/Mol-Kelvin
Total	198.585	98.800	149.945

	1	2	3
	A	B	A
Frequencies --	12.8705	25.0147	31.9000

Part 3. $[Au(B_{11}H_{11})_2]^{3-}$: η^2,η^2; PG C_{2h}

Au	0.00000000	0.00000000	0.00000000
B	-1.73561700	3.97398800	0.00000000
B	-0.83302800	2.99974100	1.04504400
B	0.83302800	2.04536100	0.85836300
B	0.83302800	2.04536100	-0.85836300
B	-0.83302800	2.99974100	-1.04504400
B	1.82097700	3.26119400	0.00000000
B	0.78935700	3.71826700	-1.39675400
B	-0.54269100	4.83917300	-0.89991400
B	-0.54269100	4.83917300	0.89991400
B	0.78935700	3.71826700	1.39675400
B	1.00043100	4.84071900	0.00000000
B	1.73561700	-3.97398800	0.00000000
B	0.83302800	-2.99974100	1.04504400
B	-0.83302800	-2.04536100	0.85836300
B	-0.83302800	-2.04536100	-0.85836300
B	0.83302800	-2.99974100	-1.04504400
B	-1.82097700	-3.26119400	0.00000000
B	-0.78935700	-3.71826700	-1.39675400
B	0.54269100	-4.83917300	-0.89991400
B	0.54269100	-4.83917300	0.89991400
B	-0.78935700	-3.71826700	1.39675400
B	-1.00043100	-4.84071900	0.00000000
H	-2.93466700	4.08854800	0.00000000
H	-1.50250500	2.36134600	1.81474600
H	1.24553400	1.34488200	1.74859300
H	1.24553400	1.34488200	-1.74859300
H	-1.50250500	2.36134600	-1.81474600
H	3.02748200	3.23336900	0.00000000
H	1.21147500	3.89782800	-2.51236700
H	-0.82356000	5.74279600	-1.65466300
H	-0.82356000	5.74279600	1.65466300
H	1.21147500	3.89782800	2.51236700
H	1.72019000	5.81117100	0.00000000
H	2.93466700	-4.08854800	0.00000000
H	1.50250500	-2.36134600	1.81474600
H	-1.24553400	-1.34488200	1.74859300
H	-1.24553400	-1.34488200	-1.74859300
H	1.50250500	-2.36134600	-1.81474600
H	-3.02748200	-3.23336900	0.00000000
H	-1.21147500	-3.89782800	-2.51236700
H	0.82356000	-5.74279600	-1.65466300
H	0.82356000	-5.74279600	1.65466300
H	-1.21147500	-3.89782800	2.51236700
H	-1.72019000	-5.81117100	0.00000000

E(RB3LYP) = -696.044480885
Zero-point correction= 0.295366 (Hartree/Particle)
Thermal correction to Energy= 0.316487
Thermal correction to Enthalpy= 0.317431
Thermal correction to Gibbs Free Energy= 0.246111
Sum of electronic and zero-point Energies= -695.749115
Sum of electronic and thermal Energies= -695.727994
Sum of electronic and thermal Enthalpies= -695.727050
Sum of electronic and thermal Free Energies= -695.798370

	E (Thermal)	CV	S
	KCal/Mol	Cal/Mol-Kelvin	Cal/Mol-Kelvin
Total	198.598	98.813	150.106

	1	2	3
	AU	BU	AU
Frequencies --	14.8982	19.5577	37.1615

Part 3. $[Cu(B_{11}F_{11})_2]^{3-}$: η[5],η[5]; PG D_{5d}

Cu	0.00000000	0.00000000	0.00000000
B	-1.52722700	1.58653300	0.00000000
B	-1.52724500	0.49031000	-1.50905400
B	-1.52711800	-1.28349100	-0.93276900
B	-1.52711800	-1.28349100	0.93276900
B	-1.52724500	0.49031000	1.50905400
B	-3.04030100	-1.52772600	0.00000000
B	-3.04051200	-0.47206000	1.45298600
B	-3.04054100	1.23595300	0.89794800
B	-3.04054100	1.23595300	-0.89794800
B	-3.04051200	-0.47206000	-1.45298600
B	-3.98969900	-0.00010500	0.00000000
B	1.52722700	-1.58653300	0.00000000
B	1.52724500	-0.49031000	1.50905400
B	1.52711800	1.28349100	0.93276900
B	1.52711800	1.28349100	-0.93276900
B	1.52724500	-0.49031000	-1.50905400
B	3.04030100	1.52772600	0.00000000
B	3.04051200	0.47206000	-1.45298600
B	3.04054100	-1.23595300	-0.89794800
B	3.04054100	-1.23595300	0.89794800
B	3.04051200	0.47206000	1.45298600
B	3.98969900	0.00010500	0.00000000
F	-1.12184700	2.90625300	0.00000000
F	-1.12179900	0.89803700	-2.76416200
F	-1.12184800	-2.35122200	-1.70838700
F	-1.12184800	-2.35122200	1.70838700
F	-1.12179900	0.89803700	2.76416200
F	-3.67974700	-2.76869600	0.00000000
F	-3.68012800	-0.85554000	2.63311100
F	-3.68010200	2.23988700	1.62729000
F	-3.68010200	2.23988700	-1.62729000
F	-3.68012800	-0.85554000	-2.63311100
F	-5.38084900	-0.00019300	0.00000000
F	1.12184700	-2.90625300	0.00000000
F	1.12179900	-0.89803700	2.76416200
F	1.12184800	2.35122200	1.70838700
F	1.12184800	2.35122200	-1.70838700
F	1.12179900	-0.89803700	-2.76416200
F	3.67974700	2.76869600	0.00000000
F	3.68012800	0.85554000	-2.63311100
F	3.68010200	-2.23988700	-1.62729000
F	3.68010200	-2.23988700	1.62729000
F	3.68012800	0.85554000	2.63311100
F	5.38084900	0.00019300	0.00000000

E(RB3LYP) = -2942.37534916

Zero-point correction= 0.157110 (Hartree/Particle)
Thermal correction to Energy= 0.201475
Thermal correction to Enthalpy= 0.202419
Thermal correction to Gibbs Free Energy= 0.082130
Sum of electronic and zero-point Energies= -2942.218239
Sum of electronic and thermal Energies= -2942.173874
Sum of electronic and thermal Enthalpies= -2942.172930
Sum of electronic and thermal Free Energies= -2942.293220

	E (Thermal)	CV	S
	KCal/Mol	Cal/Mol-Kelvin	Cal/Mol-Kelvin
Total	126.427	162.953	253.170

	1	2	3
	A(A1U)	A(E1U)	A(E1U)
Frequencies --	12.7554	17.0905	25.7521

Part 3. $[Cu(B_{11}F_{11})_2]^{3-}$: η^2,η^2; PG C_2

Cu	0.00000000	0.00000000	0.23596900
B	1.73095100	3.15555000	-0.72632600
B	0.36196900	2.37925900	-1.40824900
B	-1.28143300	1.71636700	-0.32756400
B	-0.66896200	1.85811200	1.24081100
B	1.23217100	2.57272500	0.80468900
B	-1.62982400	3.22424700	0.58482900
B	-0.10574200	3.55217200	1.47650000
B	1.14519200	4.35195500	0.36299600
B	0.49232600	4.20473400	-1.29916000
B	-1.13350300	3.32212500	-1.13609100
B	-0.61606200	4.58698800	0.06806000
B	-1.73095100	-3.15555000	-0.72632600
B	-1.23217100	-2.57272500	0.80468900
B	0.66896200	-1.85811200	1.24081100
B	1.28143300	-1.71636700	-0.32756400
B	-0.36196900	-2.37925900	-1.40824900
B	1.62982400	-3.22424700	0.58482900
B	1.13350300	-3.32212500	-1.13609100
B	-0.49232600	-4.20473400	-1.29916000
B	-1.14519200	-4.35195500	0.36299600
B	0.10574200	-3.55217200	1.47650000
B	0.61606200	-4.58698800	0.06806000
F	3.03252800	3.02213600	-1.22498600
F	0.55482800	1.55908800	-2.49269500
F	-2.27111100	0.97402500	-0.95657200
F	-0.93279300	1.29223100	2.47889800
F	2.15581400	1.91358800	1.58388600
F	-2.91128000	3.51957900	1.06342900
F	-0.12009000	3.99484100	2.80132000
F	1.88880800	5.41076500	0.89390200
F	0.61907900	5.12450300	-2.34465300
F	-2.06657300	3.56038200	-2.14664800
F	-1.15846600	5.86802400	0.16973200
F	-3.03252800	-3.02213500	-1.22498600
F	-2.15581400	-1.91358800	1.58388600
F	0.93279300	-1.29223100	2.47889800
F	2.27111100	-0.97402500	-0.95657200
F	-0.55482800	-1.55908800	-2.49269500
F	2.91128000	-3.51957900	1.06342900
F	2.06657300	-3.56038200	-2.14664800
F	-0.61907900	-5.12450300	-2.34465300
F	-1.88880800	-5.41076500	0.89390200
F	0.12009000	-3.99484100	2.80132000
F	1.15846600	-5.86802400	0.16973200

E(RB3LYP) = -2942.37534916

Zero-point correction= 0.157110 (Hartree/Particle)
Thermal correction to Energy= 0.201475
Thermal correction to Enthalpy= 0.202419
Thermal correction to Gibbs Free Energy= 0.082130
Sum of electronic and zero-point Energies= -2942.218239
Sum of electronic and thermal Energies= -2942.173874
Sum of electronic and thermal Enthalpies= -2942.172930
Sum of electronic and thermal Free Energies= -2942.293220

	E (Thermal)	CV	S
	KCal/Mol	Cal/Mol-Kelvin	Cal/Mol-Kelvin
Total	126.427	162.953	253.170

	1	2	3
	A(A)	A(B)	A(A)
Frequencies --	12.7554	17.0905	25.7521

Part 3. $[Ag(B_{11}F_{11})_2]^{3-}$: η^5,η^5; PG D_{5d}

Ag	0.00000000	0.00000000	0.00000000
B	-1.70191900	1.59929600	0.00000000
B	-1.70200500	0.49418200	1.52124700
B	-1.70216300	-1.29422500	0.94039100
B	-1.70216300	-1.29422500	-0.94039100
B	-1.70200500	0.49418200	-1.52124700
B	-3.20960800	-1.53035800	0.00000000
B	-3.20967400	-0.47291100	-1.45534900
B	-3.20961400	1.23783900	-0.89935800
B	-3.20961400	1.23783900	0.89935800
B	-3.20967400	-0.47291100	1.45534900
B	-4.15570700	-0.00009800	0.00000000
B	1.70191900	-1.59929600	0.00000000
B	1.70200500	-0.49418200	-1.52124700
B	1.70216300	1.29422500	-0.94039100
B	1.70216300	1.29422500	0.94039100
B	1.70200500	-0.49418200	1.52124700
B	3.20960800	1.53035800	0.00000000
B	3.20967400	0.47291100	1.45534900
B	3.20961400	-1.23783900	0.89935800
B	3.20961400	-1.23783900	-0.89935800
B	3.20967400	0.47291100	-1.45534900
B	4.15570700	0.00009800	0.00000000
F	-1.27371300	2.91310400	0.00000000
F	-1.27369700	0.90044400	2.77063500
F	-1.27320600	-2.35699800	1.71245900
F	-1.27320600	-2.35699800	-1.71245900
F	-1.27369700	0.90044400	-2.77063500
F	-3.85665700	-2.76796800	0.00000000
F	-3.85670600	-0.85522800	-2.63242100
F	-3.85638800	2.23920500	-1.62685700
F	-3.85638800	2.23920500	1.62685700
F	-3.85670600	-0.85522800	2.63242100
F	-5.54768600	-0.00008900	0.00000000
F	1.27371300	-2.91310400	0.00000000
F	1.27369700	-0.90044400	-2.77063500
F	1.27320600	2.35699800	-1.71245900
F	1.27320600	2.35699800	1.71245900
F	1.27369700	-0.90044400	2.77063500
F	3.85665700	2.76796800	0.00000000
F	3.85670600	0.85522800	2.63242100
F	3.85638800	-2.23920500	1.62685700
F	3.85638800	-2.23920500	-1.62685700
F	3.85670600	0.85522800	-2.63242100
F	5.54768600	0.00008900	0.00000000

E(RB3LYP) = -2892.02859002

Zero-point correction=	0.158410 (Hartree/Particle)
Thermal correction to Energy=	0.201157
Thermal correction to Enthalpy=	0.202102
Thermal correction to Gibbs Free Energy=	0.090855
Sum of electronic and zero-point Energies=	-2891.870180
Sum of electronic and thermal Energies=	-2891.827433
Sum of electronic and thermal Enthalpies=	-2891.826488
Sum of electronic and thermal Free Energies=	-2891.937735

	E (Thermal)	CV	S
	KCal/Mol	Cal/Mol-Kelvin	Cal/Mol-Kelvin
Total	126.228	162.162	234.139

	1	2	3
	A(A1U)	A(E1U)	A(E1U)
Frequencies --	28.2615	46.6336	47.2405

Part 3. $[Ag(B_{11}F_{11})_2]^{3-}$: η^2,η^2; PG C_2

Ag	0.00000000	0.00000000	0.16795900
B	1.76645100	3.57184800	-0.67324000
B	0.42754700	2.78019200	-1.38387700
B	-1.24134900	2.09116700	-0.41309900
B	-0.68463200	2.18800700	1.16604700
B	1.19859000	2.91257700	0.79709800
B	-1.64433900	3.57007000	0.53039200
B	-0.15132100	3.87613100	1.47686900
B	1.13508000	4.71590300	0.43756700
B	0.53616800	4.61289900	-1.25950700
B	-1.08605400	3.71638900	-1.16723700
B	-0.61691300	4.95188700	0.08433200
B	-1.76645100	-3.57184800	-0.67324000
B	-1.19859000	-2.91257700	0.79709800
B	0.68463200	-2.18800700	1.16604700
B	1.24134900	-2.09116700	-0.41309900
B	-0.42754700	-2.78019200	-1.38387700
B	1.64433900	-3.57007000	0.53039200
B	1.08605400	-3.71638900	-1.16723700
B	-0.53616800	-4.61289900	-1.25950700
B	-1.13508000	-4.71590300	0.43756700
B	0.15132100	-3.87613100	1.47686900
B	0.61691300	-4.95188700	0.08433200
F	3.08245500	3.43489900	-1.12883200
F	0.67963300	1.94840700	-2.45381000
F	-2.18428700	1.35045100	-1.11667500
F	-0.95079000	1.56664400	2.38016400
F	2.09723300	2.19082800	1.55657800
F	-2.94338700	3.83992300	0.97359400
F	-0.20114700	4.26939000	2.81582600
F	1.84827500	5.76264600	1.02936000
F	0.68235000	5.56236100	-2.27487200
F	-1.97780200	3.96650600	-2.21159000
F	-1.16684700	6.22802400	0.20188900
F	-3.08245500	-3.43489900	-1.12883200
F	-2.09723300	-2.19082800	1.55657800
F	0.95079000	-1.56664400	2.38016400
F	2.18428700	-1.35045100	-1.11667500
F	-0.67963300	-1.94840700	-2.45381000
F	2.94338700	-3.83992300	0.97359400
F	1.97780200	-3.96650600	-2.21159000
F	-0.68235000	-5.56236100	-2.27487200
F	-1.84827500	-5.76264600	1.02936000
F	0.20114700	-4.26939000	2.81582600
F	1.16684700	-6.22802400	0.20188900

E(RB3LYP) = -2892.00895916
Zero-point correction= 0.156515 (Hartree/Particle)
Thermal correction to Energy= 0.201288
Thermal correction to Enthalpy= 0.202232
Thermal correction to Gibbs Free Energy= 0.079472
Sum of electronic and zero-point Energies= -2891.852444
Sum of electronic and thermal Energies= -2891.807671
Sum of electronic and thermal Enthalpies= -2891.806727
Sum of electronic and thermal Free Energies= -2891.929488

	E (Thermal)	CV	S
	KCal/Mol	Cal/Mol-Kelvin	Cal/Mol-Kelvin
Total	126.310	163.236	258.371

	1	2	3
	A(A)	A(B)	A(A)
Frequencies --	5.7167	15.5067	19.3325

Part 3. $[Au(B_{11}F_{11})_2]^{3-}$: η^5,η^5; PG D_{5d}

Au	0.00000000	0.00000000	0.00000000
B	-1.67791800	1.60848400	0.00000000
B	-1.67807700	0.49703800	1.53001000
B	-1.67813800	-1.30158100	0.94576800
B	-1.67813800	-1.30158100	-0.94576800
B	-1.67807700	0.49703800	-1.53001000
B	-3.18167700	-1.52909100	0.00000000
B	-3.18177300	-0.47253400	-1.45414900
B	-3.18173000	1.23680700	-0.89860200
B	-3.18173000	1.23680700	0.89860200
B	-3.18177300	-0.47253400	1.45414900
B	-4.12531500	-0.00012700	0.00000000
B	1.67791800	-1.60848400	0.00000000
B	1.67807700	-0.49703800	-1.53001000
B	1.67813800	1.30158100	-0.94576800
B	1.67813800	1.30158100	0.94576800
B	1.67807700	-0.49703800	1.53001000
B	3.18167700	1.52909100	0.00000000
B	3.18177300	0.47253400	1.45414900
B	3.18173000	-1.23680700	0.89860200
B	3.18173000	-1.23680700	-0.89860200
B	3.18177300	0.47253400	-1.45414900
B	4.12531500	0.00012700	0.00000000
F	-1.25695600	2.92373000	0.00000000
F	-1.25708900	0.90381300	2.78076500
F	-1.25640300	-2.36555400	1.71861300
F	-1.25640300	-2.36555400	-1.71861300
F	-1.25708900	0.90381300	-2.78076500
F	-3.83085800	-2.76654500	0.00000000
F	-3.83100100	-0.85482100	-2.63103300
F	-3.83073000	2.23801600	-1.62597600
F	-3.83073000	2.23801600	1.62597600
F	-3.83100100	-0.85482100	2.63103300
F	-5.51900700	-0.00012900	0.00000000
F	1.25695600	-2.92373000	0.00000000
F	1.25708900	-0.90381300	-2.78076500
F	1.25640300	2.36555400	-1.71861300
F	1.25640300	2.36555400	1.71861300
F	1.25708900	-0.90381300	2.78076500
F	3.83085800	2.76654500	0.00000000
F	3.83100100	0.85482100	2.63103300
F	3.83073000	-2.23801600	1.62597600
F	3.83073000	-2.23801600	-1.62597600
F	3.83100100	0.85482100	-2.63103300
F	5.51900700	0.00012900	0.00000000

E(RB3LYP) = -2880.86725503

Zero-point correction= 0.159362 (Hartree/Particle)
Thermal correction to Energy= 0.201790
Thermal correction to Enthalpy= 0.202734
Thermal correction to Gibbs Free Energy= 0.092325
Sum of electronic and zero-point Energies= -2880.707893
Sum of electronic and thermal Energies= -2880.665465
Sum of electronic and thermal Enthalpies= -2880.664521
Sum of electronic and thermal Free Energies= -2880.774930

	E (Thermal)	CV	S
	KCal/Mol	Cal/Mol-Kelvin	Cal/Mol-Kelvin
Total	126.625	161.494	232.376

	1	2	3
	A(A1U)	A(E1U)	A(E1U)
Frequencies --	32.0240	49.3650	49.7695

Part 3. $[Au(B_{11}F_{11})_2]^{3-}$: η^2,η^2; PG C_2

Au	0.00000000	0.00000000	0.30241100
B	1.65205700	3.09096600	-0.93125200
B	0.18952400	2.38591900	-1.50896700
B	-1.38480200	1.79708300	-0.19781600
B	-0.59849500	1.98680500	1.33535500
B	1.33507900	2.65113500	0.69628400
B	-1.54423000	3.35376700	0.67668300
B	0.07263900	3.67092000	1.41311200
B	1.22334100	4.38167900	0.13892400
B	0.41060000	4.18838500	-1.43506500
B	-1.22283700	3.37523900	-1.09416000
B	-0.55125700	4.65867300	0.01383400
B	-1.65205700	-3.09096600	-0.93125200
B	-1.33507900	-2.65113500	0.69628400
B	0.59849500	-1.98680500	1.33535500
B	1.38480200	-1.79708300	-0.19781600
B	-0.18952400	-2.38591900	-1.50896700
B	1.54423000	-3.35376700	0.67668300
B	1.22283700	-3.37523900	-1.09416000
B	-0.41060000	-4.18838500	-1.43506500
B	-1.22334100	-4.38167900	0.13892400
B	-0.07263900	-3.67092000	1.41311200
B	0.55125700	-4.65867300	0.01383400
F	2.89288300	2.90133900	-1.55539600
F	0.23849000	1.56882400	-2.60683500
F	-2.52705600	1.16618300	-0.66113700
F	-0.83294800	1.56494200	2.63306400
F	2.32004900	2.05788000	1.44602700
F	-2.76007900	3.72040900	1.26180000
F	0.17570400	4.18862300	2.70701600
F	2.04667600	5.43935700	0.53264100
F	0.48098300	5.06465300	-2.52076000
F	-2.25476100	3.63461100	-1.99708900
F	-1.04613400	5.95643800	0.11757300
F	-2.89288300	-2.90133900	-1.55539600
F	-2.32004900	-2.05788000	1.44602700
F	0.83294800	-1.56494200	2.63306400
F	2.52705600	-1.16618300	-0.66113700
F	-0.23849000	-1.56882400	-2.60683500
F	2.76007900	-3.72040900	1.26180000
F	2.25476100	-3.63461100	-1.99708900
F	-0.48098300	-5.06465300	-2.52076000
F	-2.04667600	-5.43935700	0.53264100
F	-0.17570400	-4.18862300	2.70701600
F	1.04613400	-5.95643800	0.11757300

E(RB3LYP) = -2880.80062728

Zero-point correction= 0.156642 (Hartree/Particle)
Thermal correction to Energy= 0.201038
Thermal correction to Enthalpy= 0.201982
Thermal correction to Gibbs Free Energy= 0.081113
Sum of electronic and zero-point Energies= -2880.643985
Sum of electronic and thermal Energies= -2880.599589
Sum of electronic and thermal Enthalpies= -2880.598645
Sum of electronic and thermal Free Energies= -2880.719514

	E (Thermal)	CV	S
	KCal/Mol	Cal/Mol-Kelvin	Cal/Mol-Kelvin
Total	126.153	163.221	254.391

	1	2	3
	A(A)	A(B)	A(A)
Frequencies --	3.5750	19.5070	23.7621

Part 4. $[Cu(B_{11}H_{11})_2]^{3-}$: η^5,η^5; PG D_{5d}

Cu	0.00000000	0.00000000	0.00000000
B	0.48791300	2.11999800	0.00000000
B	1.04368400	1.19175400	1.48881000
B	1.94447000	-0.30953200	0.92057400
B	1.94447000	-0.30953200	-0.92057400
B	1.04368400	1.19175400	-1.48881000
B	3.35846400	0.24240100	0.00000000
B	2.81827700	1.14357600	-1.44584100
B	1.94447000	2.60135600	-0.89364700
B	1.94447000	2.60135600	0.89364700
B	2.81827700	1.14357600	1.44584100
B	3.38213600	2.02907900	0.00000000
B	-0.48791300	-2.11999800	0.00000000
B	-1.04368400	-1.19175400	-1.48881000
B	-1.94447000	0.30953200	-0.92057400
B	-1.94447000	0.30953200	0.92057400
B	-1.04368400	-1.19175400	1.48881000
B	-3.35846400	-0.24240100	0.00000000
B	-2.81827700	-1.14357600	1.44584100
B	-1.94447000	-2.60135600	0.89364700
B	-1.94447000	-2.60135600	-0.89364700
B	-2.81827700	-1.14357600	-1.44584100
B	-3.38213600	-2.02907900	0.00000000
H	-0.47263800	2.83447000	0.00000000
H	0.47508500	1.24847400	2.54053800
H	2.01189700	-1.31137900	1.57195000
H	2.01189700	-1.31137900	-1.57195000
H	0.47508500	1.24847400	-2.54053800
H	4.36917900	-0.41282400	0.00000000
H	3.44564900	1.12994300	-2.47400300
H	1.94961300	3.62405000	-1.52997700
H	1.94961300	3.62405000	1.52997700
H	3.44564900	1.12994300	2.47400300
H	4.41539600	2.64896600	0.00000000
H	0.47263800	-2.83447000	0.00000000
H	-0.47508500	-1.24847400	-2.54053800
H	-2.01189700	1.31137900	-1.57195000
H	-2.01189700	1.31137900	1.57195000
H	-0.47508500	-1.24847400	2.54053800
H	-4.36917900	0.41282400	0.00000000
H	-3.44564900	-1.12994300	2.47400300
H	-1.94961300	-3.62405000	1.52997700
H	-1.94961300	-3.62405000	-1.52997700
H	-3.44564900	-1.12994300	-2.47400300
H	-4.41539600	-2.64896600	0.00000000

E(RB3LYP) = -758.106055157

Zero-point correction= 0.303111 (Hartree/Particle)
Thermal correction to Energy= 0.321233
Thermal correction to Enthalpy= 0.322177
Thermal correction to Gibbs Free Energy= 0.262898
Sum of electronic and zero-point Energies= -757.802944
Sum of electronic and thermal Energies= -757.784822
Sum of electronic and thermal Enthalpies= -757.783878
Sum of electronic and thermal Free Energies= -757.843157

	E (Thermal)	CV	S
	KCal/Mol	Cal/Mol-Kelvin	Cal/Mol-Kelvin
Total	201.577	94.582	124.763

	1	2	3
	AU(A1U)	AU(E1U)	BU(E1U)
Frequencies --	69.1093	90.8618	96.5582

Part 4. $[Cu(B_{11}H_{11})_2]^{3-}$: η^2,η^2; PG C_2

Cu	0.00000000	0.00000000	0.45186100
B	0.54386400	4.15386700	-1.36843300
B	1.16178700	2.58853800	-1.40996700
B	1.59746400	1.32491600	-0.10318200
B	0.61930400	1.98620300	1.08332100
B	-0.01604800	3.39056000	0.02321800
B	2.37539200	2.30350100	1.17118700
B	1.15378300	3.58940200	1.42369800
B	1.20796600	4.80815100	0.07814700
B	2.25481100	4.09665000	-1.19613900
B	2.78901900	2.47866100	-0.56461300
B	2.69119800	3.92034400	0.52673900
B	-0.54386300	-4.15386700	-1.36843300
B	0.01604800	-3.39055900	0.02321800
B	-0.61930500	-1.98620300	1.08332100
B	-1.59746400	-1.32491600	-0.10318200
B	-1.16178700	-2.58853800	-1.40996700
B	-2.37539300	-2.30350100	1.17118700
B	-2.78901900	-2.47866200	-0.56461300
B	-2.25481000	-4.09665100	-1.19613900
B	-1.20796600	-4.80815100	0.07814700
B	-1.15378300	-3.58940200	1.42369800
B	-2.69119800	-3.92034500	0.52673900
H	-0.13290600	4.67106300	-2.21408700
H	0.84950100	1.95763300	-2.38744100
H	1.82390200	0.17109100	-0.43432600
H	-0.11616400	1.48729200	1.91723700
H	-1.21575200	3.36181400	0.12869800
H	3.08405300	1.82758200	2.01885400
H	0.84979700	4.05538600	2.49079100
H	1.07330200	5.96536500	0.38514400
H	3.00030700	4.65483300	-1.96032000
H	3.78988300	2.05951900	-1.08503400
H	3.63927500	4.51094600	0.97511700
H	0.13290600	-4.67106300	-2.21408800
H	1.21575200	-3.36181300	0.12869800
H	0.11616400	-1.48729100	1.91723700
H	-1.82390300	-0.17109100	-0.43432600
H	-0.84950200	-1.95763300	-2.38744100
H	-3.08405400	-1.82758200	2.01885400
H	-3.78988300	-2.05951900	-1.08503500
H	-3.00030700	-4.65483400	-1.96032000
H	-1.07330100	-5.96536500	0.38514500
H	-0.84979700	-4.05538600	2.49079100
H	-3.63927500	-4.51094700	0.97511700

E(RB3LYP) = -758.076162513

Zero-point correction= 0.296726 (Hartree/Particle)
Thermal correction to Energy= 0.317864
Thermal correction to Enthalpy= 0.318808
Thermal correction to Gibbs Free Energy= 0.247057
Sum of electronic and zero-point Energies= -757.779436
Sum of electronic and thermal Energies= -757.758299
Sum of electronic and thermal Enthalpies= -757.757355
Sum of electronic and thermal Free Energies= -757.829106

	E (Thermal)	CV	S
	KCal/Mol	Cal/Mol-Kelvin	Cal/Mol-Kelvin
Total	199.462	97.808	151.013

	1	2	3
	A(A)	A(A)	A(B)
Frequencies --	19.1515	21.3346	25.0024

Part 4. $[Cu(B_{11}H_{11})_2]^{3-}$: η^2,η^2; PG C_{2h}

Cu	0.00000000	0.00000000	0.00000000
B	-1.80689900	3.55378800	0.00000000
B	-0.85201400	2.62600300	1.03462900
B	0.85201400	1.80200400	0.84708900
B	0.85201400	1.80200400	-0.84708900
B	-0.85201400	2.62600300	-1.03462900
B	1.78493500	3.05412400	0.00000000
B	0.73088400	3.44157500	-1.39868500
B	-0.66484100	4.48301600	-0.89648600
B	-0.66484100	4.48301600	0.89648600
B	0.73088400	3.44157500	1.39868500
B	0.87270100	4.57714900	0.00000000
B	1.80689900	-3.55378800	0.00000000
B	0.85201400	-2.62600300	1.03462900
B	-0.85201400	-1.80200400	0.84708900
B	-0.85201400	-1.80200400	-0.84708900
B	0.85201400	-2.62600300	-1.03462900
B	-1.78493500	-3.05412400	0.00000000
B	-0.73088400	-3.44157500	-1.39868500
B	0.66484100	-4.48301600	-0.89648600
B	0.66484100	-4.48301600	0.89648600
B	-0.73088400	-3.44157500	1.39868500
B	-0.87270100	-4.57714900	0.00000000
H	-3.00650200	3.59606500	0.00000000
H	-1.48503300	1.95121200	1.80338100
H	1.24457700	1.01948000	1.67957800
H	1.24457700	1.01948000	-1.67957800
H	-1.48503300	1.95121200	-1.80338100
H	2.98791000	3.07712000	0.00000000
H	1.12905500	3.65096400	-2.51488600
H	-0.99342800	5.36371700	-1.64956200
H	-0.99342800	5.36371700	1.64956200
H	1.12905500	3.65096400	2.51488600
H	1.52027100	5.59171000	0.00000000
H	3.00650200	-3.59606500	0.00000000
H	1.48503300	-1.95121200	1.80338100
H	-1.24457700	-1.01948000	1.67957800
H	-1.24457700	-1.01948000	-1.67957800
H	1.48503300	-1.95121200	-1.80338100
H	-2.98791000	-3.07712000	0.00000000
H	-1.12905500	-3.65096400	-2.51488600
H	0.99342800	-5.36371700	-1.64956200
H	0.99342800	-5.36371700	1.64956200
H	-1.12905500	-3.65096400	2.51488600
H	-1.52027100	-5.59171000	0.00000000

E(RB3LYP) = -758.074470462

Zero-point correction= 0.297002 (Hartree/Particle)
Thermal correction to Energy= 0.316213
Thermal correction to Enthalpy= 0.317158
Thermal correction to Gibbs Free Energy= 0.253060
Sum of electronic and zero-point Energies= -757.777469
Sum of electronic and thermal Energies= -757.758257
Sum of electronic and thermal Enthalpies= -757.757313
Sum of electronic and thermal Free Energies= -757.821411

	E (Thermal)	CV	S
	KCal/Mol	Cal/Mol-Kelvin	Cal/Mol-Kelvin
Total	198.427	94.268	134.906

	1	2	3
	BU	AU	AG
Frequencies --	-32.5595	-11.8750	37.5424

Part 4. $[Ag(B_{11}H_{11})_2]^{3-}$: η[5],η[5]; PG D_{5d}

Ag	0.00000000	0.00000000	0.00000000
B	0.00000000	1.57859600	-1.67531000
B	1.50189700	0.48765000	-1.67667200
B	0.92813300	-1.27785800	-1.67645700
B	-0.92813300	-1.27785800	-1.67645700
B	-1.50189700	0.48765000	-1.67667200
B	0.00000000	-1.52239500	-3.16790000
B	-1.44798600	-0.47050500	-3.16804300
B	-0.89469500	1.23186000	-3.16762800
B	0.89469500	1.23186000	-3.16762800
B	1.44798600	-0.47050500	-3.16804300
B	0.00000000	0.00006900	-4.10274600
B	0.00000000	-1.57859600	1.67531000
B	-1.50189700	-0.48765000	1.67667200
B	-0.92813300	1.27785800	1.67645700
B	0.92813300	1.27785800	1.67645700
B	1.50189700	-0.48765000	1.67667200
B	0.00000000	1.52239500	3.16790000
B	1.44798600	0.47050500	3.16804300
B	0.89469500	-1.23186000	3.16762800
B	-0.89469500	-1.23186000	3.16762800
B	-1.44798600	0.47050500	3.16804300
B	0.00000000	-0.00006900	4.10274600
H	0.00000000	2.68102100	-1.20428300
H	2.55224300	0.82918600	-1.21058200
H	1.57544400	-2.17237500	-1.20944600
H	-1.57544400	-2.17237400	-1.20944600
H	-2.55224300	0.82918600	-1.21058200
H	0.00000000	-2.59847500	-3.70870700
H	-2.47153300	-0.80348600	-3.70828200
H	-1.52732500	2.10262900	-3.70787700
H	1.52732500	2.10262900	-3.70787700
H	2.47153300	-0.80348600	-3.70828100
H	0.00000000	0.00026700	-5.30672100
H	0.00000000	-2.68102100	1.20428300
H	-2.55224300	-0.82918600	1.21058200
H	-1.57544400	2.17237500	1.20944600
H	1.57544400	2.17237400	1.20944600
H	2.55224300	-0.82918600	1.21058200
H	0.00000000	2.59847500	3.70870700
H	2.47153300	0.80348600	3.70828200
H	1.52732500	-2.10262900	3.70787700
H	-1.52732500	-2.10262900	3.70787700
H	-2.47153300	0.80348600	3.70828100
H	0.00000000	-0.00026700	5.30672100

E(RB3LYP) = -707.712297452

Zero-point correction= 0.301286 (Hartree/Particle)
Thermal correction to Energy= 0.319991
Thermal correction to Enthalpy= 0.320936
Thermal correction to Gibbs Free Energy= 0.258976
Sum of electronic and zero-point Energies= -707.411012
Sum of electronic and thermal Energies= -707.392306
Sum of electronic and thermal Enthalpies= -707.391362
Sum of electronic and thermal Free Energies= -707.453322

	E (Thermal)	CV	S
	KCal/Mol	Cal/Mol-Kelvin	Cal/Mol-Kelvin
Total	200.798	95.752	130.405

	1	2	3
	AU(A1U)	AU(E1U)	AU(E1U)
Frequencies --	37.0056	75.4451	89.6182

Part 4. $[Ag(B_{11}H_{11})_2]^{3-}$: η²,η²; PG C_2

Ag	0.00000000	0.00000000	0.39226300
B	-1.43255800	3.64494500	-0.94090900
B	-0.17493600	2.59405200	-1.33377800
B	1.28760600	1.87971500	-0.35187700
B	0.61793400	2.16931500	1.17801000
B	-0.99001000	2.93986800	0.52486900
B	1.84732600	3.29402600	0.56537200
B	0.33804900	3.87354800	1.34448500
B	-0.71141000	4.75816200	0.16039800
B	0.00000000	4.45446900	-1.45745500
B	1.44666800	3.39827600	-1.17986800
B	1.05578700	4.75959000	-0.05652800
B	1.43255800	-3.64494500	-0.94090900
B	0.99001000	-2.93986800	0.52486900
B	-0.61793400	-2.16931500	1.17801000
B	-1.28760600	-1.87971500	-0.35187700
B	0.17493600	-2.59405200	-1.33377800
B	-1.84732600	-3.29402600	0.56537200
B	-1.44666800	-3.39827600	-1.17986800
B	0.00000000	-4.45446900	-1.45745500
B	0.71141000	-4.75816200	0.16039800
B	-0.33804900	-3.87354800	1.34448500
B	-1.05578700	-4.75959000	-0.05652800
H	-2.53232300	3.63754000	-1.42232100
H	-0.47358100	1.77127900	-2.16081300
H	1.96802900	0.99823900	-0.81932100
H	0.63393900	1.57792900	2.23126600
H	-1.89753700	2.37300900	1.07914500
H	2.95269500	3.36712900	1.03629500
H	0.26481200	4.28789800	2.47217300
H	-1.28533000	5.73990600	0.55855200
H	0.02201000	5.18147000	-2.41796100
H	2.25960200	3.43322200	-2.06687500
H	1.68490400	5.78221500	0.02808100
H	2.53232300	-3.63754000	-1.42232100
H	1.89753700	-2.37300900	1.07914500
H	-0.63393900	-1.57792900	2.23126600
H	-1.96802900	-0.99823900	-0.81932100
H	0.47358100	-1.77127900	-2.16081300
H	-2.95269500	-3.36712900	1.03629500
H	-2.25960200	-3.43322200	-2.06687500
H	-0.02201000	-5.18147000	-2.41796100
H	1.28533000	-5.73990600	0.55855200
H	-0.26481200	-4.28789800	2.47217300
H	-1.68490400	-5.78221500	0.02808100

E(RB3LYP) = -707.701837017

Zero-point correction=	0.297262 (Hartree/Particle)
Thermal correction to Energy=	0.318351
Thermal correction to Enthalpy=	0.319295
Thermal correction to Gibbs Free Energy=	0.247989
Sum of electronic and zero-point Energies=	-707.404575
Sum of electronic and thermal Energies=	-707.383486
Sum of electronic and thermal Enthalpies=	-707.382542
Sum of electronic and thermal Free Energies=	-707.453848

	E (Thermal)	CV	S
	KCal/Mol	Cal/Mol-Kelvin	Cal/Mol-Kelvin
Total	199.768	97.973	150.075

	1	2	3
	A	B	A
Frequencies --	16.8088	27.4841	27.4927

Part 4. $[Ag(B_{11}H_{11})_2]^{3-}$: η^2,η^2; PG C_{2h}

Ag	0.00000000	0.00000000	0.00000000
B	-1.82377300	3.70891200	0.00000000
B	-0.83333400	2.81416100	1.02955600
B	0.89844400	2.05270100	0.84779900
B	0.89844400	2.05270100	-0.84779900
B	-0.83333400	2.81416100	-1.02955600
B	1.78329800	3.33738700	0.00000000
B	0.71602800	3.68883100	-1.39871000
B	-0.71602800	4.67978600	-0.89659400
B	-0.71602800	4.67978600	0.89659400
B	0.71602800	3.68883100	1.39871000
B	0.81485100	4.82859500	0.00000000
B	1.82377300	-3.70891200	0.00000000
B	0.83333400	-2.81416100	1.02955600
B	-0.89844400	-2.05270100	0.84779900
B	-0.89844400	-2.05270100	-0.84779900
B	0.83333400	-2.81416100	-1.02955600
B	-1.78329800	-3.33738700	0.00000000
B	-0.71602800	-3.68883100	-1.39871000
B	0.71602800	-4.67978600	-0.89659400
B	0.71602800	-4.67978600	0.89659400
B	-0.71602800	-3.68883100	1.39871000
B	-0.81485100	-4.82859500	0.00000000
H	-3.02412200	3.70632000	0.00000000
H	-1.44381600	2.11287100	1.79551100
H	1.33259900	1.30429100	1.69013800
H	1.33259900	1.30429100	-1.69013800
H	-1.44381600	2.11287100	-1.79551100
H	2.98493700	3.40764400	0.00000000
H	1.10499200	3.91453300	-2.51521900
H	-1.07760000	5.54819600	-1.64938200
H	-1.07760000	5.54819600	1.64938200
H	1.10499200	3.91453300	2.51521900
H	1.42954500	5.86345200	0.00000000
H	3.02412200	-3.70632000	0.00000000
H	1.44381600	-2.11287100	1.79551100
H	-1.33259900	-1.30429100	1.69013800
H	-1.33259900	-1.30429100	-1.69013800
H	1.44381600	-2.11287100	-1.79551100
H	-2.98493700	-3.40764400	0.00000000
H	-1.10499200	-3.91453300	-2.51521900
H	1.07760000	-5.54819600	-1.64938200
H	1.07760000	-5.54819600	1.64938200
H	-1.10499200	-3.91453300	2.51521900
H	-1.42954500	-5.86345200	0.00000000

E(RB3LYP) = -707.701489603

Zero-point correction= 0.296867 (Hartree/Particle)
Thermal correction to Energy= 0.317134
Thermal correction to Enthalpy= 0.318078
Thermal correction to Gibbs Free Energy= 0.249704
Sum of electronic and zero-point Energies= -707.404622
Sum of electronic and thermal Energies= -707.384355
Sum of electronic and thermal Enthalpies= -707.383411
Sum of electronic and thermal Free Energies= -707.451785

	E (Thermal)	CV	S
	KCal/Mol	Cal/Mol-Kelvin	Cal/Mol-Kelvin
Total	199.005	96.201	143.905

	1	2	3
	AU	BU	AU
Frequencies --	-19.7904	23.8574	27.2955

Part 4. $[Au(B_{11}H_{11})_2]^{3-}$: η^5,η^5; PG D_{5d}

Au	0.00000000	0.00000000	0.00000000
B	0.00000000	1.58619700	-1.65680100
B	1.50892500	0.49003000	-1.65815700
B	0.93251700	-1.28372200	-1.65790500
B	-0.93251600	-1.28372200	-1.65790500
B	-1.50892500	0.49003000	-1.65815700
B	0.00000000	-1.52050000	-3.14875300
B	-1.44622100	-0.46986800	-3.14894700
B	-0.89361300	1.23040500	-3.14856500
B	0.89361300	1.23040500	-3.14856500
B	1.44622100	-0.46986800	-3.14894700
B	0.00000000	0.00008500	-4.08354700
B	0.00000000	-1.58619700	1.65680100
B	-1.50892500	-0.49003000	1.65815700
B	-0.93251700	1.28372200	1.65790500
B	0.93251600	1.28372200	1.65790500
B	1.50892500	-0.49003000	1.65815700
B	0.00000000	1.52050000	3.14875300
B	1.44622100	0.46986800	3.14894700
B	0.89361300	-1.23040500	3.14856500
B	-0.89361300	-1.23040500	3.14856500
B	-1.44622100	0.46986800	3.14894700
B	0.00000000	-0.00008500	4.08354700
H	0.00000000	2.69567000	-1.20315900
H	2.56556200	0.83358800	-1.20856900
H	1.58371700	-2.18357200	-1.20726800
H	-1.58371700	-2.18357200	-1.20726800
H	-2.56556200	0.83358800	-1.20856900
H	0.00000000	-2.59474200	-3.69300200
H	-2.46801500	-0.80229300	-3.69262600
H	-1.52515900	2.09965100	-3.69232600
H	1.52515900	2.09965100	-3.69232600
H	2.46801500	-0.80229200	-3.69262600
H	0.00000000	0.00025400	-5.28777600
H	0.00000000	-2.69567000	1.20315900
H	-2.56556200	-0.83358800	1.20856900
H	-1.58371700	2.18357200	1.20726800
H	1.58371700	2.18357200	1.20726800
H	2.56556200	-0.83358800	1.20856900
H	0.00000000	2.59474200	3.69300200
H	2.46801500	0.80229300	3.69262600
H	1.52515900	-2.09965100	3.69232600
H	-1.52515900	-2.09965100	3.69232600
H	-2.46801500	0.80229200	3.69262600
H	0.00000000	-0.00025400	5.28777600

E(RB3LYP) = -696.555704742
Zero-point correction= 0.302817 (Hartree/Particle)
Thermal correction to Energy= 0.321175
Thermal correction to Enthalpy= 0.322119
Thermal correction to Gibbs Free Energy= 0.260589
Sum of electronic and zero-point Energies= -696.252887
Sum of electronic and thermal Energies= -696.234530
Sum of electronic and thermal Enthalpies= -696.233586
Sum of electronic and thermal Free Energies= -696.295116

	E (Thermal)	CV	S
	KCal/Mol	Cal/Mol-Kelvin	Cal/Mol-Kelvin
Total	201.540	94.655	129.502

	1	2	3
	AU(A1U)	AU(E1U)	AU(E1U)
Frequencies --	42.0927	78.7975	86.7806

Part 4. $[Au(B_{11}H_{11})_2]^{3-}$: η^2,η^2; PG C_2

Au	0.00000000	0.00000000	0.38955100
B	-0.78059300	3.53544600	-1.17344600
B	0.36795000	2.32574700	-1.44653600
B	1.66795200	1.47085800	-0.27802500
B	0.92173800	1.95625500	1.20580000
B	-0.55452000	2.92017100	0.38352600
B	2.30558800	2.88412000	0.59047500
B	0.83673100	3.70512100	1.21340200
B	0.00000000	4.63237400	-0.09276300
B	0.77446600	4.13227600	-1.62962500
B	2.04234300	2.92630300	-1.18224900
B	1.76216100	4.39306500	-0.17206800
B	0.78059300	-3.53544600	-1.17344600
B	0.55452000	-2.92017100	0.38352600
B	-0.92173800	-1.95625500	1.20580000
B	-1.66795200	-1.47085800	-0.27802500
B	-0.36795000	-2.32574700	-1.44653600
B	-2.30558800	-2.88412000	0.59047500
B	-2.04234300	-2.92630300	-1.18224900
B	-0.77446600	-4.13227600	-1.62962500
B	0.00000000	-4.63237400	-0.09276300
B	-0.83673100	-3.70512100	1.21340200
B	-1.76216100	-4.39306500	-0.17206800
H	-1.83537700	3.63584900	-1.73771600
H	0.02897300	1.51907600	-2.27052300
H	2.35286800	0.55080300	-0.64615800
H	0.85405900	1.51951100	2.32699400
H	-1.57051700	2.54585000	0.90767900
H	3.37746200	2.86046600	1.13707000
H	0.75953100	4.19023500	2.31117000
H	-0.46795600	5.70290400	0.19851400
H	0.95263100	4.78500000	-2.62572100
H	2.92533300	2.79141100	-1.98770200
H	2.51876100	5.32494900	-0.09334400
H	1.83537700	-3.63584900	-1.73771600
H	1.57051700	-2.54585000	0.90767900
H	-0.85405900	-1.51951100	2.32699400
H	-2.35286800	-0.55080300	-0.64615800
H	-0.02897300	-1.51907600	-2.27052300
H	-3.37746200	-2.86046600	1.13707000
H	-2.92533300	-2.79141100	-1.98770200
H	-0.95263100	-4.78500000	-2.62572100
H	0.46795600	-5.70290400	0.19851400
H	-0.75953100	-4.19023500	2.31117000
H	-2.51876100	-5.32494900	-0.09334400

E(RB3LYP) = -696.486345812

Zero-point correction= 0.297934 (Hartree/Particle)
Thermal correction to Energy= 0.318610
Thermal correction to Enthalpy= 0.319554
Thermal correction to Gibbs Free Energy= 0.250079
Sum of electronic and zero-point Energies= -696.188411
Sum of electronic and thermal Energies= -696.167736
Sum of electronic and thermal Enthalpies= -696.166792
Sum of electronic and thermal Free Energies= -696.236267

	E (Thermal)	CV	S
	KCal/Mol	Cal/Mol-Kelvin	Cal/Mol-Kelvin
Total	199.931	97.916	146.222

	1	2	3
	A	B	A
Frequencies --	13.9255	32.2295	35.2338

Part 4. $[Au(B_{11}H_{11})_2]^{3-}$: η^2,η^2; PG C_{2h}

Au	0.00000000	0.00000000	0.00000000
B	-1.80379900	3.52934600	0.00000000
B	-0.80088300	2.68233300	1.06329000
B	0.97840800	1.92900300	0.86372700
B	0.97840800	1.92900300	-0.86372700
B	-0.80088300	2.68233300	-1.06329000
B	1.80023500	3.24393500	0.00000000
B	0.72368200	3.57799700	-1.39605800
B	-0.72368200	4.53646800	-0.89590000
B	-0.72368200	4.53646800	0.89590000
B	0.72368200	3.57799700	1.39605800
B	0.80369100	4.71541000	0.00000000
B	1.80379900	-3.52934600	0.00000000
B	0.80088300	-2.68233300	1.06329000
B	-0.97840800	-1.92900300	0.86372700
B	-0.97840800	-1.92900300	-0.86372700
B	0.80088300	-2.68233300	-1.06329000
B	-1.80023500	-3.24393500	0.00000000
B	-0.72368200	-3.57799700	-1.39605800
B	0.72368200	-4.53646800	-0.89590000
B	0.72368200	-4.53646800	0.89590000
B	-0.72368200	-3.57799700	1.39605800
B	-0.80369100	-4.71541000	0.00000000
H	-3.00373600	3.50028900	0.00000000
H	-1.40136500	1.99431800	1.84591900
H	1.49329300	1.27082200	1.73129400
H	1.49329300	1.27082200	-1.73129400
H	-1.40136500	1.99431800	-1.84591900
H	2.99857300	3.35404500	0.00000000
H	1.12443400	3.80421000	-2.50731600
H	-1.10788800	5.39642700	-1.64600800
H	-1.10788800	5.39642700	1.64600800
H	1.12443400	3.80421000	2.50731600
H	1.40532000	5.75710500	0.00000000
H	3.00373600	-3.50028900	0.00000000
H	1.40136500	-1.99431800	1.84591900
H	-1.49329300	-1.27082200	1.73129400
H	-1.49329300	-1.27082200	-1.73129400
H	1.40136500	-1.99431800	-1.84591900
H	-2.99857300	-3.35404500	0.00000000
H	-1.12443400	-3.80421000	-2.50731600
H	1.10788800	-5.39642700	-1.64600800
H	1.10788800	-5.39642700	1.64600800
H	-1.12443400	-3.80421000	2.50731600
H	-1.40532000	-5.75710500	0.00000000

E(RB3LYP) = -696.484865744

Zero-point correction= 0.297676 (Hartree/Particle)
Thermal correction to Energy= 0.317531
Thermal correction to Enthalpy= 0.318476
Thermal correction to Gibbs Free Energy= 0.252066
Sum of electronic and zero-point Energies= -696.187189
Sum of electronic and thermal Energies= -696.167334
Sum of electronic and thermal Enthalpies= -696.166390
Sum of electronic and thermal Free Energies= -696.232800

	E (Thermal)	CV	S
	KCal/Mol	Cal/Mol-Kelvin	Cal/Mol-Kelvin
Total	199.254	96.075	139.772

	1	2	3
	AU	BU	AU
Frequencies --	-12.7695	28.3934	41.5529

Part 4. $[Cu(B_{11}F_{11})_2]^{3-}$: η^5,η^5; PG D_{5d}

Cu	0.00000000	0.00000000	0.00000000
B	-1.51152100	1.58068600	0.00000000
B	-1.51064800	0.48933000	-1.50245300
B	-1.51014700	-1.27766500	-0.92924600
B	-1.51014700	-1.27766500	0.92924600
B	-1.51064800	0.48933000	1.50245300
B	-3.01998900	-1.52530100	0.00000000
B	-3.02059800	-0.47096100	1.45075100
B	-3.02138200	1.23415100	0.89653500
B	-3.02138200	1.23415100	-0.89653500
B	-3.02059800	-0.47096100	-1.45075100
B	-3.97429700	-0.00038300	0.00000000
B	1.51152100	-1.58068600	0.00000000
B	1.51064800	-0.48933000	1.50245300
B	1.51014700	1.27766500	0.92924600
B	1.51014700	1.27766500	-0.92924600
B	1.51064800	-0.48933000	-1.50245300
B	3.01998900	1.52530100	0.00000000
B	3.02059800	0.47096100	-1.45075100
B	3.02138200	-1.23415100	-0.89653500
B	3.02138200	-1.23415100	0.89653500
B	3.02059800	0.47096100	1.45075100
B	3.97429700	0.00038300	0.00000000
F	-1.10900000	2.90430300	0.00000000
F	-1.10575200	0.90008400	-2.75996000
F	-1.10587600	-2.34733300	-1.70771300
F	-1.10587600	-2.34733300	1.70771300
F	-1.10575200	0.90008400	2.75996000
F	-3.63855700	-2.77070900	0.00000000
F	-3.63956900	-0.85565100	2.63500600
F	-3.63995600	2.24144000	1.62883300
F	-3.63995600	2.24144000	-1.62883300
F	-3.63956900	-0.85565100	-2.63500600
F	-5.35521200	-0.00107400	0.00000000
F	1.10900000	-2.90430300	0.00000000
F	1.10575200	-0.90008400	2.75996000
F	1.10587600	2.34733400	1.70771300
F	1.10587600	2.34733300	-1.70771300
F	1.10575200	-0.90008400	-2.75996000
F	3.63855700	2.77070900	0.00000000
F	3.63956900	0.85565100	-2.63500600
F	3.63995600	-2.24144000	-1.62883300
F	3.63995600	-2.24144000	1.62883300
F	3.63956900	0.85565100	2.63500600
F	5.35521200	0.00107400	0.00000000

E(RB3LYP) = -2942.86254809

Zero-point correction= 0.160200 (Hartree/Particle)
Thermal correction to Energy= 0.201932
Thermal correction to Enthalpy= 0.202876
Thermal correction to Gibbs Free Energy= 0.094552
Sum of electronic and zero-point Energies= -2942.702348
Sum of electronic and thermal Energies= -2942.660616
Sum of electronic and thermal Enthalpies= -2942.659672
Sum of electronic and thermal Free Energies= -2942.767996

	E (Thermal)	CV	S
	KCal/Mol	Cal/Mol-Kelvin	Cal/Mol-Kelvin
Total	126.714	160.770	227.988

	1	2	3
	A(A1U)	A(E1U)	A(E1U)
Frequencies --	55.2862	55.9143	59.1352

Part 4. $[Cu(B_{11}F_{11})_2]^{3-}$: η^2,η^2; PG C_2

Cu	0.00000000	0.00000000	0.14771300
B	1.63298300	2.80557700	-0.74432900
B	0.20824700	2.14313000	-1.43763300
B	-1.42501900	1.57759600	-0.29778800
B	-0.78763800	1.70026900	1.26589400
B	1.15024900	2.29717300	0.82261500
B	-1.67974000	3.10272600	0.60680200
B	-0.13509900	3.35273100	1.47996800
B	1.14614000	4.06304800	0.32842300
B	0.46080900	3.94219100	-1.30793800
B	-1.21769100	3.16342900	-1.11977500
B	-0.60074400	4.40294900	0.06790100
B	-1.63298300	-2.80557700	-0.74432900
B	-1.15024900	-2.29717300	0.82261400
B	0.78763700	-1.70026900	1.26589400
B	1.42501900	-1.57759600	-0.29778800
B	-0.20824700	-2.14313000	-1.43763300
B	1.67974000	-3.10272500	0.60680200
B	1.21769100	-3.16342800	-1.11977500
B	-0.46080900	-3.94219100	-1.30793800
B	-1.14614000	-4.06304800	0.32842300
B	0.13509900	-3.35273100	1.47996700
B	0.60074400	-4.40294900	0.06790100
F	2.91205500	2.58556000	-1.26687300
F	0.33049400	1.36053500	-2.56076300
F	-2.46892900	0.86850700	-0.88498800
F	-1.08585400	1.14948100	2.50340700
F	2.06279800	1.65209800	1.62345900
F	-2.93631300	3.45577200	1.10055400
F	-0.10444700	3.81044700	2.79528800
F	1.96068900	5.06514000	0.84242700
F	0.62957100	4.82713200	-2.36740200
F	-2.15568000	3.44932200	-2.10856300
F	-1.06460300	5.70664100	0.16955200
F	-2.91205500	-2.58556000	-1.26687300
F	-2.06279800	-1.65209800	1.62345900
F	1.08585400	-1.14948100	2.50340700
F	2.46892900	-0.86850700	-0.88498800
F	-0.33049500	-1.36053500	-2.56076300
F	2.93631300	-3.45577200	1.10055300
F	2.15568000	-3.44932200	-2.10856300
F	-0.62957100	-4.82713200	-2.36740200
F	-1.96068900	-5.06514000	0.84242700
F	0.10444800	-3.81044600	2.79528800
F	1.06460300	-5.70664100	0.16955200

E(RB3LYP) = -2942.79617247

Zero-point correction= 0.157052 (Hartree/Particle)
Thermal correction to Energy= 0.201301
Thermal correction to Enthalpy= 0.202245
Thermal correction to Gibbs Free Energy= 0.083412
Sum of electronic and zero-point Energies= -2942.639120
Sum of electronic and thermal Energies= -2942.594872
Sum of electronic and thermal Enthalpies= -2942.593927
Sum of electronic and thermal Free Energies= -2942.712761

	E (Thermal)	CV	S
	KCal/Mol	Cal/Mol-Kelvin	Cal/Mol-Kelvin
Total	126.318	162.901	250.106

	1	2	3
	A(A)	A(B)	A(A)
Frequencies --	20.2626	21.7174	33.9640

Part 4. $[Ag(B_{11}F_{11})_2]^{3-}$: η^5,η^5; PG D_{5d}

Ag	0.00000000	0.00000000	0.00000000
B	-1.68423000	1.59486300	0.00000000
B	-1.68363300	0.49295900	1.51603200
B	-1.68371900	-1.28896800	0.93729500
B	-1.68371900	-1.28896800	-0.93729500
B	-1.68363300	0.49295900	-1.51603200
B	-3.18747100	-1.52832900	0.00000000
B	-3.18794000	-0.47198100	-1.45344700
B	-3.18802500	1.23650800	-0.89837900
B	-3.18802500	1.23650800	0.89837900
B	-3.18794000	-0.47198100	1.45344700
B	-4.13694500	-0.00012900	0.00000000
B	1.68423000	-1.59486300	0.00000000
B	1.68363300	-0.49295900	-1.51603200
B	1.68371900	1.28896800	-0.93729500
B	1.68371900	1.28896800	0.93729500
B	1.68363300	-0.49295900	1.51603200
B	3.18747100	1.52832900	0.00000000
B	3.18794000	0.47198100	1.45344700
B	3.18802500	-1.23650800	0.89837900
B	3.18802500	-1.23650800	-0.89837900
B	3.18794000	0.47198100	-1.45344700
B	4.13694500	0.00012900	0.00000000
F	-1.25640900	2.91175500	0.00000000
F	-1.25202700	0.89900500	2.76739300
F	-1.25188300	-2.35243400	1.71145700
F	-1.25188300	-2.35243400	-1.71145700
F	-1.25202700	0.89900500	-2.76739300
F	-3.81621200	-2.76924200	0.00000000
F	-3.81710800	-0.85535500	-2.63337700
F	-3.81680000	2.24016300	-1.62803100
F	-3.81680000	2.24016300	1.62803100
F	-3.81710800	-0.85535500	2.63337700
F	-5.51953200	-0.00042900	0.00000000
F	1.25640900	-2.91175500	0.00000000
F	1.25202700	-0.89900500	-2.76739300
F	1.25188300	2.35243400	-1.71145700
F	1.25188300	2.35243400	1.71145700
F	1.25202700	-0.89900500	2.76739300
F	3.81621200	2.76924200	0.00000000
F	3.81710800	0.85535500	2.63337700
F	3.81680000	-2.24016300	1.62803100
F	3.81680000	-2.24016300	-1.62803100
F	3.81710800	0.85535500	-2.63337700
F	5.51953200	0.00042900	0.00000000

E(RB3LYP) = -2892.46112938
Zero-point correction= 0.158511 (Hartree/Particle)
Thermal correction to Energy= 0.201185
Thermal correction to Enthalpy= 0.202130
Thermal correction to Gibbs Free Energy= 0.090367
Sum of electronic and zero-point Energies= -2892.302619
Sum of electronic and thermal Energies= -2892.259944
Sum of electronic and thermal Enthalpies= -2892.259000
Sum of electronic and thermal Free Energies= -2892.370762

	E (Thermal)	CV	S
	KCal/Mol	Cal/Mol-Kelvin	Cal/Mol-Kelvin
Total	126.246	161.850	235.223

	1	2	3
	A(A1U)	A(E1U)	A(E1U)
Frequencies --	29.4558	47.3047	47.8732

Part 4. $[Ag(B_{11}F_{11})_2]^{3-}$: η^2,η^2; PG C_2

Ag	0.00000000	0.00000000	0.19136600
B	1.68334800	3.21123800	-0.70829900
B	0.28934400	2.50412100	-1.40171200
B	-1.37784300	1.92325300	-0.33850100
B	-0.78333800	2.04468500	1.22983300
B	1.14505100	2.66992500	0.82058300
B	-1.67596800	3.44575700	0.56141500
B	-0.15435600	3.70475000	1.47021600
B	1.14804700	4.44334600	0.36391900
B	0.50703500	4.31664000	-1.30212500
B	-1.16092900	3.50840700	-1.15078500
B	-0.59595200	4.75613800	0.05033200
B	-1.68334800	-3.21123800	-0.70829900
B	-1.14505100	-2.66992500	0.82058300
B	0.78333800	-2.04468500	1.22983300
B	1.37784300	-1.92325300	-0.33850100
B	-0.28934400	-2.50412100	-1.40171200
B	1.67596800	-3.44575700	0.56141500
B	1.16092900	-3.50840700	-1.15078500
B	-0.50703500	-4.31664000	-1.30212500
B	-1.14804700	-4.44334600	0.36391900
B	0.15435600	-3.70475000	1.47021600
B	0.59595200	-4.75613800	0.05033200
F	2.97644000	2.97710200	-1.18990800
F	0.46279600	1.66998900	-2.48664100
F	-2.39252500	1.21212700	-0.97435300
F	-1.08531600	1.47873500	2.46482700
F	2.03581600	1.97590900	1.61272500
F	-2.94914500	3.78596400	1.02236500
F	-0.16052100	4.14915800	2.79209500
F	1.92386600	5.46398400	0.90409200
F	0.67628500	5.21624000	-2.35013600
F	-2.07027800	3.77439200	-2.17358300
F	-1.07729100	6.05598500	0.13891500
F	-2.97644000	-2.97710200	-1.18990800
F	-2.03581600	-1.97590900	1.61272500
F	1.08531600	-1.47873500	2.46482700
F	2.39252500	-1.21212700	-0.97435300
F	-0.46279600	-1.66998900	-2.48664100
F	2.94914500	-3.78596400	1.02236500
F	2.07027800	-3.77439200	-2.17358300
F	-0.67628500	-5.21624000	-2.35013600
F	-1.92386600	-5.46398400	0.90409200
F	0.16052100	-4.14915800	2.79209500
F	1.07729100	-6.05598500	0.13891500

E(RB3LYP) = -2892.42297294

Zero-point correction= 0.156472 (Hartree/Particle)
Thermal correction to Energy= 0.201048
Thermal correction to Enthalpy= 0.201992
Thermal correction to Gibbs Free Energy= 0.080894
Sum of electronic and zero-point Energies= -2892.266500
Sum of electronic and thermal Energies= -2892.221925
Sum of electronic and thermal Enthalpies= -2892.220980
Sum of electronic and thermal Free Energies= -2892.342079

	E (Thermal)	CV	S
	KCal/Mol	Cal/Mol-Kelvin	Cal/Mol-Kelvin
Total	126.160	163.152	254.874

	1	2	3
	A(B)	A(A)	A(A)
Frequencies --	11.5568	17.9972	29.5803

Part 4. $[Au(B_{11}F_{11})_2]^{3-}$: η[5],η[5]; PG D_{5d}

Au	0.00000000	0.00000000	0.00000000
B	-1.66457900	1.60403100	0.00000000
B	-1.66414100	0.49569300	1.52488600
B	-1.66417300	-1.29652000	0.94267600
B	-1.66417300	-1.29652000	-0.94267600
B	-1.66414100	0.49569300	-1.52488600
B	-3.16412000	-1.52724500	0.00000000
B	-3.16452400	-0.47172800	-1.45239700
B	-3.16452900	1.23556100	-0.89771200
B	-3.16452900	1.23556100	0.89771200
B	-3.16452400	-0.47172800	1.45239700
B	-4.11028600	-0.00013000	0.00000000
B	1.66457900	-1.60403100	0.00000000
B	1.66414100	-0.49569300	-1.52488600
B	1.66417300	1.29652000	-0.94267600
B	1.66417300	1.29652000	0.94267600
B	1.66414100	-0.49569300	1.52488600
B	3.16412000	1.52724500	0.00000000
B	3.16452400	0.47172800	1.45239700
B	3.16452900	-1.23556100	0.89771200
B	3.16452900	-1.23556100	-0.89771200
B	3.16452400	0.47172800	-1.45239700
B	4.11028600	0.00013000	0.00000000
F	-1.24304600	2.92177100	0.00000000
F	-1.23924000	0.90202500	2.77718600
F	-1.23876300	-2.36087300	1.71711600
F	-1.23876300	-2.36087300	-1.71711600
F	-1.23924000	0.90202500	-2.77718600
F	-3.79615300	-2.76749700	0.00000000
F	-3.79699400	-0.85488800	-2.63171300
F	-3.79648000	2.23874700	-1.62701400
F	-3.79648000	2.23874700	1.62701400
F	-3.79699400	-0.85488800	2.63171300
F	-5.49508300	-0.00035600	0.00000000
F	1.24304600	-2.92177100	0.00000000
F	1.23924000	-0.90202500	-2.77718600
F	1.23876300	2.36087300	-1.71711600
F	1.23876300	2.36087300	1.71711600
F	1.23924000	-0.90202500	2.77718600
F	3.79615300	2.76749700	0.00000000
F	3.79699400	0.85488800	2.63171300
F	3.79648000	-2.23874700	1.62701400
F	3.79648000	-2.23874700	-1.62701400
F	3.79699400	0.85488800	-2.63171300
F	5.49508300	0.00035600	0.00000000

E(RB3LYP) = -2881.29974554
Zero-point correction= 0.159423 (Hartree/Particle)
Thermal correction to Energy= 0.201828
Thermal correction to Enthalpy= 0.202772
Thermal correction to Gibbs Free Energy= 0.091669
Sum of electronic and zero-point Energies= -2881.140323
Sum of electronic and thermal Energies= -2881.097917
Sum of electronic and thermal Enthalpies= -2881.096973
Sum of electronic and thermal Free Energies= -2881.208077

	E (Thermal)	CV	S
	KCal/Mol	Cal/Mol-Kelvin	Cal/Mol-Kelvin
Total	126.649	161.225	233.837

	1	2	3
	A(A1U)	A(E1U)	A(E1U)
Frequencies --	32.7708	48.8586	51.4226

Part 4. $[Au(B_{11}F_{11})_2]^{3-}$: η^2,η^2; PG C_{2h}

Au	0.00000000	0.00000000	-0.00000300
B	-1.86185900	3.11856100	0.00000300
B	-0.84903300	2.52305700	1.24804100
B	1.12597300	1.84472100	0.86143700
B	1.12597000	1.84472300	-0.86144300
B	-0.84903700	2.52305900	-1.24803900
B	1.71250500	3.29081300	-0.00000300
B	0.62210900	3.49051800	-1.41643500
B	-0.96571200	4.30094400	-0.89029700
B	-0.96570800	4.30094300	0.89030300
B	0.62211300	3.49051600	1.41643400
B	0.56190800	4.63510000	0.00000000
B	1.86185900	-3.11856100	0.00000300
B	0.84903300	-2.52305700	1.24804100
B	-1.12597300	-1.84472100	0.86143700
B	-1.12597000	-1.84472200	-0.86144300
B	0.84903700	-2.52305900	-1.24803900
B	-1.71250500	-3.29081300	-0.00000300
B	-0.62210900	-3.49051800	-1.41643500
B	0.96571200	-4.30094400	-0.89029700
B	0.96570800	-4.30094300	0.89030300
B	-0.62211300	-3.49051600	1.41643400
B	-0.56190800	-4.63510000	0.00000000
F	-3.25095100	2.92980300	0.00000600
F	-1.40681600	1.83120900	2.29422000
F	1.90518800	1.27311200	1.85515700
F	1.90518200	1.27311500	-1.85516700
F	-1.40682400	1.83121300	-2.29421700
F	3.07017600	3.61263000	-0.00000500
F	1.13612700	3.85562300	-2.65935000
F	-1.48707200	5.28131200	-1.72664100
F	-1.48706600	5.28131000	1.72664900
F	1.13613700	3.85561900	2.65934700
F	1.08480000	5.91892500	0.00000000
F	3.25095100	-2.92980300	0.00000600
F	1.40681600	-1.83120900	2.29422000
F	-1.90518800	-1.27311200	1.85515700
F	-1.90518200	-1.27311500	-1.85516700
F	1.40682400	-1.83121300	-2.29421700
F	-3.07017600	-3.61263000	-0.00000500
F	-1.13612700	-3.85562300	-2.65935000
F	1.48707200	-5.28131300	-1.72664100
F	1.48706600	-5.28131000	1.72664900
F	-1.13613700	-3.85561900	2.65934700
F	-1.08480000	-5.91892500	0.00000000

E(RB3LYP) = -2881.21297131

Zero-point correction=	0.156375 (Hartree/Particle)
Thermal correction to Energy=	0.199936
Thermal correction to Enthalpy=	0.200881
Thermal correction to Gibbs Free Energy=	0.084063
Sum of electronic and zero-point Energies=	-2881.056596
Sum of electronic and thermal Energies=	-2881.013035
Sum of electronic and thermal Enthalpies=	-2881.012091
Sum of electronic and thermal Free Energies=	-2881.128909

	E (Thermal)	CV	S
	KCal/Mol	Cal/Mol-Kelvin	Cal/Mol-Kelvin
Total	125.462	161.238	245.864

	1	2	3
	A(Au)	A(Bu)	A(Au)
Frequencies --	-22.1231	23.1216	28.8628

Part 5. $[Cu(B_{11}H_{11})_2]^{3-}$: η^5,η^5; PG D_{5d}

Cu	0.00000000	0.00000000	0.00000000
B	1.53503400	1.56349200	0.00000000
B	1.53525700	0.48317300	1.48726900
B	1.53530300	-1.26504000	0.91932700
B	1.53530300	-1.26504000	-0.91932700
B	1.53525700	0.48317300	-1.48726900
B	3.02740100	-1.51459800	0.00000000
B	3.02752800	-0.46790100	-1.44058400
B	3.02737200	1.22553500	-0.89027300
B	3.02737200	1.22553500	0.89027300
B	3.02752800	-0.46790100	1.44058400
B	3.96214000	0.00012800	0.00000000
B	-1.53503400	-1.56349200	0.00000000
B	-1.53525700	-0.48317300	-1.48726900
B	-1.53530300	1.26504000	-0.91932700
B	-1.53530300	1.26504000	0.91932700
B	-1.53525700	-0.48317300	1.48726900
B	-3.02740100	1.51459800	0.00000000
B	-3.02752800	0.46790100	1.44058400
B	-3.02737200	-1.22553500	0.89027300
B	-3.02737200	-1.22553500	-0.89027300
B	-3.02752800	0.46790100	-1.44058400
B	-3.96214000	-0.00012800	0.00000000
H	1.07681400	2.66065200	0.00000000
H	1.07698500	0.82225800	2.53068800
H	1.07731100	-2.15279300	1.56417400
H	1.07731100	-2.15279300	-1.56417400
H	1.07698500	0.82225800	-2.53068800
H	3.56525100	-2.58828100	0.00000000
H	3.56538600	-0.79969300	-2.46170800
H	3.56508300	2.09427600	-1.52131900
H	3.56508300	2.09427600	1.52131900
H	3.56538600	-0.79969300	2.46170800
H	5.16421200	0.00019800	0.00000000
H	-1.07681400	-2.66065200	0.00000000
H	-1.07698500	-0.82225800	-2.53068800
H	-1.07731100	2.15279300	-1.56417400
H	-1.07731100	2.15279300	1.56417400
H	-1.07698500	-0.82225800	2.53068800
H	-3.56525100	2.58828100	0.00000000
H	-3.56538600	0.79969300	2.46170800
H	-3.56508300	-2.09427600	1.52131900
H	-3.56508300	-2.09427600	-1.52131900
H	-3.56538600	0.79969300	-2.46170800
H	-5.16421200	-0.00019800	0.00000000

E(RB3LYP) = -757.787388489

Zero-point correction= 0.303719 (Hartree/Particle)
Thermal correction to Energy= 0.321846
Thermal correction to Enthalpy= 0.322790
Thermal correction to Gibbs Free Energy= 0.262837
Sum of electronic and zero-point Energies= -757.483670
Sum of electronic and thermal Energies= -757.465543
Sum of electronic and thermal Enthalpies= -757.464599
Sum of electronic and thermal Free Energies= -757.524551

E (Thermal)	CV	S
KCal/Mol	Cal/Mol-Kelvin	Cal/Mol-Kelvin

	1	2	3
	A(A1U)	A(E1U)	A(E1U)
Frequencies --	71.3149	98.3379	98.9188

Part 5. $[Cu(B_{11}H_{11})_2]^{3-}$: η^2,η^2; PG C_2

Cu	0.00000000	0.00000000	0.06474900
B	-1.19948700	4.44204100	-1.24145300
B	-0.10000200	3.19361000	-1.46632500
B	0.75613700	1.99166200	-0.33136300
B	-0.36903600	2.10435100	0.88004600
B	-1.45942400	3.35533500	0.00856700
B	1.16696200	2.99020800	1.08398900
B	-0.40827100	3.73002400	1.45909500
B	-0.78305200	5.07022700	0.29655400
B	0.42201200	4.93603400	-1.01762400
B	1.48214000	3.52465400	-0.58829600
B	0.91135800	4.68371300	0.67407800
B	1.19948700	-4.44204100	-1.24145300
B	1.45942400	-3.35533500	0.00856700
B	0.36903600	-2.10435100	0.88004600
B	-0.75613700	-1.99166200	-0.33136200
B	0.10000200	-3.19361000	-1.46632500
B	-1.16696200	-2.99020800	1.08398900
B	-1.48214000	-3.52465400	-0.58829600
B	-0.42201200	-4.93603400	-1.01762400
B	0.78305200	-5.07022700	0.29655400
B	0.40827100	-3.73002400	1.45909500
B	-0.91135800	-4.68371300	0.67407800
H	-2.01397800	4.81808300	-2.03348600
H	-0.19159000	2.64220100	-2.52451300
H	1.36029500	1.03817800	-0.80063200
H	-0.87693100	1.27591400	1.59557800
H	-2.57282700	2.91933600	0.06905400
H	1.99841700	2.67350700	1.88335500
H	-0.83490800	3.91388200	2.56338600
H	-1.29579500	6.06087900	0.74373600
H	0.92146000	5.81183100	-1.67132500
H	2.55546900	3.55388200	-1.11840000
H	1.60459500	5.49040700	1.22814200
H	2.01397800	-4.81808300	-2.03348600
H	2.57282700	-2.91933600	0.06905400
H	0.87693100	-1.27591400	1.59557800
H	-1.36029500	-1.03817800	-0.80063200
H	0.19159000	-2.64220100	-2.52451200
H	-1.99841700	-2.67350700	1.88335600
H	-2.55546900	-3.55388200	-1.11839900
H	-0.92146000	-5.81183100	-1.67132500
H	1.29579500	-6.06087900	0.74373600
H	0.83490800	-3.91388200	2.56338600
H	-1.60459500	-5.49040700	1.22814200

E(RB3LYP) = -757.798324889

Zero-point correction= 0.297288 (Hartree/Particle)
Thermal correction to Energy= 0.318572
Thermal correction to Enthalpy= 0.319516
Thermal correction to Gibbs Free Energy= 0.247190
Sum of electronic and zero-point Energies= -757.501037
Sum of electronic and thermal Energies= -757.479753
Sum of electronic and thermal Enthalpies= -757.478809
Sum of electronic and thermal Free Energies= -757.551135

	E (Thermal)	CV	S
	KCal/Mol	Cal/Mol-Kelvin	Cal/Mol-Kelvin
Total	199.907	97.403	152.223

	1	2	3
	A(A)	A(B)	A(A)
Frequencies --	22.7877	31.0838	31.1344

Part 5. $[Ag(B_{11}H_{11})_2]^{3-}$: η[5],η[5]; PG D_{5d}

Ag	0.00000000	0.00000000	0.00000000
B	-1.70411100	1.57595100	0.00000000
B	-1.70411500	0.48708300	-1.49888400
B	-1.70434400	-1.27510700	-0.92671500
B	-1.70434400	-1.27510700	0.92671500
B	-1.70411500	0.48708300	1.49888400
B	-3.19042800	-1.51683700	0.00000000
B	-3.19053400	-0.46857800	1.44263200
B	-3.19048300	1.22719400	0.89151300
B	-3.19048300	1.22719400	-0.89151300
B	-3.19053400	-0.46857800	-1.44263200
B	-4.12202600	0.00000700	0.00000000
B	1.70411100	-1.57595100	0.00000000
B	1.70411500	-0.48708300	1.49888400
B	1.70434400	1.27510700	0.92671500
B	1.70434400	1.27510700	-0.92671500
B	1.70411500	-0.48708300	-1.49888400
B	3.19042800	1.51683700	0.00000000
B	3.19053400	0.46857800	-1.44263200
B	3.19048300	-1.22719400	-0.89151300
B	3.19048300	-1.22719400	0.89151300
B	3.19053400	0.46857800	1.44263200
B	4.12202600	-0.00000700	0.00000000
H	-1.23827100	2.67147500	0.00000000
H	-1.23818700	0.82576300	-2.54070200
H	-1.23852200	-2.16134000	-1.57079500
H	-1.23852200	-2.16134000	1.57079500
H	-1.23818700	0.82576300	2.54070200
H	-3.73614700	-2.58610700	0.00000000
H	-3.73623900	-0.79899700	2.45957100
H	-3.73611500	2.09230400	1.51999800
H	-3.73611500	2.09230400	-1.51999800
H	-3.73623900	-0.79899700	-2.45957100
H	-5.32321500	0.00002900	0.00000000
H	1.23827100	-2.67147500	0.00000000
H	1.23818700	-0.82576300	2.54070200
H	1.23852200	2.16134000	1.57079500
H	1.23852200	2.16134000	-1.57079500
H	1.23818700	-0.82576300	-2.54070200
H	3.73614700	2.58610700	0.00000000
H	3.73623900	0.79899700	-2.45957100
H	3.73611500	-2.09230400	-1.51999800
H	3.73611500	-2.09230400	1.51999800
H	3.73623900	0.79899700	2.45957100
H	5.32321500	-0.00002900	0.00000000

E(RB3LYP) = -707.391790871

Zero-point correction= 0.302278 (Hartree/Particle)
Thermal correction to Energy= 0.320968
Thermal correction to Enthalpy= 0.321912
Thermal correction to Gibbs Free Energy= 0.259994
Sum of electronic and zero-point Energies= -707.089513
Sum of electronic and thermal Energies= -707.070823
Sum of electronic and thermal Enthalpies= -707.069879
Sum of electronic and thermal Free Energies= -707.131797

	E (Thermal)	CV	S
	KCal/Mol	Cal/Mol-Kelvin	Cal/Mol-Kelvin
Total	201.410	95.219	130.317

	1	2	3
	A(A1U)	A(E1U)	A(E1U)
Frequencies --	43.0985	84.0405	84.2834

Part 5. $[Ag(B_{11}H_{11})_2]^{3-}$: η^2,η^2; PG C_2

Ag	0.00000000	0.00000000	0.09810000
B	-1.30519500	4.53666500	-1.22789000
B	-0.16110800	3.32370500	-1.43560500
B	0.82913900	2.23402500	-0.29617500
B	-0.24178000	2.37698300	0.97273300
B	-1.44475900	3.53246900	0.10615800
B	1.25625000	3.33741000	1.02943700
B	-0.32617400	4.03986700	1.45213200
B	-0.83107200	5.28550300	0.24176900
B	0.29814200	5.12113800	-1.13400800
B	1.44863900	3.79131700	-0.68582300
B	0.89817500	4.99595000	0.53468400
B	1.30519500	-4.53666500	-1.22789000
B	1.44475900	-3.53246900	0.10615800
B	0.24178000	-2.37698300	0.97273300
B	-0.82913900	-2.23402500	-0.29617500
B	0.16110800	-3.32370500	-1.43560500
B	-1.25625000	-3.33741000	1.02943700
B	-1.44863900	-3.79131700	-0.68582300
B	-0.29814200	-5.12113800	-1.13400800
B	0.83107200	-5.28550300	0.24176900
B	0.32617400	-4.03986700	1.45213200
B	-0.89817500	-4.99595000	0.53468400
H	-2.18034900	4.82159900	-1.99201100
H	-0.29696000	2.70334900	-2.45001300
H	1.50304400	1.33290200	-0.75513600
H	-0.67018800	1.60866000	1.79413900
H	-2.53226200	3.05688000	0.26383900
H	2.14683100	3.11666900	1.79801800
H	-0.69307000	4.26402100	2.57017700
H	-1.36505200	6.27933100	0.65481200
H	0.71494800	5.97287400	-1.87148400
H	2.48631100	3.83536400	-1.28123000
H	1.58807900	5.86006900	0.99838300
H	2.18034900	-4.82159900	-1.99201100
H	2.53226200	-3.05688000	0.26383800
H	0.67018800	-1.60866000	1.79413900
H	-1.50304400	-1.33290200	-0.75513600
H	0.29696000	-2.70334900	-2.45001300
H	-2.14683100	-3.11666900	1.79801800
H	-2.48631100	-3.83536400	-1.28123000
H	-0.71494800	-5.97287400	-1.87148400
H	1.36505200	-6.27933100	0.65481200
H	0.69307000	-4.26402100	2.57017700
H	-1.58807900	-5.86006900	0.99838300

E(RB3LYP) = -707.417358850

Zero-point correction= 0.297196 (Hartree/Particle)
Thermal correction to Energy= 0.318672
Thermal correction to Enthalpy= 0.319617
Thermal correction to Gibbs Free Energy= 0.244984
Sum of electronic and zero-point Energies= -707.120163
Sum of electronic and thermal Energies= -707.098686
Sum of electronic and thermal Enthalpies= -707.097742
Sum of electronic and thermal Free Energies= -707.172375

	E (Thermal)	CV	S
	KCal/Mol	Cal/Mol-Kelvin	Cal/Mol-Kelvin
Total	199.970	97.549	157.078

	1	2	3
	A(A)	A(A)	A(B)
Frequencies --	9.8471	25.1110	26.3511

Part 5. $[Au(B_{11}H_{11})_2]^{3-}$: η^5,η^5; PG D_{5d}

Au	0.00000000	0.00000000	0.00000000
B	-1.67706500	1.58321800	0.00000000
B	-1.67716800	0.48929400	-1.50582000
B	-1.67743200	-1.28097400	-0.93096400
B	-1.67743200	-1.28097400	0.93096400
B	-1.67716800	0.48929400	1.50582000
B	-3.16346200	-1.51493300	0.00000000
B	-3.16354100	-0.46792600	1.44088800
B	-3.16345100	1.22579400	0.89041300
B	-3.16345100	1.22579400	-0.89041300
B	-3.16354100	-0.46792600	-1.44088800
B	-4.09477000	0.00006300	0.00000000
B	1.67706500	-1.58321800	0.00000000
B	1.67716800	-0.48929400	1.50582000
B	1.67743200	1.28097400	0.93096400
B	1.67743200	1.28097400	-0.93096400
B	1.67716800	-0.48929400	-1.50582000
B	3.16346200	1.51493300	0.00000000
B	3.16354100	0.46792600	-1.44088800
B	3.16345100	-1.22579400	-0.89041300
B	3.16345100	-1.22579400	0.89041300
B	3.16354100	0.46792600	1.44088800
B	4.09477000	-0.00006300	0.00000000
H	-1.23073800	2.68663000	0.00000000
H	-1.23082200	0.83032200	-2.55520300
H	-1.23127100	-2.17369600	-1.57960600
H	-1.23127100	-2.17369600	1.57960600
H	-1.23082200	0.83032200	2.55520300
H	-3.71198500	-2.58258300	0.00000000
H	-3.71194000	-0.79779300	2.45636100
H	-3.71169200	2.08970700	1.51795100
H	-3.71169200	2.08970700	-1.51795100
H	-3.71194000	-0.79779300	-2.45636100
H	-5.29606500	0.00004800	0.00000000
H	1.23073800	-2.68663000	0.00000000
H	1.23082200	-0.83032200	2.55520300
H	1.23127100	2.17369600	1.57960600
H	1.23127100	2.17369600	-1.57960600
H	1.23082200	-0.83032200	-2.55520300
H	3.71198500	2.58258300	0.00000000
H	3.71194000	0.79779300	-2.45636100
H	3.71169200	-2.08970700	-1.51795100
H	3.71169200	-2.08970700	1.51795100
H	3.71194000	0.79779300	2.45636100
H	5.29606500	-0.00004800	0.00000000

E(RB3LYP) = -696.235401701

Zero-point correction= 0.304096 (Hartree/Particle)
Thermal correction to Energy= 0.322348
Thermal correction to Enthalpy= 0.323292
Thermal correction to Gibbs Free Energy= 0.261965
Sum of electronic and zero-point Energies= -695.931306
Sum of electronic and thermal Energies= -695.913054
Sum of electronic and thermal Enthalpies= -695.912110
Sum of electronic and thermal Free Energies= -695.973437

	E (Thermal)	CV	S
	KCal/Mol	Cal/Mol-Kelvin	Cal/Mol-Kelvin
Total	202.276	93.928	129.074

	1	2	3
	A(A1U)	A(E1U)	A(E1U)
Frequencies --	45.1406	86.3032	86.4951

Part 5. $[Au(B_{11}H_{11})_2]^{3-}$: η^2,η^2; PG C_2

Au	0.00000000	0.00000000	0.12917600
B	-1.74264500	3.87915500	-0.65421200
B	-0.57524500	2.87516600	-1.33150200
B	0.99131900	2.00993700	-0.62450300
B	0.52166800	2.13812500	1.01257200
B	-1.14815900	3.03401100	0.67133800
B	1.67304000	3.32136300	0.36164500
B	0.29804700	3.83507500	1.37854000
B	-0.86354600	4.85362900	0.45315300
B	-0.37607500	4.72010400	-1.26138800
B	1.05839700	3.62856800	-1.28604700
B	0.85715000	4.85259000	0.01037000
B	1.74264500	-3.87915500	-0.65421200
B	1.14815900	-3.03401100	0.67133800
B	-0.52166800	-2.13812500	1.01257200
B	-0.99131900	-2.00993700	-0.62450400
B	0.57524500	-2.87516600	-1.33150200
B	-1.67304000	-3.32136300	0.36164500
B	-1.05839700	-3.62856800	-1.28604700
B	0.37607500	-4.72010400	-1.26138800
B	0.86354600	-4.85362900	0.45315300
B	-0.29804800	-3.83507500	1.37854000
B	-0.85715000	-4.85259000	0.01037000
H	-2.88939500	3.94506100	-0.98643400
H	-0.99456100	2.16021500	-2.19072800
H	1.64834300	1.27157500	-1.29975700
H	0.69175700	1.52831400	2.02807900
H	-1.98306100	2.43411400	1.28029600
H	2.82525400	3.34436900	0.68959800
H	0.39925600	4.11212300	2.53909500
H	-1.35655200	5.79269300	1.01765100
H	-0.46105700	5.54728100	-2.12840300
H	1.76210300	3.74620100	-2.24706100
H	1.52301000	5.84342300	0.12312300
H	2.88939500	-3.94506100	-0.98643400
H	1.98306100	-2.43411400	1.28029600
H	-0.69175700	-1.52831400	2.02807900
H	-1.64834300	-1.27157500	-1.29975700
H	0.99456100	-2.16021500	-2.19072800
H	-2.82525400	-3.34436900	0.68959800
H	-1.76210300	-3.74620100	-2.24706100
H	0.46105700	-5.54728100	-2.12840300
H	1.35655200	-5.79269300	1.01765100
H	-0.39925600	-4.11212300	2.53909500
H	-1.52301000	-5.84342300	0.12312300

E(RB3LYP) = -696.193160397

Zero-point correction= 0.298366 (Hartree/Particle)
Thermal correction to Energy= 0.319250
Thermal correction to Enthalpy= 0.320195
Thermal correction to Gibbs Free Energy= 0.248677
Sum of electronic and zero-point Energies= -695.894794
Sum of electronic and thermal Energies= -695.873910
Sum of electronic and thermal Enthalpies= -695.872966
Sum of electronic and thermal Free Energies= -695.944483

	E (Thermal)	CV	S
	KCal/Mol	Cal/Mol-Kelvin	Cal/Mol-Kelvin
Total	200.333	97.611	150.521

	1	2	3
	A(A)	A(B)	A(A)
Frequencies --	14.7050	21.8078	36.1337

Part 5. $[Cu(B_{11}F_{11})_2]^{3-}$: η[5],η[5]; PG D_{5d}

Cu	0.00000000	0.00000000	0.00000000
B	-1.53142000	1.58748200	0.00000000
B	-1.53146900	0.49061500	-1.50997400
B	-1.53132700	-1.28427200	-0.93335400
B	-1.53132700	-1.28427200	0.93335400
B	-1.53146900	0.49061500	1.50997400
B	-3.04220200	-1.52601700	0.00000000
B	-3.04242800	-0.47152800	1.45134900
B	-3.04244900	1.23456100	0.89693100
B	-3.04244900	1.23456100	-0.89693100
B	-3.04242800	-0.47152800	-1.45134900
B	-3.98975800	-0.00011100	0.00000000
B	1.53142000	-1.58748200	0.00000000
B	1.53146900	-0.49061500	1.50997400
B	1.53132700	1.28427200	0.93335400
B	1.53132700	1.28427200	-0.93335400
B	1.53146900	-0.49061500	-1.50997400
B	3.04220200	1.52601700	0.00000000
B	3.04242800	0.47152800	-1.45134900
B	3.04244900	-1.23456100	-0.89693100
B	3.04244900	-1.23456100	0.89693100
B	3.04242800	0.47152800	1.45134900
B	3.98975800	0.00011100	0.00000000
F	-1.13129500	2.89662600	0.00000000
F	-1.13131700	0.89512700	-2.75502400
F	-1.13126700	-2.34344100	-1.70277300
F	-1.13126700	-2.34344100	1.70277300
F	-1.13131700	0.89512700	2.75502400
F	-3.67829200	-2.75551900	0.00000000
F	-3.67868800	-0.85146700	2.62056900
F	-3.67862400	2.22923700	1.61954000
F	-3.67862400	2.22923700	-1.61954000
F	-3.67868800	-0.85146700	-2.62056900
F	-5.36897000	-0.00019800	0.00000000
F	1.13129500	-2.89662600	0.00000000
F	1.13131700	-0.89512700	2.75502400
F	1.13126700	2.34344100	1.70277300
F	1.13126700	2.34344100	-1.70277300
F	1.13131700	-0.89512700	-2.75502400
F	3.67829200	2.75551900	0.00000000
F	3.67868800	0.85146700	-2.62056900
F	3.67862400	-2.22923700	-1.61954000
F	3.67862400	-2.22923700	1.61954000
F	3.67868800	0.85146700	2.62056900
F	5.36897000	0.00019800	0.00000000

E(RB3LYP) = -2943.19659516

Zero-point correction= 0.161211 (Hartree/Particle)
Thermal correction to Energy= 0.202875
Thermal correction to Enthalpy= 0.203819
Thermal correction to Gibbs Free Energy= 0.095553
Sum of electronic and zero-point Energies= -2943.035385
Sum of electronic and thermal Energies= -2942.993720
Sum of electronic and thermal Enthalpies= -2942.992776
Sum of electronic and thermal Free Energies= -2943.101042

	E (Thermal)	CV	S
	KCal/Mol	Cal/Mol-Kelvin	Cal/Mol-Kelvin
Total	127.306	160.511	227.865

	1	2	3
	A(A1U)	A(E1U)	A(E1U)
Frequencies --	49.5272	55.4812	55.7754

Part 5. $[Cu(B_{11}F_{11})_2]^{3-}$: η^2,η^2; PG C_2

Cu	0.00000000	0.00000000	0.22857500
B	1.74387800	3.21085500	-0.71306200
B	0.38969800	2.42946000	-1.41192300
B	-1.26983700	1.74072900	-0.34204600
B	-0.67007800	1.87545600	1.22960200
B	1.24241700	2.61570100	0.80919900
B	-1.62487500	3.24201300	0.57284200
B	-0.10940900	3.57192000	1.47601500
B	1.14270000	4.39147300	0.37875200
B	0.50401800	4.25050600	-1.28800500
B	-1.11461900	3.35257200	-1.14216200
B	-0.61703300	4.61114200	0.07283500
B	-1.74387800	-3.21085500	-0.71306200
B	-1.24241700	-2.61570100	0.80919900
B	0.67007800	-1.87545600	1.22960200
B	1.26983700	-1.74072900	-0.34204600
B	-0.38969800	-2.42946000	-1.41192400
B	1.62487500	-3.24201300	0.57284200
B	1.11461900	-3.35257200	-1.14216200
B	-0.50401800	-4.25050600	-1.28800500
B	-1.14270000	-4.39147300	0.37875200
B	0.10940900	-3.57192000	1.47601500
B	0.61703300	-4.61114200	0.07283500
F	3.03895300	3.08675500	-1.19832300
F	0.59871000	1.62117700	-2.48633200
F	-2.24413700	1.01231900	-0.98211200
F	-0.93539700	1.31269300	2.45419700
F	2.15790100	1.96124800	1.57900100
F	-2.89921900	3.53190100	1.03854000
F	-0.13858100	4.00519400	2.79163900
F	1.86335600	5.44584800	0.91697800
F	0.62715100	5.17299600	-2.31456600
F	-2.03469800	3.59212400	-2.14858800
F	-1.16376000	5.87670700	0.17732300
F	-3.03895300	-3.08675500	-1.19832300
F	-2.15790100	-1.96124800	1.57900100
F	0.93539700	-1.31269300	2.45419700
F	2.24413700	-1.01231900	-0.98211200
F	-0.59871000	-1.62117700	-2.48633200
F	2.89921900	-3.53190100	1.03854000
F	2.03469800	-3.59212400	-2.14858800
F	-0.62715100	-5.17299600	-2.31456600
F	-1.86335600	-5.44584700	0.91697800
F	0.13858100	-4.00519300	2.79163900
F	1.16375900	-5.87670700	0.17732300

E(RB3LYP) = -2943.15386373

Zero-point correction= 0.158555 (Hartree/Particle)
Thermal correction to Energy= 0.202636
Thermal correction to Enthalpy= 0.203581
Thermal correction to Gibbs Free Energy= 0.083874
Sum of electronic and zero-point Energies= -2942.995309
Sum of electronic and thermal Energies= -2942.951227
Sum of electronic and thermal Enthalpies= -2942.950283
Sum of electronic and thermal Free Energies= -2943.069990

	E (Thermal)	CV	S
	KCal/Mol	Cal/Mol-Kelvin	Cal/Mol-Kelvin
Total	127.156	162.136	251.943

	1	2	3
	A(A)	A(B)	A(A)
Frequencies --	12.7155	17.4870	24.7693

Part 5. $[Ag(B_{11}F_{11})_2]^{3-}$: η^5,η^5; PG D_{5d}

Ag	0.00000000	0.00000000	0.00000000
B	-1.70747800	1.60135900	0.00000000
B	-1.70753700	0.49485100	1.52320000
B	-1.70768800	-1.29586200	0.94163000
B	-1.70768800	-1.29586200	-0.94163000
B	-1.70753700	0.49485100	-1.52320000
B	-3.21226000	-1.52873500	0.00000000
B	-3.21234800	-0.47239700	-1.45380700
B	-3.21230900	1.23652300	-0.89839500
B	-3.21230900	1.23652300	0.89839500
B	-3.21234800	-0.47239700	1.45380700
B	-4.15663900	-0.00011000	0.00000000
B	1.70747800	-1.60135900	0.00000000
B	1.70753700	-0.49485100	-1.52320000
B	1.70768800	1.29586200	-0.94163000
B	1.70768800	1.29586200	0.94163000
B	1.70753700	-0.49485100	1.52320000
B	3.21226000	1.52873500	0.00000000
B	3.21234800	0.47239700	1.45380700
B	3.21230900	-1.23652300	0.89839500
B	3.21230900	-1.23652300	-0.89839500
B	3.21234800	0.47239700	-1.45380700
B	4.15663900	0.00011000	0.00000000
F	-1.28748500	2.90521400	0.00000000
F	-1.28742100	0.89810500	2.76309900
F	-1.28690400	-2.35054500	1.70789000
F	-1.28690400	-2.35054500	-1.70789000
F	-1.28742100	0.89810500	-2.76309900
F	-3.85602000	-2.75458700	0.00000000
F	-3.85611600	-0.85108400	-2.61968600
F	-3.85580300	2.22838000	-1.61898400
F	-3.85580300	2.22838000	1.61898400
F	-3.85611600	-0.85108400	2.61968600
F	-5.53638300	-0.00011900	0.00000000
F	1.28748500	-2.90521400	0.00000000
F	1.28742100	-0.89810500	-2.76309900
F	1.28690400	2.35054500	-1.70789000
F	1.28690400	2.35054500	1.70789000
F	1.28742100	-0.89810500	2.76309900
F	3.85602000	2.75458700	0.00000000
F	3.85611600	0.85108400	2.61968600
F	3.85580300	-2.22838000	1.61898400
F	3.85580300	-2.22838000	-1.61898400
F	3.85611600	0.85108400	-2.61968600
F	5.53638300	0.00011900	0.00000000

E(RB3LYP) = -2892.79298820

Zero-point correction= 0.159670 (Hartree/Particle)
Thermal correction to Energy= 0.202210
Thermal correction to Enthalpy= 0.203155
Thermal correction to Gibbs Free Energy= 0.091587
Sum of electronic and zero-point Energies= -2892.633318
Sum of electronic and thermal Energies= -2892.590778
Sum of electronic and thermal Enthalpies= -2892.589834
Sum of electronic and thermal Free Energies= -2892.701401

	E (Thermal)	CV	S
	KCal/Mol	Cal/Mol-Kelvin	Cal/Mol-Kelvin
Total	126.889	161.489	234.814

	1	2	3
	A(A1U)	A(E1U)	A(E1U)
Frequencies --	27.1796	45.9844	46.4719

Part 5. $[Ag(B_{11}F_{11})_2]^{3-}$: η^2,η^2; PG C_2

Ag	0.00000000	0.00000000	0.16840700
B	1.76795800	3.61077100	-0.67762600
B	0.43437600	2.81904400	-1.39277400
B	-1.23785800	2.11164100	-0.41133800
B	-0.67830900	2.20621000	1.16534600
B	1.21253700	2.94814600	0.79351200
B	-1.63618000	3.58700700	0.53363500
B	-0.14270400	3.89571700	1.47599900
B	1.13649800	4.74620500	0.43665800
B	0.53447300	4.64624900	-1.25697800
B	-1.08239300	3.74006500	-1.16352900
B	-0.61652000	4.97150200	0.08969700
B	-1.76795800	-3.61077100	-0.67762600
B	-1.21253700	-2.94814600	0.79351200
B	0.67830900	-2.20621000	1.16534600
B	1.23785800	-2.11164100	-0.41133800
B	-0.43437600	-2.81904400	-1.39277400
B	1.63618000	-3.58700700	0.53363500
B	1.08239300	-3.74006500	-1.16352900
B	-0.53447300	-4.64624900	-1.25697800
B	-1.13649800	-4.74620500	0.43665800
B	0.14270400	-3.89571700	1.47599900
B	0.61652000	-4.97150200	0.08969700
F	3.07224000	3.47917200	-1.13275100
F	0.68628800	2.00123700	-2.45630000
F	-2.17342500	1.38272900	-1.11022000
F	-0.93758500	1.59293400	2.36968700
F	2.10870700	2.23580500	1.54022400
F	-2.92267700	3.85583300	0.97623900
F	-0.19280000	4.28503100	2.80404600
F	1.83695100	5.78711200	1.02432400
F	0.67010700	5.59361900	-2.25842000
F	-1.97175500	3.99118600	-2.19421900
F	-1.16689400	6.23361100	0.21154200
F	-3.07224000	-3.47917200	-1.13275100
F	-2.10870700	-2.23580500	1.54022400
F	0.93758500	-1.59293400	2.36968700
F	2.17342500	-1.38272900	-1.11022000
F	-0.68628800	-2.00123700	-2.45630000
F	2.92267700	-3.85583300	0.97623900
F	1.97175500	-3.99118600	-2.19421900
F	-0.67010700	-5.59361900	-2.25842000
F	-1.83695100	-5.78711200	1.02432400
F	0.19280000	-4.28503100	2.80404600
F	1.16689400	-6.23361100	0.21154200

E(RB3LYP) = -2892.77867134

Zero-point correction= 0.157992 (Hartree/Particle)
Thermal correction to Energy= 0.202455
Thermal correction to Enthalpy= 0.203399
Thermal correction to Gibbs Free Energy= 0.081557
Sum of electronic and zero-point Energies= -2892.620680
Sum of electronic and thermal Energies= -2892.576216
Sum of electronic and thermal Enthalpies= -2892.575272
Sum of electronic and thermal Free Energies= -2892.697115

	E (Thermal)	CV	S
	KCal/Mol	Cal/Mol-Kelvin	Cal/Mol-Kelvin
Total	127.043	162.397	256.440

	1	2	3
	A(A)	A(B)	A(A)
Frequencies --	7.2578	16.0952	19.8482

Part 5. $[Au(B_{11}F_{11})_2]^{3-}$: η^5,η^5; PG D_{5d}

Au	0.00000000	0.00000000	0.00000000
B	-1.68236400	1.60984700	0.00000000
B	-1.68253800	0.49747000	1.53132600
B	-1.68259000	-1.30269100	0.94659600
B	-1.68259000	-1.30269100	-0.94659600
B	-1.68253800	0.49747000	-1.53132600
B	-3.18305000	-1.52728500	0.00000000
B	-3.18315900	-0.47197400	-1.45242800
B	-3.18311100	1.23533800	-0.89753000
B	-3.18311100	1.23533800	0.89753000
B	-3.18315900	-0.47197400	1.45242800
B	-4.12426600	-0.00013400	0.00000000
B	1.68236400	-1.60984700	0.00000000
B	1.68253800	-0.49747000	-1.53132600
B	1.68259000	1.30269100	-0.94659600
B	1.68259000	1.30269100	0.94659600
B	1.68253800	-0.49747000	1.53132600
B	3.18305000	1.52728500	0.00000000
B	3.18315900	0.47197400	1.45242800
B	3.18311100	-1.23533800	0.89753000
B	3.18311100	-1.23533800	-0.89753000
B	3.18315900	0.47197400	-1.45242800
B	4.12426600	0.00013400	0.00000000
F	-1.27035100	2.91564600	0.00000000
F	-1.27048900	0.90137400	2.77308700
F	-1.26977700	-2.35903600	1.71387900
F	-1.26977700	-2.35903600	-1.71387900
F	-1.27048900	0.90137400	-2.77308700
F	-3.82988900	-2.75262800	0.00000000
F	-3.83006300	-0.85051900	-2.61778900
F	-3.82975800	2.22676400	-1.61778900
F	-3.82975800	2.22676400	1.61778900
F	-3.83006300	-0.85051900	2.61778900
F	-5.50591100	-0.00014000	0.00000000
F	1.27035100	-2.91564600	0.00000000
F	1.27048900	-0.90137400	-2.77308700
F	1.26977700	2.35903600	-1.71387900
F	1.26977700	2.35903600	1.71387900
F	1.27048900	-0.90137400	2.77308700
F	3.82988900	2.75262800	0.00000000
F	3.83006300	0.85051900	2.61778900
F	3.82975800	-2.22676400	1.61778900
F	3.82975800	-2.22676400	-1.61778900
F	3.83006300	0.85051900	-2.61778900
F	5.50591100	0.00014000	0.00000000

E(RB3LYP) = -2881.63254435

Zero-point correction= 0.160692 (Hartree/Particle)
Thermal correction to Energy= 0.202887
Thermal correction to Enthalpy= 0.203831
Thermal correction to Gibbs Free Energy= 0.093824
Sum of electronic and zero-point Energies= -2881.471852
Sum of electronic and thermal Energies= -2881.429658
Sum of electronic and thermal Enthalpies= -2881.428713
Sum of electronic and thermal Free Energies= -2881.538721

	E (Thermal)	CV	S
	KCal/Mol	Cal/Mol-Kelvin	Cal/Mol-Kelvin
Total	127.313	160.760	231.530

	1	2	3
	A(A1U)	A(E1U)	A(E1U)
Frequencies --	30.3809	49.1131	49.3904

Part 5. $[Au(B_{11}F_{11})_2]^{3-}$: η^2,η^2; PG C_2

Au	0.00000000	0.00000000	0.28066900
B	1.69168300	3.15066500	-0.87943600
B	0.25984700	2.43649300	-1.51078200
B	-1.36856500	1.81254300	-0.24933300
B	-0.63271200	1.98973500	1.30641200
B	1.32862800	2.68534000	0.72831500
B	-1.55701600	3.35769900	0.63068000
B	0.03356900	3.67641000	1.41970800
B	1.21604300	4.41711400	0.19312100
B	0.45608000	4.23606300	-1.40782700
B	-1.17679200	3.39957300	-1.12779500
B	-0.55474100	4.67463400	0.01493000
B	-1.69168300	-3.15066500	-0.87943600
B	-1.32862800	-2.68534000	0.72831500
B	0.63271200	-1.98973500	1.30641200
B	1.36856500	-1.81254300	-0.24933300
B	-0.25984700	-2.43649300	-1.51078200
B	1.55701600	-3.35769900	0.63068000
B	1.17679200	-3.39957300	-1.12779500
B	-0.45608000	-4.23606300	-1.40782700
B	-1.21604300	-4.41711400	0.19312100
B	-0.03356900	-3.67641000	1.41970800
B	0.55474100	-4.67463400	0.01493000
F	2.94306900	2.97837500	-1.45907500
F	0.35559500	1.63459900	-2.60119400
F	-2.48138200	1.19005500	-0.75257800
F	-0.89979100	1.55861000	2.57994400
F	2.28730600	2.08992300	1.48670100
F	-2.78155300	3.71403500	1.17419800
F	0.09082500	4.17751500	2.71059200
F	2.00451500	5.47113900	0.62249900
F	0.54637700	5.12258000	-2.46752700
F	-2.17245300	3.66140600	-2.05310000
F	-1.06001200	5.95553600	0.11625200
F	-2.94306900	-2.97837500	-1.45907500
F	-2.28730600	-2.08992300	1.48670100
F	0.89979100	-1.55861000	2.57994400
F	2.48138200	-1.19005500	-0.75257800
F	-0.35559500	-1.63459900	-2.60119400
F	2.78155300	-3.71403500	1.17419800
F	2.17245300	-3.66140600	-2.05310000
F	-0.54637700	-5.12258000	-2.46752700
F	-2.00451500	-5.47113900	0.62249900
F	-0.09082500	-4.17751500	2.71059200
F	1.06001200	-5.95553600	0.11625200

E(RB3LYP) = -2881.57155653

Zero-point correction= 0.158246 (Hartree/Particle)
Thermal correction to Energy= 0.202309
Thermal correction to Enthalpy= 0.203253
Thermal correction to Gibbs Free Energy= 0.084041
Sum of electronic and zero-point Energies= -2881.413311
Sum of electronic and thermal Energies= -2881.369248
Sum of electronic and thermal Enthalpies= -2881.368304
Sum of electronic and thermal Free Energies= -2881.487515

	E (Thermal)	CV	S
	KCal/Mol	Cal/Mol-Kelvin	Cal/Mol-Kelvin
Total	126.951	162.317	250.902

	1	2	3
	A(A)	A(B)	A(A)
Frequencies --	9.4718	20.4523	23.5964

Part 6. $[Cu(B_{11}H_{11})_2]^{3-}$: η^5,η^5; PG D_{5d}

Cu	0.00000000	0.00000000	0.00000000
B	1.51797500	1.56055000	0.00000000
B	1.51726000	0.48346900	1.48332300
B	1.51658600	-1.26053400	0.91708000
B	1.51658600	-1.26053400	-0.91708000
B	1.51726000	0.48346900	-1.48332300
B	3.00490600	-1.51079400	0.00000000
B	3.00581500	-0.46630700	-1.43746600
B	3.00676300	1.22314800	-0.88823500
B	3.00676300	1.22314800	0.88823500
B	3.00581500	-0.46630700	1.43746600
B	3.93907000	-0.00026100	0.00000000
B	-1.51797500	-1.56055100	0.00000000
B	-1.51726000	-0.48346900	-1.48332300
B	-1.51658600	1.26053400	-0.91708000
B	-1.51658600	1.26053400	0.91708000
B	-1.51726000	-0.48346900	1.48332300
B	-3.00490600	1.51079400	0.00000000
B	-3.00581500	0.46630700	1.43746600
B	-3.00676300	-1.22314800	0.88823500
B	-3.00676300	-1.22314800	-0.88823500
B	-3.00581500	0.46630700	-1.43746600
B	-3.93907000	0.00026100	0.00000000
H	1.06721000	2.66015800	0.00000000
H	1.06423800	0.82430900	2.52802100
H	1.06565900	-2.14916200	1.56448600
H	1.06565900	-2.14916200	-1.56448600
H	1.06423800	0.82430900	-2.52802100
H	3.53591900	-2.58183500	0.00000000
H	3.53678500	-0.79813200	-2.45583500
H	3.53823700	2.08941200	-1.51760000
H	3.53823700	2.08941200	1.51760000
H	3.53678500	-0.79813200	2.45583500
H	5.13369300	-0.00100400	0.00000000
H	-1.06721000	-2.66015800	0.00000000
H	-1.06423800	-0.82430900	-2.52802100
H	-1.06565900	2.14916200	-1.56448600
H	-1.06565900	2.14916200	1.56448600
H	-1.06423800	-0.82430900	2.52802100
H	-3.53591900	2.58183500	0.00000000
H	-3.53678500	0.79813200	2.45583500
H	-3.53823700	-2.08941200	1.51760000
H	-3.53823700	-2.08941200	-1.51760000
H	-3.53678500	0.79813200	-2.45583500
H	-5.13369300	0.00100400	0.00000000

E(RB3LYP) = -758.261664545

Zero-point correction= 0.306287 (Hartree/Particle)
Thermal correction to Energy= 0.324259
Thermal correction to Enthalpy= 0.325203
Thermal correction to Gibbs Free Energy= 0.265163
Sum of electronic and zero-point Energies= -757.955378
Sum of electronic and thermal Energies= -757.937406
Sum of electronic and thermal Enthalpies= -757.936462
Sum of electronic and thermal Free Energies= -757.996502

	E (Thermal)	CV	S
	KCal/Mol	Cal/Mol-Kelvin	Cal/Mol-Kelvin
Total	203.475	93.388	126.365

	1	2	3
	A(A1U)	A(E1U)	A(E1U)
Frequencies --	45.4063	93.0234	99.8464

Part 6. $[Cu(B_{11}H_{11})_2]^{3-}$: η^2,η^2; PG C_2

Cu	0.00000000	0.00000000	0.45924500
B	-1.16140900	4.01015800	-1.35965100
B	0.02742900	2.83140400	-1.40211400
B	0.93219600	1.85707500	-0.09476800
B	-0.21866700	2.07612300	1.08884100
B	-1.36529600	3.10153000	0.03181500
B	1.25267300	3.06731500	1.16221600
B	-0.37092000	3.75465700	1.41551100
B	-0.81145700	4.88015900	0.07092400
B	0.41981800	4.64182200	-1.19766700
B	1.55463600	3.38332600	-0.56583400
B	0.89495300	4.66397100	0.51363200
B	1.16140900	-4.01015800	-1.35965100
B	1.36529600	-3.10153000	0.03181500
B	0.21866700	-2.07612300	1.08884100
B	-0.93219600	-1.85707500	-0.09476800
B	-0.02742900	-2.83140400	-1.40211400
B	-1.25267300	-3.06731600	1.16221600
B	-1.55463600	-3.38332600	-0.56583400
B	-0.41981800	-4.64182100	-1.19766700
B	0.81145700	-4.88015900	0.07092400
B	0.37092000	-3.75465700	1.41551100
B	-0.89495300	-4.66397200	0.51363200
H	-1.98503500	4.20833600	-2.19751500
H	-0.00845400	2.13024000	-2.36995800
H	1.60075400	0.89821200	-0.41893800
H	-0.68325400	1.33821200	1.92725600
H	-2.44657700	2.60293300	0.14099800
H	2.08979300	2.92453000	2.00202500
H	-0.82646000	4.06394300	2.47546800
H	-1.39414700	5.88136500	0.36801300
H	0.86880100	5.44294300	-1.96367400
H	2.63094300	3.39849100	-1.08329100
H	1.52268500	5.58101400	0.95162800
H	1.98503500	-4.20833600	-2.19751500
H	2.44657700	-2.60293300	0.14099800
H	0.68325400	-1.33821200	1.92725600
H	-1.60075400	-0.89821200	-0.41893800
H	0.00845400	-2.13024000	-2.36995800
H	-2.08979300	-2.92453000	2.00202500
H	-2.63094300	-3.39849100	-1.08329100
H	-0.86880100	-5.44294300	-1.96367400
H	1.39414700	-5.88136500	0.36801300
H	0.82646000	-4.06394300	2.47546800
H	-1.52268500	-5.58101400	0.95162800

E(RB3LYP) = -758.237646518

Zero-point correction= 0.300041 (Hartree/Particle)
Thermal correction to Energy= 0.320938
Thermal correction to Enthalpy= 0.321882
Thermal correction to Gibbs Free Energy= 0.250857
Sum of electronic and zero-point Energies= -757.937605
Sum of electronic and thermal Energies= -757.916709
Sum of electronic and thermal Enthalpies= -757.915765
Sum of electronic and thermal Free Energies= -757.986789

	E (Thermal)	CV	S
	KCal/Mol	Cal/Mol-Kelvin	Cal/Mol-Kelvin
Total	201.391	96.545	149.483

	1	2	3
	A(A)	A(B)	A(A)
Frequencies --	22.3085	25.2376	31.5307

Part 6. $[Ag(B_{11}H_{11})_2]^{3-}$: η^5,η^5; PG D_{5d}

Ag	0.00000000	0.00000000	0.00000000
B	-1.68496200	1.57274500	0.00000000
B	-1.68553800	0.48602100	-1.49591000
B	-1.68546900	-1.27259200	-0.92462000
B	-1.68546900	-1.27259200	0.92462000
B	-1.68553800	0.48602100	1.49591000
B	-3.16784500	-1.51349400	0.00000000
B	-3.16811800	-0.46763000	1.43958600
B	-3.16803700	1.22464900	0.88954000
B	-3.16803700	1.22464900	-0.88954000
B	-3.16811800	-0.46763000	-1.43958600
B	-4.09769100	-0.00006800	0.00000000
B	1.68496200	-1.57274500	0.00000000
B	1.68553800	-0.48602100	1.49591000
B	1.68546900	1.27259200	0.92462000
B	1.68546900	1.27259200	-0.92462000
B	1.68553800	-0.48602100	-1.49591000
B	3.16784500	1.51349400	0.00000000
B	3.16811800	0.46763000	-1.43958600
B	3.16803700	-1.22464900	-0.88954000
B	3.16803700	-1.22464900	0.88954000
B	3.16811800	0.46763000	1.43958600
B	4.09769100	0.00006800	0.00000000
H	-1.22378400	2.66982400	0.00000000
H	-1.22659900	0.82547400	-2.54018200
H	-1.22692100	-2.16171400	-1.56912100
H	-1.22692100	-2.16171400	1.56912100
H	-1.22659900	0.82547400	2.54018200
H	-3.70738800	-2.57981400	0.00000000
H	-3.70724500	-0.79765900	2.45376800
H	-3.70726900	2.08739900	1.51637500
H	-3.70726900	2.08739900	-1.51637500
H	-3.70724500	-0.79765900	-2.45376800
H	-5.29173100	-0.00007600	0.00000000
H	1.22378400	-2.66982400	0.00000000
H	1.22659900	-0.82547400	2.54018200
H	1.22692100	2.16171400	1.56912100
H	1.22692100	2.16171400	-1.56912100
H	1.22659900	-0.82547400	-2.54018200
H	3.70738800	2.57981400	0.00000000
H	3.70724500	0.79765900	-2.45376800
H	3.70726900	-2.08739900	-1.51637500
H	3.70726900	-2.08739900	1.51637500
H	3.70724500	0.79765900	2.45376800
H	5.29173100	0.00007600	0.00000000

E(RB3LYP) = -707.860859271

Zero-point correction= 0.304893 (Hartree/Particle)
Thermal correction to Energy= 0.323406
Thermal correction to Enthalpy= 0.324350
Thermal correction to Gibbs Free Energy= 0.262104
Sum of electronic and zero-point Energies= -707.555966
Sum of electronic and thermal Energies= -707.537453
Sum of electronic and thermal Enthalpies= -707.536509
Sum of electronic and thermal Free Energies= -707.598755

	E (Thermal)	CV	S
	KCal/Mol	Cal/Mol-Kelvin	Cal/Mol-Kelvin
Total	202.940	94.329	131.007

	1	2	3
	A(A1U)	A(E1U)	A(E1U)
Frequencies --	22.3765	62.7683	94.7981

Part 6. $[Ag(B_{11}H_{11})_2]^{3-}$: η^2,η^2; PG C_2

Ag	0.00000000	0.00000000	0.72026200
B	-1.20382000	3.48722000	-1.34278600
B	0.14961800	2.50207600	-1.24964700
B	1.26905000	1.95862600	0.17138200
B	0.15831700	2.29968800	1.39119700
B	-1.20169500	2.91774300	0.23381200
B	1.45882400	3.45530200	1.08950900
B	-0.22906300	3.98186800	1.32735400
B	-0.90185300	4.70958400	-0.17984600
B	0.27190900	4.34849400	-1.47004000
B	1.60544700	3.41859400	-0.68791700
B	0.83653400	4.81375600	0.14323400
B	1.20382000	-3.48722000	-1.34278600
B	1.20169500	-2.91774300	0.23381200
B	-0.15831700	-2.29968800	1.39119700
B	-1.26905000	-1.95862600	0.17138200
B	-0.14961800	-2.50207600	-1.24964700
B	-1.45882400	-3.45530200	1.08950900
B	-1.60544700	-3.41859400	-0.68791700
B	-0.27190900	-4.34849400	-1.47004000
B	0.90185300	-4.70958400	-0.17984600
B	0.22906300	-3.98186800	1.32735400
B	-0.83653400	-4.81375600	0.14323400
H	-2.09638000	3.37866800	-2.12500800
H	0.15053200	1.61122600	-2.04665600
H	2.09580000	1.10449500	0.00579000
H	-0.12032300	1.78244400	2.43787400
H	-2.20114300	2.33566700	0.53728400
H	2.35677900	3.63133800	1.85806200
H	-0.65305300	4.46180500	2.33572400
H	-1.60728200	5.66826300	-0.06300400
H	0.54793100	5.00557300	-2.43038100
H	2.64025900	3.45004500	-1.28374700
H	1.35968300	5.87232400	0.32363600
H	2.09638000	-3.37866800	-2.12500800
H	2.20114300	-2.33566700	0.53728400
H	0.12032300	-1.78244400	2.43787400
H	-2.09580000	-1.10449500	0.00579000
H	-0.15053200	-1.61122600	-2.04665600
H	-2.35677900	-3.63133800	1.85806200
H	-2.64025900	-3.45004500	-1.28374700
H	-0.54793100	-5.00557300	-2.43038100
H	1.60728200	-5.66826300	-0.06300400
H	0.65305300	-4.46180500	2.33572400
H	-1.35968300	-5.87232400	0.32363600

E(RB3LYP) = -707.853129061

Zero-point correction= 0.300231 (Hartree/Particle)
Thermal correction to Energy= 0.321145
Thermal correction to Enthalpy= 0.322089
Thermal correction to Gibbs Free Energy= 0.250711
Sum of electronic and zero-point Energies= -707.552898
Sum of electronic and thermal Energies= -707.531984
Sum of electronic and thermal Enthalpies= -707.531040
Sum of electronic and thermal Free Energies= -707.602418

	E (Thermal)	CV	S
	KCal/Mol	Cal/Mol-Kelvin	Cal/Mol-Kelvin
Total	201.522	96.784	150.228

	1	2	3
	A(A)	A(A)	A(B)
Frequencies --	18.0495	21.2430	23.4202

Part 6. $[Au(B_{11}H_{11})_2]^{3-}$: η[5],η[5]; PG D_{5d}

Au	0.00000000	0.00000000	0.00000000
B	-1.66382200	1.57988200	0.00000000
B	-1.66473300	0.48827800	-1.50261400
B	-1.66455400	-1.27825400	-0.92884700
B	-1.66455400	-1.27825400	0.92884700
B	-1.66473300	0.48827800	1.50261400
B	-3.14681100	-1.51173700	0.00000000
B	-3.14714300	-0.46701400	1.43797300
B	-3.14703200	1.22343900	0.88853000
B	-3.14703200	1.22343900	-0.88853000
B	-3.14714300	-0.46701400	-1.43797300
B	-4.07653200	0.00001200	0.00000000
B	1.66382200	-1.57988200	0.00000000
B	1.66473300	-0.48827800	1.50261400
B	1.66455400	1.27825400	0.92884700
B	1.66455400	1.27825400	-0.92884700
B	1.66473300	-0.48827800	-1.50261400
B	3.14681100	1.51173700	0.00000000
B	3.14714300	0.46701400	-1.43797300
B	3.14703200	-1.22343900	-0.88853000
B	3.14703200	-1.22343900	0.88853000
B	3.14714300	0.46701400	1.43797300
B	4.07653200	-0.00001200	0.00000000
H	-1.22028500	2.68399500	0.00000000
H	-1.22356700	0.82983000	-2.55357300
H	-1.22333000	-2.17311000	-1.57718200
H	-1.22333000	-2.17311000	1.57718200
H	-1.22356700	0.82983000	2.55357300
H	-3.68946800	-2.57637200	0.00000000
H	-3.68922300	-0.79648900	2.45064500
H	-3.68914700	2.08492700	1.51441900
H	-3.68914700	2.08492700	-1.51441900
H	-3.68922300	-0.79648900	-2.45064500
H	-5.27072000	-0.00005300	0.00000000
H	1.22028500	-2.68399500	0.00000000
H	1.22356700	-0.82983000	2.55357300
H	1.22333000	2.17311000	1.57718200
H	1.22333000	2.17311000	-1.57718200
H	1.22356700	-0.82983000	-2.55357300
H	3.68946800	2.57637200	0.00000000
H	3.68922300	0.79648900	-2.45064500
H	3.68914700	-2.08492700	-1.51441900
H	3.68914700	-2.08492700	1.51441900
H	3.68922300	0.79648900	2.45064500
H	5.27072000	0.00005300	0.00000000

E(RB3LYP) = -696.704782538

Zero-point correction= 0.306621 (Hartree/Particle)
Thermal correction to Energy= 0.324723
Thermal correction to Enthalpy= 0.325667
Thermal correction to Gibbs Free Energy= 0.264233
Sum of electronic and zero-point Energies= -696.398161
Sum of electronic and thermal Energies= -696.380059
Sum of electronic and thermal Enthalpies= -696.379115
Sum of electronic and thermal Free Energies= -696.440550

	E (Thermal)	CV	S
	KCal/Mol	Cal/Mol-Kelvin	Cal/Mol-Kelvin
Total	203.767	93.112	129.301

	1	2	3
	A(A1U)	A(E1U)	A(E1U)
Frequencies --	30.5766	74.3253	93.6026

Part 6. $[Au(B_{11}H_{11})_2]^{3-}$: η^2,η^2; PG C_2

Au	0.00000000	0.00000000	0.47285000
B	-1.51548500	3.29651000	-1.03267400
B	-0.14861200	2.36424300	-1.33805500
B	1.31356300	1.79509600	-0.19299400
B	0.50577600	2.09971000	1.29683200
B	-1.15032800	2.73965100	0.51111400
B	1.64530800	3.29665000	0.67584500
B	0.05592400	3.78392900	1.32238200
B	-0.97061100	4.51958800	0.04115200
B	-0.13665400	4.20647600	-1.49956700
B	1.35678500	3.29615700	-1.08330000
B	0.79128800	4.65618800	-0.06066600
B	1.51548500	-3.29651000	-1.03267400
B	1.15032800	-2.73965100	0.51111400
B	-0.50577600	-2.09971000	1.29683200
B	-1.31356300	-1.79509600	-0.19299400
B	0.14861200	-2.36424300	-1.33805500
B	-1.64530800	-3.29665000	0.67584500
B	-1.35678500	-3.29615700	-1.08330000
B	0.13665400	-4.20647600	-1.49956700
B	0.97061100	-4.51958800	0.04115200
B	-0.05592400	-3.78392900	1.32238200
B	-0.79128800	-4.65618800	-0.06066600
H	-2.56837100	3.18097100	-1.57905800
H	-0.31908300	1.51619600	-2.15954000
H	2.16914000	1.05048800	-0.57110200
H	0.55378500	1.65449200	2.40606200
H	-2.05090600	2.16398800	1.04293000
H	2.69801200	3.49484900	1.20450500
H	-0.10054300	4.23227700	2.41752800
H	-1.64556300	5.45904800	0.34159300
H	-0.11830400	4.88572800	-2.48288700
H	2.23214400	3.35970100	-1.89244400
H	1.33042000	5.71845800	0.01592800
H	2.56837100	-3.18097100	-1.57905800
H	2.05090600	-2.16398800	1.04293000
H	-0.55378400	-1.65449200	2.40606200
H	-2.16914000	-1.05048700	-0.57110200
H	0.31908300	-1.51619600	-2.15954000
H	-2.69801200	-3.49484900	1.20450500
H	-2.23214400	-3.35970100	-1.89244400
H	0.11830400	-4.88572800	-2.48288700
H	1.64556300	-5.45904800	0.34159300
H	0.10054300	-4.23227700	2.41752800
H	-1.33042000	-5.71845800	0.01592800

E(RB3LYP) = -696.637866624

Zero-point correction= 0.301241 (Hartree/Particle)
Thermal correction to Energy= 0.321652
Thermal correction to Enthalpy= 0.322596
Thermal correction to Gibbs Free Energy= 0.253156
Sum of electronic and zero-point Energies= -696.336626
Sum of electronic and thermal Energies= -696.316215
Sum of electronic and thermal Enthalpies= -696.315271
Sum of electronic and thermal Free Energies= -696.384710

	E (Thermal)	CV	S
	KCal/Mol	Cal/Mol-Kelvin	Cal/Mol-Kelvin
Total	201.840	96.633	146.148

	1	2	3
	A(A)	A(B)	A(A)
Frequencies --	17.8802	31.1284	40.6432

Part 6. $[Cu(B_{11}F_{11})_2]^{3-}$: η^5,η^5; PG D_{5d}

Cu	0.00000000	0.00000000	0.00000000
B	-1.51682700	1.58272400	0.00000000
B	-1.51577100	0.49009600	-1.50425900
B	-1.51530600	-1.27907800	-0.93039300
B	-1.51530600	-1.27907800	0.93039300
B	-1.51577100	0.49009600	1.50425900
B	-3.02186800	-1.52397200	0.00000000
B	-3.02250500	-0.47048200	1.44957600
B	-3.02334800	1.23312500	0.89579500
B	-3.02334800	1.23312500	-0.89579500
B	-3.02250500	-0.47048200	-1.44957600
B	-3.97405200	-0.00041200	0.00000000
B	1.51682700	-1.58272400	0.00000000
B	1.51577100	-0.49009600	1.50425900
B	1.51530600	1.27907800	0.93039300
B	1.51530600	1.27907800	-0.93039300
B	1.51577100	-0.49009600	-1.50425900
B	3.02186800	1.52397200	0.00000000
B	3.02250500	0.47048200	-1.44957600
B	3.02334800	-1.23312500	-0.89579500
B	3.02334800	-1.23312500	0.89579500
B	3.02250500	0.47048200	1.44957600
B	3.97405200	0.00041200	0.00000000
F	-1.11613200	2.89452000	0.00000000
F	-1.11211100	0.89722700	-2.75028700
F	-1.11231900	-2.33911300	-1.70172800
F	-1.11231900	-2.33911300	1.70172800
F	-1.11211100	0.89722700	2.75028700
F	-3.63973100	-2.75701000	0.00000000
F	-3.64080100	-0.85134900	2.62206900
F	-3.64134900	2.23039500	1.62076300
F	-3.64134900	2.23039500	-1.62076300
F	-3.64080100	-0.85134900	-2.62206900
F	-5.34351900	-0.00117300	0.00000000
F	1.11613200	-2.89452000	0.00000000
F	1.11211100	-0.89722700	2.75028700
F	1.11231900	2.33911300	1.70172800
F	1.11231900	2.33911300	-1.70172800
F	1.11211100	-0.89722700	-2.75028700
F	3.63973100	2.75701000	0.00000000
F	3.64080100	0.85134900	-2.62206900
F	3.64134900	-2.23039500	-1.62076300
F	3.64134900	-2.23039500	1.62076300
F	3.64080100	0.85134900	2.62206900
F	5.34351900	0.00117300	0.00000000

E(RB3LYP) = -2943.63575886

Zero-point correction= 0.161377 (Hartree/Particle)
Thermal correction to Energy= 0.202920
Thermal correction to Enthalpy= 0.203864
Thermal correction to Gibbs Free Energy= 0.096567
Sum of electronic and zero-point Energies= -2943.474382
Sum of electronic and thermal Energies= -2943.432839
Sum of electronic and thermal Enthalpies= -2943.431895
Sum of electronic and thermal Free Energies= -2943.539192

	E (Thermal)	CV	S
	KCal/Mol	Cal/Mol-Kelvin	Cal/Mol-Kelvin
Total	127.334	160.170	225.826

	1	2	3
	A(A1U)	A(E1U)	A(E1U)
Frequencies --	54.3258	56.3664	58.8494

Part 6. $[Cu(B_{11}F_{11})_2]^{3-}$: η^2,η^2; PG C_2

Cu	0.00000000	0.00000000	0.17529900
B	1.64635100	2.86179300	-0.74498200
B	0.23213800	2.17903400	-1.43292300
B	-1.41230400	1.59858000	-0.29110100
B	-0.77512200	1.72868500	1.26895900
B	1.17008200	2.35113800	0.81866300
B	-1.66861200	3.12389600	0.60551000
B	-0.12558600	3.38608900	1.47603800
B	1.14759500	4.11093600	0.32545700
B	0.46461300	3.97902000	-1.31121900
B	-1.20430400	3.18156100	-1.11958600
B	-0.60162300	4.42802000	0.06349800
B	-1.64635100	-2.86179300	-0.74498200
B	-1.17008200	-2.35113800	0.81866300
B	0.77512200	-1.72868500	1.26895900
B	1.41230400	-1.59858000	-0.29110100
B	-0.23213800	-2.17903300	-1.43292300
B	1.66861200	-3.12389600	0.60551000
B	1.20430400	-3.18156000	-1.11958600
B	-0.46461300	-3.97902000	-1.31121900
B	-1.14759500	-4.11093600	0.32545700
B	0.12558600	-3.38608900	1.47603800
B	0.60162300	-4.42802000	0.06349800
F	2.91601800	2.64490400	-1.26046200
F	0.36601100	1.38803800	-2.53367700
F	-2.44625700	0.89733100	-0.87419500
F	-1.06439400	1.18649900	2.49870800
F	2.07855100	1.70765600	1.60445800
F	-2.91558300	3.47366000	1.09305500
F	-0.10195200	3.84475600	2.77931600
F	1.93914500	5.11839700	0.83227700
F	0.61724700	4.86073700	-2.35977000
F	-2.13628800	3.45670200	-2.10139800
F	-1.07305400	5.71685200	0.15981700
F	-2.91601800	-2.64490400	-1.26046200
F	-2.07855100	-1.70765600	1.60445800
F	1.06439400	-1.18649900	2.49870800
F	2.44625700	-0.89733100	-0.87419500
F	-0.36601100	-1.38803800	-2.53367700
F	2.91558300	-3.47366000	1.09305500
F	2.13628800	-3.45670200	-2.10139800
F	-0.61724700	-4.86073700	-2.35977000
F	-1.93914500	-5.11839700	0.83227700
F	0.10195200	-3.84475600	2.77931600
F	1.07305400	-5.71685200	0.15981700

E(RB3LYP) = -2943.57465830

Zero-point correction= 0.158460 (Hartree/Particle)
Thermal correction to Energy= 0.202452
Thermal correction to Enthalpy= 0.203396
Thermal correction to Gibbs Free Energy= 0.085101
Sum of electronic and zero-point Energies= -2943.416198
Sum of electronic and thermal Energies= -2943.372207
Sum of electronic and thermal Enthalpies= -2943.371262
Sum of electronic and thermal Free Energies= -2943.489557

	E (Thermal)	CV	S
	KCal/Mol	Cal/Mol-Kelvin	Cal/Mol-Kelvin
Total	127.040	162.100	248.972

	1	2	3
	A(B)	A(A)	A(A)
Frequencies --	18.8107	26.9187	29.9035

Part 6. $[Ag(B_{11}F_{11})_2]^{3-}$: η^5,η^5; PG D_{5d}

Ag	0.00000000	0.00000000	0.00000000
B	-1.68928900	1.59680900	0.00000000
B	-1.68866500	0.49341800	1.51818800
B	-1.68892000	-1.29107700	0.93871600
B	-1.68892000	-1.29107700	-0.93871600
B	-1.68866500	0.49341800	-1.51818800
B	-3.18933400	-1.52709900	0.00000000
B	-3.18973600	-0.47166400	-1.45221700
B	-3.18972900	1.23530500	-0.89760400
B	-3.18972900	1.23530500	0.89760400
B	-3.18973600	-0.47166400	1.45221700
B	-4.13725500	-0.00015200	0.00000000
B	1.68928900	-1.59680900	0.00000000
B	1.68866500	-0.49341800	-1.51818800
B	1.68892000	1.29107700	-0.93871600
B	1.68892000	1.29107700	0.93871600
B	1.68866500	-0.49341800	1.51818800
B	3.18933400	1.52709900	0.00000000
B	3.18973600	0.47166400	1.45221700
B	3.18972900	-1.23530500	0.89760400
B	3.18972900	-1.23530500	-0.89760400
B	3.18973600	0.47166400	-1.45221700
B	4.13725500	0.00015200	0.00000000
F	-1.26697900	2.90294700	0.00000000
F	-1.26288000	0.89637100	2.75937700
F	-1.26332500	-2.34609900	1.70668200
F	-1.26332500	-2.34609900	-1.70668200
F	-1.26288000	0.89637100	-2.75937700
F	-3.81626100	-2.75601300	0.00000000
F	-3.81702200	-0.85124800	-2.62081800
F	-3.81641000	2.22940600	-1.62027100
F	-3.81641000	2.22940600	1.62027100
F	-3.81702200	-0.85124800	2.62081800
F	-5.50804600	-0.00042300	0.00000000
F	1.26697900	-2.90294700	0.00000000
F	1.26288000	-0.89637100	-2.75937700
F	1.26332500	2.34609900	-1.70668200
F	1.26332500	2.34609900	1.70668200
F	1.26288000	-0.89637100	2.75937700
F	3.81626100	2.75601300	0.00000000
F	3.81702200	0.85124800	2.62081800
F	3.81641000	-2.22940600	1.62027100
F	3.81641000	-2.22940600	-1.62027100
F	3.81702200	0.85124800	-2.62081800
F	5.50804600	0.00042300	0.00000000

E(RB3LYP) = -2893.22624583

Zero-point correction= 0.159694 (Hartree/Particle)
Thermal correction to Energy= 0.202165
Thermal correction to Enthalpy= 0.203109
Thermal correction to Gibbs Free Energy= 0.091733
Sum of electronic and zero-point Energies= -2893.066552
Sum of electronic and thermal Energies= -2893.024081
Sum of electronic and thermal Enthalpies= -2893.023137
Sum of electronic and thermal Free Energies= -2893.134513

	E (Thermal)	CV	S
	KCal/Mol	Cal/Mol-Kelvin	Cal/Mol-Kelvin
Total	126.860	161.229	234.411

	1	2	3
	A(A1U)	A(E1U)	A(E1U)
Frequencies --	28.6140	47.6000	47.6583
Red. masses --	17.1343	18.7589	18.7602

Part 6. $[Ag(B_{11}F_{11})_2]^{3-}$: η^2,η^2; PG C_2

Ag	0.00000000	0.00000000	0.17875900
B	1.70790700	3.27012200	-0.67149400
B	0.33888300	2.55547200	-1.39944200
B	-1.35721300	1.94640400	-0.37309100
B	-0.79605600	2.05867000	1.20562700
B	1.14501500	2.71060600	0.83858700
B	-1.67740000	3.45917500	0.52915800
B	-0.17755000	3.72096100	1.46957100
B	1.14020800	4.48322900	0.39932100
B	0.53596100	4.36521600	-1.28030000
B	-1.12548800	3.53866000	-1.16968400
B	-0.59873800	4.77912400	0.05177600
B	-1.70790700	-3.27012200	-0.67149400
B	-1.14501500	-2.71060600	0.83858700
B	0.79605600	-2.05867000	1.20562700
B	1.35721300	-1.94640400	-0.37309100
B	-0.33888300	-2.55547200	-1.39944200
B	1.67740000	-3.45917500	0.52915800
B	1.12548800	-3.53866000	-1.16968400
B	-0.53596100	-4.36521600	-1.28030000
B	-1.14020800	-4.48322900	0.39932100
B	0.17755000	-3.72096100	1.46957100
B	0.59873800	-4.77912400	0.05177600
F	3.00112900	3.04784800	-1.12315100
F	0.54473700	1.73471900	-2.47251300
F	-2.34537900	1.24734100	-1.03396100
F	-1.11320000	1.49085100	2.42089600
F	2.01681200	2.01891300	1.63114000
F	-2.94995800	3.79128900	0.96152600
F	-0.21680600	4.15246900	2.78326800
F	1.88396600	5.50038600	0.95972900
F	0.71296200	5.27093700	-2.30546500
F	-2.00880000	3.80654200	-2.19956000
F	-1.08871200	6.06321000	0.13963900
F	-3.00112900	-3.04784800	-1.12315100
F	-2.01681200	-2.01891300	1.63114000
F	1.11320000	-1.49085100	2.42089600
F	2.34537900	-1.24734100	-1.03396100
F	-0.54473700	-1.73471900	-2.47251300
F	2.94995800	-3.79128900	0.96152600
F	2.00880000	-3.80654200	-2.19956000
F	-0.71296200	-5.27093700	-2.30546500
F	-1.88396600	-5.50038600	0.95972900
F	0.21680600	-4.15246900	2.78326800
F	1.08871200	-6.06321000	0.13963900

E(RB3LYP) = -2893.19296567
Zero-point correction= 0.157937 (Hartree/Particle)
Thermal correction to Energy= 0.202218
Thermal correction to Enthalpy= 0.203162
Thermal correction to Gibbs Free Energy= 0.082920
Sum of electronic and zero-point Energies= -2893.035028
Sum of electronic and thermal Energies= -2892.990748
Sum of electronic and thermal Enthalpies= -2892.989804
Sum of electronic and thermal Free Energies= -2893.110046

	E (Thermal)	CV	S
	KCal/Mol	Cal/Mol-Kelvin	Cal/Mol-Kelvin
Total	126.893	162.301	253.070

	1	2	3
	A(A)	A(B)	A(A)
Frequencies --	9.7032	15.5773	23.3813

Part 6. $[Au(B_{11}F_{11})_2]^{3-}$: η^5,η^5; PG D_{5d}

Au	0.00000000	0.00000000	0.00000000
B	-1.66826200	1.60590800	0.00000000
B	-1.66768800	0.49625700	1.52683400
B	-1.66782400	-1.29830300	0.94395600
B	-1.66782400	-1.29830300	-0.94395600
B	-1.66768800	0.49625700	-1.52683400
B	-3.16454400	-1.52589500	0.00000000
B	-3.16492000	-0.47132500	-1.45113900
B	-3.16494000	1.23437500	-0.89691600
B	-3.16494000	1.23437500	0.89691600
B	-3.16492000	-0.47132500	1.45113900
B	-4.10838400	-0.00015400	0.00000000
B	1.66826200	-1.60590800	0.00000000
B	1.66768800	-0.49625700	-1.52683400
B	1.66782400	1.29830300	-0.94395600
B	1.66782400	1.29830300	0.94395600
B	1.66768800	-0.49625700	1.52683400
B	3.16454400	1.52589500	0.00000000
B	3.16492000	0.47132500	1.45113900
B	3.16494000	-1.23437500	0.89691600
B	3.16494000	-1.23437500	-0.89691600
B	3.16492000	0.47132500	-1.45113900
B	4.10838400	0.00015400	0.00000000
F	-1.25243500	2.91287500	0.00000000
F	-1.24843600	0.89958400	2.76880000
F	-1.24841400	-2.35408000	1.71211500
F	-1.24841400	-2.35408000	-1.71211500
F	-1.24843600	0.89958400	-2.76880000
F	-3.79579200	-2.75359000	0.00000000
F	-3.79658600	-0.85056700	-2.61855200
F	-3.79596800	2.22748900	-1.61886500
F	-3.79596800	2.22748900	1.61886500
F	-3.79658600	-0.85056700	2.61855200
F	-5.48143900	-0.00043400	0.00000000
F	1.25243500	-2.91287500	0.00000000
F	1.24843600	-0.89958400	-2.76880000
F	1.24841400	2.35408000	-1.71211500
F	1.24841400	2.35408000	1.71211500
F	1.24843600	-0.89958400	2.76880000
F	3.79579200	2.75359000	0.00000000
F	3.79658600	0.85056700	2.61855200
F	3.79596800	-2.22748900	1.61886500
F	3.79596800	-2.22748900	-1.61886500
F	3.79658600	0.85056700	-2.61855200
F	5.48143900	0.00043400	0.00000000

E(RB3LYP) = -2882.06586758

Zero-point correction= 0.160631 (Hartree/Particle)
Thermal correction to Energy= 0.202841
Thermal correction to Enthalpy= 0.203785
Thermal correction to Gibbs Free Energy= 0.093013
Sum of electronic and zero-point Energies= -2881.905236
Sum of electronic and thermal Energies= -2881.863027
Sum of electronic and thermal Enthalpies= -2881.862082
Sum of electronic and thermal Free Energies= -2881.972854

E (Thermal)	CV	S
KCal/Mol	Cal/Mol-Kelvin	Cal/Mol-Kelvin

	1	2	3
	A(A1U)	A(E1U)	A(E1U)
Frequencies --	31.4723	48.9871	50.2145

Part 6. $[Au(B_{11}F_{11})_2]^{3-}$: η^2,η^2; PG C_{2h}

Au	0.00000000	0.00000000	-0.00012800
B	-1.87107000	3.15388600	0.00014700
B	-0.86591200	2.55002900	1.24649600
B	1.12089400	1.85354200	0.86065400
B	1.12076400	1.85361000	-0.86092500
B	-0.86610700	2.55012500	-1.24640500
B	1.70429100	3.29658200	-0.00012400
B	0.61374900	3.50042700	-1.41532200
B	-0.96676700	4.32438400	-0.88979700
B	-0.96662800	4.32431600	0.89004000
B	0.61396800	3.50031900	1.41525600
B	0.56201100	4.64387600	0.00001500
B	1.87107000	-3.15388600	0.00014700
B	0.86591200	-2.55002900	1.24649600
B	-1.12089400	-1.85354200	0.86065400
B	-1.12076300	-1.85361000	-0.86092500
B	0.86610700	-2.55012500	-1.24640500
B	-1.70429100	-3.29658200	-0.00012400
B	-0.61374900	-3.50042700	-1.41532200
B	0.96676700	-4.32438400	-0.88979700
B	0.96662800	-4.32431600	0.89004000
B	-0.61396800	-3.50031900	1.41525600
B	-0.56201100	-4.64387500	0.00001500
F	-3.24889600	2.97306900	0.00024800
F	-1.42778000	1.86194300	2.27577800
F	1.89073600	1.28823900	1.84667400
F	1.89045800	1.28838500	-1.84710500
F	-1.42813800	1.86212000	-2.27565300
F	3.05045700	3.61615200	-0.00021500
F	1.12498100	3.86268200	-2.64740700
F	-1.47282300	5.30250000	-1.71800600
F	-1.47255600	5.30236800	1.71840300
F	1.12539100	3.86247800	2.64728900
F	1.08789900	5.91383800	0.00002300
F	3.24889600	-2.97306900	0.00024800
F	1.42778000	-1.86194300	2.27577800
F	-1.89073600	-1.28823900	1.84667400
F	-1.89045800	-1.28838500	-1.84710500
F	1.42813800	-1.86212000	-2.27565300
F	-3.05045700	-3.61615200	-0.00021500
F	-1.12498100	-3.86268200	-2.64740700
F	1.47282300	-5.30250000	-1.71800600
F	1.47255600	-5.30236800	1.71840300
F	-1.12539100	-3.86247800	2.64728900
F	-1.08790000	-5.91383800	0.00002300

E(RB3LYP) = -2881.98472162

Zero-point correction= 0.157922 (Hartree/Particle)
Thermal correction to Energy= 0.201159
Thermal correction to Enthalpy= 0.202103
Thermal correction to Gibbs Free Energy= 0.086038
Sum of electronic and zero-point Energies= -2881.826800
Sum of electronic and thermal Energies= -2881.783563
Sum of electronic and thermal Enthalpies= -2881.782618
Sum of electronic and thermal Free Energies= -2881.898684

	E (Thermal)	CV	S
	KCal/Mol	Cal/Mol-Kelvin	Cal/Mol-Kelvin
Total	126.229	160.364	244.281

	1	2	3
	A(Au)	A(Bu)	A(Au)
Frequencies --	-17.3400	22.7574	30.2495
