# Supplementary material for: The Biicosahedral Complex Anions [M(B11H11)2]3− (M = Cu, Ag, Au): Synthesis and Unexpected Low‐Temperature Phase Transition of [Ag(η5‐B11H11)2]3− to [Ag(η2‐B11H11)2]3−
Source: Angew Chem Int Ed Engl. 2025 Nov 10;65(2):e19283. doi: 10.1002/anie.202519283 (PMC12790380; doi:10.1002/anie.202519283)
Supplement: Supplementary file 2 — Supporting Information [file ANIE-65-e19283-s001.zip › Au_MO.pdf]

Functional: B3LYP, SCRF(Solvent=Water) Basis set: B, H, F: 6-311++g(d,p); Au: SDD
Isovalue: 0.03

Alpha occ. eigenvalues --	-6.70543	-6.70538	-6.70535	-6.70533	-6.70532
Alpha occ. eigenvalues --	-6.70530	-6.70514	-6.70512	-6.70510	-6.70509
Alpha occ. eigenvalues --	-6.69040	-6.69038	-6.68862	-6.68860	-6.68854
Alpha occ. eigenvalues --	-6.68852	-6.68852	-6.68850	-6.68823	-6.68821
Alpha occ. eigenvalues --	-6.68821	-6.68819	-4.24976	-2.43255	-2.41299
Alpha occ. eigenvalues --	-2.41297	-0.72437	-0.71735	-0.59440	-0.59433
Alpha occ. eigenvalues --	-0.59027	-0.59020	-0.57171	-0.53604	-0.46853
Alpha occ. eigenvalues --	-0.46840	-0.45879	-0.45879	-0.44906	-0.44475
Alpha occ. eigenvalues --	-0.44474	-0.43381	-0.43364	-0.41995	-0.41846
Alpha occ. eigenvalues --	-0.39321	-0.39319	-0.38958	-0.37402	-0.36702
Alpha occ. eigenvalues --	-0.36700	-0.34932	-0.34922	-0.34897	-0.34896
Alpha occ. eigenvalues --	-0.34217	-0.34216	-0.33979	-0.33971	-0.31663
Alpha occ. eigenvalues --	-0.30948	-0.30113	-0.27389	-0.27386	-0.27304
Alpha occ. eigenvalues --	-0.27298	-0.27012	-0.26990	-0.26277	-0.26259
Alpha occ. eigenvalues --	-0.25666	-0.25664	-0.25601	-0.24690	-0.24688
Alpha occ. eigenvalues --	-0.21218	-0.21217			
Alpha virt. eigenvalues --	-0.05488	-0.05486	-0.00565	0.00684	0.01290

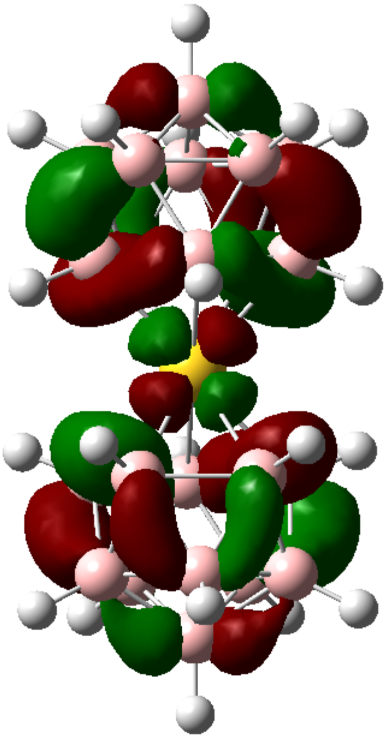

LUMO_00,01

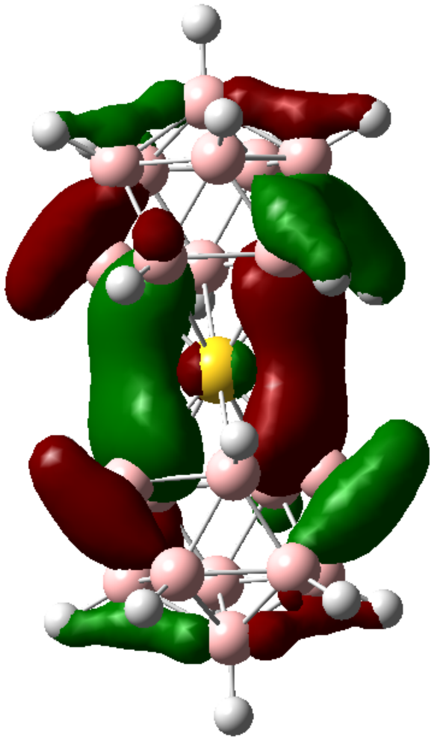

HOMO_00,01

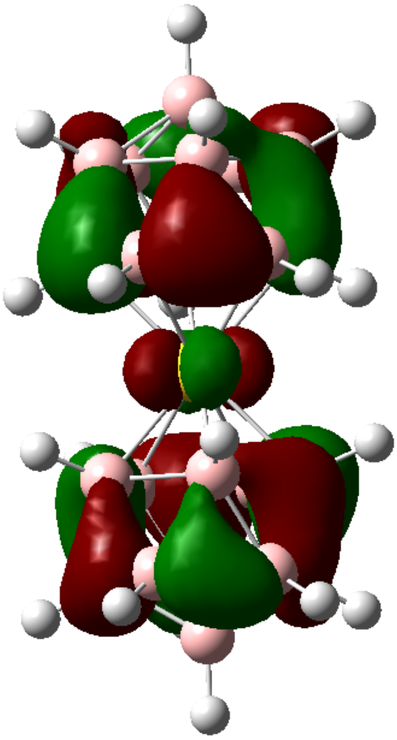

HOMO_02,03

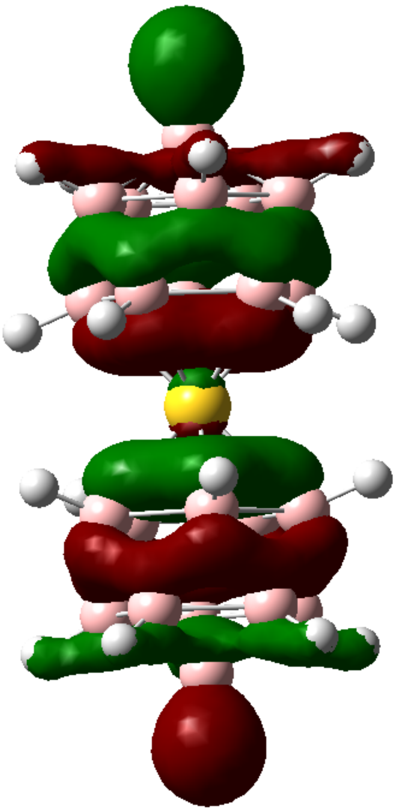

HOMO_04

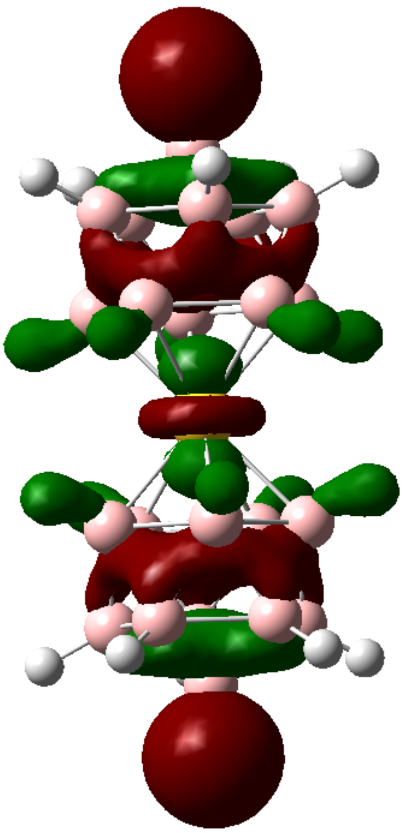

HOMO_15

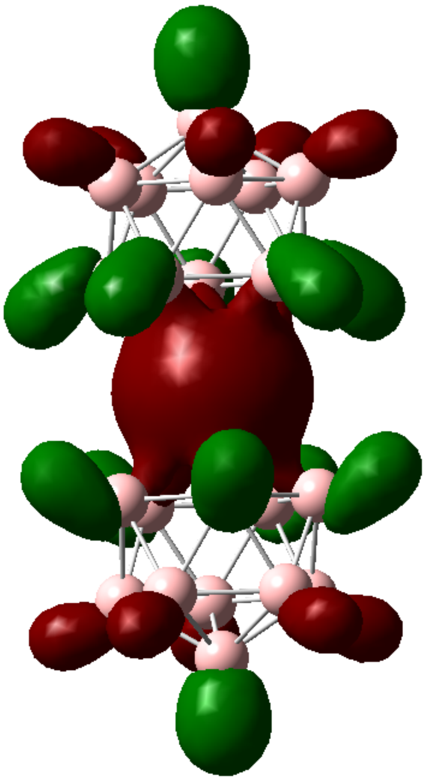

HOMO_16

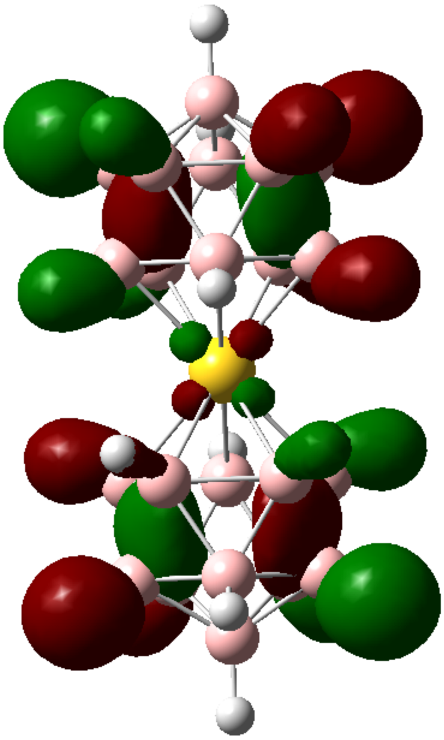

HOMO_18,19

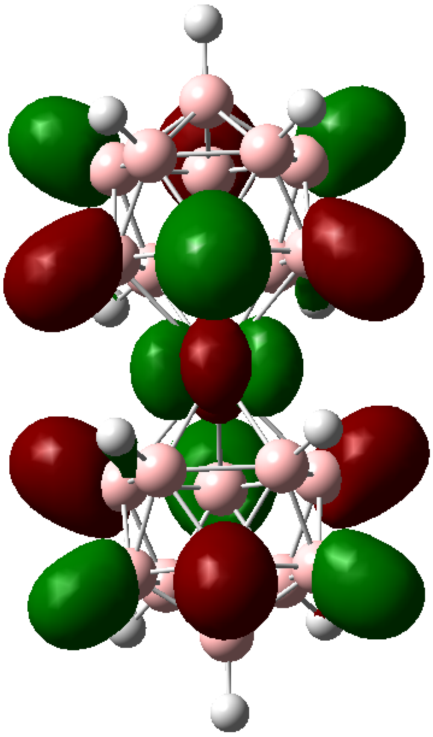

HOMO_20,21

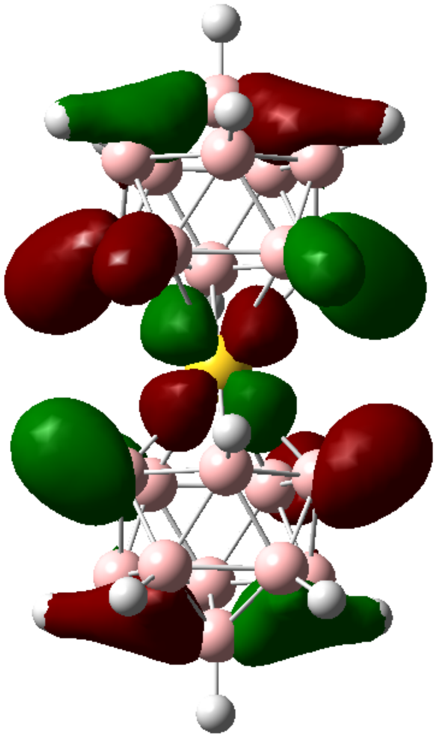

HOMO_26,27

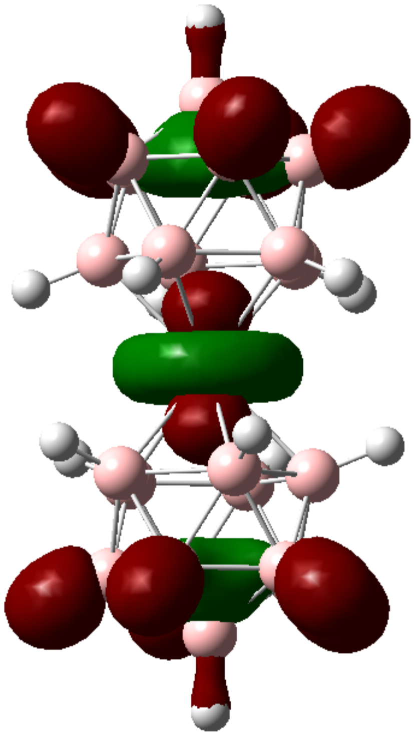

HOMO_29

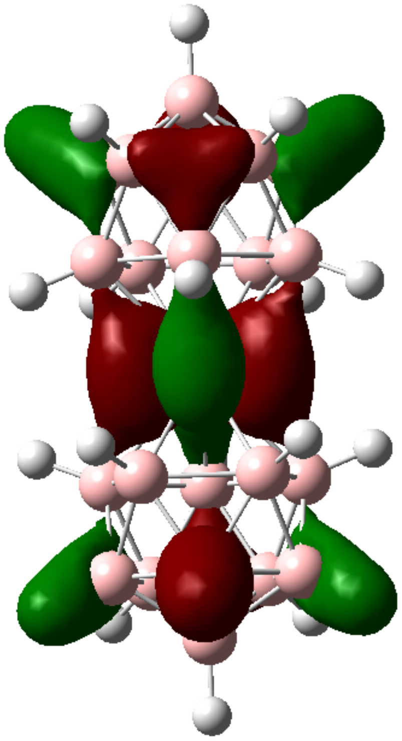

HOMO_30,31

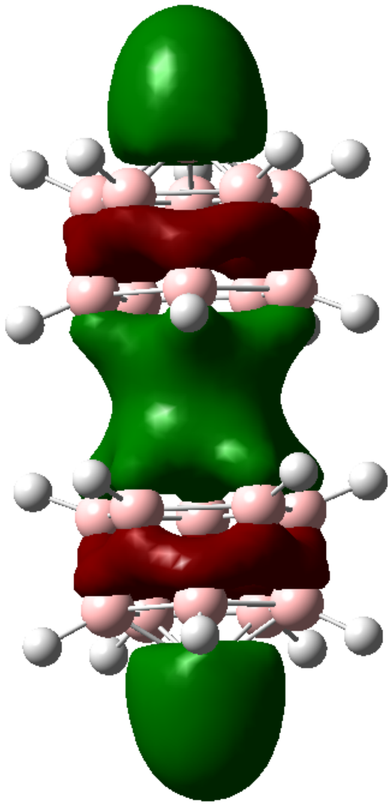

HOMO_32

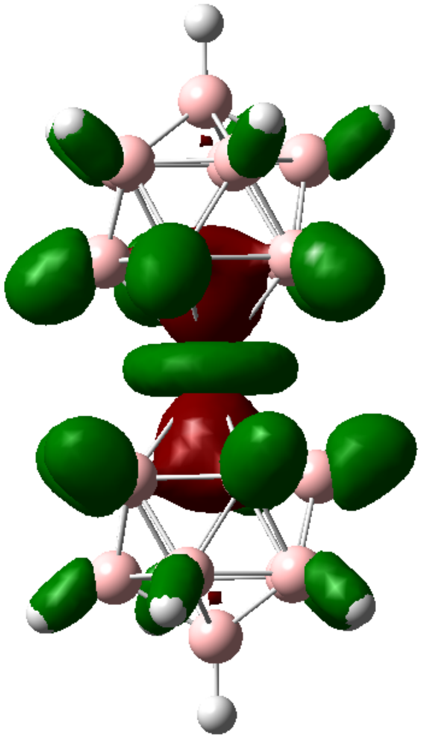

HOMO_38

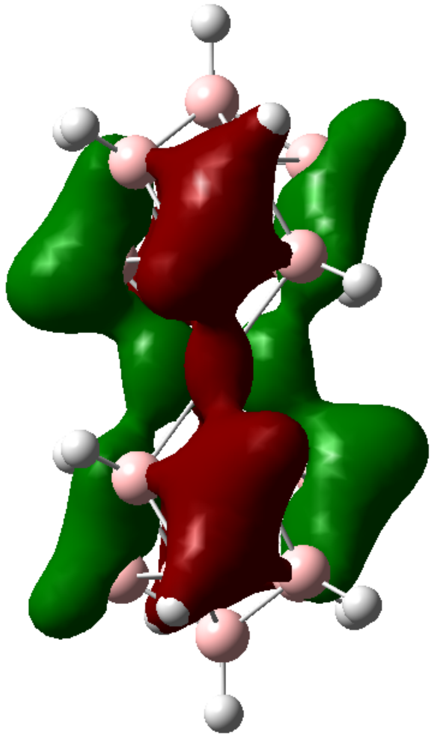

HOMO_39,40

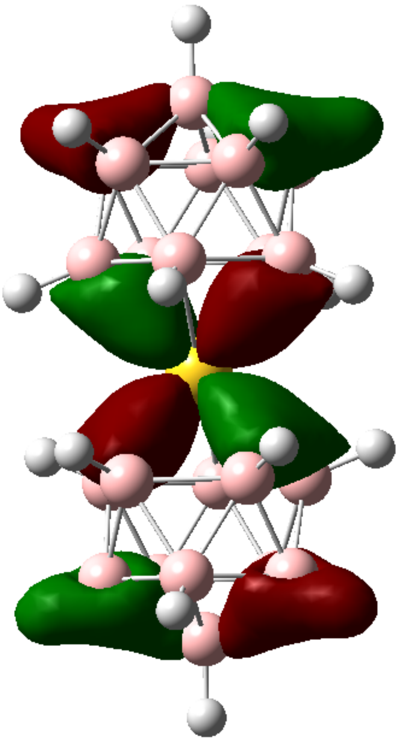

HOMO_41,42

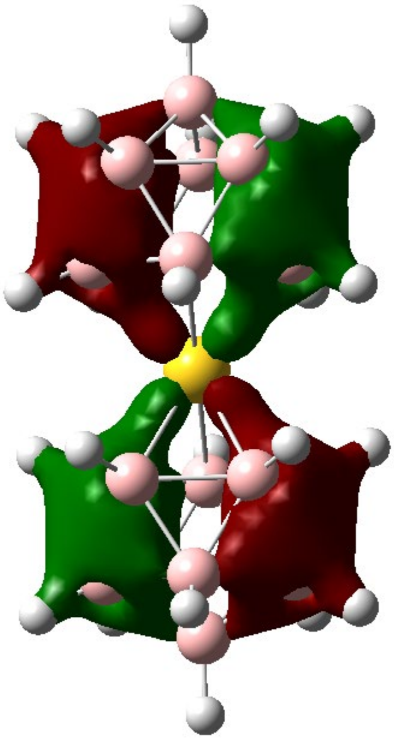

HOMO_47,48
