# Supplementary material for: The Biicosahedral Complex Anions [M(B11H11)2]3− (M = Cu, Ag, Au): Synthesis and Unexpected Low‐Temperature Phase Transition of [Ag(η5‐B11H11)2]3− to [Ag(η2‐B11H11)2]3−
Source: Angew Chem Int Ed Engl. 2025 Nov 10;65(2):e19283. doi: 10.1002/anie.202519283 (PMC12790380; doi:10.1002/anie.202519283)
Supplement: Supplementary file 2 — Supporting Information [file ANIE-65-e19283-s001.zip › Cu_MO.pdf]

Functional: B3LYP, SCRF(Solvent=Water) Basis set: B, H, F: 6-311++g(d,p); Cu: SDD
Isovalue: 0.03

```
 Alpha  occ. eigenvalues --   -6.69851  -6.69843  -6.69841  -6.69838  -6.69831
 Alpha  occ. eigenvalues --   -6.69828  -6.69819  -6.69817  -6.69807  -6.69805
 Alpha  occ. eigenvalues --   -6.69191  -6.69185  -6.68834  -6.68827  -6.68822
 Alpha  occ. eigenvalues --   -6.68822  -6.68815  -6.68815  -6.68792  -6.68791
 Alpha  occ. eigenvalues --   -6.68785  -6.68784  -4.61431  -2.91439  -2.89916
 Alpha  occ. eigenvalues --   -2.89915  -0.72144  -0.71659  -0.58926  -0.58925
 Alpha  occ. eigenvalues --   -0.58634  -0.58634  -0.56214  -0.53253  -0.45124
 Alpha  occ. eigenvalues --   -0.45114  -0.44059  -0.44051  -0.43680  -0.43666
 Alpha  occ. eigenvalues --   -0.43431  -0.42723  -0.42716  -0.41768  -0.40984
 Alpha  occ. eigenvalues --   -0.37759  -0.37225  -0.37217  -0.36911  -0.35199
 Alpha  occ. eigenvalues --   -0.35199  -0.34833  -0.34829  -0.34469  -0.34461
 Alpha  occ. eigenvalues --   -0.34079  -0.34074  -0.33456  -0.33455  -0.31767
 Alpha  occ. eigenvalues --   -0.31103  -0.29837  -0.27187  -0.27180  -0.27094
 Alpha  occ. eigenvalues --   -0.27093  -0.26969  -0.26967  -0.25903  -0.25898
 Alpha  occ. eigenvalues --   -0.25097  -0.25089  -0.25000  -0.24995  -0.24761
 Alpha  occ. eigenvalues --   -0.21292  -0.21285
 Alpha virt. eigenvalues --   -0.08056  -0.08051  -0.00744   0.00764   0.01425
```

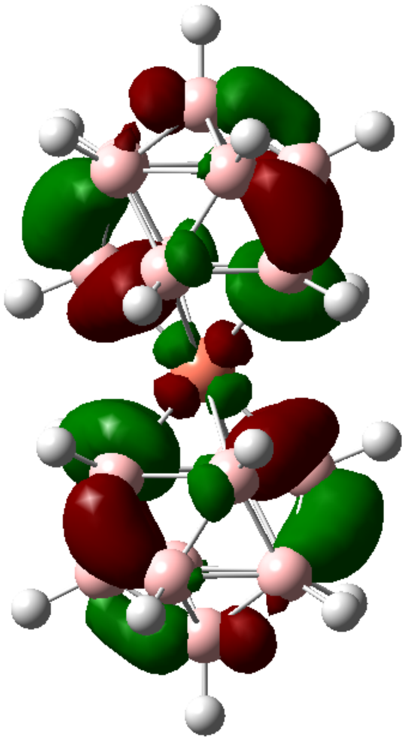

LUMO_00,01

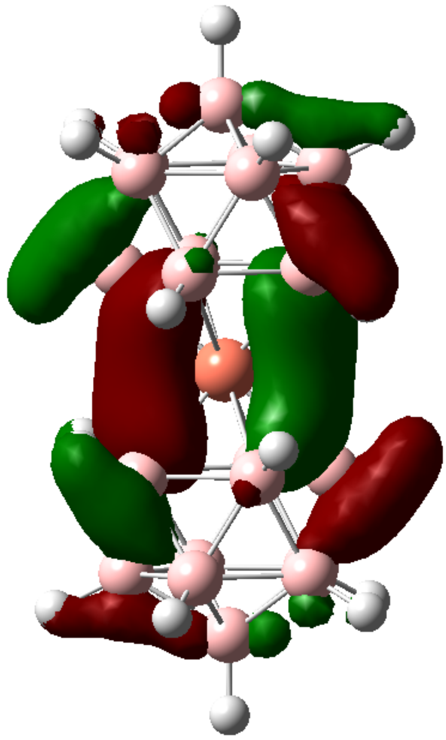

HOMO_00,01

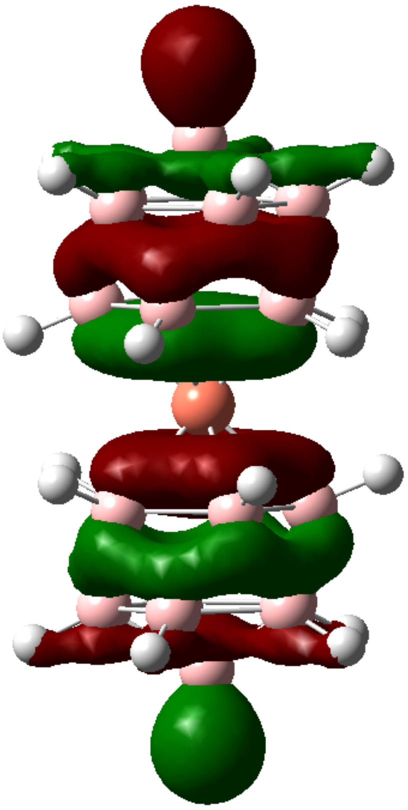

HOMO_02

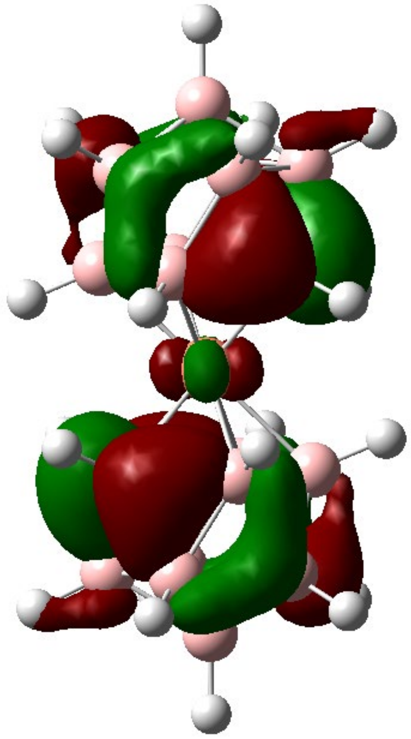

HOMO_03,04

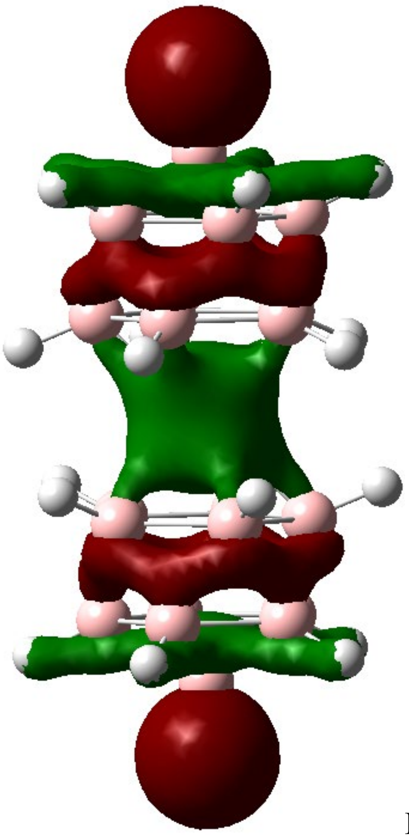

HOMO_15

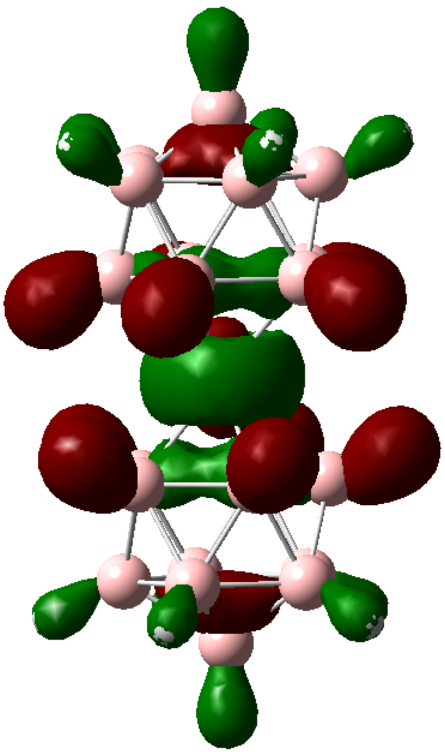

HOMO_17

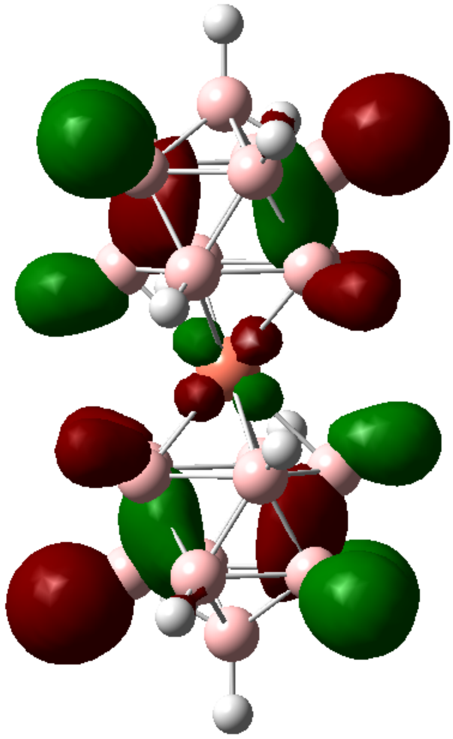

HOMO_18,19

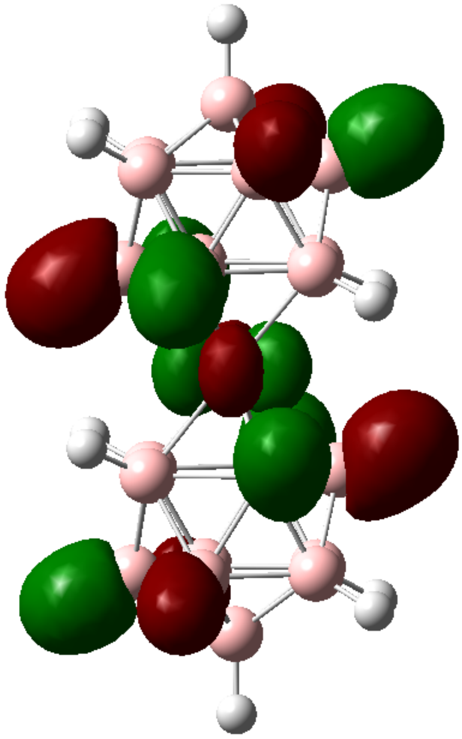

HOMO_20,21

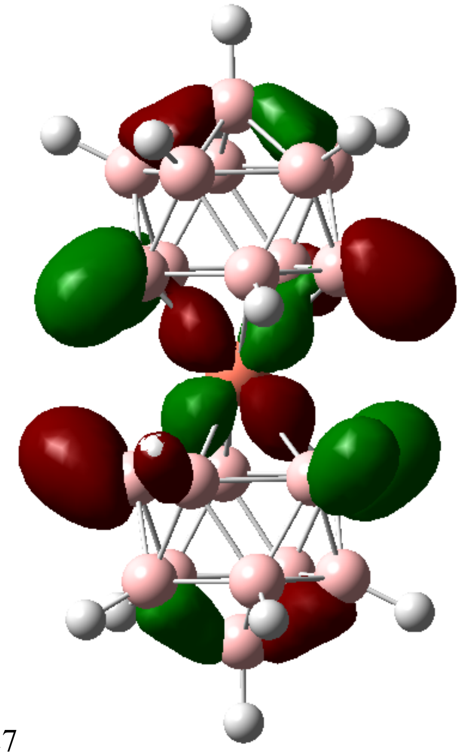

HOMO_26,27

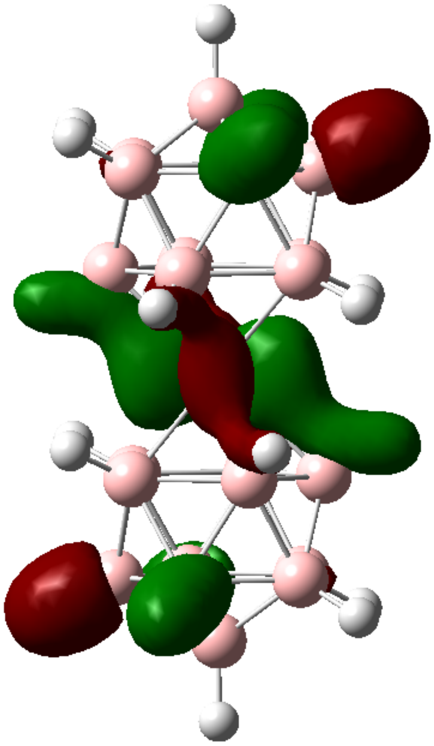

HOMO_29,30

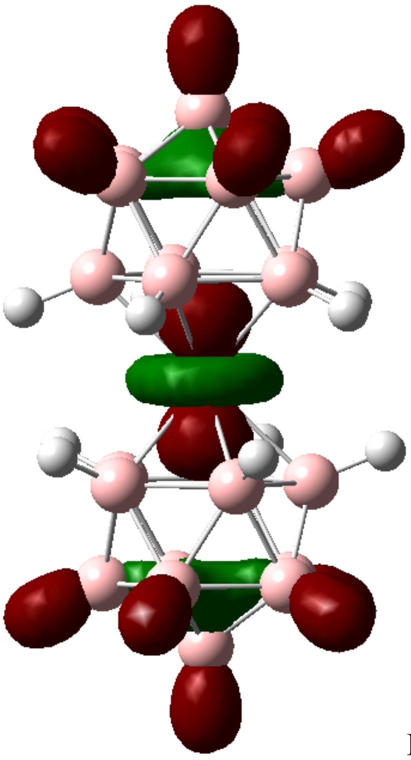

HOMO_31

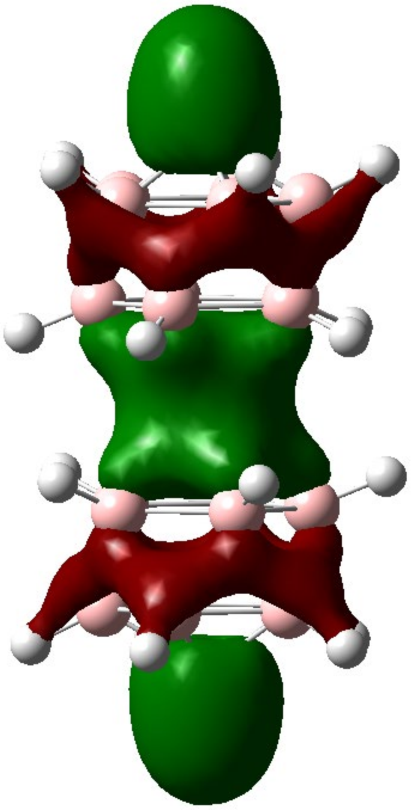

HOMO_32

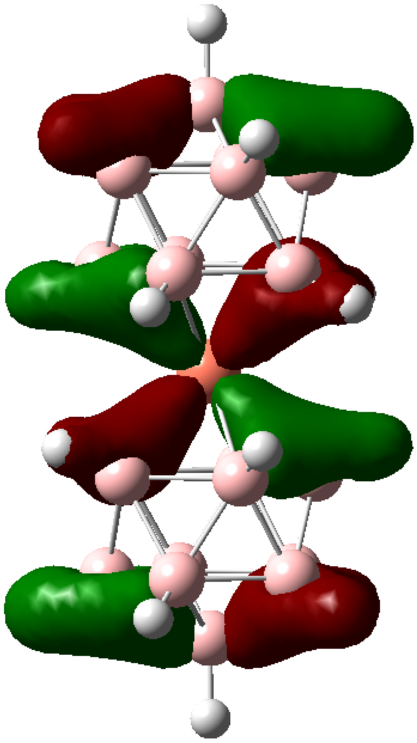

HOMO_34,35

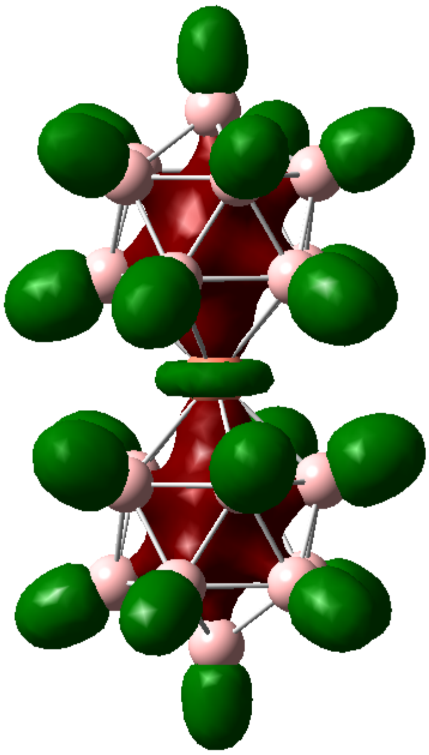

HOMO_36

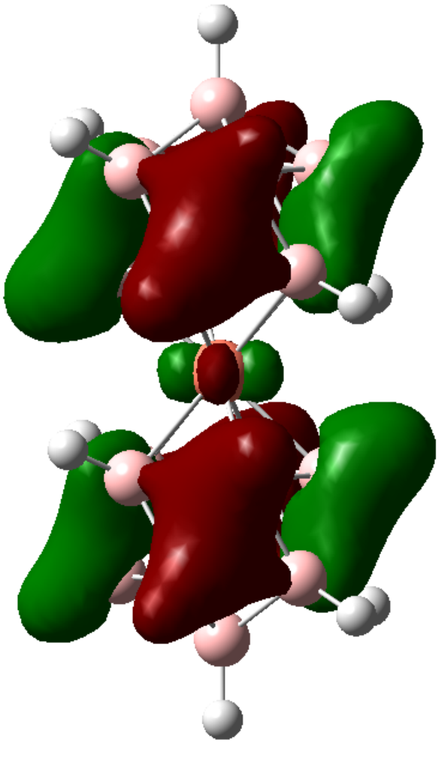

HOMO_41,42
